# Phosphoproteomic Profiling of Human Myocardial Tissues Distinguishes Ischemic from Non-Ischemic End Stage Heart Failure

**DOI:** 10.1371/journal.pone.0104157

**Published:** 2014-08-12

**Authors:** Matthew A. Schechter, Michael K. H. Hsieh, Linda W. Njoroge, J. Will Thompson, Erik J. Soderblom, Bryan J. Feger, Constantine D. Troupes, Kathleen A. Hershberger, Olga R. Ilkayeva, Whitney L. Nagel, Gina P. Landinez, Kishan M. Shah, Virginia A. Burns, Lucia Santacruz, Matthew D. Hirschey, Matthew W. Foster, Carmelo A. Milano, M. Arthur Moseley, Valentino Piacentino, Dawn E. Bowles

**Affiliations:** 1 Department of Surgery, Duke University Medical Center, Durham, North Carolina, United States of America; 2 Duke Proteomics Core, Duke University Medical Center, Durham, North Carolina, United States of America; 3 Sarah W. Stedman Nutrition and Metabolism Center, Duke University Medical Center, Durham, North Carolina, United States of America; 4 Department of Medicine, Duke University Medical Center, Durham, North Carolina, United States of America; 5 Duke Translational Research Institute, Duke University Medical Center, Durham, North Carolina, United States of America; 6 Division of Pulmonary, Allergy and Critical Care, Medicine, Duke University Medical Center, Durham, North Carolina, United States of America; Tokai University, Japan

## Abstract

The molecular differences between ischemic (IF) and non-ischemic (NIF) heart failure are poorly defined. A better understanding of the molecular differences between these two heart failure etiologies may lead to the development of more effective heart failure therapeutics. In this study extensive proteomic and phosphoproteomic profiles of myocardial tissue from patients diagnosed with IF or NIF were assembled and compared.

Proteins extracted from left ventricular sections were proteolyzed and phosphopeptides were enriched using titanium dioxide resin. Gel- and label-free nanoscale capillary liquid chromatography coupled to high resolution accuracy mass tandem mass spectrometry allowed for the quantification of 4,436 peptides (corresponding to 450 proteins) and 823 phosphopeptides (corresponding to 400 proteins) from the unenriched and phospho-enriched fractions, respectively.

Protein abundance did not distinguish NIF from IF. In contrast, 37 peptides (corresponding to 26 proteins) exhibited a ≥2-fold alteration in phosphorylation state (p<0.05) when comparing IF and NIF. The degree of protein phosphorylation at these 37 sites was specifically dependent upon the heart failure etiology examined. Proteins exhibiting phosphorylation alterations were grouped into functional categories: transcriptional activation/RNA processing; cytoskeleton structure/function; molecular chaperones; cell adhesion/signaling; apoptosis; and energetic/metabolism.

Phosphoproteomic analysis demonstrated profound post-translational differences in proteins that are involved in multiple cellular processes between different heart failure phenotypes. Understanding the roles these phosphorylation alterations play in the development of NIF and IF has the potential to generate etiology-specific heart failure therapeutics, which could be more effective than current therapeutics in addressing the growing concern of heart failure.

## Introduction

Despite earlier diagnosis and improved therapy, heart failure (HF) continues to be a major health concern. In 2012, 5.7 million Americans were diagnosed with HF [1]. The lifetime risk of developing HF after age 40 is 20%, with the annual incidence approaching 10 per 1000 people after age 65 [2]. More than half of all HF patients will die within a 5-year period of being diagnosed [3], [4]. These statistics are complicated by the fact that HF is a complex, multi-faceted disease that presents in two major forms: ischemic and non-ischemic.

Ischemic HF describes significantly impaired left ventricular function resulting from reduced blood supply to the heart muscle. In contrast, the reduced left ventricular function seen in non-ischemic HF has a range of etiologies, including congenital, infectious agents, autoimmune, and idiopathic. However, the current standard of care treats all cases of HF similarly, regardless of etiology. The treatment options for advanced heart failure are limited to implantation of a ventricular assist device to mechanically unload the heart, heart transplantation, or palliation with continuous intravenous inotropic support. These options, however, are associated with high morbidity and mortality, highlighting the continued need for improving HF treatment.

One such avenue of exploration is etiology-specific treatment. Such precise therapy would require an enhanced understanding of the molecular differences between heart failure phenotypes. Uncovering these molecular differences in a systematic and comprehensive way is made possible by utilizing high throughput ‘omics profiling [5], [6]. Genomics, proteomics, metabolomics, etc. have primarily focused on comparing the differences between one HF etiology and non-failing samples (reviewed by Gonzalez *et al* [7]). Recently, using two dimensional gel-based proteomics on tissue from diseased human hearts, Klawitter *et al* were able to demonstrate differences in the relative abundance of proteins in signaling pathways between ischemic or idiopathic cardiomyopathies [8]. However, differences in protein quantity represent only one aspect of the molecular picture; many of the key proteins involved in signal transduction pathways are highly regulated by post translational modification, such as phosphorylation. Whether protein phosphorylation patterns differ between heart failure etiologies is unknown. Such knowledge may enable the development of better and more precise heart failure therapeutics and diagnostics.

In the current investigation, cardiac tissues from a well-characterized and well-preserved human heart tissue bank were subjected to titanium dioxide resin to enrich for phosphopeptides, and then analyzed by a bottom-up LC/MS/MS global proteomics approach. This approach revealed amino acid residue-specific phosphorylation patterns on 400 cardiac proteins, which were compared between the IF and NIF etiologies. This revealed, for the first time, cardiac disease-specific phosphorylation pattern variations on key proteins involved in various aspects of cardiac physiology. Understanding how distinct protein phosphorylation patterns impact specific heart failure etiologies will support the development of therapeutics that better treat heart failure.

## Materials and Methods

### Human Cardiac Tissue Acquisition and Tissue Repository

The study protocol was approved by the Duke University Health System Institutional Review Board (DUHS IRB). The DUHS IRB, FWA #00009025, is duly constituted and complies with all U.S. regulatory requirements related to the protection of human research participants and the Guidelines of the International Conference on Harmonization (ICH) as adopted by the U.S. Food and Drug Administration. Tissue samples used for this study were procured from the Duke Human Heart Repository (DHHR), which is a DUHS IRB approved tissue repository. Samples were procured by the DHHR in accordance under an approved DUHS IRB protocol using written informed consent or a waiver of consent for discarded tissues. Some samples procured had been anonymized with all HIPAA identifiers removed, others were de-identified. Only DHHR personnel were privy to a key that could link the samples to patient information. No HIPAA information was provided with any of the samples used in this study. Human myocardium was acquired from the left ventricular free wall of explanted IF or NIF hearts following cardiac transplantation. Non-failing (NF) left ventricular tissue was acquired from donors whose hearts were not utilized for transplant, thus becoming available for research. After explantation, transmural tissue samples were processed and stored as described in the Methods S1.

### Sample Preparation for Mass Spectrometry

Heart tissue samples were homogenized and subjected to phase separation by addition of chloroform. Protein was precipitated from the organic layer, washed, sonicated, recovered by centrifugation, and re-suspended in mass spectrometry-compatible detergent (RapiGest, Waters Corp., Milford, MA). Cardiac tissue homogenates used for mass spectrometry were subjected to Bradford Assay for protein quantification. A 625 µg aliquot of protein (per sample) was subjected to reduction, alkylation, followed by overnight proteolysis with sequencing grade trypsin (Promega, Madison, WI). A 25 µg aliquot from each sample was used for unenriched proteomic analysis of protein expression in the heart tissue. This 25 µg was spiked with 1.25 pmol ADH1_YEAST digest (Massprep standard, Waters Corporation) as a surrogate standard prior to analysis.

The remaining 600 µg of protein were then enriched for phosphopeptides using in-house packed TiO_2_ spin columns as previously described [9].

### LC/MS/MS Data collection

The sample cohort was randomized prior to LC/MS/MS analysis. Peptide digests obtained from each of the samples were analyzed in a label-free quantitative fashion using a nanoAcquity UPLC system coupled to a Synapt HDMS mass spectrometer (Waters Corp, Milford, MA) for unenriched peptide analyses and an LTQ Orbitrap XL (Thermo Fisher Scientific, Waltham, MA) for phosphopeptide analyses.

### LC-MS Data Processing

Robust peak detection and label-free alignment of individual peptides across all sample injections was performed using the commercial package Rosetta Elucidator v3.3 (Rosetta Biosoftware, Inc., Seattle, WA) with PeakTeller algorithm [10]. Details are described in supporting methods. The raw data in the form of a flat file (excel) Excel File S1 is provided via this link: https://discovery.genome.duke.edu/express/resources/3772/Schechter_PLOSone_Supplemental_Data_June2014.xlsx.

### Statistical Analysis

Basic statistical analysis was performed on both the unenriched (protein-level) and phosphopeptide (peptide-level) datasets in order to obtain candidate (phospho) proteins that were differentially expressed. Fold-changes were calculated for each failing group versus non-failing control, as the ratio of the average intensity between the groups; directionality of the ratio was established that positive fold-changes mean up-regulated in failing versus nonfailing control, and negative fold-changes mean down-regulated in failing versus nonfailing. P-values were calculated using an error-weighted ANOVA with Benjamini-Hochberg FDR correction for multiple hypotheses testing (Rosetta Elucidator v3.3). The input for this test was the protein-level data for unenriched analysis (intensity for all peptides summed per sample), or the peptide-level data for phosphopeptides, and the raw intensities were scaled to a normal distribution using the Error Model in Elucidator software prior to ANOVA. Fold-changes and p-values are shown for all proteins (Table S1) and phosphopeptides (Table S2). Statistical cutoffs for fold-change were established for proteins based on a power calculation using the average biological variation within each group. Using the protein average %CV (23%) and 4 reps per group at a 95% confidence, minimum cutoffs were set to 2-fold (98% powering) for proteins. ANOVA p-value of 0.05 or less was required.

### Western Blot Analysis

Cryopreserved heart tissue was mechanically disrupted by mortar and pestle in liquid nitrogen and suspended in lysis buffer (1% IGEPAL CA-630, 0.5% Deoxycholate, 2% SDS, 5 mM EDTA in 1X PBS) with protease and phosphatase inhibitor cocktail tablets (Roche Diagnostics, Indianapolis, IN). Samples were then pulse homogenized on ice via mechanical homogenization.

Cardiac tissue homogenates were subjected to Bicinchoninic acid (BCA) assay (Pierce Biotechnology/Thermo Fisher Scientific, Rockford, IL) for protein quantification. Western Blot for protein immunodetection was performed as described previously [11].

### Metabolomics

Organic acids were quantified using methods described previously [12] employing Trace Ultra GC coupled to ISQ MS operating under Xcalibur 2.2 (Thermo Fisher Scientific, Austin, TX).

### Bioinformatics Analysis of Protein Data Sets

Ingenuity Pathway Analysis (IPA, Winter 2012 Release) (Ingenuity Systems, Redwood City, CA) was used to classify the proteins according to primary function as well as for pathway analysis. KinasePhose 2.0 (http://kinasephos2.mbc.nctu.edu.tw/index.html) [13] was used to identify protein-kinase specific phosphorylation sites among the differentially phosphorylated proteins with a specificity threshold of at least 80%.

### Supporting Materials and Methods

Detailed sample preparation, LC-MS processing, and western blot methodology is described in Methods S1.

## Results

### Patient Samples

Left ventricular tissue from 12 male patients matched for age and race was used in this study, with 4 patients in each of the following groups: ischemic failing (IF); non-ischemic failing (NIF); or non-failing (NF) hearts not used for transplantation. Groups were closely matched for cardiac function and treatment history (Table 1). All patients with HF exhibited significantly lower ejection fractions compared to NF controls, which had normal left ventricular function. All HF patients received intravenous inotropic agents and intra-aortic balloon pump support, whereas only patients from the IF subset received prior coronary artery bypass surgery. In the NIF group, two patients were diagnosed with non-ischemic cardiomyopathy of unknown etiology, one patient had a viral cardiomyopathy, and the fourth patient developed HF secondary to valvular disease.

**Table 1:** Demographic and clinical information.

Group	Sample Size (n)	Age (y±m)	Sex (% Male)	Race (% Caucasian)	% Prior Bypass Surgery	Ejection Fraction (%)	% Inotropic Agent	% Intra-aortic Balloon Pump
Non Failing (NF)	4	57.0±3.4	100	50	0	>55%	0	0
Ischemic Failing (IF)	4	58.0±7.0	100	75	100	<15%	100	100
Non Ischemic Failing (NIF)	4	51.5±7.6	100	50	0	<15%	100	100

### Quantitative Analysis of Proteins in Human Left Ventricles

**Figure 1:**
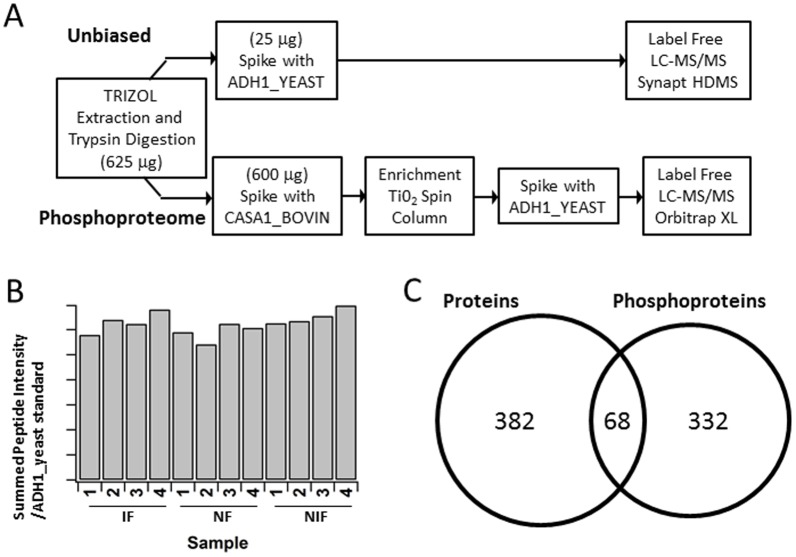
Experimental Approach. A: Illustration of the sample preparation and MS work flow. B: Reproducibility/Internal Control. Relative levels of the ADH1 protein spiked into each heart lysate as determined by chromatographic peak intensity. C: Venn Diagram demonstrating the protein and phosphoprotein yield and overlap.

Following the workflow in Figure 1A, all heart tissue samples were subjected to quantitative analyses of the phospho-enriched and unenriched proteome. The reproducibility of the unenriched analytical approach was validated with a spike-in of the internal standard yeast ADH1 digest at a known concentration. Consistent ADH1 abundance (7% coefficient of variation (CV)) across all 12 samples was observed (Figure 1B).

Peptide/protein identification and quantification by Rosetta Elucidator with Mascot and IdentityE search algorithms yielded expression data for a total of 850 proteins: 4,436 peptide annotations representing 450 proteins in the unenriched samples, for which relative quantitation between samples was performed (Figure 1C; Table S1 for the complete list), and site-specific quantitation for 823 phosphopeptides corresponding to 400 phosphorylated proteins in the phospho-enriched samples were determined (Figure 1C; Table S2 for the complete list). Between the observed unenriched proteome and the phosphoproteome, 68 proteins overlapped (Figure 1C). These 68 proteins and their respective fold changes and p-values are listed in Table S3.

### Differential Expression Analysis

**Figure 2:**
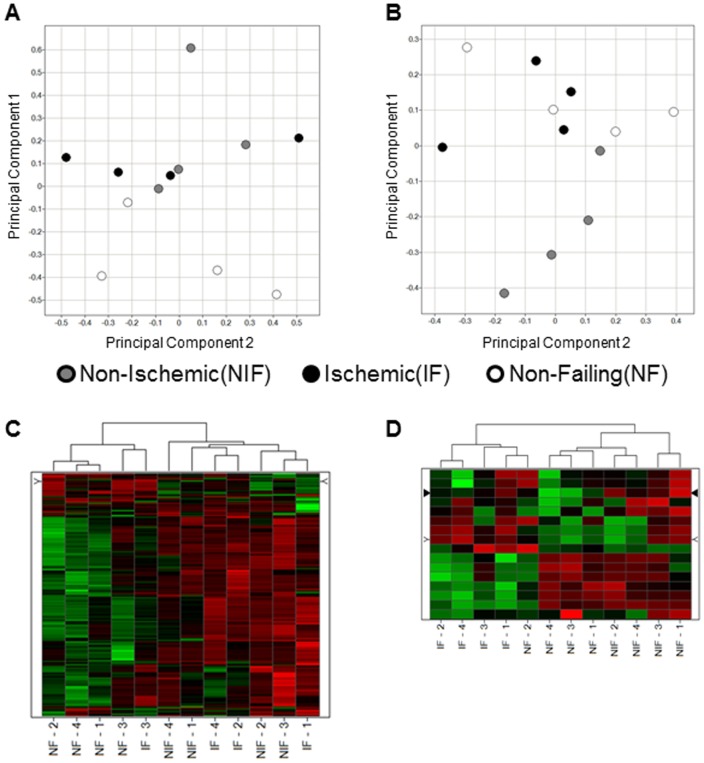
Principal Components Analysis and Hierarchical Clustering. The expression data for all peptides from the unenriched (A) and phosphopeptide enriched (B) samples were used to perform Principal Components Analysis (PCA) after z-score transformation of the peptide intensities. The top two principal components are plotted in each figure, showing no extreme outlier samples among the twelve individual patients tested, either among the unenriched samples or phosphopeptide-enriched. The statistically-significant differentially expressed peptides for each experiment were used to calculate 2D hierarchical clusters in order to view sample-to-sample relationships within these differentially expressed signals, at the unenriched proteome level from Tables 2 and 3 (C) and from the phosphoproteome in Table 4 (D).

Principal components analysis (PCA) was performed in order to observe any high-level differences between sample groups and to screen for outlier samples using the unenriched and phospho-enriched proteome expression data. Expression data was z-score transformed across all samples at the protein-level for unenriched samples (Figure 2A) or at the peptide level for phospho-enriched samples (Figure 2B), and Rosetta Elucidator v3.3 was used to calculate principal components. The two most prominent components (PC1 versus PC2) are plotted in Figures 2A and 2B, and no significant outliers are observed for either unenriched or phosphoproteomes. Interestingly, in unenriched proteomes (Figure 2A), the four NF samples separate from the failing samples along PC1; however, in the phosphoproteome, at least three of the four NIF appear unique along PC1 compared to the IF or NF groups (Figure 2B). This global observation of group classification suggests that the unenriched proteome may best separate failing from NF hearts, whereas phosphorylation status may play a larger role in distinguishing IF versus NIF.

Using the abundance levels of significantly different proteins and phosphopeptides, an unbiased 2D hierarchical clustering analysis was performed at the protein-level (Figure 2C) or phosphopeptide level (Figure 2D) in order to observe the overall expression pattern of these molecules in each individual sample and to determine how these candidates segregate the failing and NF hearts. Figure 2C shows clear differentiation between failing and NF hearts, but none between the two failing etiologies was observed. In contrast, the phosphopeptide analyses (Figure 2D) show a more robust differentiation between all three groups.

Comparison of the unenriched NIF and IF proteomes revealed no differential protein expression, except for carbonic anhydrase 3. The data from the unenriched proteome was filtered to exclude blood proteins and antibodies from consideration. 18 proteins represented by at least two high-confidence peptides and a significant (ANOVA, p< 0.05) fold change of at least two were identified between unenriched failing and NF proteomes (Table 2 and 3). Three proteins in the IF (Table 2) and 15 proteins in NIF (Table 3) differed in abundance from the NF. Moreover, four proteins were decreased in abundance, while the remaining proteins were increased in abundance in the failing hearts (Table 2 and 3). Among the proteins whose abundance was significantly changed in the failing human myocardial tissue were secreted glycoproteins, ceruloplasmin, carbonic anhydrase 1 and 3, serum amyloid A, and extracellular matrix proteins. Peptides from the fibulin family of extracellular matrix proteins (fibulin 1, fibulin2, and latent transforming growth factor beta binding protein 2 (fibulin 3)) were consistently up-regulated across the NIF samples.

**Table 2:** Proteins with statistical significant differential expression between IF and NF heart tissue.

Entry Name	Protein Name	Peptide Count	Fold Change	p-value (ANOVA)	Protein Function
**CAH1_HUMAN**	**Carbonic anhydrase 1**	2	7.24	1.6×10^−3^	Acid-Base balance
**A2MG_HUMAN**	**Alpha-2-macroglobulin**	33	2.31	1.0×10^−4^	Protease Inhibitor
**SAA_HUMAN**	**Serum amyloid A protein**	2	−7.32	1.0×10^−4^	Acute Phase Protein

Data was filtered to eliminate blood proteins and antibodies and to show only proteins with Protein Prophet probability >0.8, FDR-corrected p-value <0.05, absolute fold-change >2. Proteins in bold were validated by western blot analysis.

**Table 3:** Proteins with statistically significant differential expression between NIF and NF heart tissue.

Entry Name	Protein Name	Peptide Count	Fold Change	*p*-value (ANOVA)	Protein Function
CAH3_HUMAN	Carbonic anhydrase 3	2	7.02	1.2×10^−02^	Acid-Base balance
LTBP2_HUMAN	Latent-transforming growth factor â-binding protein 2	6	4.12	9.8×10^−03^	Elastic Fiber Structure
**CAH1_HUMAN**	**Carbonic anhydrase 1**	2	4.06	3.1×10^−04^	Acid-Base balance
ASPN_HUMAN	Asporin	9	3.03	1.5×10^−02^	Cartilage Homeostasis
CO6A3_HUMAN	Collagen alpha-3(VI) chain	6	2.93	5.6×10^−03^	ECM Fibrillar Protein
**FBLN2_HUMAN**	**Fibulin-2**	4	2.52	7.1×10^−03^	ECM Remodeling Protein
MFAP4_HUMAN	Microfibril-associated glycoprotein 4	5	2.50	1.4×10^−02^	Elastic Fiber Formation
FIBG_HUMAN	Fibrinogen gamma chain	13	2.29	1.4×10^02^	Primary Platelet Receptor Binding Site
FBLN3_HUMAN	EGF-containing fibulin-like extracellular matrix protein 1	2	2.25	1.7×10^−02^	Elastic Fiber Formation
**FBLN1_HUMAN**	**Fibulin-1**	2	2.16	4.7×10^−02^	ECM Organization
**CERU_HUMAN**	**Ceruloplasmin**	15	2.09	4.0×10^−03^	Copper Homeostasis
DERM_HUMAN	Dermatopontin	5	2.08	2.8×10^−02^	Fibroblast Cell Adhesion
MYH2_HUMAN	Myosin-2	2	−2.08	4.5×10^−03^	Skeletal Muscle Contraction
ATPD_HUMAN	ATP synthase subunit delta, mitochondrial	2	−2.22	6.2×10^−04^	ATP Synthase Core Subunit
**SAA_HUMAN**	**Serum amyloid A protein**	2	−6.41	1.7×10^−04^	Acute Phase Protein

Data was filtered to eliminate blood proteins and antibodies and to show only proteins with Protein Prophet probability >0.8, FDR-corrected p-value <0.05 and absolute fold-change >2. Proteins in bold were validated by western blot analysis.

Our primary goal was to determine etiology-specific changes in site-specific phosphorylation for the phosphopeptides. A phosphopeptide was considered of interest if it fulfilled the following criteria: 1) a ≥2-fold alteration in phosphorylation state (ANOVA p-value≤0.05) between NIF and IF; 2) significantly different phosphorylation levels between at least one etiology of failing hearts and NF hearts; and 3) phosphorylation level differences observed were unlikely to be due to protein level differences. The last criteria was fulfilled by using either the unenriched protein levels (if available; Table S3 for the 68 overlapping proteins) or the comparative levels of other phosphopeptides from the protein of interest (Table S4) and demonstrating that the levels of these other phosphopeptides are not significantly different between the three heart tissue groups nor do they trend in the same direction as the phosphopeptide under consideration.

From this analysis, 26 proteins were identified to contain at least one differently phosphorylated site between NIF and IF tissue that met our criteria (Table 4). Some of these proteins, such as lyric and leiomodin 1, were differentially phosphorylated in the two types of heart failure. Other phosphoproteins demonstrated either a significant hyper-phosphorylation or dephosphorylation in one or more amino acid sites, while phosphorylation levels at the corresponding amino acid sites were unchanged in the other etiology compared to NF tissue.

**Table 4:** Phosphopeptides with statistically significant differences between IF and NIF human heart tissue.

Primary Protein Name	Protein Description	Modified Peptide Sequence	NIF v IF Fold Change	NIF v IF p-value (ANOVA)	IF v NF Fold Change	IF v NF p-value (ANOVA)	NIF v NF Fold Change	NIF v NF p-value (ANOVA)	Function
LYRIC_HUMAN	Protein LYRIC	LSSQI**S**AGEEK	−7.29	<0.0001	3.31	0.0002	−2.21	0.0080	Transcription co-activator; regulator of apoptosis
LMOD1_HUMAN	Leiomodin-1	GSPKPSPQP**S**PKPSPK	9.66	<0.0001	−1.92	0.0001	5.04	0.0080	Poorly characterized (muscle contraction?)
LMOD1_HUMAN	Leiomodin-1	NSL**S**PATQR	9.18	<0.0001	−4.93	0.0030	1.86	0.7820	
LMOD1_HUMAN	Leiomodin-1	G**S**PKP**S**PQPSPKP**S**PK	4.40	0.0400	−6.84	0.0020	−1.56	0.7110	
									
BASI_HUMAN	Basigin precursor	KPEDVLDDDDAGSAPLKS**S**GQHQNDK	−7.27	<0.0001	4.62	0.0005	−1.57	0.6750	Tissue remodeling; cell shape & tensile properties
LARP7_HUMAN	La-related protein 7	KRS**SS**EDAESLAPR	−4.46	0.0100	3.48	0.0070	−1.28	0.8900	RNA processing; tumorgenesis
MLRV_HUMAN	Myosin regulatory light chain 2, ventricular/cardiac muscle isoform	AGGAN**S**NVFSMFEQTQIQEFK	−3.53	0.0060	1.70	0.7800	−2.08	0.0880	Cardiac muscle contraction, morphogenesis
HS90A_HUMAN	Heat shock protein HSP 90-alpha	E**S**EDKPEIEDVGSDEEEEKK	−3.42	0.0002	3.74	0.0000	1.09	0.9040	Molecular chaperone; cardiac muscle cell apoptosis
ACINU_HUMAN	Apoptotic chromatin condensation inducer in the nucleus	KSS**S**ISEEKGD**S**DDEKPR	−2.15	0.0000	2.50	0.0000	1.16	0.8960	Apoptosis
									
POPD1_HUMAN	Blood vessel epicardial substance	N**S**IASSSDSDDGLHQFLR	−10.06	<0.0001	1.30	0.9980	−7.72	<0.0001	Cell adhesion & signaling
POPD1_HUMAN	Blood vessel epicardial substance	GTS**S**M**S**SLHVSSPHQR	−8.02	0.0120	2.03	0.9410	−3.96	0.1710	Cell adhesion & signaling
FHL2_HUMAN	Four and a half LIM domains protein 2	YI**S**FEER	−3.34	0.0130	−1.04	0.9210	−3.49	0.0350	Transcription co-activator; ECM assembly
HSPB1_HUMAN	Heat shock protein beta-1	GP**S**WDPFRDWYPHSR	−3.32	<0.0001	−1.07	0.8020	−3.56	0.0001	Molecular chaperone
KAP0_HUMAN	cAMP-dependent protein kinase type I-alpha regulatory subunit	TD**S**REDEI**S**PPPPNPVVK	−2.81	0.0040	−1.77	0.3500	−4.98	<0.0001	Regulation of cAMP activity; cardiac muscle cell proliferation
MPRI_HUMAN	Cation-independent mannose-6-phosphate receptor	LV**S**FHDD**S**DEDLLHI	−2.64	0.0120	1.03	0.9350	−2.58	0.0360	Insulin-like growth factor 2 and mannose 6-phosphate signaling
SRCH_HUMAN	Sarcoplasmic reticulum histidine-rich calcium-binding protein	GHDGEDDEGEEEEEEEEEEEEA**S**TEYGHQAHR	−2.57	0.0280	−1.36	0.6690	−3.50	0.0040	Calcium homeostasis; regulation of heart contraction
KCRM_HUMAN	Creatine kinase M-type	GTGGVDTAAVG**S**VFDVSNADR	−2.11	0.0370	−1.79	0.5830	−3.77	0.0001	Energy homeostasis; biomarker for myocardial infarction
									
SRBS2_HUMAN	Sorbin and SH3 domain-containing protein 2	SFTSS**S**P**S**SPSR	5.55	<0.0001	−1.69	0.2940	3.28	0.0350	Z-band signaling; cytoskeleton regulation
AKA12_HUMAN	A-kinase anchor protein 12	EGVTPWA**S**FKK	4.43	0.0030	−1.02	0.9800	4.33	0.0080	Cell growth; signal transduction
SRBS2_HUMAN	Sorbin and SH3 domain-containing protein 2	TSPGRVDLPG**S**STTLTK	3.70	<0.0001	1.04	0.9970	3.85	0.0004	Z-band signaling; cytoskeleton regulation
MAP4_HUMAN	Microtubule-associated protein 4	VG**S**LDNVGHLPAGGAVK	3.32	0.0190	1.37	0.5590	4.55	<0.0001	Cell cycle progression
SRBS2_HUMAN	Sorbin and SH3 domain-containing protein 2	**T**SPGRVDLPGSSTTLTK	3.15	0.0010	-1.07	0.8410	2.95	0.0060	Z-band signaling; cytoskeleton regulation
TITIN_HUMAN	Titin	SR**S**TPPSIAAK	3.10	0.0300	1.73	0.5250	5.36	0.0001	Cardiac muscle development & contraction; tissue elasticity
LMO7_HUMAN	LIM domain only protein 7	RGE**S**LDNLDSPR	2.79	0.0340	1.32	0.7820	3.67	0.0040	Cell adhesion
SRBS2_HUMAN	Sorbin and SH3 domain-containing protein 2	DAS**S**PVPPPHVPPPVPPLRPR	2.64	0.0140	2.64	0.2380	6.98	0.0007	Z-band signaling; cytoskeleton regulation
MATR3_HUMAN	Matrin-3	SY**S**PDGKESPSDKK	2.61	0.0060	−1.11	0.0003	2.36	0.0750	Cell growth & proliferation; DNA damage response
SRBS2_HUMAN	Sorbin and SH3 domain-containing protein 2	DA**S**SPVPPPHVPPPVPPLRPR	2.26	0.0040	1.52	0.3420	3.44	<0.0001	Z-band signaling; cytoskeleton regulation
									
ODPA_HUMAN	Pyruvate dehydrogenase E1 component subunit alpha, somatic form, mitochondrial	YGMG**T**SVER	123.00	<0.0001	−61.57	<0.0001	2.00	0.7030	Glycolysis; cellular metabolism
ODPA_HUMAN	Pyruvate dehydrogenase E1 component subunit alpha, somatic form, mitochondrial	YHGH**S**MSDPGV**S**YR	32.46	<0.0001	−32.49	<0.0001	−1.00	0.9390	
ODPA_HUMAN	Pyruvate dehydrogenase E1 component subunit alpha, somatic form, mitochondrial	YHGH**S**MSDPGVS**Y**R	25.87	<0.0001	−27.30	<0.0001	−1.06	0.9110	
NEXN_HUMAN	Nexilin	EMLA**S**DDEEDVSSKVEK	13.46	0.0005	−16.05	0.0003	−1.19	0.6860	Z-disc protein; cytoskeleton organization
PTRFL_HUMAN	PTRF/SDPR family protein	EIP**T**PEPLK	9.33	<0.0001	−2.60	0.0120	3.59	0.1390	Cardiac myofibril assembly
LMOD1_HUMAN	Leiomodin-1	NSL**S**PATQR	9.18	<0.0001	−4.93	0.0030	1.86	0.7820	Poorly characterized (? Muscle contraction)
HSPB8_HUMAN	Heat shock protein beta-8	DPFRD**S**PLSSR	5.28	<0.0001	−5.87	<0.0001	−1.11	0.8580	Molecular chaperone
TITIN_HUMAN	Titin	RVK**S**PEPSHPK	3.76	<0.0001	−2.18	0.0001	1.72	0.6440	Cardiac muscle development & contraction; tissue elasticity
TITIN_HUMAN	Titin	AV**S**PTETKPTPTEK	2.56	0.0370	−2.64	0.0450	−1.03	0.9640	
FA54B_HUMAN	Protein FAM54B	NA**S**VPNLR	2.51	0.0010	−4.67	<0.0001	−1.86	0.0320	Mitochondrial fission regulator

Phosphorylation sites are underlined.

### Validation Western Blot Analysis

**Figure 3:**
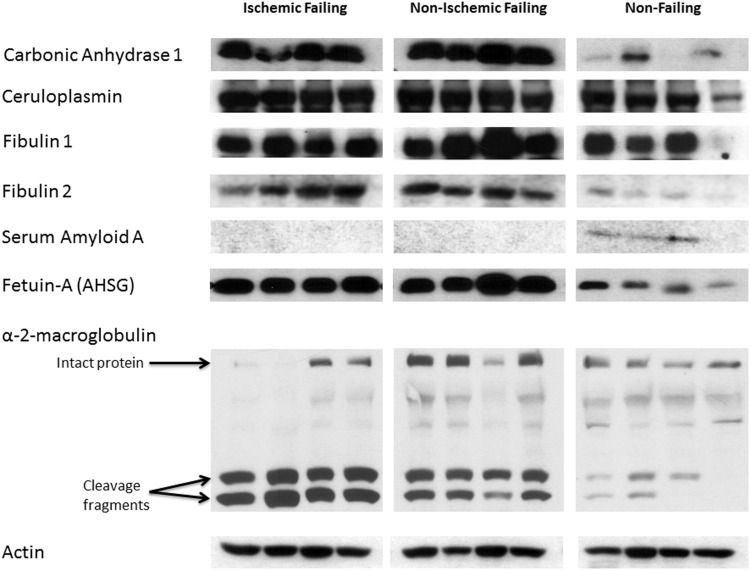
Western blot validations of selected statistically significant proteins identified by proteomics analysis. Protein extracts from IF, NIF, or NF samples (40 µg) were subjected to polyacrylamide gel electrophoresis. Replica nitrocellulose blots were incubated with anti- carbonic anhydrase, -ceruloplasmin, -fibulin 1, -fibulin 2, -serum amyloid A, -fetuin A, -alpha 2 macroglobulin, -PDH Serine 300, or -sarcomeric actin.

**Figure 4:**
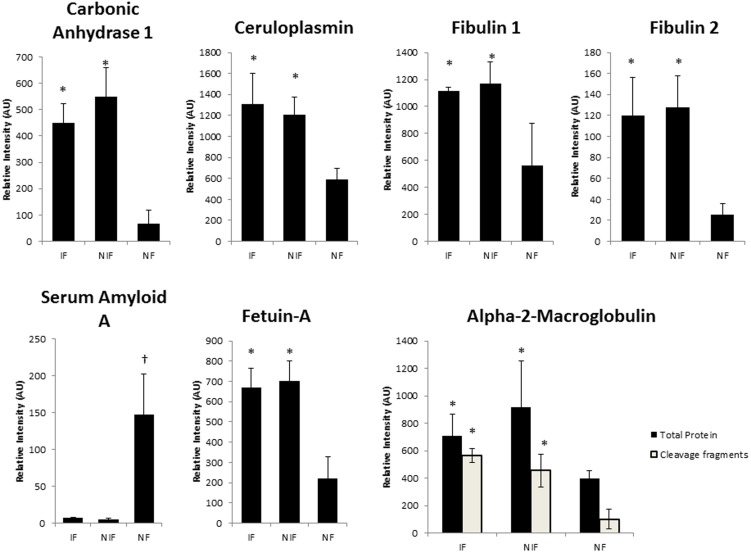
Quantitation of carbonic anhydrase 1, ceruloplasmin, fibulin 1, fibulin 2, serum amyloid A, fetuin A, and total and cleavage products of alpha 2 macroglobulin as determined by densitometry analysis and expressed as relative intensity (arbitrary units (AU)) normalized to actin levels. *: significantly elevated relative to NF samples, †: significantly elevated relative to both NIF and IF.

The unenriched proteomic mass spec data were validated by Western Blot analysis of independently prepared tissue homogenates from the same 12 patients (Figure 3). Western blot quantitative data is shown in Figure 4. These Western blots confirmed the altered changes in IF and NIF compared to NF that were revealed by the mass spec data: an increase in carbonic anhydrase, ceruloplasmin, Fibulin1, Fibulin2 and Alpha 2-HS glycoprotein (FETUA); a decrease in serum amyloid A; and an increase in α2-Macroglobulin (α2M). Interestingly, a cleaved version of α2M was the prevalent form of α2M in both NIF and IF hearts, whereas the full-length α2M was the prevalent species in NF heart tissue (Figures 3 and 4).

### Metabolomic analysis

**Figure 5:**
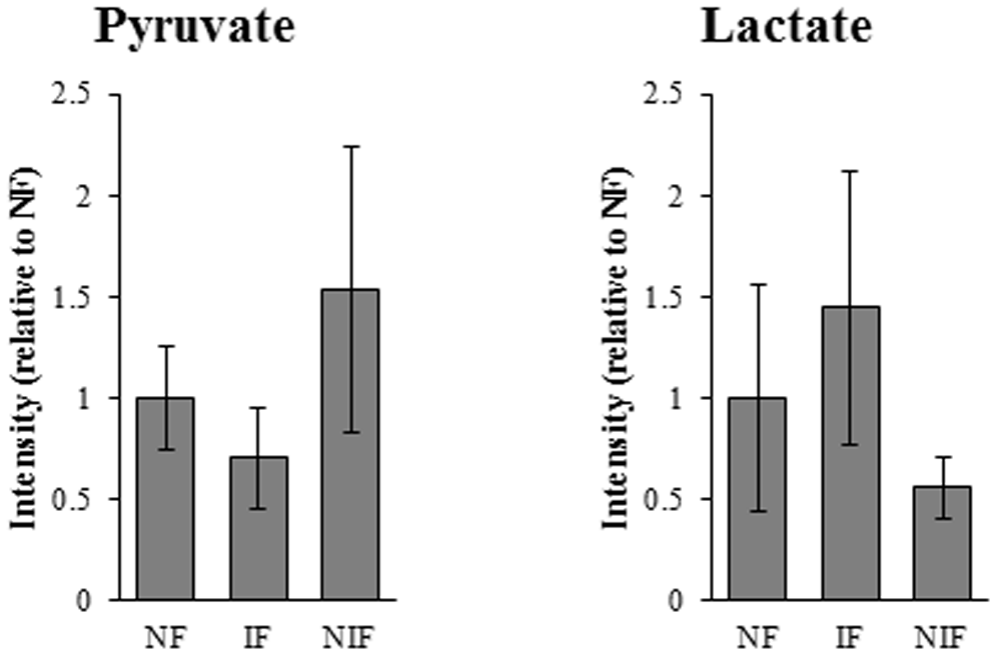
Evaluation of lactate and pyruvate levels in NF, non-failing; IF, ischemic failing; NIF, non-ischemic failing human myocardial tissues. Data is reported as fluorescent intensity relative to NF tissue.

Pyruvate dehydrogenase E1 component subunit alpha (ODPA or PDH) catalyzes the overall conversion of pyruvate to acetyl-CoA and CO_2_, and provides the primary link between glycolysis and the tricarboxylic acid cycle. A significant decrease in phosphorylation of PDH at Ser 300 was observed in IF heart compared to both NIF and NF samples (Table 4) suggestive that PDH is more active in IF hearts than NIF or NF hearts. Mass spectrometry methods were used to examine pyruvate content in the same 12 human myocardial samples (Figure 5). The IF tissue had the lowest levels of pyruvate, supportive of our hypothesis that of the three groups of patients, PDH is most active in IF. Another enzyme that influences pyruvate levels in the myocardium is lactate dehydrogenase (LDH). LDH catalyzes the conversion of pyruvate to lactate and back. Lactate levels were the highest in IF (Figure 5) suggestive that in IF tissues both PDH and LDH are more active than in NIF.

### Bioinformatics Analysis

**Figure 6:**
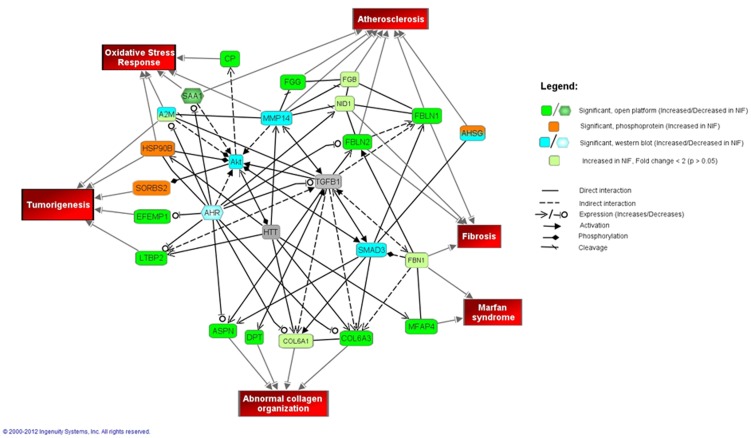
Interaction network of non-ischemic failing hearts. Relevant interactions of the differentially expressed proteins and their relationships with selected pathologies are depicted.

**Figure 7:**
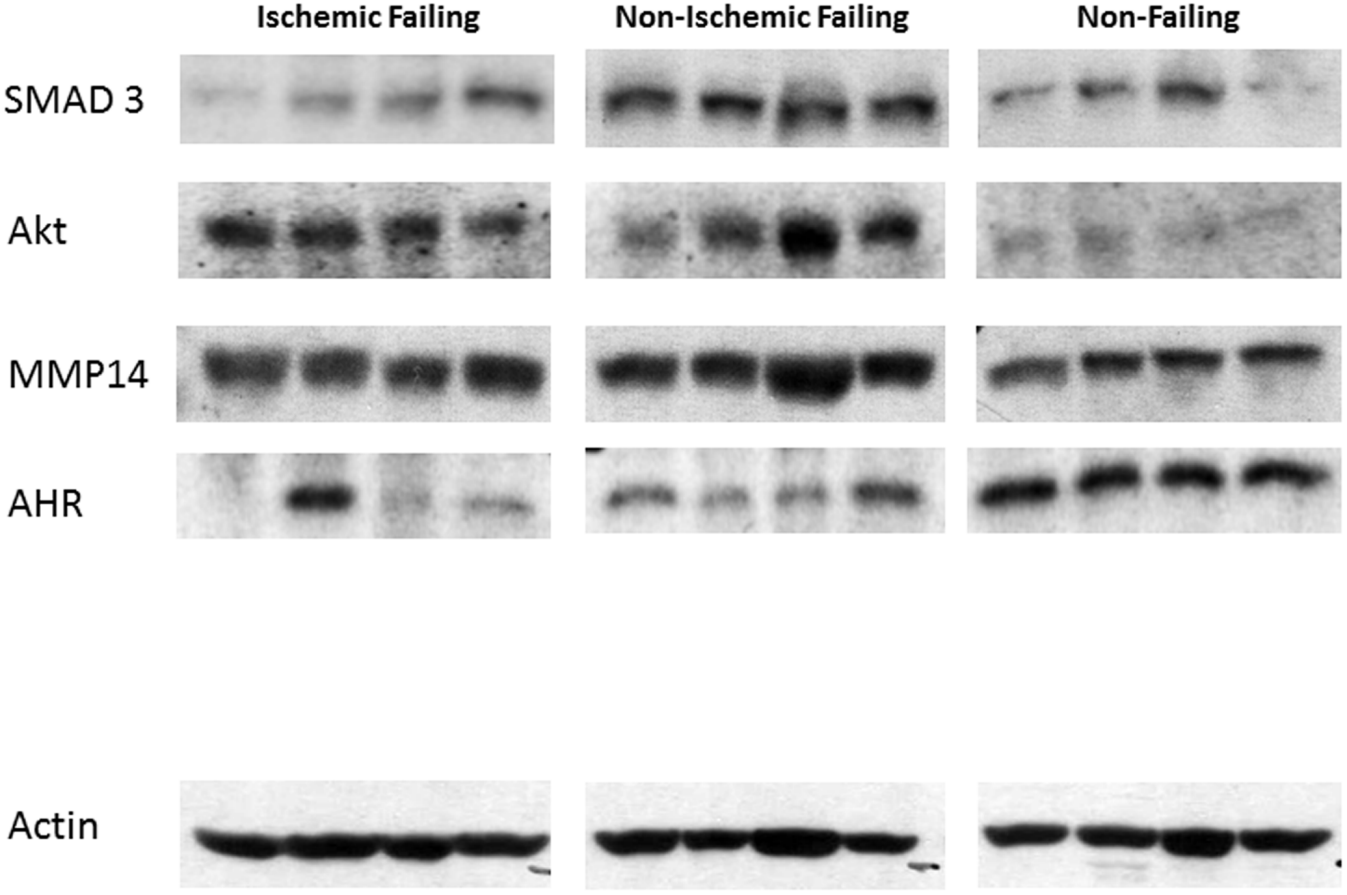
Western blot analysis of central proteins identified from pathway analysis. Protein extracts from IF, NIF, or NF samples (40 µg) were subjected to polyacrylamide gel electrophoresis. Replica nitrocellulose blots were incubated with anti- SMAD3, -AKT, -MMP14, -AHR or -sarcomeric actin.

**Figure 8:**
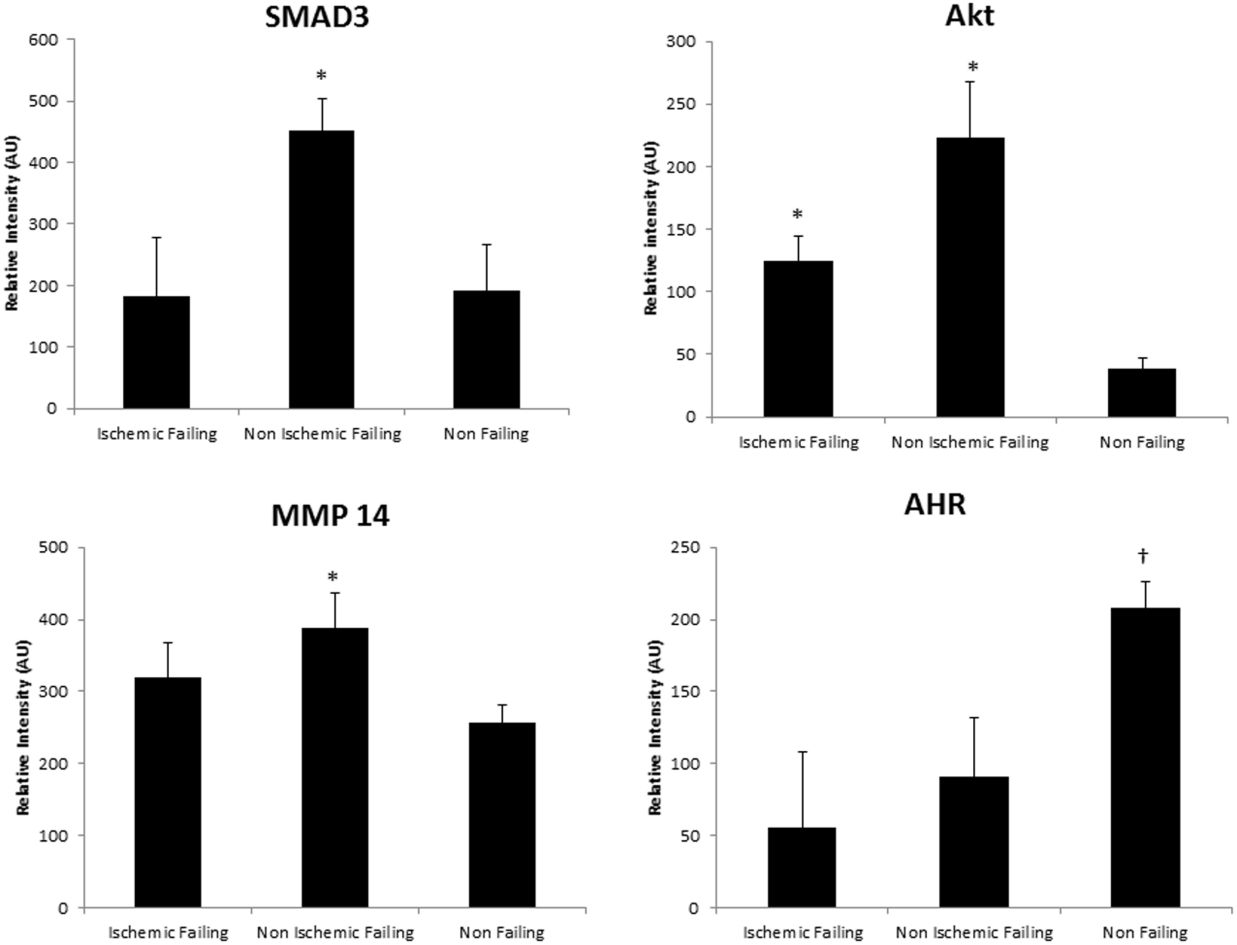
Quantitation of SMAD3, AKT, MMP14, and AHR as determined by densitometry analysis and expressed as relative intensity (arbitrary units (AU)) normalized to actin levels. *: significantly elevated relative to non-failing, †: significantly elevated relative to both NIF and IF.

Focusing on differential protein or phosphoprotein levels between NIF and NF samples, Ingenuity Pathway Analysis (IPA) was used to construct an interaction network of proteins (Figure 6). Many of the proteins that were significantly different (>2-fold change, p<0.05) were glycoproteins, proteoglycans, and structural proteins, all components of the extracellular matrix (ECM). More ECM proteins were discovered when the inclusion criteria were expanded to significantly different proteins between NIF and NF with <2-fold change (min fold change 1.46) using the IPA analysis (Figure 6, light green). Pathway analysis also suggested that Akt, SMAD3, MMP14, and aryl hydrocarbon receptor (AHR), all of which have been implicated in cardiac remodeling [14]–[17], may be central regulators controlling the expression levels of many of the proteins included in the IPA analysis. IPA analysis validation was performed by Western blot analysis, which established that several of these more central proteins (AKT, SMAD3, and AHR) were also differentially expressed in NIF (Figures 7 and 8).

**Figure 9:**
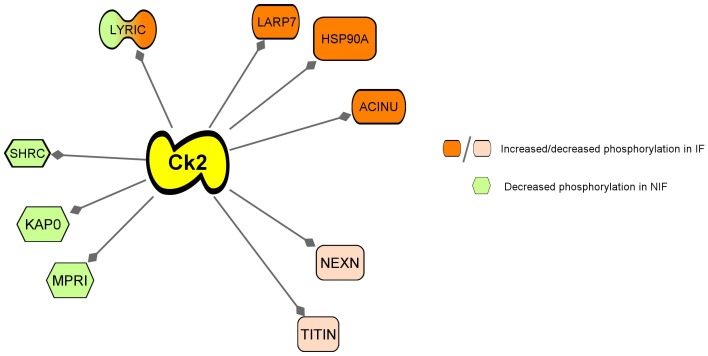
Casein kinase phosphorylation targets. Nine of the differentially phosphorylated proteins shown in Table 4 are possible targets of casein kinase.

Further bioinformatics analysis of the phosphorylation data demonstrated that nine of the differentially phosphorylated proteins are possible targets of casein kinase 2 (Ck2, Figure 9). The four differentially phosphorylated proteins in NIF (LYRIC, SHRC, FHL2, KAP0 and MPRI) were all dephosphorylated in this disease state, while four of the six differentially phosphorylated proteins in IF (LYRIC, LARP7, HSP90A, ACINU) were increasingly phosphorylated.

## Discussion

Heart failure remains a progressive disease with limited treatment options. Current standard of care does not differentiate between ischemic and non-ischemic etiologies, treating them similarly. However, as this study suggests, there are important protein phosphorylation differences between the two HF etiologies. Given the variety of cellular processes these proteins are involved in, these differences may be important in the design of therapies for heart failure.

Our analysis revealed the following general differences between the two main types of heart failure: 1) Protein abundances were similar between both types of heart failure compared to NF controls, with one exception; and 2) Site-specific phosphorylation differences on many proteins clearly distinguished non-ischemic and ischemic HF.

### Protein abundance differences between non-ischemic and ischemic heart tissue

The only protein that displayed statistically significant differential expression between NIF and IF was carbonic anhydrase 3 (CAH3). CAH3 levels were 2.83-fold higher in the NIF than in the IF samples. Why the levels of CAH3 increase in HF, especially in NIF, is unclear. In general, many tissues contain carbonic anhydrases, which interconvert carbon dioxide and bicarbonate to maintain acid-base balance and help transport carbon dioxide out of tissues, especially metabolically active tissues like heart and brain. Therefore, the increase of CAH3 could be a sign that these hearts are increasingly metabolically active in an attempt to compensate for the decrease in function.

### Protein abundance differences between failing and non-failing human tissue

#### Extracellular matrix remodeling in the context of NIF

The involvement of ECM remodeling in NIF was our most robust finding from evaluation of protein level differences between failing and non-failing tissues (Figure 6). This finding is not only consistent with other studies that have used proteomic analysis to investigate protein expression variation in cardiac disease, but it complements their findings as well [18], [19]. The Barallobre-Barreiro *et al* study, which focused on the ECM remodeling in ischemia/reperfusion injury using a pig model, found an interaction network centered on transforming growth factor-β1 (TGFB1), a known profibrotic molecule[19]. TGFB1 was also heavily involved in our network (Figure 6). Additionally, our network demonstrated what the Barallobre-Barreiro *et al* study suggested, that fibulin 1, 2, and 3 (components of elastic fibers) were significantly up regulated in NIF and trended toward up regulation in IF. These revelations were confirmed by western blot analysis of fibulin 1 and 2 (Figures 3 and 4). Up regulation of fibulin-1 is associated with cardiac disease, [20]–[22] while up regulation of fibulin-2 and fibulin-3, although not implicated in cardiac disease, is associated with a variety of other fibrotic disorders [23]. Down regulating fibulins may be a therapeutic target in future treatments of heart failure.

#### Oxidative stress, cellular damage, and inflammation

NIF and IF samples had similarly modulated proteins involved in the oxidative stress and inflammatory pathways, including ceruloplasmin, heat shock protein 90, serum amyloid A, and α2-macroglobulin (α2M). These proteins had comparative abundances in human heart tissue that were confirmed by Western blot analyses (Figures 3 and 4).

When exposed to activated proteases, α2M exposes a “bait region” within its structure that traps the protease. Following protease binding, α2M promotes clearance and degradation of the bound protease while undergoing limited proteolysis [24]. This limited proteolysis, however, does result in α2M cleavage, and our findings reveal an increase in α2M cleavage products. Therefore, the increased levels of cleaved α2M in HF tissue could be protective attempts to limit the effects of harmful proteases. In addition to binding proteases, the native form of α2M can bind to damaged proteins in order to limit cellular damage and abnormal protein deposition [25]. However this broader protective function of α2M is lost upon exposure to proteases [25] and this decreased ability of α2M to clear damaging protein deposits could be contributing to HF progression. Whether a cause of or response to cellular damage, the cleaved form of α-2M could be a useful biomarker for the progression of HF.

### NIF and IF etiologies have different phosphorylation signatures

While the unenriched data set demonstrates the common pathways underlying both types of heart failure, the phosphorylation analysis indicates differences between the two disease etiologies. These differences can be grouped as: 1) etiology-specific differences in phosphorylation at a particular site; and 2) significantly different single- or multiple-site phosphorylation in only one type of HF (compared to NF control). Another notable finding was that over one-third of the differentially phosphorylated protein sequences (9/26) were potential targets of casein kinase 2 (Ck2) (Figure 9). Common functional themes of the significant phosphoproteins include cell growth/death, cardiac muscle development and/or function, and stress response, suggesting etiology-specific regulation of these functions contribute to the development or progression of heart failure.

#### Etiology-specific differences in phosphorylation

Lyric, also known as metadherin, is a transcription co-factor. Increased Lyric content and phosphorylation play a major role in cancer cell survival [26], [27]. Our finding is the first report indicating an association between phosphorylation of Lyric and heart failure. The NIF heart samples in this study displayed increased phosphorylation at lyric amino acid 298, whereas IF samples displayed a decrease in lyric phosphorylation at the same site (Table 4). Although no studies have established the role of phosphorylation at site 298 in the regulation of Lyric activity, other regions on the Lyric protein are involved in protein/protein interactions and appear to be regulated by phosphorylation events. Assuming that this phosphorylation/dephosphorylation of Lyric at 298 also functions as a toggle switch for protein binding, the increased phosphorylation in IF could be an attempt to recruit a partner transcription factor to activate cell growth in the viable myocardium to compensate for the injury already suffered. Conversely, dephosphorylation of the pro-growth Lyric protein could be contributing to progression of NIF heart failure by activating pro-cell death pathways or suppressing pro-cell growth pathways.

Leiomodin-1 is primarily expressed in smooth muscle, with some expression in the heart, and is speculated to play a role in cardiac hypertrophy and muscle contraction [28]. Phosphorylation of leiomodin-1 was suggested to be a mediator of the actin cytoskeleton formation in human trabecular meshwork cells [29]. Multiple amino acid sites on leiomodin-1 exhibited greater phosphorylation in the NIF samples than the IF samples, with differences ranging from 4 to 9 fold greater, depending on the site (Table 4). The presence of leiomodin-1 in the smooth muscle and the cytoskeleton links well to a pathological role in heart failure. Generation of mutants in leiomodin-1 unable to be phosphorylated at the amino acids revealed herein will be critical in determining the involvement of this relatively uncharacterized protein in the development of these two distinct types of heart failure.

#### Significantly different phosphorylation only seen in one HF etiology

Phosphorylation of basigin at serine 368 near the C-terminus was increased in IF (compared to NIF and NF). Basigin is a transmembrane glycoprotein that induces MMP activity and subsequent ECM breakdown that is associated with adverse tissue remodeling seen in many pathological conditions such as HF[30]. Basigin is also connected to energy metabolism by directly mediating monocarboxylate transporter (MCT) localization and activity in the sarcolemma. Dissociation of basigin from MCT1, the primary isoform in heart, reduces cellular lactate influx limiting lactate oxidation inside the cardiomyocyte [31]. Considering basigin-MCT1 interactions, the phosphorylation state of basigin could affect lactate influx into the cardiomyocyte and thus influence cardiac metabolism. In fact, the C-terminus of basigin is necessary for dimerization and association with MCT1 [32], and a serine-to-alanine mutation at serine 193 disrupted basigin dimerization and MMP2 induction [33]. This data suggests that phosphorylation of basigin may alter its activity and function, and could contribute to the increase in lactate pools seen in the IF. The role of differential phosphorylation states of basigin in heart failure deserves further attention.

POPD1, a member of the Popeye domain containing family, is a membrane protein abundantly expressed in the heart. POPD1 has been shown to play an important role in cardiac pacemaker cells, cellular adhesion, and cellular signaling, especially in times of stress [34], [35]. Some literature suggest that POPD1 content is decreased in human heart failure [36]. While the overall abundance of POPD1 was not changed between the three groups in our data, POPD1 phosphorylation at 2 sites was diminished in NIF heart relative to NF heart, while phosphorylation at these sites was minimally changed in IF (Table 4). POPD1 phosphorylation has not been examined, but this protein is important in the RhoA signaling pathway, a key regulator of myosin light chain phosphorylation [37]. Myosin light chain phosphorylation is decreased when POPD1 is overexpressed, and a recent study demonstrated that the transition from compensated to decompensated heart failure is centrally mediated by a reduction in myosin light chain phosphorylation [38]. Interestingly, in our study, slow cardiac myosin regulatory light chain 2 (MLRV) was significantly less phosphorylated at serine 15 in NIF compared to IF heart (Table 4). Together, these findings suggest etiology-specific phosphorylation events in the RhoA/POPD1/MLRV pathway may play a role in the development of non-ischemic heart failure.

Multiple sites on SORBS2 exhibited increased phosphorylation in NIF samples compared to the other two groups (Table 4). SORBS2 is an adaptor protein that mediates interactions between structural proteins, membrane proteins, other signaling molecules, and actin filaments in cardiomyocytes [39]. From a mass spectrometry based proteomics approach, SORBS2 has been recently identified as a protein released from cardiac tissue immediately following acute myocardial infarction [40]. SORBS2 also promotes the ubiquitination and degradation of c-Abl, an important regulator of the actin cytoskeleton and apoptosis, [41] and alterations in SORBS2 activity by differential phosphorylation could activate the apoptotic cascade contributing to the development of non-ischemic heart failure. SORBS2 is expressed exclusively within the myofibril Z-bands, which link the sarcomeric contractile units together, and regulates the signaling cascade needed for proper force production and transmission in these contractile units [39], [42]. Another sarcomeric protein that plays a key role in force transmission at the Z-line, titin, was differentially phosphorylated in NIF (Table 4). The phosphorylation of these two structural proteins may contribute to the disruption of the tightly regulated contractile apparatus leading to the cardiac contractile deficiencies in NIF.

Phosphorylation/dephosphorylation of PDH/ODPA is complex. The activity of PDH is regulated via phosphorylation of three serine resides (232, 293, and 300) [43]. *In vitro*, phosphorylation at a single site is sufficient to inactivate PDH, [43] and dephosphorylation by pyruvate dehydrogenase phosphatases activate PDH [44]. A decrease in phosphorylation of PDH at Ser 300 is observed in IF, while phosphorylation at Ser 293 is unchanged or only modestly changed. In non-failing, well-perfused heart, fatty acids provide 60–90% of the energy for ATP production, with the remaining 10–40% derived from carbohydrate (glucose and lactate) oxidation [45]. In the failing heart, the preferential fuel source switches to glucose from fatty acids. The marked decrease in PDH phosphorylation in IF supports the current dogma regarding the switch towards glucose, at least in IF, and suggests a mechanism by which this is occurring, namely reduction of PDH inhibition. Interestingly, PDH phosphorylation was not significantly changed in NIF hearts, suggesting that this shift in energy substrate is etiology specific. The trend in lower pyruvate levels in the IF tissue fits with our hypothesis that PDH activity is increased in IF. Despite the differences in pyruvate and PDH Ser 300 levels between HF etiologies, PDH enzyme activity was not statistically different between groups (data not shown, p = 0.866). In all, the data suggest that PDH activity may differentiate between NIF and IF, but additional studies will be required. Whether the increase in glucose oxidation within the ischemic heart represents a protective response to chronic ischemia or a maladaptive response that further stresses the ischemic heart remains unclear.

### Role of Casein Kinase 2 in the development of heart failure: Etiology specific?

Of the 26 differently phosphorylated proteins, nine had sites that were predicted targets of casein kinase 2 (Ck2, Figure 9). Ck2 is a ubiquitous serine/threonine protein kinases thought to have over 300 protein substrates that modulate a variety of cellular processes, including cell cycle control, cellular differentiation, and proliferation [46]. Classified as a “messenger-independent kinase,” Ck2 is a constitutively active kinase that is regulated through protein-protein interactions, localization, and extent of phosphorylation and oligomerization [46], [47]. Ck2 activity has been shown to both induce hypertrophy and inhibit apoptosis within cardiomyocytes, primarily through its interaction with apoptosis repressor with caspase recruitment domain (ARC), histone deacetylases, and the tumor suppressor gene p27 [48]–[52]. Many studies have demonstrated that Ck2 activity is critical for ischemic and stress-induced cardiac hypertrophic growth [48]–[54].

The majority of significant Ck2-specific sites in the IF hearts were increasingly phosphorylated, while all of the significant phosphoproteins with Ck2-specific sites in the NIF group were all relatively dephosphorylated. Furthermore, Lyric, a protein showing etiology-specific differences in phosphorylation, is predicted to be a target of Ck2 [13].

The observation that many of these phosphorylation sites may be targets of Ck2 suggest that variation in Ck2 activity may point to a branching point between these two disease states. In ischemic heart failure, the initial injury and cardiomyocyte loss triggers pro-hypertrophic and anti-apoptotic pathways, of which Ck2 is a central player. This sustained cardiac hypertrophy, due in part to increased Ck2 activity, leads to maladaptive cardiac remodeling and eventually heart failure. Conversely, in non-ischemic heart disease, Ck2 activity is decreased, leading to increased apoptotic activity and suppression of pro-growth pathways. As such, modulation of Ck2 activity could be an etiology-specific treatment, although further analysis into the role of Ck2 in the development of ischemic and non-ischemic heart failure is needed.

### Conclusions

This is the first study using a multi-‘omics-driven systems biology approach to obtain a more integrated and comprehensive molecular assessment of the different etiologies of heart failure. Using an unbiased assessment of the unenriched and phosphoenriched proteomes of two HF etiologies, combined with selected metabolomics and Western blot analysis, the previously undocumented molecular fingerprints of the different heart failure etiologies were explored. These molecular differences between heart failure etiologies have enabled us to complement pathways believed to be involved in the development of HF in general. As our human heart repository becomes more robust in terms of increased specimen number and cohort size, the evaluation of additional post translational modifications (PTMs) and the use of multiple reaction monitoring [55] for more sensitive and precise quantitative analyses of these PTMs becomes achievable. Comprehensive understanding of the proteome of this common disease through study of human cardiac tissue from a robust biorepository will lead to better, more focused treatments, and ultimately improved patient outcomes.

## Supporting Information

Excel File S1 **The following information is contained within Excel File S1.** Key sheet-Contains an index of the remainder of the excel file sheets, sample identifiers, patient ID, disease etiology, peptide modifications, and Modloc scoring criteria. ST1-peptide expression data from unbiased proteomics. ST2-protein expression data from unbiased proteomics. ST3-Phosphopeptide expression data from phospho-enriched proteomics. ST4-Differential Unbiased Protein Expression (Fold Changes, ANOVA) for unbiased proteomics. ST5-Differential Phosphopeptide Expression (Fold Changes, ANOVA).
(XLSX)

Methods S1 **Supplemental Materials and Methods.**
(DOCX)

Table S1 **Unenriched protein expression profiles.**
(PDF)

Table S2 **Phosphoenriched peptide expression profiles.**
(PDF)

Table S3 **Expression profile of 68 unenriched overlapping proteins.**
(PDF)

Table S4 **Differential phosphorylation of proteins in NIF and IF heart.**
(PDF)

## References

[1] Roger VL, Go AS, Lloyd-Jones DM, Benjamin EJ, Berry JD, et al (2012) Heart disease and stroke statistics–2012 update: a report from the American Heart Association. Circulation 125: e2–e220. 22179539 10.1161/CIR.0b013e31823ac046 PMC4440543

[2] Lloyd-Jones DM, Larson MG, Leip EP, Beiser A, D'Agostino RB, et al (2002) Lifetime risk for developing congestive heart failure: the Framingham Heart Study. Circulation 106: 3068–3072. 12473553 10.1161/01.cir.0000039105.49749.6f

[3] Levy D, Kenchaiah S, Larson MG, Benjamin EJ, Kupka MJ, et al (2002) Long-term trends in the incidence of and survival with heart failure. N Engl J Med 347: 1397–1402. 12409541 10.1056/NEJMoa020265

[4] Roger VL, Go AS, Lloyd-Jones DM, Benjamin EJ, Berry JD, et al (2012) Executive summary: heart disease and stroke statistics–2012 update: a report from the American Heart Association. Circulation 125: 188–197. 22215894 10.1161/CIR.0b013e3182456d46

[5] Margulies KB, Bednarik DP, Dries DL (2009) Genomics, transcriptional profiling, and heart failure. J Am Coll Cardiol 53: 1752–1759. 19422981 10.1016/j.jacc.2008.12.064 PMC2738978

[6] Sharma P, Cosme J, Gramolini AO (2012) Recent advances in cardiovascular proteomics. J Proteomics 10.1016/j.jprot.2012.10.026 PMC3707843 23153792

[7] Gonzalez A, Lopez B, Beaumont J, Ravassa S, Arias T, et al (2009) Cardiovascular translational medicine (III). Genomics and proteomics in heart failure research. Rev Esp Cardiol 62: 305–313. 19268076 10.1016/s1885-5857(09)71561-0

[8] Klawitter J, Klawitter J, Agardi E, Corby K, Leibfritz D, et al (2013) Association of DJ-1/PTEN/AKT- and ASK1/p38-mediated cell signalling with ischaemic cardiomyopathy. Cardiovasc Res 97: 66–76. 23015639 10.1093/cvr/cvs302

[9] Soderblom EJ, Philipp M, Thompson JW, Caron MG, Moseley MA (2011) Quantitative label-free phosphoproteomics strategy for multifaceted experimental designs. Anal Chem 83: 3758–3764. 21491946 10.1021/ac200213b PMC3093925

[10] Weng L, Dai H, Zhan Y, He Y, Stepaniants SB, et al (2006) Rosetta error model for gene expression analysis. Bioinformatics 22: 1111–1121. 16522673 10.1093/bioinformatics/btl045

[11] Piacentino V 3rd, Milano CA, Bolanos M, Schroder J, Messina E, et al (2012) X-linked inhibitor of apoptosis protein-mediated attenuation of apoptosis, using a novel cardiac-enhanced adeno-associated viral vector. Hum Gene Ther 23: 635–646. 22339372 10.1089/hum.2011.186 PMC3392616

[12] Jensen MV, Joseph JW, Ilkayeva O, Burgess S, Lu D, et al (2006) Compensatory responses to pyruvate carboxylase suppression in islet beta-cells. Preservation of glucose-stimulated insulin secretion. J Biol Chem 281: 22342–22351. 16740637 10.1074/jbc.M604350200

[13] Wong YH, Lee TY, Liang HK, Huang CM, Wang TY, et al (2007) KinasePhos 2.0: a web server for identifying protein kinase-specific phosphorylation sites based on sequences and coupling patterns. Nucleic Acids Res 35: W588–594. 17517770 10.1093/nar/gkm322 PMC1933228

[14] Jourdan-Lesaux C, Zhang J, Lindsey ML (2010) Extracellular matrix roles during cardiac repair. Life Sci 87: 391–400. 20670633 10.1016/j.lfs.2010.07.010 PMC2946433

[15] Vilahur G, Cubedo J, Casani L, Padro T, Sabate-Tenas M, et al (2012) Reperfusion-triggered stress protein response in the myocardium is blocked by post-conditioning. Systems biology pathway analysis highlights the key role of the canonical aryl-hydrocarbon receptor pathway. Eur Heart J 10.1093/eurheartj/ehs211 22851653

[16] Bujak M, Ren G, Kweon HJ, Dobaczewski M, Reddy A, et al (2007) Essential role of Smad3 in infarct healing and in the pathogenesis of cardiac remodeling. Circulation 116: 2127–2138. 17967775 10.1161/CIRCULATIONAHA.107.704197

[17] Chaanine AH, Hajjar RJ (2011) AKT signalling in the failing heart. Eur J Heart Fail 13: 825–829. 21724622 10.1093/eurjhf/hfr080 PMC3143831

[18] Rosello-Lleti E, Alonso J, Cortes R, Almenar L, Martinez-Dolz L, et al (2012) Cardiac protein changes in ischaemic and dilated cardiomyopathy: a proteomic study of human left ventricular tissue. J Cell Mol Med 16: 2471–2486. 22435364 10.1111/j.1582-4934.2012.01565.x PMC3823441

[19] Barallobre-Barreiro J, Didangelos A, Schoendube FA, Drozdov I, Yin X, et al (2012) Proteomics analysis of cardiac extracellular matrix remodeling in a porcine model of ischemia/reperfusion injury. Circulation 125: 789–802. 22261194 10.1161/CIRCULATIONAHA.111.056952

[20] de Vega S, Iwamoto T, Yamada Y (2009) Fibulins: multiple roles in matrix structures and tissue functions. Cell Mol Life Sci 66: 1890–1902. 19189051 10.1007/s00018-009-8632-6 PMC11115505

[21] Argraves WS, Greene LM, Cooley MA, Gallagher WM (2003) Fibulins: physiological and disease perspectives. EMBO Rep 4: 1127–1131. 14647206 10.1038/sj.embor.7400033 PMC1326425

[22] Argraves WS, Tanaka A, Smith EP, Twal WO, Argraves KM, et al (2009) Fibulin-1 and fibrinogen in human atherosclerotic lesions. Histochem Cell Biol 132: 559–565. 19693531 10.1007/s00418-009-0628-7

[23] Hu B, Thirtamara-Rajamani KK, Sim H, Viapiano MS (2009) Fibulin-3 is uniquely upregulated in malignant gliomas and promotes tumor cell motility and invasion. Mol Cancer Res 7: 1756–1770. 19887559 10.1158/1541-7786.MCR-09-0207 PMC3896096

[24] Sottrup-Jensen L (1989) Alpha-macroglobulins: structure, shape, and mechanism of proteinase complex formation. J Biol Chem 264: 11539–11542. 2473064

[25] French K, Yerbury JJ, Wilson MR (2008) Protease activation of alpha2-macroglobulin modulates a chaperone-like action with broad specificity. Biochemistry 47: 1176–1185. 18171086 10.1021/bi701976f

[26] Zhang N, Wang X, Huo Q, Li X, Wang H, et al (2013) The oncogene metadherin modulates the apoptotic pathway based on the tumor necrosis factor superfamily member TRAIL (Tumor Necrosis Factor-related Apoptosis-inducing Ligand) in breast cancer. J Biol Chem 288: 9396–9407. 23408429 10.1074/jbc.M112.395913 PMC3611009

[27] Kim JY, Welsh EA, Oguz U, Fang B, Bai Y, et al (2013) Dissection of TBK1 signaling via phosphoproteomics in lung cancer cells. Proc Natl Acad Sci U S A 110: 12414–12419. 23836654 10.1073/pnas.1220674110 PMC3725062

[28] Kostyukova AS (2007) Leiomodin/tropomyosin interactions are isoform specific. Arch Biochem Biophys 465: 227–230. 17572376 10.1016/j.abb.2007.05.012

[29] Clark R, Nosie A, Walker T, Faralli JA, Filla MS, et al (2013) Comparative genomic and proteomic analysis of cytoskeletal changes in dexamethasone-treated trabecular meshwork cells. Mol Cell Proteomics 12: 194–206. 23105009 10.1074/mcp.M112.019745 PMC3536900

[30] Huet E, Gabison EE, Mourah S, Menashi S (2008) Role of emmprin/CD147 in tissue remodeling. Connect Tissue Res 49: 175–179. 18661337 10.1080/03008200802151722

[31] Wilson MC, Meredith D, Fox JE, Manoharan C, Davies AJ, et al (2005) Basigin (CD147) is the target for organomercurial inhibition of monocarboxylate transporter isoforms 1 and 4: the ancillary protein for the insensitive MCT2 is EMBIGIN (gp70). J Biol Chem 280: 27213–27221. 15917240 10.1074/jbc.M411950200

[32] Wilson MC, Meredith D, Halestrap AP (2002) Fluorescence resonance energy transfer studies on the interaction between the lactate transporter MCT1 and CD147 provide information on the topology and stoichiometry of the complex in situ. J Biol Chem 277: 3666–3672. 11719518 10.1074/jbc.M109658200

[33] Cui HY, Guo T, Wang SJ, Zhao P, Dong ZS, et al (2012) Dimerization is essential for HAb18G/CD147 promoting tumor invasion via MAPK pathway. Biochem Biophys Res Commun 419: 517–522. 22366034 10.1016/j.bbrc.2012.02.049

[34] Hager HA, Bader DM (2009) Bves: ten years after. Histol Histopathol 24: 777–787. 19337975 10.14670/hh-24.777 PMC2853719

[35] Froese A, Breher SS, Waldeyer C, Schindler RF, Nikolaev VO, et al (2012) Popeye domain containing proteins are essential for stress-mediated modulation of cardiac pacemaking in mice. J Clin Invest 122: 1119–1130. 22354168 10.1172/JCI59410 PMC3287222

[36] Gingold-Belfer R, Bergman M, Alcalay Y, Schlesinger H, Aravot D, et al (2011) Popeye domain-containing 1 is down-regulated in failing human hearts. Int J Mol Med 27: 25–31. 21069264 10.3892/ijmm.2010.558

[37] Russ PK, Kupperman AI, Presley SH, Haselton FR, Chang MS (2010) Inhibition of RhoA signaling with increased Bves in trabecular meshwork cells. Invest Ophthalmol Vis Sci 51: 223–230. 19628742 10.1167/iovs.09-3539 PMC2857412

[38] Warren SA, Briggs LE, Zeng H, Chuang J, Chang EI, et al (2012) Myosin light chain phosphorylation is critical for adaptation to cardiac stress. Circulation 126: 2575–2588. 23095280 10.1161/CIRCULATIONAHA.112.116202 PMC3510779

[39] Sanger JM, Wang J, Gleason LM, Chowrashi P, Dube DK, et al (2010) Arg/Abl-binding protein, a Z-body and Z-band protein, binds sarcomeric, costameric, and signaling molecules. Cytoskeleton (Hoboken) 67: 808–823. 20886612 10.1002/cm.20490 PMC3019100

[40] Kakimoto Y, Ito S, Abiru H, Kotani H, Ozeki M, et al (2013) Sorbin and SH3 domain-containing protein 2 is released from infarcted heart in the very early phase: proteomic analysis of cardiac tissues from patients. J Am Heart Assoc 2: e000565. 24342996 10.1161/JAHA.113.000565 PMC3886759

[41] Soubeyran P, Barac A, Szymkiewicz I, Dikic I (2003) Cbl-ArgBP2 complex mediates ubiquitination and degradation of c-Abl. Biochem J 370: 29–34. 12475393 10.1042/BJ20021539 PMC1223168

[42] Frank D, Kuhn C, Katus HA, Frey N (2006) The sarcomeric Z-disc: a nodal point in signalling and disease. J Mol Med (Berl) 84: 446–468. 16416311 10.1007/s00109-005-0033-1

[43] Korotchkina LG, Patel MS (1995) Mutagenesis studies of the phosphorylation sites of recombinant human pyruvate dehydrogenase. Site-specific regulation. J Biol Chem 270: 14297–14304. 7782287 10.1074/jbc.270.24.14297

[44] Karpova T, Danchuk S, Kolobova E, Popov KM (2003) Characterization of the isozymes of pyruvate dehydrogenase phosphatase: implications for the regulation of pyruvate dehydrogenase activity. Biochim Biophys Acta 1652: 126–135. 14644048 10.1016/j.bbapap.2003.08.010

[45] Lopaschuk GD, Ussher JR, Folmes CD, Jaswal JS, Stanley WC (2010) Myocardial fatty acid metabolism in health and disease. Physiol Rev 90: 207–258. 20086077 10.1152/physrev.00015.2009

[46] Bolanos-Garcia VM, Fernandez-Recio J, Allende JE, Blundell TL (2006) Identifying interaction motifs in CK2beta–a ubiquitous kinase regulatory subunit. Trends Biochem Sci 31: 654–661. 17084631 10.1016/j.tibs.2006.10.005

[47] Veis A, Sfeir C, Wu CB (1997) Phosphorylation of the proteins of the extracellular matrix of mineralized tissues by casein kinase-like activity. Crit Rev Oral Biol Med 8: 360–379. 9391750 10.1177/10454411970080040101

[48] Tan WQ, Wang JX, Lin ZQ, Li YR, Lin Y, et al (2008) Novel cardiac apoptotic pathway: the dephosphorylation of apoptosis repressor with caspase recruitment domain by calcineurin. Circulation 118: 2268–2276. 19001025 10.1161/CIRCULATIONAHA.107.750869

[49] Li PF, Li J, Muller EC, Otto A, Dietz R, et al (2002) Phosphorylation by protein kinase CK2: a signaling switch for the caspase-inhibiting protein ARC. Mol Cell 10: 247–258. 12191471 10.1016/s1097-2765(02)00600-7

[50] Eom GH, Cho YK, Ko JH, Shin S, Choe N, et al (2011) Casein kinase-2alpha1 induces hypertrophic response by phosphorylation of histone deacetylase 2 S394 and its activation in the heart. Circulation 123: 2392–2403. 21576649 10.1161/CIRCULATIONAHA.110.003665

[51] Murtaza I, Wang HX, Feng X, Alenina N, Bader M, et al (2008) Down-regulation of catalase and oxidative modification of protein kinase CK2 lead to the failure of apoptosis repressor with caspase recruitment domain to inhibit cardiomyocyte hypertrophy. J Biol Chem 283: 5996–6004. 18171680 10.1074/jbc.M706466200

[52] Hauck L, Harms C, An J, Rohne J, Gertz K, et al (2008) Protein kinase CK2 links extracellular growth factor signaling with the control of p27(Kip1) stability in the heart. Nat Med 14: 315–324. 18311148 10.1038/nm1729

[53] Kim SO, Baines CP, Critz SD, Pelech SL, Katz S, et al (1999) Ischemia induced activation of heat shock protein 27 kinases and casein kinase 2 in the preconditioned rabbit heart. Biochem Cell Biol 77: 559–567. 10668633

[54] Konecny F, Zou J, Husain M, von Harsdorf R (2012) Post-myocardial infarct p27 fusion protein intravenous delivery averts adverse remodelling and improves heart function and survival in rodents. Cardiovasc Res 94: 492–500. 22492676 10.1093/cvr/cvs138

[55] Liebler DC, Zimmerman LJ (2013) Targeted quantitation of proteins by mass spectrometry. Biochemistry 52: 3797–3806. 23517332 10.1021/bi400110b PMC3674507

