# Supplementary material for: Phosphoproteomic Profiling of Human Myocardial Tissues Distinguishes Ischemic from Non-Ischemic End Stage Heart Failure
Source: PLoS One. 2014 Aug 12;9(8):e104157. doi: 10.1371/journal.pone.0104157 (PMC4130503; doi:10.1371/journal.pone.0104157)
Supplement: Methods S1 — Supplemental Materials and Methods. (DOCX) [file pone.0104157.s002.docx]

**Supporting**

**Materials and Methods.**

**Human Cardiac Tissue Acquisition and Tissue Repository**

1-2mm thick were obtained from the anterolateral LV free wall and immediately flash frozen in liquid nitrogen and stored in a -80 ˚C freezer. In the ischemic hearts, the area of infarct was identified at the time of tissue procurement and only sites remote from the infarct with grossly transmural muscle and minimal scar were used in this study.

**Sample Preparation for Mass Spectrometry**

Heart tissue samples were homogenized in 1 mL TRIzol (Life Technologies, Grand Island NY) per 0.1 mg heart tissue. After tissue homogenization using an electric homogenizer (BioSPEC Products Inc., Model 985-370) and centrifugation, 0.8 mL of the supernatant fraction was subjected to phase separation by addition of 0.2 mL chloroform. Following centrifugation, protein was precipitated from the organic layer by addition of 1.2 mL methanol. After washing and sonication of the pellet, the protein precipitate was recovered by centrifugation and re-suspended in 0.2 mL of 0.25% w/v mass spectrometry (MS)-compatible detergent (RapiGest, Waters Corp., Milford, MA) in 50 mM Ammonium Bicarbonate pH 8.0. A 625 µg aliquot of protein (per sample) was subjected to reduction (10 mM dithiothreitol, 80°C for 30 min), alkylation (20 mM iodoacetamide, RT in dark for 1 h) followed by overnight proteolysis with 1:50 w/w sequencing grade trypsin (Promega, Madison, WI) at 37 °C. A 25 µg aliquot from each sample was used for unenriched proteomic analysis of protein expression in the heart tissue. The 25 µg unenriched proteomics profiling aliquot was acidified to 1% v/v final Trifluoroacetic acid (TFA), heated to 60 °C for 2 h, and spiked with 1.25 pmol ADH1_YEAST digest (Massprep standard, Waters Corporation) as a surrogate standard prior to analysis.

The remaining 600 μg of protein was utilized for spin-column based phosphopeptide enrichment and LC/MS/MS analysis of the phosphoproteome. Prior to phosphopeptide enrichment, each sample was spiked with trypsin digested bovine alpha-casein at 30 fmol per μg/protein lysate for use as a surrogate standard. These samples were then enriched for phosphopeptides using an in-house packed TiO_2_ spin columns as previously described [[1](#_ENREF_1)]. Briefly, samples were dried using vacuum centrifugation and re-suspended in 100 μL 80% acetonitrile, 50 mg/mL MassPrep enhancer (Waters Corp.), 1% TFA (pH 2.5). Samples were then loaded onto a TiO_2_ column containing approximately 12 mg TiO_2_ resin (Protea Biosciences Group, Inc., Morgantown, WV) which were subsequently washed with 400 μL 80% acetonitrile, 50 mg/mL MassPrep enhancer, 1% TFA (pH 2.5) and then 400 μL 80% acetonitrile, 1% TFA (pH 2.5). Phosphopeptides were eluted using 200 μL 5% aqueous ammonia, 20% acetonitrile (pH 10.5) and were immediately acidified with neat formic acid down to pH 3.5. Samples were dried using vacuum centrifugation and then re-suspended in 2% acetonitrile, 0.1% TFA, 10 mM citric acid (pH 2.5) prior to LC/MS/MS analysis.

**LC/MS/MS Data collection and processing**

For proteomics or unenriched samples, 1 μg of peptides were first trapped at 20 μL/min for 2 min in 99.9% water with 0.1% v/v formic acid on a 20 μm × 180 mm Symmetry C18 column. Peptides were eluted from the trapping column onto a 75 μm × 250 mm column with 1.7 μm C18 BEH particles (Waters, Corp.). Peptide separations were accomplished using a 90-min gradient of 5 to 40% acetonitrile (0.1% formic acid) at a flow rate of 0.3μl/min and a 45 °C column temperature. MS and MS/MS data was collected using data-independent analysis (MS^E^) for simultaneous peptide quantification and identification using a 0.9 s cycle time, alternating between MS (low collision energy - 6 V) and MS/MS (high collision energy ramp - 15 to 40 V). These qualitative/quantitative analyses were followed by an additional, supplementary qualitative LC/MS/MS experiment using data-dependent analysis (DDA) with a 0.9 s MS scan followed by MS/MS acquisition on the ‘top 3’ ions with charge greater than 1. The MS/MS acquisition for each ion used an isolation window of approximately 3 Da, a maximum of 4 s per precursor, and dynamic exclusion for 120 s (within 1.2 Da).

LTQ-Orbitrap phosphopeptide analyses was performed using the same nanoscale capillary LC column hardware and LC system that was employed for unenriched proteome analysis except that the gradient was modified by increasing the trapping time to 5 min and then a gradient hold at 5% acetonitrile (0.1% formic acid) for 5 min prior to initiating the linear gradient from 5 to 40% acetonitrile (0.1% formic acid). MS data were acquired in the Orbitrap from m/z 400–2000 with r = 60,000 at m/z 400 and a target AGC setting of 1^6^ ions. The qualitative/quantitative LC/MS/MS analyses spectra utilized DDA for the ‘top 3’ precursor ions and supplementary qualitative LC/MS/MS analyses used DDA for the ‘top 10’ precursor ions. Peptide fragmentation was performed in the LTQ linear ion trap, with a CID energy setting of 35% and a dynamic exclusion of 60 s.

**LC-MS Data Processing**

Unenriched and phospho-enriched proteomics datasets were independently aligned on the basis of their accurate mass and retention time. After alignment and annotation, chromatographic peak intensities belonging to the same precursor mass in the aligned chromatograms were then used to calculate the relative peptide and protein abundance on a per-sample basis. MS^E^ from the Q-ToF was used exclusively for peptide quantitation of unenriched proteomes. Protein intensities for each sample were calculated as the simple sum of the peptide intensity values. Phosphopeptide quantitation was performed on the LTQ-Orbitrap XL instrument at the peptide level from the qualitative/quantitative acquisitions.

Both MS/MS DDA and MS^E^ were used to generate peptide identifications for the unenriched analysis, and DDA exclusively for phosphopeptides. For DDA acquisition files, .mgf searchable files were produced in Rosetta Elucidator®, and searches were then submitted to and retrieved from the Mascot v2.2 (Matrix Sciences, Boston, MA) search engine in an automated fashion. For MS^E^ data, ProteinLynx Global Server 2.4 (Waters Corp.) was used to generate searchable files which were then submitted to the IdentityE search engine (Waters Corp.) [[2](#_ENREF_2),[3](#_ENREF_3)] and results files were then imported back into Elucidator®.

Both DDA and MS^E^ data were searched against the Uniprot/reviewed database with *human* taxonomy ([www.uniprot.org](http://www.uniprot.org), downloaded March 23, 2009) with full 1X reverse database appended for peptide false discovery rate determination. The final database contained 40,668 sequences including reverse entries. Q-ToF data (unenriched proteome), used a precursor ion mass tolerance of 20 ppm for both PLGS and Mascot database (DB) searches, and a product ion tolerance of 0.1 Da for Mascot and 40 ppm for PLGS. Orbitrap data (phosphoproteomics) was searched with Mascot using 10 ppm precursor and 0.8 Da product ion tolerances. Enzyme specificity was set to fully tryptic and allowed for up to 2 missed cleavages, with the exception that semi-tryptic specificity was allowed for Mascot (DDA) searches of unenriched data. Carbamidomethyl cysteine was included as a fixed modification, and variable modifications were allowed for including oxidized methionine and deamidated asparagine and glutamine. Additionally, for phosphopeptide enriched mixtures, variable phosphorylation on serine, threonine, and tyrosine was allowed.

The spectra were submitted for database searching and results were imported into Elucidator®. To enable global spectra scoring across results from both search engines these search results were concurrently validated using the PeptideProphet and ProteinProphet algorithms in Elucidator® using an independent reverse decoy database validation [[4](#_ENREF_4),[5](#_ENREF_5)]. Annotation was performed to achieve a maximum 1% FDR at the peptide level, which corresponded to a minimum PeptideProphet score of 0.6. Each peptide identified was allowed to be assigned to only a single protein entry, and these assignments were made by ProteinProphet according to the rules of parsimony. For the phosphoproteomic experiments, a mascot ion score of 26 was applied to achieve a spectral false discovery rate of 1.0%.

**Western Blot Analysis**

Cryopreserved heart tissue sample of each of the 12 hearts examined in the LC/MS/MS analysis were weighed and mechanically disrupted by mortar and pestle in liquid nitrogen. Pulverized heart tissues were suspended in a 5:1 volume-to-tissue weight of lysis buffer (1% IGEPAL CA-630, 0.5% Deoxycholate, 2% SDS, 5mM EDTA in 1X PBS) with protease and phosphatase inhibitor cocktail tablets (Roche Diagnostics, Indianapolis, IN). Samples were then pulse homogenized on ice with a handheld tissue tearor (BioSPEC Products Inc., Model 985-370). Homogenates were placed on ice for an additional 30 min followed by centrifugation for 30 min at 4°C at 16,000xg (Heraeus Biofuge® Pico). Resulting supernatants were aliquoted and stored at -80 °C until analysis.

Cardiac tissue homogenates were subjected to Bicinchoninic acid (BCA) assay (Pierce Biotechnology/Thermo Fisher Scientific, Rockford, IL) for protein quantification. Western Blot for protein immunodetection was performed using a modification of a recently published method [[6](#_ENREF_6)]. The primary antibodies used in this study were the following: anti-Fetuin A (#5258 Cell Signaling Technologies, Danver, MA), anti-fibulin 1 (ab54652 Abcam, Cambridge, MA), anti-ceruloplasmin (ab8813, Abcam), anti-alpha 2 macroglobulin (ab58703, Abcam), anti-carbonic anhydrase I (ab6619-1, Abcam), anti-serum amyloid A (ab687, Abcam), anti-fibulin 2 (ab66333, Abcam), anti-AKT (#9272, Cell Signaling), anti-SMAD3 (ab28379, Abcam), anti-MMP14 (ab51074, Abcam), and anti-AHR (ab28698, Abcam).

The secondary antibodies used were horseradish peroxidase conjugated anti-rabbit IgG (GE Healthcare, UK), anti-mouse IgG (Pierce), anti-sheep IgG (ab6747, Abcam), or anti-goat IgG (Sigma-Aldrich, St. Louis, MO). Bands were visualized using an enhanced chemiluminescence Western blotting detection system (GE Healthcare Bio-Sciences, Piscataway, NJ). Western blots were stripped and re-probed with anti-sarcomeric actin (Sigma, A2172). The intensity of the actin band signal was used for normalization. Proteins detected were quantitated by densitometry utilizing the Image J algorithm (National Institutes of Health, Bethesda, MD).

References

1. Soderblom EJ, Philipp M, Thompson JW, Caron MG, Moseley MA (2011) Quantitative label-free phosphoproteomics strategy for multifaceted experimental designs. Anal Chem 83: 3758-3764.

2. Geromanos SJ, Vissers JP, Silva JC, Dorschel CA, Li GZ, et al. (2009) The detection, correlation, and comparison of peptide precursor and product ions from data independent LC-MS with data dependant LC-MS/MS. Proteomics 9: 1683-1695.

3. Li GZ, Vissers JP, Silva JC, Golick D, Gorenstein MV, et al. (2009) Database searching and accounting of multiplexed precursor and product ion spectra from the data independent analysis of simple and complex peptide mixtures. Proteomics 9: 1696-1719.

4. Keller A, Nesvizhskii AI, Kolker E, Aebersold R (2002) Empirical statistical model to estimate the accuracy of peptide identifications made by MS/MS and database search. Anal Chem 74: 5383-5392.

5. Nesvizhskii AI, Keller A, Kolker E, Aebersold R (2003) A statistical model for identifying proteins by tandem mass spectrometry. Anal Chem 75: 4646-4658.

6. Piacentino V, 3rd, Milano CA, Bolanos M, Schroder J, Messina E, et al. (2012) X-linked inhibitor of apoptosis protein-mediated attenuation of apoptosis, using a novel cardiac-enhanced adeno-associated viral vector. Hum Gene Ther 23: 635-646.
