# Supplementary material for: Phosphoproteomic Profiling of Human Myocardial Tissues Distinguishes Ischemic from Non-Ischemic End Stage Heart Failure
Source: PLoS One. 2014 Aug 12;9(8):e104157. doi: 10.1371/journal.pone.0104157 (PMC4130503; doi:10.1371/journal.pone.0104157)
Supplement: Table S1 — Unenriched protein expression profiles. (PDF) [file pone.0104157.s003.pdf]

SUPPLEMENTAL TABLES

SUPPLEMENTAL TABLE 1 UNENRICHED PROTEIN EXPRESSION PROFILES

Entry Name	Protein Description	ProteinTeller Probability	Peptide Count	NIF v IF Fold Change	NIF v IF p-value (ANOVA)	IF v NF Fold Change	IF v NF p-value (ANOVA)	NIF v NF Fold Change	NIF v NF p-value (ANOVA)
1433B_HUMAN	14-3-3 protein beta/alpha OS=Homo sapiens GN=YWHAB PE=1 SV=3	1	11	1.02	9.6E-01	1.06	6.9E-01	1.08	3.9E-01
1433E_HUMAN	14-3-3 protein epsilon OS=Homo sapiens GN=YWHAE PE=1 SV=1	1	7	1.02	9.7E-01	-1.08	8.0E-01	-1.06	8.1E-01
1433F_HUMAN	14-3-3 protein eta OS=Homo sapiens GN=YWHAH PE=1 SV=4	0.87	2	-1.24	6.8E-01	-1.24	4.9E-01	-1.53	2.2E-01
1433G_HUMAN	14-3-3 protein gamma OS=Homo sapiens GN=YWHAG PE=1 SV=2	1	10	1.14	7.8E-01	-1.18	4.2E-01	-1.03	8.4E-01
1433T_HUMAN	14-3-3 protein theta OS=Homo sapiens GN=YWHAQ PE=1 SV=1	1	5	-1.11	8.7E-01	1.16	5.8E-01	1.05	8.6E-01
1433Z_HUMAN	14-3-3 protein zeta/delta OS=Homo sapiens GN=YWHAZ PE=1 SV=1	1	7	1.08	9.2E-01	-1.13	6.2E-01	-1.05	6.7E-01
A1AG1_HUMAN	Alpha-1-acid glycoprotein 1 OS=Homo sapiens GN=ORM1 PE=1 SV=1	1	13	-1.14	8.8E-01	1.43	3.0E-01	1.25	5.4E-01
A1AG2_HUMAN	Alpha-1-acid glycoprotein 2 OS=Homo sapiens GN=ORM2 PE=1 SV=2	1	21	-1.16	7.7E-01	1.53	9.7E-02	1.31	2.9E-01
A1AT_HUMAN	Alpha-1-antitrypsin OS=Homo sapiens GN=SERPINA1 PE=1 SV=3	1	57	-1.03	9.7E-01	1.29	2.0E-01	1.25	4.4E-01
A1BG_HUMAN	Alpha-1B-glycoprotein OS=Homo sapiens GN=A1BG PE=1 SV=3	1	19	-1.20	7.5E-01	1.77	3.8E-02	1.48	1.7E-01
A26CA_HUMAN	ANKRD26-like family C member 1A - Homo sapiens	1	32	-1.01	9.7E-01	1.01	8.8E-01	1.00	8.7E-01
A26CB_HUMAN	ANKRD26-like family C member 1B - Homo sapiens	1	8	1.09	8.7E-01	-1.08	5.2E-01	1.00	9.7E-01
A2GL_HUMAN	Leucine-rich alpha-2-glycoprotein OS=Homo sapiens GN=LRG1 PE=1 SV=2	1	12	-1.46	7.1E-01	1.02	9.7E-01	-1.43	4.0E-01
A2MG_HUMAN	Alpha-2-macroglobulin OS=Homo sapiens GN=A2M PE=1 SV=1	1	33	-1.25	6.8E-01	2.31	1.0E-04	1.84	2.0E-04
AACT_HUMAN	Alpha-1-antichymotrypsin OS=Homo sapiens GN=SERPINA3 PE=1 SV=2	1	26	-1.11	9.4E-01	-1.07	7.9E-01	-1.19	6.1E-01
AATC_HUMAN	Aspartate aminotransferase, cytoplasmic OS=Homo sapiens GN=GOT1 PE=1 SV=3	0.84	2	-1.15	8.7E-01	-1.19	4.7E-01	-1.37	1.1E-01
ABCAD_HUMAN	ATP-binding cassette sub-family A member 13 OS=Homo sapiens GN=ABCA13 PE=2 SV=2	0	1	-1.02	9.7E-01	1.12	4.1E-01	1.11	4.2E-01
ACADM_HUMAN	Medium-chain specific acyl-CoA dehydrogenase, mitochondrial OS=Homo sapiens GN=ACADM PE=1 SV=1	1	5	1.36	2.1E-01	-1.13	5.9E-01	1.21	4.5E-01
ACPM_HUMAN	Acyl carrier protein, mitochondrial OS=Homo sapiens GN=NDUFAB1 PE=1 SV=3	1	6	-1.04	9.7E-01	-1.28	1.8E-01	-1.33	2.5E-01
ACTA_HUMAN	Actin, aortic smooth muscle OS=Homo sapiens GN=ACTA2 PE=1 SV=1	1	10	-1.00	9.9E-01	-1.39	3.3E-01	-1.40	1.4E-01
ACTB_HUMAN	Actin, cytoplasmic 1 OS=Homo sapiens GN=ACTB PE=1 SV=1	1	10	1.27	6.8E-01	-1.11	6.6E-01	1.15	5.0E-01
ACTBL_HUMAN	Beta-actin-like protein 2 OS=Homo sapiens GN=ACTBL2 PE=1 SV=2	1	12	1.04	8.7E-01	-1.15	5.7E-02	-1.11	1.4E-01
ACTK_HUMAN	Kappa-actin - Homo sapiens	1	8	1.04	9.4E-01	-1.46	2.3E-02	-1.40	1.8E-04
ACTS_HUMAN	Actin, alpha skeletal muscle OS=Homo sapiens GN=ACTA1 PE=1 SV=1	1	49	1.04	9.4E-01	-1.18	4.6E-01	-1.14	5.0E-01
ADH1_YEAST	Alcohol dehydrogenase 1 OS=Saccharomyces cerevisiae GN=ADH1 PE=1 SV=4	1	21	1.04	8.1E-01	1.07	2.7E-01	1.11	2.2E-02
ADH1G_HUMAN	Alcohol dehydrogenase 1C OS=Homo sapiens GN=ADH1C PE=1 SV=2	0	1	-1.04	9.7E-01	1.29	7.3E-01	1.23	7.4E-01
ADIP_HUMAN	Afadin- and alpha-actinin-binding protein OS=Homo sapiens GN=SSX2IP PE=1 SV=3	0	1	-1.35	3.4E-01	-1.41	5.6E-02	-1.90	8.6E-07
ADIPO_HUMAN	Adiponectin OS=Homo sapiens GN=ADIPOQ PE=1 SV=1	0	1	-1.01	1.0E+00	-1.23	4.1E-01	-1.24	3.1E-01
AHNK2_HUMAN	Protein AHNAK2 OS=Homo sapiens GN=AHNAK2 PE=1 SV=2	0.55	1	-1.17	8.7E-01	1.08	8.6E-01	-1.09	7.3E-01
AIFM1_HUMAN	Apoptosis-inducing factor 1, mitochondrial OS=Homo sapiens GN=AIFM1 PE=1 SV=1	0.91	1	1.08	9.4E-01	-1.47	4.1E-01	-1.36	4.1E-01
AIM1_HUMAN	Absent in melanoma 1 protein OS=Homo sapiens GN=AIM1 PE=1 SV=3	0.34	1	-1.36	7.1E-01	1.56	1.4E-01	1.14	6.7E-01
ALBU_HUMAN	Serum albumin OS=Homo sapiens GN=ALB PE=1 SV=2	1	242	1.07	6.8E-01	1.36	5.0E-07	1.46	1.9E-06
ALDOA_HUMAN	Fructose-bisphosphate aldolase A OS=Homo sapiens GN=ALDOA PE=1 SV=2	1	24	1.10	8.2E-01	-1.25	4.1E-01	-1.13	5.4E-01
ALDOC_HUMAN	Fructose-bisphosphate aldolase C OS=Homo sapiens GN=ALDOC PE=1 SV=2	1	12	-1.01	9.7E-01	-1.08	7.1E-01	-1.09	5.4E-01
ALPK2_HUMAN	Alpha-protein kinase 2 OS=Homo sapiens GN=ALPK2 PE=1 SV=3	0	1	1.27	7.3E-01	-1.12	7.1E-01	1.14	6.4E-01
AMBP_HUMAN	Protein AMBP OS=Homo sapiens GN=AMBP PE=1 SV=1	1	9	-1.42	4.3E-01	1.50	5.4E-02	1.05	8.3E-01
ANGT_HUMAN	Angiotensinogen OS=Homo sapiens GN=AGT PE=1 SV=1	0.83	1	1.21	8.7E-01	1.29	6.4E-01	1.56	3.5E-01
ANT3_HUMAN	Antithrombin-III OS=Homo sapiens GN=SERPINC1 PE=1 SV=1	1	16	-1.32	4.3E-01	1.27	4.3E-01	-1.04	7.9E-01
ANXA2_HUMAN	Annexin A2 OS=Homo sapiens GN=ANXA2 PE=1 SV=2	1	5	-1.00	9.5E-01	1.13	6.6E-01	1.13	5.4E-01
ANXA5_HUMAN	Annexin A5 OS=Homo sapiens GN=ANXA5 PE=1 SV=2	1	6	1.13	9.4E-01	1.28	4.2E-01	1.45	1.1E-02
AOC3_HUMAN	Membrane primary amine oxidase OS=Homo sapiens GN=AOC3 PE=1 SV=3	1	9	-1.02	9.6E-01	1.13	7.8E-01	1.11	7.4E-01
APOA4_HUMAN	Apolipoprotein A-IV OS=Homo sapiens GN=APOA4 PE=1 SV=3	0.31	1	-1.07	8.9E-01	1.27	3.1E-01	1.19	2.5E-01
APOH_HUMAN	Beta-2-glycoprotein 1 OS=Homo sapiens GN=APOH PE=1 SV=3	1	17	-1.21	7.8E-01	1.56	8.1E-02	1.29	4.6E-01
APOOL_HUMAN	Apolipoprotein O-like OS=Homo sapiens GN=APOOL PE=1 SV=1	0.83	1	1.04	9.3E-01	-1.23	2.1E-01	-1.17	2.4E-01
ARGFX_HUMAN	Arginine-fifty homeobox OS=Homo sapiens GN=ARGFX PE=2 SV=1	0	1	-1.39	6.8E-01	1.76	2.3E-02	1.27	3.5E-01
ARHGH_HUMAN	Rho guanine nucleotide exchange factor 17 OS=Homo sapiens GN=ARHGEF17 PE=1 SV=1	0.68	1	-1.17	8.2E-01	-1.17	4.9E-01	-1.37	1.2E-01
ARHGJ_HUMAN	Rho guanine nucleotide exchange factor 19 OS=Homo sapiens GN=ARHGEF19 PE=2 SV=1	0	1	1.13	6.8E-01	-1.05	7.9E-01	1.08	5.9E-01
ASAH1_HUMAN	Acid ceramidase OS=Homo sapiens GN=ASAH1 PE=1 SV=5	1	22	-1.29	2.3E-02	-1.19	1.3E-01	-1.53	8.2E-08
ASPN_HUMAN	Asporin OS=Homo sapiens GN=ASPN PE=1 SV=2	1	9	1.64	6.8E-01	1.84	2.1E-01	3.03	1.5E-02
AT1B1_HUMAN	Sodium/potassium-transporting ATPase subunit beta-1 OS=Homo sapiens GN=ATP1B1 PE=1 SV=1	1	14	-1.17	6.8E-01	-1.17	1.3E-01	-1.38	2.3E-02
ATPB_HUMAN	ATP synthase subunit beta, mitochondrial OS=Homo sapiens GN=ATP5B PE=1 SV=3	1	13	1.02	9.6E-01	-1.26	6.8E-01	-1.23	2.5E-01
ATPD_HUMAN	ATP synthase subunit delta, mitochondrial OS=Homo sapiens GN=ATP5D PE=1 SV=2	0.97	2	-1.34	7.3E-01	-1.66	1.5E-01	-2.22	6.2E-04
ATPG_HUMAN	ATP synthase subunit gamma, mitochondrial OS=Homo sapiens GN=ATP5C1 PE=1 SV=1	1	6	1.14	5.0E-01	-1.47	2.8E-03	-1.29	1.4E-02
ATPK_HUMAN	ATP synthase subunit f, mitochondrial OS=Homo sapiens GN=ATP5J2 PE=1 SV=3	0.83	1	-1.11	8.8E-01	-1.50	1.3E-02	-1.66	2.8E-02
ATS20_HUMAN	A disintegrin and metalloproteinase with thrombospondin motifs 20 OS=Homo sapiens GN=ADAMTS20 PE=2 SV=2	0	1	1.05	9.3E-01	1.47	5.1E-05	1.54	4.9E-03
AXA2L_HUMAN	Putative annexin A2-like protein OS=Homo sapiens GN=ANXA2P2 PE=5 SV=2	1	18	1.05	9.4E-01	-1.02	9.8E-01	1.03	7.4E-01
BASI_HUMAN	Basigin OS=Homo sapiens GN=BSG PE=1 SV=2	1	14	-1.13	8.1E-01	-1.19	2.7E-01	-1.35	5.0E-02
BIEA_HUMAN	Biliverdin reductase A OS=Homo sapiens GN=BLVRA PE=1 SV=2	0.8	2	-1.04	8.9E-01	1.50	2.8E-03	1.44	1.8E-03
BST2_HUMAN	Bone marrow stromal antigen 2 OS=Homo sapiens GN=BST2 PE=1 SV=1	0.83	1	-1.18	8.8E-01	-1.35	4.5E-01	-1.59	1.8E-01
BUD13_HUMAN	BUD13 homolog OS=Homo sapiens GN=BUD13 PE=1 SV=1	0.31	1	1.03	9.4E-01	1.56	7.8E-04	1.60	9.0E-05
C1QBP_HUMAN	Complement component 1 Q subcomponent-binding protein, mitochondrial OS=Homo sapiens GN=C1QBP PE=1 SV=1	1	10	-1.14	6.8E-01	-1.17	2.2E-02	-1.33	4.5E-03
CAD13_HUMAN	Cadherin-13 OS=Homo sapiens GN=CDH13 PE=1 SV=1	1	22	-1.11	8.9E-01	-1.25	3.3E-01	-1.39	1.4E-01
CADH2_HUMAN	Cadherin-2 OS=Homo sapiens GN=CDH2 PE=1 SV=4	1	24	-1.15	7.5E-01	-1.08	7.3E-01	-1.24	1.6E-01
CAH1_HUMAN	Carbonic anhydrase 1 OS=Homo sapiens GN=CA1 PE=1 SV=2	0.98	2	-1.78	6.5E-01	7.24	1.6E-03	4.06	3.1E-04
CAH3_HUMAN	Carbonic anhydrase 3 OS=Homo sapiens GN=CA3 PE=1 SV=3	0.97	2	2.83	3.4E-01	2.48	3.0E-01	7.02	1.2E-02
CALD1_HUMAN	Caldesmon OS=Homo sapiens GN=CALD1 PE=1 SV=2	1	6	1.17	6.8E-01	1.03	8.9E-01	1.21	1.4E-01
CALM_HUMAN	Calmodulin OS=Homo sapiens GN=CALM1 PE=1 SV=2	1	16	1.02	9.5E-01	-1.15	2.1E-01	-1.12	1.4E-01
CALR_HUMAN	Calreticulin OS=Homo sapiens GN=CALR PE=1 SV=1	1	10	-1.08	6.8E-01	-1.11	3.1E-01	-1.20	6.9E-04
CALU_HUMAN	Calumenin OS=Homo sapiens GN=CALU PE=1 SV=2	0.98	2	-1.18	6.8E-01	-1.48	3.6E-02	-1.75	5.0E-14
CAPZB_HUMAN	F-actin-capping protein subunit beta OS=Homo sapiens GN=CAPZB PE=1 SV=4	1	2	1.02	9.7E-01	-1.07	8.4E-01	-1.05	8.2E-01
CASQ2_HUMAN	Calsequestrin-2 OS=Homo sapiens GN=CASQ2 PE=1 SV=2	1	33	-1.23	3.2E-01	-1.03	8.4E-01	-1.27	2.9E-02
CATB_HUMAN	Cathepsin B OS=Homo sapiens GN=CTSB PE=1 SV=3	1	6	1.06	8.9E-01	-1.24	9.4E-02	-1.17	2.4E-01
CATD_HUMAN	Cathepsin D OS=Homo sapiens GN=CTSD PE=1 SV=1	1	29	-1.03	9.4E-01	-1.33	2.3E-02	-1.37	6.5E-03
CAZA2_HUMAN	F-actin-capping protein subunit alpha-2 OS=Homo sapiens GN=CAPZA2 PE=1 SV=3	0.61	1	1.11	8.7E-01	-2.00	3.1E-03	-1.80	5.1E-03
CCD57_HUMAN	Coiled-coil domain-containing protein 57 OS=Homo sapiens GN=CCDC57 PE=2 SV=1	0.32	1	-1.24	9.1E-01	2.05	1.3E-01	1.65	4.4E-01
CD36_HUMAN	Platelet glycoprotein 4 OS=Homo sapiens GN=CD36 PE=1 SV=2	1	19	-1.44	8.4E-02	-1.01	6.8E-01	-1.45	8.3E-02
CD59_HUMAN	CD59 glycoprotein OS=Homo sapiens GN=CD59 PE=1 SV=1	1	8	-1.09	8.7E-01	-1.22	4.3E-01	-1.33	3.0E-02
CD99_HUMAN	CD99 antigen OS=Homo sapiens GN=CD99 PE=1 SV=1	0.83	1	-1.24	7.5E-01	-1.24	2.8E-01	-1.53	1.2E-01
CERU_HUMAN	Ceruloplasmin OS=Homo sapiens GN=CP PE=1 SV=1	1	15	1.14	8.2E-01	1.83	6.3E-03	2.09	4.0E-03
CFAB_HUMAN	Complement factor B OS=Homo sapiens GN=CFB PE=1 SV=2	1	24	-1.05	9.4E-01	1.79	1.4E-01	1.70	1.6E-01
CFAH_HUMAN	Complement factor H OS=Homo sapiens GN=CFH PE=1 SV=4	1	18	-1.10	9.4E-01	1.61	5.3E-02	1.46	1.4E-01
CH10_HUMAN	10 kDa heat shock protein, mitochondrial OS=Homo sapiens GN=HSPE1 PE=1 SV=2	1	9	1.07	6.8E-01	-1.19	1.3E-03	-1.12	1.2E-01
CHCH3_HUMAN	Coiled-coil-helix-coiled-coil-helix domain-containing protein 3, mitochondrial OS=Homo sapiens GN=CHCHD3 PE=1 SV=1	1	14	-1.10	7.5E-01	-1.16	5.2E-02	-1.28	2.6E-02
CHDH_HUMAN	Choline dehydrogenase, mitochondrial OS=Homo sapiens GN=CHDH PE=2 SV=1	0	1	-1.21	7.8E-01	-1.21	4.6E-01	-1.47	1.4E-01
CHST7_HUMAN	Carbohydrate sulfotransferase 7 OS=Homo sapiens GN=CHST7 PE=1 SV=2	0	1	-1.56	6.8E-01	-1.42	6.2E-01	-2.21	1.0E-01
CISD1_HUMAN	CDGSH iron sulfur domain-containing protein 1 OS=Homo sapiens GN=CISD1 PE=1 SV=1	1	4	-1.16	6.8E-01	-1.29	1.0E-02	-1.49	2.4E-03
CK067_HUMAN	UPF0366 protein C11orf67 OS=Homo sapiens GN=C11orf67 PE=1 SV=1	0.98	2	-1.33	7.5E-01	-1.21	6.2E-01	-1.60	2.4E-01
CLCB_HUMAN	Clathrin light chain B OS=Homo sapiens GN=CLTB PE=1 SV=1	0.83	1	-1.13	6.8E-01	1.05	6.9E-01	-1.07	5.3E-01
CLUS_HUMAN	Clusterin OS=Homo sapiens GN=CLU PE=1 SV=1	1	7	-1.23	6.8E-01	1.47	1.2E-01	1.19	5.3E-01
CMA1_HUMAN	Chymase OS=Homo sapiens GN=CMA1 PE=1 SV=1	1	3	1.37	6.8E-01	-1.30	4.7E-01	1.05	8.2E-01
CN045_HUMAN	Uncharacterized protein C14orf45 OS=Homo sapiens GN=C14orf45 PE=1 SV=3	0	1	1.01	9.9E-01	-1.08	6.9E-01	-1.07	6.7E-01
CNBP_HUMAN	Cellular nucleic acid-binding protein OS=Homo sapiens GN=CNBP PE=1 SV=1	0.83	1	-1.18	7.8E-01	-1.37	1.3E-01	-1.62	1.1E-02
CO4A1_HUMAN	Collagen alpha-1(IV) chain OS=Homo sapiens GN=COL4A1 PE=1 SV=3	0.83	1	-1.01	9.6E-01	1.20	6.7E-01	1.19	5.4E-01
CO4A2_HUMAN	Collagen alpha-2(IV) chain OS=Homo sapiens GN=COL4A2 PE=1 SV=4	0.91	2	1.24	6.8E-01	-1.28	1.4E-01	-1.04	9.0E-01
CO6A1_HUMAN	Collagen alpha-1(VI) chain OS=Homo sapiens GN=COL6A1 PE=1 SV=3	1	9	1.13	6.8E-01	1.29	1.9E-02	1.46	3.5E-04
CO6A2_HUMAN	Collagen alpha-2(VI) chain OS=Homo sapiens GN=COL6A2 PE=1 SV=4	0.61	1	1.34	7.4E-01	1.38	2.6E-01	1.85	2.4E-02
CO6A3_HUMAN	Collagen alpha-3(VI) chain OS=Homo sapiens GN=COL6A3 PE=1 SV=4	1	6	1.67	6.8E-01	1.75	1.8E-01	2.93	5.6E-03
CO9_HUMAN	Complement component C9 OS=Homo sapiens GN=C9 PE=1 SV=2	1	6	-1.15	8.8E-01	1.56	7.8E-02	1.35	4.0E-01
COF2_HUMAN	Cofilin-2 OS=Homo sapiens GN=CFL2 PE=1 SV=1	0.83	1	1.58	3.4E-01	-1.35	4.2E-01	1.17	5.3E-01
COFA1_HUMAN	Collagen alpha-1(XV) chain OS=Homo sapiens GN=COL15A1 PE=1 SV=2	1	9	-1.23	6.8E-01	1.02	9.6E-01	-1.21	3.5E-01
COG1_HUMAN	Conserved oligomeric Golgi complex subunit 1 OS=Homo sapiens GN=COG1 PE=1 SV=1	0.81	2	1.26	4.7E-01	-1.39	2.3E-03	-1.11	5.4E-01
COIA1_HUMAN	Collagen alpha-1(XVIII) chain OS=Homo sapiens GN=COL18A1 PE=1 SV=5	1	3	-1.00	9.6E-01	1.27	5.0E-01	1.27	4.0E-01
CORL2_HUMAN	Ladybird homeobox corepressor 1-like protein OS=Homo sapiens GN=CORL2 PE=1 SV=2	0	1	1.21	8.7E-01	-1.17	7.4E-01	1.04	9.0E-01
COX2_HUMAN	Cytochrome c oxidase subunit 2 OS=Homo sapiens GN=MT-CO2 PE=1 SV=1	0.98	3	1.19	8.7E-01	-1.48	4.2E-01	-1.24	4.5E-01
COX41_HUMAN	Cytochrome c oxidase subunit 4 isoform 1, mitochondrial OS=Homo sapiens GN=COX4I1 PE=1 SV=1	1	15	1.06	8.9E-01	-1.42	3.8E-03	-1.33	1.1E-01
COX5A_HUMAN	Cytochrome c oxidase subunit 5A, mitochondrial OS=Homo sapiens GN=COX5A PE=1 SV=2	1	14	1.09	8.6E-01	-1.53	1.8E-03	-1.40	8.4E-02
COX5B_HUMAN	Cytochrome c oxidase subunit 5B, mitochondrial OS=Homo sapiens GN=COX5B PE=1 SV=2	1	4	1.07	8.7E-01	-1.41	1.2E-02	-1.31	2.5E-01
CRIP2_HUMAN	Cysteine-rich protein 2 OS=Homo sapiens GN=CRIP2 PE=1 SV=1	1	11	-1.00	9.7E-01	1.06	7.3E-01	1.05	5.9E-01
CRYAB_HUMAN	Alpha-crystallin B chain OS=Homo sapiens GN=CRYAB PE=1 SV=2	1	12	-1.10	7.5E-01	-1.02	8.8E-01	-1.12	2.5E-01
CSPG2_HUMAN	Versican core protein OS=Homo sapiens GN=VCAN PE=1 SV=3	1	9	1.50	8.7E-01	1.58	4.9E-01	2.37	1.4E-01
CSRP1_HUMAN	Cysteine and glycine-rich protein 1 OS=Homo sapiens GN=CSRP1 PE=1 SV=3	0.83	1	1.29	7.5E-01	-1.09	8.2E-01	1.18	7.0E-01

CSRP3_HUMAN	Cysteine and glycine-rich protein 3 OS=Homo sapiens GN=CSRP3 PE=1 SV=1	1	39	-1.11	7.1E-01	-1.00	1.0E+00	-1.12	5.4E-01
CX6B1_HUMAN	Cytochrome c oxidase subunit 6B1 OS=Homo sapiens GN=COX6B1 PE=1 SV=2	1	4	1.09	8.7E-01	-1.37	2.1E-02	-1.26	3.0E-01
CX7A1_HUMAN	Cytochrome c oxidase polypeptide 7A1, mitochondrial OS=Homo sapiens GN=COX7A1 PE=1 SV=2	0.83	1	-1.01	9.7E-01	-1.32	1.7E-01	-1.33	3.4E-01
CY1_HUMAN	Cytochrome c1, heme protein, mitochondrial OS=Homo sapiens GN=CYC1 PE=1 SV=2	1	5	-1.09	8.9E-01	-1.23	5.9E-01	-1.34	1.4E-01
CYC_HUMAN	Cytochrome c OS=Homo sapiens GN=CYCS PE=1 SV=2	1	36	-1.03	9.5E-01	-1.17	1.6E-01	-1.20	2.6E-01
DAG1_HUMAN	Dystroglycan OS=Homo sapiens GN=DAG1 PE=1 SV=2	1	7	-1.19	7.5E-01	-1.15	4.1E-01	-1.37	8.6E-02
DECR_HUMAN	2,4-dienoyl-CoA reductase, mitochondrial OS=Homo sapiens GN=DECR1 PE=1 SV=1	0.96	1	1.16	9.2E-01	1.20	7.5E-01	1.40	3.3E-01
DERM_HUMAN	Dermatopontin OS=Homo sapiens GN=DPT PE=2 SV=2	1	5	1.48	6.8E-01	1.40	3.8E-01	2.08	2.8E-02
DESM_HUMAN	Desmin OS=Homo sapiens GN=DES PE=1 SV=3	1	33	1.15	8.7E-01	1.07	8.3E-01	1.23	4.0E-01
DHSB_HUMAN	Succinate dehydrogenase [ubiquinone] iron-sulfur subunit, mitochondrial OS=Homo sapiens GN=SDHB PE=1 SV=3	1	7	-1.01	9.7E-01	-1.22	2.6E-01	-1.24	1.4E-01
DLDH_HUMAN	Dihydrolipoyl dehydrogenase, mitochondrial OS=Homo sapiens GN=DLD PE=1 SV=1	1	5	1.10	8.7E-01	-1.15	6.5E-01	-1.04	8.5E-01
DMC1_HUMAN	Meiotic recombination protein DMC1/LIM15 homolog OS=Homo sapiens GN=DMC1 PE=1 SV=2	0.92	1	-1.17	8.7E-01	1.25	4.5E-01	1.06	8.6E-01
DSG2_HUMAN	Desmoglein-2 OS=Homo sapiens GN=DSG2 PE=1 SV=2	1	4	1.08	9.4E-01	-1.27	4.9E-01	-1.18	5.3E-01
DYH11_HUMAN	Dynein heavy chain 11, axonemal OS=Homo sapiens GN=DNAH11 PE=1 SV=2	0	1	-1.22	8.9E-01	1.12	6.9E-01	-1.09	8.2E-01
DYXC1_HUMAN	Dyslexia susceptibility 1 candidate gene 1 protein OS=Homo sapiens GN=DYX1C1 PE=2 SV=2	0	1	-1.15	5.5E-01	1.03	4.9E-01	-1.12	2.5E-01
ECH1_HUMAN	Delta(3,5)-Delta(2,4)-dienoyl-CoA isomerase, mitochondrial OS=Homo sapiens GN=ECH1 PE=1 SV=2	0.47	1	1.25	7.3E-01	-1.33	4.4E-01	-1.07	6.7E-01
ECHM_HUMAN	Enoyl-CoA hydratase, mitochondrial OS=Homo sapiens GN=ECHS1 PE=1 SV=4	1	7	1.08	9.4E-01	-1.12	7.5E-01	-1.03	7.9E-01
ECT2_HUMAN	Protein ECT2 OS=Homo sapiens GN=ECT2 PE=1 SV=3	0	1	1.01	9.7E-01	-1.43	1.9E-02	-1.41	1.2E-02
EF1A2_HUMAN	Elongation factor 1-alpha 2 OS=Homo sapiens GN=EEF1A2 PE=1 SV=1	1	11	-1.05	9.5E-01	-1.27	4.2E-01	-1.34	2.5E-01
EF1A3_HUMAN	Putative elongation factor 1-alpha-like 3 OS=Homo sapiens GN=EEF1AL3 PE=5 SV=1	1	12	1.05	9.4E-01	1.17	5.9E-01	1.23	4.0E-01
EF1B_HUMAN	Elongation factor 1-beta OS=Homo sapiens GN=EEF1B2 PE=1 SV=3	0.82	1	-1.13	6.8E-01	1.10	4.5E-01	-1.03	8.6E-01
EFTU_HUMAN	Elongation factor Tu, mitochondrial OS=Homo sapiens GN=TUFM PE=1 SV=2	1	6	1.06	8.7E-01	-1.28	5.3E-02	-1.21	3.3E-02
ENPL_HUMAN	Endoplasmin OS=Homo sapiens GN=HSP90B1 PE=1 SV=1	1	15	1.06	8.9E-01	-1.05	7.8E-01	1.01	9.5E-01
EPDR1_HUMAN	Mammalian ependymin-related protein 1 OS=Homo sapiens GN=EPDR1 PE=1 SV=2	0.83	1	-1.72	1.8E-01	-1.25	4.9E-01	-2.15	9.4E-03
ES1_HUMAN	ES1 protein homolog, mitochondrial OS=Homo sapiens GN=C21orf33 PE=1 SV=3	1	15	-1.09	8.7E-01	-1.27	1.6E-01	-1.38	2.6E-02
ETFA_HUMAN	Electron transfer flavoprotein subunit alpha, mitochondrial OS=Homo sapiens GN=ETFA PE=1 SV=1	1	20	-1.01	9.7E-01	-1.22	4.4E-01	-1.23	2.5E-01
F168B_HUMAN	UPF0541 protein FAM168B OS=Homo sapiens GN=FAM168B PE=2 SV=1	0	1	-1.01	9.7E-01	1.96	1.3E-02	1.94	1.2E-02
FA9_HUMAN	Coagulation factor IX OS=Homo sapiens GN=F9 PE=1 SV=2	1	8	-1.53	3.4E-01	-1.34	4.4E-01	-2.05	6.2E-04
FABP4_HUMAN	Fatty acid-binding protein, adipocyte OS=Homo sapiens GN=FABP4 PE=1 SV=3	0.62	1	1.04	9.6E-01	-1.02	9.6E-01	1.02	9.4E-01
FABP5_HUMAN	Fatty acid-binding protein, epidermal OS=Homo sapiens GN=FABP5 PE=1 SV=3	1	6	1.10	8.7E-01	-1.22	3.3E-01	-1.11	6.7E-01
FABPH_HUMAN	Fatty acid-binding protein, heart OS=Homo sapiens GN=FABP3 PE=1 SV=4	1	19	-1.02	9.7E-01	-1.22	2.3E-02	-1.24	1.2E-01
FB5L3_HUMAN	Putative fatty acid-binding protein 5-like protein 3 OS=Homo sapiens GN=FABP5L3 PE=3 SV=1	0.69	1	-1.08	8.8E-01	-1.01	9.8E-01	-1.10	6.7E-01
FBLN1_HUMAN	Fibulin-1 OS=Homo sapiens GN=FBLN1 PE=1 SV=4	0.99	2	1.13	8.9E-01	1.90	2.8E-02	2.16	4.7E-02
FBLN2_HUMAN	Fibulin-2 OS=Homo sapiens GN=FBLN2 PE=1 SV=2	1	4	1.71	5.8E-01	1.48	2.0E-01	2.52	7.1E-03
FBLN3_HUMAN	EGF-containing fibulin-like extracellular matrix protein 1 OS=Homo sapiens GN=EFEMP1 PE=1 SV=2	0.95	2	1.71	5.8E-01	1.31	3.1E-01	2.25	1.7E-02
FBN1_HUMAN	Fibrillin-1 OS=Homo sapiens GN=FBN1 PE=1 SV=2	1	48	1.79	3.0E-02	-1.26	2.0E-01	1.42	1.3E-02
FETUA_HUMAN	Alpha-2-HS-glycoprotein OS=Homo sapiens GN=AHSG PE=1 SV=1	1	14	1.03	9.4E-01	1.66	1.1E-11	1.71	1.3E-06
FHL1_HUMAN	Four and a half LIM domains protein 1 OS=Homo sapiens GN=FHL1 PE=1 SV=4	1	22	1.12	7.8E-01	-1.06	8.4E-01	1.05	8.6E-01
FHL2_HUMAN	Four and a half LIM domains protein 2 OS=Homo sapiens GN=FHL2 PE=1 SV=3	1	40	-1.02	9.9E-01	-1.19	4.9E-01	-1.22	4.5E-01
FIBA_HUMAN	Fibrinogen alpha chain OS=Homo sapiens GN=FGA PE=1 SV=2	1	9	1.16	8.6E-01	1.56	1.6E-01	1.81	7.1E-02
FIBB_HUMAN	Fibrinogen beta chain OS=Homo sapiens GN=FGB PE=1 SV=2	1	15	1.19	8.1E-01	1.65	8.7E-02	1.96	2.2E-02
FIBG_HUMAN	Fibrinogen gamma chain OS=Homo sapiens GN=FGG PE=1 SV=3	1	13	1.22	7.8E-01	1.89	5.3E-02	2.29	1.4E-02
FKBP3_HUMAN	FK506-binding protein 3 OS=Homo sapiens GN=FKBP3 PE=1 SV=1	0.99	2	-1.26	5.0E-01	-1.25	3.8E-02	-1.57	2.9E-04
FMOD_HUMAN	Fibromodulin OS=Homo sapiens GN=FMOD PE=1 SV=2	0.97	1	1.99	6.8E-01	1.50	5.8E-01	2.98	1.0E-01
FRAS1_HUMAN	Extracellular matrix protein FRAS1 OS=Homo sapiens GN=FRAS1 PE=2 SV=1	0.72	1	-1.10	9.4E-01	-1.22	4.9E-01	-1.33	4.6E-01
FRIH_HUMAN	Ferritin heavy chain OS=Homo sapiens GN=FTH1 PE=1 SV=2	1	8	1.51	2.0E-01	-1.78	6.7E-03	-1.18	4.6E-01
FSTL4_HUMAN	Follistatin-related protein 4 OS=Homo sapiens GN=FSTL4 PE=2 SV=2	0.36	1	1.23	8.1E-01	-1.56	2.1E-01	-1.26	5.5E-01
FUMH_HUMAN	Fumarate hydratase, mitochondrial OS=Homo sapiens GN=FH PE=1 SV=3	0.32	1	1.16	8.7E-01	-1.31	6.2E-01	-1.14	8.7E-01
G3P_HUMAN	Glyceraldehyde-3-phosphate dehydrogenase OS=Homo sapiens GN=GAPDH PE=1 SV=3	1	57	-1.02	9.8E-01	-1.06	7.3E-01	-1.08	6.1E-01
GCSH_HUMAN	Glycine cleavage system H protein, mitochondrial OS=Homo sapiens GN=GCSH PE=1 SV=1	0.89	1	-1.37	5.0E-01	-1.27	9.3E-02	-1.73	7.4E-03
GDIR1_HUMAN	Rho GDP-dissociation inhibitor 1 OS=Homo sapiens GN=ARHGDIA PE=1 SV=3	1	3	-1.07	7.4E-01	-1.09	4.3E-01	-1.16	4.6E-02
GDIR2_HUMAN	Rho GDP-dissociation inhibitor 2 OS=Homo sapiens GN=ARHGDIB PE=1 SV=3	0	1	-1.02	9.7E-01	1.15	4.9E-01	1.13	6.2E-01
GGT2_HUMAN	Gamma-glutamyltranspeptidase 2 OS=Homo sapiens GN=GGT2 PE=1 SV=3	0.98	3	-1.11	8.4E-01	-1.06	8.4E-01	-1.18	4.9E-02
GLYG_HUMAN	Glycogenin-1 OS=Homo sapiens GN=GYG1 PE=1 SV=4	1	13	-1.28	4.7E-01	-1.03	9.0E-01	-1.32	2.8E-02
GPC1_HUMAN	Glypican-1 OS=Homo sapiens GN=GPC1 PE=1 SV=1	0.99	2	-1.36	6.8E-01	-1.04	9.2E-01	-1.42	2.2E-01
GPNMB_HUMAN	Transmembrane glycoprotein NMB OS=Homo sapiens GN=GPNMB PE=1 SV=2	1	8	-1.18	6.8E-01	-1.24	4.1E-01	-1.46	2.1E-02
GRB1L_HUMAN	GREB1-like protein OS=Homo sapiens GN=KIAA1772 PE=2 SV=2	0.49	1	-1.24	6.8E-01	-1.38	2.7E-01	-1.71	1.3E-03
GRP75_HUMAN	Stress-70 protein, mitochondrial OS=Homo sapiens GN=HSPA9 PE=1 SV=2	1	14	1.26	6.8E-01	-1.31	4.3E-01	-1.04	8.1E-01
GRP78_HUMAN	78 kDa glucose-regulated protein OS=Homo sapiens GN=HSPA5 PE=1 SV=2	1	6	1.10	8.9E-01	-1.13	6.6E-01	-1.03	9.4E-01
GSTO1_HUMAN	Glutathione S-transferase omega-1 OS=Homo sapiens GN=GSTO1 PE=1 SV=2	0.44	1	1.16	9.1E-01	1.19	7.6E-01	1.38	4.2E-01
H10_HUMAN	Histone H1.0 OS=Homo sapiens GN=H1F0 PE=1 SV=3	0.95	2	-1.19	6.8E-01	-1.12	6.8E-01	-1.33	8.0E-02
H11_HUMAN	Histone H1.1 OS=Homo sapiens GN=HIST1H1A PE=1 SV=3	0.58	1	1.52	4.4E-01	-1.39	3.7E-01	1.09	7.5E-01
H12_HUMAN	Histone H1.2 OS=Homo sapiens GN=HIST1H1C PE=1 SV=2	1	21	1.03	9.5E-01	-1.10	5.4E-01	-1.07	5.3E-01
H1T_HUMAN	Histone H1t OS=Homo sapiens GN=HIST1H1T PE=1 SV=4	0.98	3	1.10	8.3E-01	1.44	2.6E-02	1.59	8.1E-06
H2A1B_HUMAN	Histone H2A type 1-B/E OS=Homo sapiens GN=HIST1H2AB PE=1 SV=2	0.74	1	-1.13	9.4E-01	1.58	2.0E-01	1.40	4.1E-01
H2A2A_HUMAN	Histone H2A type 2-A OS=Homo sapiens GN=HIST2H2AA3 PE=1 SV=3	1	16	1.22	5.3E-01	-1.11	6.1E-01	1.09	5.9E-01
H2AY_HUMAN	Core histone macro-H2A.1 OS=Homo sapiens GN=H2AFY PE=1 SV=4	1	3	1.12	7.8E-01	-1.17	3.4E-01	-1.05	5.5E-01
H2AZ_HUMAN	Histone H2A.Z OS=Homo sapiens GN=H2AFZ PE=1 SV=2	0.83	1	1.36	8.1E-01	1.17	9.6E-01	1.59	4.9E-01
H2B1M_HUMAN	Histone H2B type 1-C/E/F/G/I OS=Homo sapiens GN=HIST1H2BC PE=1 SV=3	1	14	1.13	6.8E-01	-1.12	4.1E-01	1.01	9.5E-01
H2B2C_HUMAN	Putative histone H2B type 2-D OS=Homo sapiens GN=HIST2H2BD PE=5 SV=3	1	4	1.05	8.7E-01	1.20	4.5E-02	1.26	1.2E-02
H2B3B_HUMAN	Histone H2B type 3-B OS=Homo sapiens GN=HIST3H2BB PE=1 SV=3	0.97	2	-1.04	9.7E-01	-1.24	5.3E-01	-1.29	4.1E-01
H31_HUMAN	Histone H3.1 OS=Homo sapiens GN=HIST1H3A PE=1 SV=2	1	1	1.21	7.9E-01	1.01	9.0E-01	1.22	3.4E-01
H31T_HUMAN	Histone H3.1t OS=Homo sapiens GN=HIST3H3 PE=1 SV=3	0.62	1	-1.14	8.9E-01	-1.08	8.0E-01	-1.23	5.0E-01
H32_HUMAN	Histone H3.2 OS=Homo sapiens GN=HIST2H3A PE=1 SV=3	1	1	1.06	9.4E-01	1.20	7.1E-01	1.27	5.5E-01
H33_HUMAN	Histone H3.3 OS=Homo sapiens GN=H3F3A PE=1 SV=2	1	9	1.09	4.2E-01	-1.10	4.9E-01	-1.00	9.7E-01
H3L_HUMAN	Histone H3-like OS=Homo sapiens PE=2 SV=3	0.67	1	-1.57	7.1E-01	-1.25	4.1E-01	-1.97	6.1E-02
H4_HUMAN	Histone H4 OS=Homo sapiens GN=HIST1H4A PE=1 SV=2	1	10	1.30	6.8E-01	1.05	8.4E-01	1.37	1.5E-02
HBA_HUMAN	Hemoglobin subunit alpha OS=Homo sapiens GN=HBA1 PE=1 SV=2	1	12	-1.19	8.7E-01	3.70	2.6E-02	3.10	2.6E-04
HBB_HUMAN	Hemoglobin subunit beta OS=Homo sapiens GN=HBB PE=1 SV=2	1	12	-1.38	6.8E-01	3.18	1.0E-02	2.31	1.2E-04
HBD_HUMAN	Hemoglobin subunit delta OS=Homo sapiens GN=HBD PE=1 SV=2	0.92	1	1.08	9.4E-01	-1.25	4.2E-01	-1.16	6.3E-01
HBE_HUMAN	Hemoglobin subunit epsilon OS=Homo sapiens GN=HBE1 PE=1 SV=2	0.72	1	1.16	6.9E-01	1.03	8.9E-01	1.19	4.1E-01
HBG1_HUMAN	Hemoglobin subunit gamma-1 OS=Homo sapiens GN=HBG1 PE=1 SV=2	0.93	2	1.01	9.8E-01	1.84	9.7E-02	1.85	4.6E-02
HCDH_HUMAN	Hydroxyacyl-coenzyme A dehydrogenase, mitochondrial OS=Homo sapiens GN=HADH PE=1 SV=2	1	4	1.56	6.5E-01	-1.23	6.7E-01	1.27	3.6E-01
HEBP2_HUMAN	Heme-binding protein 2 OS=Homo sapiens GN=HEBP2 PE=1 SV=1	0.38	1	1.12	8.7E-01	-1.09	7.5E-01	1.03	8.5E-01
HEMO_HUMAN	Hemopexin OS=Homo sapiens GN=HPX PE=1 SV=2	1	30	-1.26	2.6E-01	1.51	6.2E-02	1.20	5.0E-01
HMGB2_HUMAN	High mobility group protein B2 OS=Homo sapiens GN=HMGB2 PE=1 SV=2	0.98	2	-1.17	6.8E-01	-1.11	7.1E-01	-1.29	1.1E-01
HNRPC_HUMAN	Heterogeneous nuclear ribonucleoproteins C1/C2 OS=Homo sapiens GN=HNRNPC PE=1 SV=4	1	3	-1.15	6.8E-01	-1.06	8.0E-01	-1.22	1.6E-01
HNRPD_HUMAN	Heterogeneous nuclear ribonucleoprotein D0 OS=Homo sapiens GN=HNRNPD PE=1 SV=1	1	5	1.07	6.8E-01	-1.16	4.5E-02	-1.09	4.6E-02
HP1B3_HUMAN	Heterochromatin protein 1-binding protein 3 OS=Homo sapiens GN=HP1BP3 PE=1 SV=1	1	4	-1.03	9.5E-01	-1.18	2.8E-01	-1.21	1.3E-01
HPT_HUMAN	Haptoglobin OS=Homo sapiens GN=HP PE=1 SV=1	1	43	-1.21	8.9E-01	1.72	1.4E-01	1.42	4.1E-01
HRG_HUMAN	Histidine-rich glycoprotein OS=Homo sapiens GN=HRG PE=1 SV=1	0.99	3	-1.06	9.1E-01	1.65	1.3E-02	1.56	2.2E-02
HS90A_HUMAN	Heat shock protein HSP 90-alpha OS=Homo sapiens GN=HSP90AA1 PE=1 SV=5	1	16	1.05	9.5E-01	-1.07	8.0E-01	-1.02	8.7E-01
HS90B_HUMAN	Heat shock protein HSP 90-beta OS=Homo sapiens GN=HSP90AB1 PE=1 SV=4	1	3	1.06	9.3E-01	-1.15	6.6E-01	-1.08	7.7E-01
HSP76_HUMAN	Heat shock 70 kDa protein 6 OS=Homo sapiens GN=HSPA6 PE=1 SV=2	0	1	1.07	9.4E-01	-1.26	4.7E-01	-1.18	6.0E-01
HSPB1_HUMAN	Heat shock protein beta-1 OS=Homo sapiens GN=HSPB1 PE=1 SV=2	1	24	-1.10	6.8E-01	1.06	5.1E-01	-1.04	7.4E-01
HSPB2_HUMAN	Heat shock protein beta-2 OS=Homo sapiens GN=HSPB2 PE=1 SV=2	0.94	1	-1.06	9.6E-01	-1.14	6.7E-01	-1.20	5.5E-01
HSPB7_HUMAN	Heat shock protein beta-7 OS=Homo sapiens GN=HSPB7 PE=1 SV=1	1	13	-1.15	3.4E-01	-1.15	4.2E-01	-1.33	1.1E-02
IC1_HUMAN	Plasma protease C1 inhibitor OS=Homo sapiens GN=SERPING1 PE=1 SV=2	1	10	-1.22	7.4E-01	1.45	1.4E-01	1.19	4.3E-01
ICAL_HUMAN	Calpastatin OS=Homo sapiens GN=CAST PE=1 SV=4	1	20	-1.40	3.4E-01	1.06	8.1E-01	-1.31	7.6E-02
IDHP_HUMAN	Isocitrate dehydrogenase [NADP], mitochondrial OS=Homo sapiens GN=IDH2 PE=1 SV=2	1	35	-1.07	8.9E-01	-1.18	5.7E-01	-1.26	2.5E-01
IF4H_HUMAN	Eukaryotic translation initiation factor 4H OS=Homo sapiens GN=EIF4H PE=1 SV=5	0.94	1	1.00	9.9E-01	-1.14	4.4E-01	-1.14	3.5E-01
IF5A1_HUMAN	Eukaryotic translation initiation factor 5A-1 OS=Homo sapiens GN=EIF5A PE=1 SV=2	1	9	-1.09	7.6E-01	-1.25	1.3E-01	-1.35	2.9E-04
IGHA1_HUMAN	Ig alpha-1 chain C region OS=Homo sapiens GN=IGHA1 PE=1 SV=2	1	17	-1.08	9.1E-01	3.61	1.3E-02	3.35	8.0E-05
IGHA2_HUMAN	Ig alpha-2 chain C region OS=Homo sapiens GN=IGHA2 PE=1 SV=3	1	4	-1.64	7.4E-01	7.94	7.5E-06	4.85	2.6E-04
IGHG1_HUMAN	Ig gamma-1 chain C region OS=Homo sapiens GN=IGHG1 PE=1 SV=1	1	16	1.21	6.8E-01	1.46	3.3E-01	1.77	5.8E-02
IGHG2_HUMAN	Ig gamma-2 chain C region OS=Homo sapiens GN=IGHG2 PE=1 SV=2	1	28	1.24	6.2E-01	1.62	9.4E-02	2.01	1.1E-02
IGHG3_HUMAN	Ig gamma-3 chain C region OS=Homo sapiens GN=IGHG3 PE=1 SV=2	1	4	-1.19	6.8E-01	-1.24	3.8E-02	-1.48	4.5E-03
IGHG4_HUMAN	Ig gamma-4 chain C region OS=Homo sapiens GN=IGHG4 PE=1 SV=1	1	6	1.61	3.9E-01	1.15	2.3E-01	1.84	1.9E-02
IGHM_HUMAN	Ig mu chain C region OS=Homo sapiens GN=IGHM PE=1 SV=3	0.99	2	-1.08	9.6E-01	4.98	3.1E-03	4.62	1.2E-03
IGKC_HUMAN	Ig kappa chain C region OS=Homo sapiens GN=IGKC PE=1 SV=1	1	19	1.05	9.4E-01	1.83	1.4E-01	1.91	6.5E-02
IPYR2_HUMAN	Inorganic pyrophosphatase 2, mitochondrial OS=Homo sapiens GN=PPA2 PE=1 SV=2	0.74	2	-1.26	7.1E-01	-1.16	6.4E-01	-1.47	1.7E-01
ITB1_HUMAN	Integrin beta-1 OS=Homo sapiens GN=ITGB1 PE=1 SV=2	1	18	-1.03	9.4E-01	-1.18	5.9E-01	-1.22	6.4E-02
ITIH1_HUMAN	Inter-alpha-trypsin inhibitor heavy chain H1 OS=Homo sapiens GN=ITIH1 PE=1 SV=3	0.81	1	-1.64	2.3E-02	1.22	5.4E-01	-1.35	3.3E-01
ITPR3_HUMAN	Inositol 1,4,5-trisphosphate receptor type 3 OS=Homo sapiens GN=ITPR3 PE=1 SV=2	0	1	1.09	7.8E-01	1.02	8.4E-01	1.11	4.7E-01
K0406_HUMAN	Uncharacterized protein KIAA0406 OS=Homo sapiens GN=KIAA0406 PE=1 SV=3	0.6	1	-1.34	6.8E-01	1.78	8.3E-02	1.32	5.1E-01
KAD1_HUMAN	Adenylate kinase isoenzyme 1 OS=Homo sapiens GN=AK1 PE=1 SV=3	1	19	1.12	2.1E-01	-1.26	3.6E-02	-1.12	2.4E-01

KCRB_HUMAN	Creatine kinase B-type OS=Homo sapiens GN=CKB PE=1 SV=1	1	17	-1.13	8.1E-01	-1.31	3.4E-01	-1.47	2.2E-02
KCRM_HUMAN	Creatine kinase M-type OS=Homo sapiens GN=CKM PE=1 SV=2	1	41	-1.23	6.8E-01	-1.41	2.8E-01	-1.73	4.5E-03
KCRS_HUMAN	Creatine kinase S-type, mitochondrial OS=Homo sapiens GN=CKMT2 PE=1 SV=2	1	22	1.03	9.7E-01	-1.28	4.8E-01	-1.24	2.6E-01
KCY_HUMAN	UMP-CMP kinase OS=Homo sapiens GN=CMPK1 PE=1 SV=3	0.99	3	1.04	9.3E-01	-1.18	2.6E-01	-1.14	3.5E-01
KLOTB_HUMAN	Beta-klotho OS=Homo sapiens GN=KLB PE=2 SV=1	0	1	1.11	8.9E-01	1.18	6.4E-01	1.30	2.8E-01
KNG1_HUMAN	Kininogen-1 OS=Homo sapiens GN=KNG1 PE=1 SV=2	1	10	-1.16	7.3E-01	1.67	4.2E-03	1.44	1.1E-01
LAC_HUMAN	Ig lambda chain C regions OS=Homo sapiens GN=IGLC1 PE=1 SV=1	1	9	1.18	8.1E-01	1.94	3.6E-02	2.29	3.4E-04
LAMA2_HUMAN	Laminin subunit alpha-2 OS=Homo sapiens GN=LAMA2 PE=1 SV=4	1	77	-1.27	6.8E-01	-1.18	3.3E-01	-1.50	2.1E-02
LAMA4_HUMAN	Laminin subunit alpha-4 OS=Homo sapiens GN=LAMA4 PE=1 SV=3	1	11	-1.07	9.2E-01	-1.02	8.4E-01	-1.10	6.1E-01
LAMB1_HUMAN	Laminin subunit beta-1 OS=Homo sapiens GN=LAMB1 PE=1 SV=1	1	28	-1.28	6.8E-01	-1.21	4.1E-01	-1.55	1.2E-02
LAMB2_HUMAN	Laminin subunit beta-2 OS=Homo sapiens GN=LAMB2 PE=1 SV=2	1	39	-1.03	9.6E-01	-1.19	1.3E-02	-1.23	1.1E-01
LAMC1_HUMAN	Laminin subunit gamma-1 OS=Homo sapiens GN=LAMC1 PE=1 SV=3	1	57	-1.14	7.3E-01	-1.17	6.2E-02	-1.33	2.5E-02
LAMP1_HUMAN	Lysosome-associated membrane glycoprotein 1 OS=Homo sapiens GN=LAMP1 PE=1 SV=3	0.78	1	-1.40	2.6E-01	1.06	8.3E-01	-1.32	2.5E-01
LCORL_HUMAN	Ligand-dependent nuclear receptor corepressor-like protein OS=Homo sapiens GN=LCORL PE=2 SV=4	0.28	1	-1.09	9.4E-01	-1.09	7.3E-01	-1.19	4.4E-01
LDB3_HUMAN	LIM domain-binding protein 3 OS=Homo sapiens GN=LDB3 PE=1 SV=2	1	4	-1.03	9.4E-01	-1.18	7.8E-01	-1.21	3.2E-01
LDHB_HUMAN	L-lactate dehydrogenase B chain OS=Homo sapiens GN=LDHB PE=1 SV=2	1	19	-1.08	8.9E-01	-1.14	6.9E-01	-1.24	2.3E-01
LEG1_HUMAN	Galectin-1 OS=Homo sapiens GN=LGALS1 PE=1 SV=2	1	17	-1.09	6.9E-01	-1.04	8.2E-01	-1.13	4.4E-01
LG3BP_HUMAN	Galectin-3-binding protein OS=Homo sapiens GN=LGALS3BP PE=1 SV=1	1	17	-1.47	4.7E-02	-1.14	4.1E-01	-1.67	1.3E-03
LTBP2_HUMAN	Latent-transforming growth factor beta-binding protein 2 OS=Homo sapiens GN=LTBP2 PE=1 SV=2	1	6	1.44	8.7E-01	2.85	1.2E-01	4.12	9.8E-03
LU_HUMAN	Lutheran blood group glycoprotein precursor - Homo sapiens	1	7	-1.07	8.7E-01	-1.17	7.4E-02	-1.26	4.6E-02
LUM_HUMAN	Lumican OS=Homo sapiens GN=LUM PE=1 SV=2	1	31	1.11	9.4E-01	1.47	3.3E-01	1.64	1.2E-01
LV202_HUMAN	Ig lambda chain V-II region NEI OS=Homo sapiens PE=1 SV=1	0.68	1	1.17	8.9E-01	-1.68	5.2E-02	-1.43	1.9E-01
LYSC_HUMAN	Lysozyme C OS=Homo sapiens GN=LYZ PE=1 SV=1	0	1	-1.10	9.5E-01	-1.23	7.3E-01	-1.35	6.0E-01
M3K5_HUMAN	Mitogen-activated protein kinase kinase kinase 5 OS=Homo sapiens GN=MAP3K5 PE=1 SV=1	0	1	-1.02	9.9E-01	1.68	5.4E-01	1.64	3.5E-01
M6PBP_HUMAN	Mannose-6-phosphate receptor-binding protein 1 OS=Homo sapiens GN=M6PRBP1 PE=1 SV=2	0.46	1	-1.09	9.4E-01	-1.21	4.5E-01	-1.32	4.0E-01
MAOM_HUMAN	NAD-dependent malic enzyme, mitochondrial OS=Homo sapiens GN=ME2 PE=1 SV=1	0	1	1.01	9.5E-01	-1.31	4.8E-01	-1.29	4.9E-01
MARCS_HUMAN	Myristoylated alanine-rich C-kinase substrate OS=Homo sapiens GN=MARCKS PE=1 SV=4	1	3	-1.16	8.7E-01	1.06	8.3E-01	-1.10	6.9E-01
MDHC_HUMAN	Malate dehydrogenase, cytoplasmic OS=Homo sapiens GN=MDH1 PE=1 SV=4	1	14	1.11	9.4E-01	-1.20	6.5E-01	-1.08	6.1E-01
MDHM_HUMAN	Malate dehydrogenase, mitochondrial OS=Homo sapiens GN=MDH2 PE=1 SV=3	1	20	1.13	9.2E-01	-1.16	7.3E-01	-1.03	8.8E-01
MFAP4_HUMAN	Microfibril-associated glycoprotein 4 OS=Homo sapiens GN=MFAP4 PE=1 SV=2	1	5	1.54	6.8E-01	1.63	1.3E-01	2.50	1.4E-02
MFAP5_HUMAN	Microfibrillar-associated protein 5 OS=Homo sapiens GN=MFAP5 PE=2 SV=1	1	4	2.01	1.5E-01	-1.39	2.8E-01	1.45	6.9E-02
MFGM_HUMAN	Lactadherin OS=Homo sapiens GN=MFGE8 PE=1 SV=2	1	14	-1.06	8.7E-01	-1.00	1.0E+00	-1.06	6.3E-01
MGST3_HUMAN	Microsomal glutathione S-transferase 3 OS=Homo sapiens GN=MGST3 PE=1 SV=1	0.83	1	1.93	4.3E-01	-1.54	2.1E-01	1.25	6.7E-01
MIME_HUMAN	Mimecan OS=Homo sapiens GN=OGN PE=1 SV=1	1	26	1.08	9.7E-01	1.47	3.7E-01	1.58	1.4E-01
MLE3_HUMAN	Myosin light chain 3, skeletal muscle isoform OS=Homo sapiens GN=MYL1 PE=2 SV=2	1	5	-1.14	4.7E-01	-1.05	7.3E-01	-1.20	4.2E-02
MLL2_HUMAN	Histone-lysine N-methyltransferase MLL2 OS=Homo sapiens GN=MLL2 PE=1 SV=1	0	1	1.42	6.8E-01	-1.28	5.3E-01	1.11	6.2E-01
MLRS_HUMAN	Myosin regulatory light chain 2, skeletal muscle isoform OS=Homo sapiens GN=MYLPF PE=2 SV=1	0.5	1	-1.04	8.7E-01	-1.00	9.2E-01	-1.05	6.4E-01
MLRV_HUMAN	Myosin regulatory light chain 2, ventricular/cardiac muscle isoform OS=Homo sapiens GN=MYL2 PE=1 SV=3	1	43	1.11	7.4E-01	-1.29	2.8E-03	-1.15	2.5E-01
MPCP_HUMAN	Phosphate carrier protein, mitochondrial OS=Homo sapiens GN=SLC25A3 PE=1 SV=2	1	5	1.04	9.6E-01	-1.35	3.9E-01	-1.30	2.4E-01
MRLC2_HUMAN	Myosin regulatory light chain MRLC2 OS=Homo sapiens GN=MYLC2B PE=1 SV=2	0.93	2	1.01	9.9E-01	1.24	3.1E-01	1.26	1.1E-01
MRLC3_HUMAN	Myosin regulatory light chain MRLC3 OS=Homo sapiens GN=MRLC3 PE=1 SV=2	0.66	1	-2.06	4.4E-01	1.67	3.7E-01	-1.23	7.3E-01
MSRB2_HUMAN	Methionine-R-sulfoxide reductase B2, mitochondrial OS=Homo sapiens GN=MSRB2 PE=2 SV=2	0	1	-1.22	6.8E-01	1.02	9.0E-01	-1.20	4.3E-01
MUC18_HUMAN	Cell surface glycoprotein MUC18 OS=Homo sapiens GN=MCAM PE=1 SV=2	1	2	-1.30	3.2E-01	-1.05	8.7E-01	-1.36	6.9E-02
MYG_HUMAN	Myoglobin OS=Homo sapiens GN=MB PE=1 SV=2	1	27	1.19	3.0E-01	-1.19	2.9E-01	-1.00	9.6E-01
MYH13_HUMAN	Myosin-13 OS=Homo sapiens GN=MYH13 PE=1 SV=1	0.59	1	-1.27	8.2E-01	1.41	4.8E-01	1.11	9.6E-01
MYH2_HUMAN	Myosin-2 OS=Homo sapiens GN=MYH2 PE=1 SV=1	0.95	2	-1.21	8.7E-01	-1.71	1.3E-01	-2.08	4.5E-03
MYH3_HUMAN	Myosin-3 OS=Homo sapiens GN=MYH3 PE=1 SV=3	0.41	1	-1.17	9.4E-01	-1.60	4.2E-01	-1.88	1.7E-01
MYH7_HUMAN	Myosin-7 OS=Homo sapiens GN=MYH7 PE=1 SV=5	1	66	1.18	3.0E-01	-1.35	2.5E-02	-1.14	2.2E-01
MYH8_HUMAN	Myosin-8 OS=Homo sapiens GN=MYH8 PE=1 SV=3	0.95	1	1.24	7.5E-01	-1.30	4.4E-01	-1.06	8.1E-01
MYL3_HUMAN	Myosin light chain 3 OS=Homo sapiens GN=MYL3 PE=1 SV=3	1	56	1.12	4.7E-01	-1.25	1.9E-03	-1.12	1.4E-01
MYL4_HUMAN	Myosin light chain 4 OS=Homo sapiens GN=MYL4 PE=1 SV=3	1	8	1.05	8.7E-01	1.59	2.5E-03	1.68	5.1E-03
MYL6_HUMAN	Myosin light polypeptide 6 OS=Homo sapiens GN=MYL6 PE=1 SV=2	1	5	1.32	6.8E-01	-1.03	9.6E-01	1.28	2.4E-01
MYL9_HUMAN	Myosin regulatory light polypeptide 9 OS=Homo sapiens GN=MYL9 PE=1 SV=4	1	12	1.05	9.4E-01	-1.02	9.1E-01	1.02	8.7E-01
MYLPL_HUMAN	Myosin light chain 2, lymphocyte-specific OS=Homo sapiens GN=MYLC2PL PE=2 SV=2	1	14	1.08	8.7E-01	-1.20	2.4E-01	-1.11	5.2E-01
MYO6_HUMAN	Myosin-VI OS=Homo sapiens GN=MYO6 PE=1 SV=4	0	1	1.29	6.8E-01	1.31	4.8E-01	1.69	7.2E-02
MYOZ2_HUMAN	Myozenin-2 OS=Homo sapiens GN=MYOZ2 PE=1 SV=1	1	38	1.02	9.6E-01	-1.16	4.9E-01	-1.13	4.4E-01
MYP2_HUMAN	Myelin P2 protein OS=Homo sapiens GN=PMP2 PE=1 SV=3	0.89	2	1.27	6.8E-01	-1.03	8.6E-01	1.23	4.9E-01
MYPT1_HUMAN	Protein phosphatase 1 regulatory subunit 12A OS=Homo sapiens GN=PPP1R12A PE=1 SV=1	0	1	-1.28	5.3E-01	1.03	8.3E-01	-1.24	2.2E-01
MYPT2_HUMAN	Protein phosphatase 1 regulatory subunit 12B OS=Homo sapiens GN=PPP1R12B PE=1 SV=2	1	4	-1.13	8.8E-01	-1.23	2.6E-01	-1.39	1.8E-01
NCAM1_HUMAN	Neural cell adhesion molecule 1 OS=Homo sapiens GN=NCAM1 PE=1 SV=3	1	3	-1.22	6.8E-01	-1.20	7.1E-01	-1.46	5.9E-02
NDKB_HUMAN	Nucleoside diphosphate kinase B OS=Homo sapiens GN=NME2 PE=1 SV=1	0.82	1	-1.04	9.5E-01	-1.18	4.1E-01	-1.23	1.7E-01
NDUA4_HUMAN	NADH dehydrogenase [ubiquinone] 1 alpha subcomplex subunit 4 OS=Homo sapiens GN=NDUFA4 PE=1 SV=1	1	2	-1.06	9.5E-01	-1.50	1.5E-01	-1.59	4.9E-02
NDUA7_HUMAN	NADH dehydrogenase [ubiquinone] 1 alpha subcomplex subunit 7 OS=Homo sapiens GN=NDUFA7 PE=1 SV=3	0.83	1	1.17	7.1E-01	-1.49	4.6E-02	-1.27	2.0E-01
NDUA8_HUMAN	NADH dehydrogenase [ubiquinone] 1 alpha subcomplex subunit 8 OS=Homo sapiens GN=NDUFA8 PE=1 SV=3	1	4	-1.03	9.8E-01	-1.38	4.7E-02	-1.42	1.4E-01
NDUAA_HUMAN	NADH dehydrogenase [ubiquinone] 1 alpha subcomplex subunit 10, mitochondrial OS=Homo sapiens GN=NDUFA10 PE=1 SV=1	1	3	1.03	9.7E-01	-1.22	6.6E-01	-1.18	5.5E-01
NDUAC_HUMAN	NADH dehydrogenase [ubiquinone] 1 alpha subcomplex subunit 12 OS=Homo sapiens GN=NDUFA12 PE=1 SV=1	0.99	2	-1.06	9.5E-01	-1.53	8.4E-03	-1.61	2.2E-02
NDUAD_HUMAN	NADH dehydrogenase [ubiquinone] 1 alpha subcomplex subunit 13 OS=Homo sapiens GN=NDUFA13 PE=1 SV=3	0.75	1	1.42	7.8E-01	-1.23	7.3E-01	1.16	7.1E-01
NDUB3_HUMAN	NADH dehydrogenase [ubiquinone] 1 beta subcomplex subunit 3 OS=Homo sapiens GN=NDUFB3 PE=1 SV=3	0.83	1	-1.11	8.2E-01	-1.21	1.2E-01	-1.34	1.1E-01
NDUB9_HUMAN	NADH dehydrogenase [ubiquinone] 1 beta subcomplex subunit 9 OS=Homo sapiens GN=NDUFB9 PE=1 SV=3	1	3	-1.05	9.7E-01	-1.19	5.2E-01	-1.25	4.3E-01
NDUBA_HUMAN	NADH dehydrogenase [ubiquinone] 1 beta subcomplex subunit 10 OS=Homo sapiens GN=NDUFB10 PE=1 SV=3	1	16	-1.00	9.7E-01	-1.37	3.5E-03	-1.37	1.9E-01
NDUS4_HUMAN	NADH dehydrogenase [ubiquinone] iron-sulfur protein 4, mitochondrial OS=Homo sapiens GN=NDUFS4 PE=1 SV=1	1	16	-1.21	6.8E-01	-1.22	1.3E-01	-1.48	2.3E-02
NDUS5_HUMAN	NADH dehydrogenase [ubiquinone] iron-sulfur protein 5 OS=Homo sapiens GN=NDUFS5 PE=1 SV=3	0.99	2	1.12	8.7E-01	-1.16	4.2E-01	-1.04	9.3E-01
NDUS6_HUMAN	NADH dehydrogenase [ubiquinone] iron-sulfur protein 6, mitochondrial OS=Homo sapiens GN=NDUFS6 PE=1 SV=1	1	15	-1.19	7.4E-01	-1.33	6.4E-02	-1.58	1.2E-02
NDUS7_HUMAN	NADH dehydrogenase [ubiquinone] iron-sulfur protein 7, mitochondrial OS=Homo sapiens GN=NDUFS7 PE=1 SV=3	1	7	-1.07	8.7E-01	-1.18	4.5E-01	-1.27	1.8E-01
NDUS8_HUMAN	NADH dehydrogenase [ubiquinone] iron-sulfur protein 8, mitochondrial OS=Homo sapiens GN=NDUFS8 PE=1 SV=1	0.61	1	1.07	9.4E-01	-1.27	4.2E-01	-1.19	4.7E-01
NDUV1_HUMAN	NADH dehydrogenase [ubiquinone] flavoprotein 1, mitochondrial OS=Homo sapiens GN=NDUFV1 PE=1 SV=4	1	5	-1.06	9.4E-01	-1.22	5.5E-01	-1.30	3.2E-01
NDUV2_HUMAN	NADH dehydrogenase [ubiquinone] flavoprotein 2, mitochondrial OS=Homo sapiens GN=NDUFV2 PE=1 SV=2	0.97	2	1.07	9.6E-01	-1.14	8.0E-01	-1.07	7.4E-01
NDUV3_HUMAN	NADH dehydrogenase [ubiquinone] flavoprotein 3, mitochondrial OS=Homo sapiens GN=NDUFV3 PE=2 SV=2	0.83	1	-1.05	9.5E-01	-1.23	1.3E-01	-1.30	2.5E-01
NEBL_HUMAN	Nebulette OS=Homo sapiens GN=NEBL PE=1 SV=1	1	7	1.30	7.1E-01	-1.23	5.5E-01	1.06	9.4E-01
NEXN_HUMAN	Nexilin OS=Homo sapiens GN=NEXN PE=1 SV=1	0.98	2	1.19	7.9E-01	-1.24	4.3E-01	-1.04	9.4E-01
NID1_HUMAN	Nidogen-1 OS=Homo sapiens GN=NID1 PE=1 SV=3	0.3	1	-1.02	9.5E-01	1.72	5.7E-04	1.69	4.5E-04
NID2_HUMAN	Nidogen-2 OS=Homo sapiens GN=NID2 PE=1 SV=2	1	29	-1.01	9.9E-01	-1.19	3.3E-02	-1.20	2.5E-01
NP1L1_HUMAN	Nucleosome assembly protein 1-like 1 OS=Homo sapiens GN=NAP1L1 PE=1 SV=1	0.43	1	-1.06	9.4E-01	-1.21	4.1E-01	-1.28	1.4E-01
NP1L4_HUMAN	Nucleosome assembly protein 1-like 4 OS=Homo sapiens GN=NAP1L4 PE=1 SV=1	0.98	2	-1.06	8.7E-01	-1.02	9.6E-01	-1.08	3.5E-01
NPM_HUMAN	Nucleophosmin OS=Homo sapiens GN=NPM1 PE=1 SV=2	1	6	-1.07	7.9E-01	-1.13	4.6E-01	-1.21	2.2E-02
NUCL_HUMAN	Nucleolin OS=Homo sapiens GN=NCL PE=1 SV=3	0.99	4	-1.00	9.7E-01	-1.07	8.0E-01	-1.08	5.4E-01
OBFC1_HUMAN	Oligonucleotide/oligosaccharide-binding fold-containing protein 1 OS=Homo sapiens GN=OBFC1 PE=2 SV=2	0	1	-1.13	7.7E-01	2.16	1.5E-05	1.92	2.3E-05
OCAD1_HUMAN	OCIA domain-containing protein 1 OS=Homo sapiens GN=OCIAD1 PE=1 SV=1	1	6	-1.02	9.5E-01	-1.04	8.4E-01	-1.05	6.9E-01
ODPA_HUMAN	Pyruvate dehydrogenase E1 component subunit alpha, somatic form, mitochondrial OS=Homo sapiens GN=PDHA1 PE=1 SV=3	1	11	1.05	9.4E-01	-1.23	4.6E-01	-1.16	5.0E-01
PABP3_HUMAN	Polyadenylate-binding protein 3 OS=Homo sapiens GN=PABPC3 PE=1 SV=2	0	1	1.14	9.2E-01	-1.21	7.1E-01	-1.06	8.9E-01
PAL4B_HUMAN	Peptidylprolyl cis-trans isomerase A-like 4B OS=Homo sapiens GN=PPIAL4B PE=1 SV=1	1	9	-1.10	4.4E-01	-1.18	2.2E-02	-1.31	1.5E-07
PARC_HUMAN	p53-associated parkin-like cytoplasmic protein - Homo sapiens	0	1	-1.07	9.2E-01	-1.08	7.3E-01	-1.16	5.2E-01
PARK7_HUMAN	Protein DJ-1 OS=Homo sapiens GN=PARK7 PE=1 SV=2	1	3	1.16	6.8E-01	1.37	1.7E-02	1.58	2.7E-07
PCDH9_HUMAN	Protocadherin-9 OS=Homo sapiens GN=PCDH9 PE=1 SV=2	0	1	1.07	8.9E-01	-1.14	4.5E-01	-1.06	7.7E-01
PDIA1_HUMAN	Protein disulfide-isomerase OS=Homo sapiens GN=P4HB PE=1 SV=3	0.83	1	-1.07	9.4E-01	1.17	5.0E-01	1.10	7.3E-01
PDIA3_HUMAN	Protein disulfide-isomerase A3 OS=Homo sapiens GN=PDIA3 PE=1 SV=4	1	5	-1.01	9.9E-01	1.08	7.3E-01	1.07	6.9E-01
PDIA6_HUMAN	Protein disulfide-isomerase A6 OS=Homo sapiens GN=PDIA6 PE=1 SV=1	0.81	1	1.02	9.9E-01	-1.01	9.7E-01	1.01	9.4E-01
PDLI1_HUMAN	PDZ and LIM domain protein 1 OS=Homo sapiens GN=PDLIM1 PE=1 SV=4	1	9	1.17	6.8E-01	-1.08	7.1E-01	1.09	5.9E-01
PDLI3_HUMAN	PDZ and LIM domain protein 3 OS=Homo sapiens GN=PDLIM3 PE=2 SV=1	1	4	1.44	2.3E-01	-1.08	7.0E-01	1.33	5.7E-02
PDLI5_HUMAN	PDZ and LIM domain protein 5 OS=Homo sapiens GN=PDLIM5 PE=1 SV=4	1	16	-1.03	9.4E-01	-1.11	4.7E-01	-1.15	1.8E-01
PEBP1_HUMAN	Phosphatidylethanolamine-binding protein 1 OS=Homo sapiens GN=PEBP1 PE=1 SV=3	1	16	-1.08	5.5E-01	-1.16	8.4E-03	-1.25	3.6E-04
PGAM1_HUMAN	Phosphoglycerate mutase 1 OS=Homo sapiens GN=PGAM1 PE=1 SV=2	1	9	-1.09	8.7E-01	1.54	1.6E-03	1.42	6.6E-03
PGAM2_HUMAN	Phosphoglycerate mutase 2 OS=Homo sapiens GN=PGAM2 PE=1 SV=3	1	11	1.15	7.1E-01	-1.51	7.2E-02	-1.32	1.2E-01
PGBM_HUMAN	Basement membrane-specific heparan sulfate proteoglycan core protein OS=Homo sapiens GN=HSPG2 PE=1 SV=3	1	21	1.16	5.0E-01	-1.01	9.2E-01	1.15	2.9E-01
PGK1_HUMAN	Phosphoglycerate kinase 1 OS=Homo sapiens GN=PGK1 PE=1 SV=3	0.99	3	1.20	9.4E-01	1.15	7.3E-01	1.39	3.2E-01
PGRC2_HUMAN	Membrane-associated progesterone receptor component 2 OS=Homo sapiens GN=PGRMC2 PE=1 SV=1	0	1	-1.07	7.5E-01	-1.15	2.1E-01	-1.23	3.0E-02
PGS1_HUMAN	Biglycan OS=Homo sapiens GN=BGN PE=1 SV=2	1	23	1.25	8.7E-01	1.47	4.1E-01	1.83	1.1E-01
PGS2_HUMAN	Decorin OS=Homo sapiens GN=DCN PE=1 SV=1	1	26	1.09	9.4E-01	1.21	5.5E-01	1.32	2.9E-01
PHP14_HUMAN	14 kDa phosphohistidine phosphatase OS=Homo sapiens GN=PHPT1 PE=1 SV=1	0	1	-1.25	2.7E-01	-1.15	2.0E-01	-1.44	1.6E-03
PLCF_HUMAN	1-acyl-sn-glycerol-3-phosphate acyltransferase zeta OS=Homo sapiens GN=AGPAT6 PE=1 SV=1	0	1	1.02	9.9E-01	-1.36	4.2E-01	-1.33	3.3E-01
POPD1_HUMAN	Blood vessel epicardial substance OS=Homo sapiens GN=BVES PE=2 SV=1	0.94	1	-1.20	8.4E-01	-1.17	5.7E-01	-1.40	1.5E-01
PPIA_HUMAN	Peptidyl-prolyl cis-trans isomerase A OS=Homo sapiens GN=PPIA PE=1 SV=2	1	18	-1.06	5.8E-01	-1.16	1.3E-02	-1.23	7.7E-06
PPIF_HUMAN	Peptidyl-prolyl cis-trans isomerase, mitochondrial OS=Homo sapiens GN=PPIF PE=1 SV=1	1	12	-1.49	2.6E-02	-1.22	1.5E-01	-1.82	2.5E-08
PRDX1_HUMAN	Peroxiredoxin-1 OS=Homo sapiens GN=PRDX1 PE=1 SV=1	1	3	1.05	9.4E-01	-1.07	7.5E-01	-1.02	9.1E-01
PRDX2_HUMAN	Peroxiredoxin-2 OS=Homo sapiens GN=PRDX2 PE=1 SV=5	1	9	-1.02	9.6E-01	-1.09	7.3E-01	-1.11	4.3E-01
PRDX3_HUMAN	Thioredoxin-dependent peroxide reductase, mitochondrial OS=Homo sapiens GN=PRDX3 PE=1 SV=3	1	6	1.08	9.1E-01	-1.19	5.0E-01	-1.11	4.4E-01

PRELP_HUMAN	Prolargin OS=Homo sapiens GN=PRELP PE=1 SV=1	1	28	1.31	8.7E-01	1.41	4.7E-01	1.85	1.4E-01
PROF1_HUMAN	Profilin-1 OS=Homo sapiens GN=PFN1 PE=1 SV=2	1	3	1.15	7.3E-01	-1.12	4.8E-01	1.02	8.3E-01
PSD7_HUMAN	26S proteasome non-ATPase regulatory subunit 7 OS=Homo sapiens GN=PSMD7 PE=1 SV=2	0	1	1.03	9.6E-01	-1.27	3.1E-01	-1.24	3.2E-01
PTGDS_HUMAN	Prostaglandin-H2 D-isomerase OS=Homo sapiens GN=PTGDS PE=1 SV=1	1	6	-1.53	2.6E-02	-1.26	6.5E-02	-1.92	6.2E-05
PTN11_HUMAN	Tyrosine-protein phosphatase non-receptor type 11 OS=Homo sapiens GN=PTPN11 PE=1 SV=2	0.38	1	-1.13	6.8E-01	-1.16	1.7E-01	-1.31	1.3E-03
PTRF_HUMAN	Polymerase I and transcript release factor OS=Homo sapiens GN=PTRF PE=1 SV=1	1	19	1.07	7.3E-01	-1.08	4.9E-01	-1.02	9.2E-01
PURA_HUMAN	Transcriptional activator protein Pur-alpha OS=Homo sapiens GN=PURA PE=1 SV=2	0.99	1	-1.01	9.7E-01	-1.13	3.1E-01	-1.15	2.4E-01
QCR6_HUMAN	Cytochrome b-c1 complex subunit 6, mitochondrial OS=Homo sapiens GN=UQCRH PE=1 SV=2	1	11	-1.17	7.9E-01	-1.36	2.8E-03	-1.59	4.1E-02
QCR7_HUMAN	Cytochrome b-c1 complex subunit 7 OS=Homo sapiens GN=UQCRB PE=1 SV=2	0.82	1	1.01	9.6E-01	-1.16	1.4E-01	-1.14	3.2E-01
QIL1_HUMAN	Protein QIL1 OS=Homo sapiens GN=QIL1 PE=1 SV=1	0.97	3	-1.07	9.2E-01	-1.10	5.4E-01	-1.17	4.1E-01
RABE2_HUMAN	Rab GTPase-binding effector protein 2 OS=Homo sapiens GN=RABEP2 PE=1 SV=2	0.38	1	-1.04	9.6E-01	-1.57	2.5E-01	-1.63	2.6E-01
RHG06_HUMAN	Rho GTPase-activating protein 6 OS=Homo sapiens GN=ARHGAP6 PE=1 SV=3	0	1	-1.88	2.1E-01	-1.52	3.8E-02	-2.87	7.7E-06
RL17_HUMAN	60S ribosomal protein L17 OS=Homo sapiens GN=RPL17 PE=1 SV=3	0.28	1	-1.12	8.7E-01	-1.22	3.4E-01	-1.37	1.6E-02
RL18_HUMAN	60S ribosomal protein L18 OS=Homo sapiens GN=RPL18 PE=1 SV=2	0.99	2	1.00	9.6E-01	-1.03	9.6E-01	-1.03	9.1E-01
RL22_HUMAN	60S ribosomal protein L22 OS=Homo sapiens GN=RPL22 PE=1 SV=2	0.98	2	-1.08	8.7E-01	-1.17	4.5E-01	-1.27	1.4E-02
RL23_HUMAN	60S ribosomal protein L23 OS=Homo sapiens GN=RPL23 PE=1 SV=1	1	7	-1.07	7.8E-01	-1.13	3.8E-01	-1.20	3.0E-03
RL23A_HUMAN	60S ribosomal protein L23a OS=Homo sapiens GN=RPL23A PE=1 SV=1	0.83	1	-1.01	9.7E-01	-1.07	7.1E-01	-1.08	4.9E-01
RL24_HUMAN	60S ribosomal protein L24 OS=Homo sapiens GN=RPL24 PE=1 SV=1	1	2	-1.16	6.8E-01	-1.15	4.8E-01	-1.34	1.8E-03
RL27A_HUMAN	60S ribosomal protein L27a OS=Homo sapiens GN=RPL27A PE=1 SV=2	1	2	-1.20	4.5E-01	-1.08	6.2E-01	-1.29	1.9E-04
RL31_HUMAN	60S ribosomal protein L31 OS=Homo sapiens GN=RPL31 PE=1 SV=1	0.99	3	-1.08	8.7E-01	-1.24	2.4E-01	-1.33	3.3E-04
RL35_HUMAN	60S ribosomal protein L35 OS=Homo sapiens GN=RPL35 PE=1 SV=2	0.34	1	-1.23	6.8E-01	-1.22	3.1E-01	-1.50	7.6E-04
RL6_HUMAN	60S ribosomal protein L6 OS=Homo sapiens GN=RPL6 PE=1 SV=3	1	4	-1.18	6.8E-01	-1.16	4.4E-01	-1.37	6.6E-04
RL7_HUMAN	60S ribosomal protein L7 OS=Homo sapiens GN=RPL7 PE=1 SV=1	0.74	1	-1.07	8.9E-01	-1.03	8.7E-01	-1.10	5.9E-01
RLA2_HUMAN	60S acidic ribosomal protein P2 OS=Homo sapiens GN=RPLP2 PE=1 SV=1	1	7	-1.16	6.8E-01	-1.18	2.7E-01	-1.36	1.0E-03
ROA1_HUMAN	Heterogeneous nuclear ribonucleoprotein A1 OS=Homo sapiens GN=HNRNPA1 PE=1 SV=4	0.83	1	1.16	6.8E-01	-1.23	2.1E-01	-1.07	6.7E-01
ROA2_HUMAN	Heterogeneous nuclear ribonucleoproteins A2/B1 OS=Homo sapiens GN=HNRNPA2B1 PE=1 SV=2	1	4	1.11	7.5E-01	-1.02	9.0E-01	1.09	5.9E-01
ROA3_HUMAN	Heterogeneous nuclear ribonucleoprotein A3 OS=Homo sapiens GN=HNRNPA3 PE=1 SV=2	0	1	1.09	7.8E-01	-1.18	3.1E-01	-1.08	5.3E-01
RPE_HUMAN	Ribulose-phosphate 3-epimerase OS=Homo sapiens GN=RPE PE=1 SV=1	0.89	1	1.06	8.7E-01	-1.01	9.2E-01	1.05	5.4E-01
RRBP1_HUMAN	Ribosome-binding protein 1 OS=Homo sapiens GN=RRBP1 PE=1 SV=4	0.82	1	1.29	8.7E-01	-1.25	4.9E-01	1.03	9.6E-01
RS13_HUMAN	40S ribosomal protein S13 OS=Homo sapiens GN=RPS13 PE=1 SV=2	0.83	1	1.04	9.5E-01	-1.16	4.2E-01	-1.12	5.1E-01
RS15_HUMAN	40S ribosomal protein S15 OS=Homo sapiens GN=RPS15 PE=1 SV=2	1	2	1.03	9.4E-01	-1.36	2.3E-02	-1.33	7.4E-03
RS18_HUMAN	40S ribosomal protein S18 OS=Homo sapiens GN=RPS18 PE=1 SV=3	0.99	3	-1.04	8.7E-01	-1.15	1.3E-01	-1.20	1.9E-02
RS24_HUMAN	40S ribosomal protein S24 OS=Homo sapiens GN=RPS24 PE=1 SV=1	0.69	1	-1.16	7.8E-01	-1.09	8.1E-01	-1.26	2.5E-01
RS25_HUMAN	40S ribosomal protein S25 OS=Homo sapiens GN=RPS25 PE=1 SV=1	0.96	1	-1.22	6.8E-01	-1.07	7.5E-01	-1.30	2.9E-03
RS6_HUMAN	40S ribosomal protein S6 OS=Homo sapiens GN=RPS6 PE=1 SV=1	1	4	-1.09	7.3E-01	-1.09	4.9E-01	-1.19	6.6E-03
RS8_HUMAN	40S ribosomal protein S8 OS=Homo sapiens GN=RPS8 PE=1 SV=2	1	3	-1.23	2.6E-01	-1.16	1.9E-01	-1.43	3.0E-08
RT36_HUMAN	28S ribosomal protein S36, mitochondrial OS=Homo sapiens GN=MRPS36 PE=1 SV=2	1	9	-1.11	8.7E-01	-1.32	2.8E-03	-1.47	9.1E-03
S10A1_HUMAN	Protein S100-A1 OS=Homo sapiens GN=S100A1 PE=1 SV=2	1	3	1.11	8.7E-01	-1.71	5.3E-03	-1.54	1.5E-01
SAA_HUMAN	Serum amyloid A protein OS=Homo sapiens GN=SAA1 PE=1 SV=2	0.99	2	1.14	9.8E-01	-7.32	1.0E-04	-6.41	1.7E-04
SAMP_HUMAN	Serum amyloid P-component OS=Homo sapiens GN=APCS PE=1 SV=2	1	10	1.09	9.4E-01	-1.25	3.4E-01	-1.15	6.7E-01
SAP_HUMAN	Proactivator polypeptide OS=Homo sapiens GN=PSAP PE=1 SV=2	1	5	-1.07	9.4E-01	-1.24	4.2E-01	-1.33	2.4E-01
SDPR_HUMAN	Serum deprivation-response protein OS=Homo sapiens GN=SDPR PE=1 SV=3	1	14	-1.08	6.8E-01	-1.03	4.9E-01	-1.12	1.2E-01
SEPT7_HUMAN	Septin-7 OS=Homo sapiens GN=SEPT7 PE=1 SV=2	0.93	1	1.26	7.5E-01	1.00	9.6E-01	1.27	4.0E-01
SET_HUMAN	Protein SET OS=Homo sapiens GN=SET PE=1 SV=3	0.7	1	-1.00	9.7E-01	-1.27	2.9E-01	-1.27	3.9E-02
SGCB_HUMAN	Beta-sarcoglycan OS=Homo sapiens GN=SGCB PE=1 SV=1	1	8	-1.17	6.8E-01	-1.05	7.3E-01	-1.23	1.5E-01
SGCG_HUMAN	Gamma-sarcoglycan OS=Homo sapiens GN=SGCG PE=1 SV=3	0.99	2	-1.03	9.7E-01	-1.13	3.8E-01	-1.17	4.8E-01
SH3BG_HUMAN	SH3 domain-binding glutamic acid-rich protein OS=Homo sapiens GN=SH3BGR PE=1 SV=3	1	7	-1.12	8.1E-01	-1.21	3.9E-01	-1.36	8.5E-03
SIAE_HUMAN	Sialate O-acetylesterase OS=Homo sapiens GN=SIAE PE=2 SV=1	1	10	-1.21	6.9E-01	-1.54	2.6E-02	-1.87	7.3E-03
SODC_HUMAN	Superoxide dismutase [Cu-Zn] OS=Homo sapiens GN=SOD1 PE=1 SV=2	1	19	-1.12	6.8E-01	-1.10	5.4E-01	-1.24	1.9E-02
SODE_HUMAN	Extracellular superoxide dismutase [Cu-Zn] OS=Homo sapiens GN=SOD3 PE=1 SV=2	1	12	-1.04	9.4E-01	1.14	7.6E-01	1.09	8.7E-01
SODM_HUMAN	Superoxide dismutase [Mn], mitochondrial OS=Homo sapiens GN=SOD2 PE=1 SV=2	1	15	1.11	3.4E-01	-1.33	1.0E-02	-1.20	8.0E-02
SRBS2_HUMAN	Sorbin and SH3 domain-containing protein 2 OS=Homo sapiens GN=SORBS2 PE=1 SV=3	0.96	1	1.44	6.5E-01	1.01	9.4E-01	1.46	2.0E-01
SRCA_HUMAN	Sarcalumenin OS=Homo sapiens GN=SRL PE=2 SV=2	1	23	-1.12	9.0E-01	-1.24	3.1E-01	-1.38	1.2E-01
SRCH_HUMAN	Sarcoplasmic reticulum histidine-rich calcium-binding protein OS=Homo sapiens GN=HRC PE=2 SV=1	1	23	-1.36	3.3E-01	1.02	8.9E-01	-1.33	6.5E-02
STIM2_HUMAN	Stromal interaction molecule 2 OS=Homo sapiens GN=STIM2 PE=1 SV=2	0.3	1	14.56	5.8E-01	-1.35	9.6E-01	10.77	1.4E-01
SUCA_HUMAN	Succinyl-CoA ligase [GDP-forming] subunit alpha, mitochondrial OS=Homo sapiens GN=SUCLG1 PE=1 SV=4	1	9	-1.01	9.7E-01	-1.33	8.3E-02	-1.35	2.7E-02
TAGL_HUMAN	Transgelin OS=Homo sapiens GN=TAGLN PE=1 SV=4	0.52	1	3.24	4.7E-01	1.36	9.6E-01	4.40	3.8E-02
TBA1B_HUMAN	Tubulin alpha-1B chain OS=Homo sapiens GN=TUBA1B PE=1 SV=1	1	3	1.19	8.1E-01	1.01	9.8E-01	1.20	5.5E-01
TBB2A_HUMAN	Tubulin beta-2A chain OS=Homo sapiens GN=TUBB2A PE=1 SV=1	0.98	2	1.11	9.6E-01	1.51	4.1E-01	1.67	1.7E-01
TBB2C_HUMAN	Tubulin beta-2C chain OS=Homo sapiens GN=TUBB2C PE=1 SV=1	0.98	3	1.13	8.7E-01	-1.06	9.0E-01	1.07	7.7E-01
TBB3_HUMAN	Tubulin beta-3 chain OS=Homo sapiens GN=TUBB3 PE=1 SV=2	0.86	1	-1.06	9.5E-01	1.13	7.3E-01	1.07	8.2E-01
TBB4_HUMAN	Tubulin beta-4 chain OS=Homo sapiens GN=TUBB4 PE=1 SV=2	0.93	1	-1.17	8.7E-01	-1.13	7.1E-01	-1.32	9.4E-02
TBB5_HUMAN	Tubulin beta chain OS=Homo sapiens GN=TUBB PE=1 SV=2	0.99	19	1.10	8.7E-01	1.02	9.6E-01	1.12	5.9E-01
TBB6_HUMAN	Tubulin beta-6 chain OS=Homo sapiens GN=TUBB6 PE=1 SV=1	0.89	2	1.05	9.6E-01	-1.13	7.3E-01	-1.07	5.9E-01
TBB8_HUMAN	Tubulin beta-8 chain OS=Homo sapiens GN=TUBB8 PE=1 SV=2	0.98	2	1.09	9.5E-01	1.41	4.3E-01	1.54	5.7E-02
TBB8B_HUMAN	Tubulin beta-8 chain B OS=Homo sapiens PE=1 SV=1	0.65	1	-1.27	6.0E-01	-1.15	4.5E-01	-1.47	5.7E-02
TCTP_HUMAN	Translationally-controlled tumor protein OS=Homo sapiens GN=TPT1 PE=1 SV=1	0.63	1	-1.07	7.9E-01	-1.38	5.3E-02	-1.48	2.9E-03
TEBP_HUMAN	Prostaglandin E synthase 3 OS=Homo sapiens GN=PTGES3 PE=1 SV=1	1	4	-1.16	6.2E-01	-1.10	6.1E-01	-1.28	2.2E-02
TELT_HUMAN	Telethonin OS=Homo sapiens GN=TCAP PE=1 SV=1	1	4	1.11	7.9E-01	-1.51	7.3E-04	-1.36	2.3E-02
TFAM_HUMAN	Transcription factor A, mitochondrial OS=Homo sapiens GN=TFAM PE=1 SV=1	0.99	2	-1.09	9.4E-01	-1.24	2.7E-01	-1.35	2.9E-01
TGM7_HUMAN	Protein-glutamine gamma-glutamyltransferase Z OS=Homo sapiens GN=TGM7 PE=2 SV=1	0.83	1	1.01	9.9E-01	1.56	7.5E-06	1.57	3.6E-04
THIL_HUMAN	Acetyl-CoA acetyltransferase, mitochondrial OS=Homo sapiens GN=ACAT1 PE=1 SV=1	1	2	1.25	9.2E-01	-1.07	9.7E-01	1.17	7.0E-01
THIM_HUMAN	3-ketoacyl-CoA thiolase, mitochondrial OS=Homo sapiens GN=ACAA2 PE=1 SV=2	1	10	1.13	8.3E-01	-1.36	2.9E-01	-1.21	3.1E-01
THIO_HUMAN	Thioredoxin OS=Homo sapiens GN=TXN PE=1 SV=3	0	1	-1.19	3.4E-01	-1.01	9.1E-01	-1.20	6.8E-02
THRB_HUMAN	Prothrombin OS=Homo sapiens GN=F2 PE=1 SV=2	1	6	-1.33	6.8E-01	1.63	9.3E-02	1.22	5.3E-01
TI21L_HUMAN	TIM21-like protein, mitochondrial OS=Homo sapiens GN=C18orf55 PE=2 SV=1	0.59	1	-1.30	5.5E-01	-1.13	3.7E-01	-1.47	3.6E-02
TINAL_HUMAN	Tubulointerstitial nephritis antigen-like OS=Homo sapiens GN=TINAGL1 PE=1 SV=1	1	15	1.05	8.7E-01	-1.26	4.8E-03	-1.20	1.4E-01
TLE3_HUMAN	Transducin-like enhancer protein 3 OS=Homo sapiens GN=TLE3 PE=1 SV=2	0.33	1	-2.21	4.7E-01	2.16	1.8E-01	-1.03	8.8E-01
TM40L_HUMAN	Mitochondrial import receptor subunit TOM40B OS=Homo sapiens GN=TOMM40L PE=2 SV=1	0	1	-1.84	3.4E-01	1.80	1.1E-01	-1.02	9.3E-01
TMEDA_HUMAN	Transmembrane emp24 domain-containing protein 10 OS=Homo sapiens GN=TMED10 PE=1 SV=2	0.81	1	-1.02	9.6E-01	-1.25	1.0E-01	-1.27	1.4E-01
TNNC1_HUMAN	Troponin C, slow skeletal and cardiac muscles OS=Homo sapiens GN=TNNC1 PE=1 SV=1	1	42	-1.12	7.1E-01	-1.22	2.3E-03	-1.36	2.4E-03
TNNI3_HUMAN	Troponin I, cardiac muscle OS=Homo sapiens GN=TNNI3 PE=1 SV=3	1	41	-1.05	9.1E-01	-1.13	6.2E-02	-1.19	1.7E-01
TNNT1_HUMAN	Troponin T, slow skeletal muscle OS=Homo sapiens GN=TNNT1 PE=1 SV=4	1	5	-1.26	3.4E-01	1.17	1.5E-01	-1.08	6.1E-01
TNNT2_HUMAN	Troponin T, cardiac muscle OS=Homo sapiens GN=TNNT2 PE=1 SV=3	1	66	-1.16	5.8E-01	-1.23	2.2E-02	-1.42	1.2E-05
TPIS_HUMAN	Triosephosphate isomerase OS=Homo sapiens GN=TPI1 PE=1 SV=2	1	23	1.11	6.8E-01	-1.22	2.0E-01	-1.10	4.4E-01
TPM1_HUMAN	Tropomyosin alpha-1 chain OS=Homo sapiens GN=TPM1 PE=1 SV=2	1	73	-1.09	5.8E-01	-1.16	1.6E-02	-1.27	2.5E-04
TPM2_HUMAN	Tropomyosin beta chain OS=Homo sapiens GN=TPM2 PE=1 SV=1	1	62	-1.10	3.4E-01	-1.23	2.7E-03	-1.35	7.7E-06
TPM3_HUMAN	Tropomyosin alpha-3 chain OS=Homo sapiens GN=TPM3 PE=1 SV=1	1	10	-1.15	6.8E-01	-1.15	4.0E-01	-1.33	8.9E-03
TPM3L_HUMAN	Putative tropomyosin alpha-3 chain-like protein OS=Homo sapiens PE=5 SV=2	0.39	1	2.18	7.1E-01	-1.15	7.5E-01	1.90	8.5E-02
TPM4_HUMAN	Tropomyosin alpha-4 chain OS=Homo sapiens GN=TPM4 PE=1 SV=3	1	4	1.08	8.7E-01	-1.04	7.1E-01	1.04	8.2E-01
TPP1_HUMAN	Tripeptidyl-peptidase 1 OS=Homo sapiens GN=TPP1 PE=1 SV=2	1	7	-1.43	2.1E-01	-1.16	3.1E-01	-1.66	6.3E-04
TPPP_HUMAN	Tubulin polymerization-promoting protein OS=Homo sapiens GN=TPPP PE=1 SV=1	0.83	1	-1.04	9.6E-01	-1.41	1.7E-01	-1.47	1.3E-01
TRFE_HUMAN	Serotransferrin OS=Homo sapiens GN=TF PE=1 SV=2	1	108	-1.09	8.3E-01	1.98	2.8E-05	1.81	4.5E-04
TTHY_HUMAN	Transthyretin OS=Homo sapiens GN=TTR PE=1 SV=1	1	7	1.02	9.7E-01	1.49	6.4E-02	1.53	1.2E-02
UB2L3_HUMAN	Ubiquitin-conjugating enzyme E2 L3 OS=Homo sapiens GN=UBE2L3 PE=1 SV=1	1	3	-1.04	8.9E-01	-1.19	8.7E-02	-1.23	3.3E-02
UBIQ_HUMAN	Ubiquitin OS=Homo sapiens GN=RPS27A PE=1 SV=1	1	6	1.15	2.3E-02	-1.20	7.1E-05	-1.04	2.5E-01
UCRI_HUMAN	Cytochrome b-c1 complex subunit Rieske, mitochondrial OS=Homo sapiens GN=UQCRFS1 PE=1 SV=2	1	11	-1.05	8.7E-01	-1.33	2.0E-02	-1.40	2.4E-03
VDAC1_HUMAN	Voltage-dependent anion-selective channel protein 1 OS=Homo sapiens GN=VDAC1 PE=1 SV=2	1	10	1.01	9.7E-01	-1.17	4.9E-01	-1.16	2.2E-01
VDAC2_HUMAN	Voltage-dependent anion-selective channel protein 2 OS=Homo sapiens GN=VDAC2 PE=1 SV=2	1	14	-1.03	9.5E-01	-1.17	3.3E-01	-1.21	8.4E-02
VDAC3_HUMAN	Voltage-dependent anion-selective channel protein 3 OS=Homo sapiens GN=VDAC3 PE=1 SV=1	1	16	-1.04	9.4E-01	-1.14	4.7E-01	-1.19	1.4E-01
VIME_HUMAN	Vimentin OS=Homo sapiens GN=VIM PE=1 SV=4	1	18	1.33	7.3E-01	1.16	6.9E-01	1.54	1.4E-01
VTDB_HUMAN	Vitamin D-binding protein OS=Homo sapiens GN=GC PE=1 SV=1	1	15	-1.07	8.9E-01	1.58	9.7E-05	1.48	1.9E-02
VTNC_HUMAN	Vitronectin OS=Homo sapiens GN=VTN PE=1 SV=1	1	8	-1.06	9.3E-01	1.07	8.2E-01	1.01	9.6E-01
WDR46_HUMAN	WD repeat-containing protein 46 OS=Homo sapiens GN=WDR46 PE=1 SV=2	0	1	1.21	8.7E-01	-1.31	5.8E-01	-1.08	8.6E-01
YD007_HUMAN	Coiled-coil domain-containing protein FLJ25770 OS=Homo sapiens PE=2 SV=2	0.43	1	1.05	9.7E-01	-1.66	3.4E-01	-1.59	1.4E-05
ZA2G_HUMAN	Zinc-alpha-2-glycoprotein OS=Homo sapiens GN=AZGP1 PE=1 SV=1	1	9	-1.29	3.4E-01	1.35	1.9E-01	1.05	8.7E-01
ZN350_HUMAN	Zinc finger protein 350 OS=Homo sapiens GN=ZNF350 PE=1 SV=2	0.81	1	-1.12	7.9E-01	1.01	9.8E-01	-1.11	4.0E-01
