# Supplementary material for: Phosphoproteomic Profiling of Human Myocardial Tissues Distinguishes Ischemic from Non-Ischemic End Stage Heart Failure
Source: PLoS One. 2014 Aug 12;9(8):e104157. doi: 10.1371/journal.pone.0104157 (PMC4130503; doi:10.1371/journal.pone.0104157)
Supplement: Table S2 — Phosphoenriched peptide expression profiles. (PDF) [file pone.0104157.s004.pdf]

SUPPLEMENTAL TABLE 2: PHOSPHOENRICHED PEPTIDE EXPRESSION PROFILES

Entry Name	Protein Description	Modified Peptide Sequence	NIF v IF Fold Change	NIF v IF p-value (ANOVA)	IF v NF Fold Change	IF v NF p-value (ANOVA)	NIF v NF Fold Change	NIF v NF p-value (ANOVA)
4EBP2_HUMAN	Eukaryotic translation initiation factor 4E-binding protein 2 OS=Homo sapiens GN=EIF4EBP2 PE=1 SV=1	TVAISDAAQLPHDYC[160.0307]TTPGGT[181.014]LFS[166.9984]TTPGGTR	1.37	5.3E-01	-1.41	5.2E-01	-1.03	9.5E-01
4EBP2_HUMAN	Eukaryotic translation initiation factor 4E-binding protein 2 OS=Homo sapiens GN=EIF4EBP2 PE=1 SV=1	TVAISDAAQLPHDYC[160.0307]TTPGGTLFST[181.014]T[181.014]PGGTR	1.33	5.9E-01	-1.40	5.6E-01	-1.06	9.5E-01
AAKB2_HUMAN	5'-AMP-activated protein kinase subunit beta-2 OS=Homo sapiens GN=PRKAB2 PE=1 SV=1	DLSSS[166.9984]PPGPYGQEM[147.0354]YAFR	-1.78	6.0E-01	2.54	3.2E-01	1.43	7.6E-01
AAKB2_HUMAN	5'-AMP-activated protein kinase subunit beta-2 OS=Homo sapiens GN=PRKAB2 PE=1 SV=1	DLSSS[166.9984]PPGPYGQEMYAFR	1.54	7.3E-01	-2.50	3.5E-01	-1.62	4.3E-01
AAKB2_HUMAN	5'-AMP-activated protein kinase subunit beta-2 OS=Homo sapiens GN=PRKAB2 PE=1 SV=1	S[166.9984]HNDFVAILDLPEGEHQYK	-1.99	5.2E-01	-1.08	9.0E-01	-2.15	5.2E-01
ABCF1_HUMAN	ATP-binding cassette sub-family F member 1 OS=Homo sapiens GN=ABCF1 PE=1 SV=2	KLS[166.9984]VPT[181.014]S[166.9984]DEEDEVPAPKPR	2.14	2.5E-01	1.05	1.0E+00	2.24	1.9E-01
ABCF1_HUMAN	ATP-binding cassette sub-family F member 1 OS=Homo sapiens GN=ABCF1 PE=1 SV=2	KLSVPT[181.014]S[166.9984]DEEDEVPAPKPR	1.65	5.9E-01	-1.06	9.6E-01	1.55	6.0E-01
ABCF1_HUMAN	ATP-binding cassette sub-family F member 1 OS=Homo sapiens GN=ABCF1 PE=1 SV=2	KLSVPTS[166.9984]DEEDEVPAPKPR	1.66	1.4E-01	-1.30	5.4E-01	1.28	6.9E-01
ABCF1_HUMAN	ATP-binding cassette sub-family F member 1 OS=Homo sapiens GN=ABCF1 PE=1 SV=2	LSVPT[181.014]S[166.9984]DEEDEVPAPKPR	1.03	1.0E+00	-1.28	7.1E-01	-1.24	6.8E-01
ABCF1_HUMAN	ATP-binding cassette sub-family F member 1 OS=Homo sapiens GN=ABCF1 PE=1 SV=2	LSVPTS[166.9984]DEEDEVPAPKPR	-1.66	4.5E-01	-1.29	7.5E-01	-2.13	1.8E-01
ABLM1_HUMAN	Actin-binding LIM protein 1 OS=Homo sapiens GN=ABLIM1 PE=1 SV=3	RSS[166.9984]GREEDDEELLR	-1.27	8.1E-01	1.41	8.9E-01	1.12	9.2E-01
ABLM1_HUMAN	Actin-binding LIM protein 1 OS=Homo sapiens GN=ABLIM1 PE=1 SV=3	SS[166.9984]GREEDDEELLR	-1.27	8.6E-01	1.19	9.2E-01	-1.06	9.5E-01
ABLM1_HUMAN	Actin-binding LIM protein 1 OS=Homo sapiens GN=ABLIM1 PE=1 SV=3	STS[166.9984]QGSINSPVYSR	-1.04	9.2E-01	-1.40	6.8E-01	-1.46	4.0E-01
ABLM1_HUMAN	Actin-binding LIM protein 1 OS=Homo sapiens GN=ABLIM1 PE=1 SV=3	TLS[166.9984]PTPSAEGY[243.0297]QDVR	-2.15	2.5E-01	-1.18	8.1E-01	-2.55	8.3E-02
ACINU_HUMAN	Apoptotic chromatin condensation inducer in the nucleus OS=Homo sapiens GN=ACIN1 PE=1 SV=1	AES[166.9984]PAEKVPEESVLPLVQK	2.32	3.9E-01	1.24	8.2E-01	2.88	1.1E-01
ACINU_HUMAN	Apoptotic chromatin condensation inducer in the nucleus OS=Homo sapiens GN=ACIN1 PE=1 SV=1	KIS[166.9984]VVSATK	-2.15	1.3E-05	2.50	1.9E-05	1.16	9.0E-01
ACINU_HUMAN	Apoptotic chromatin condensation inducer in the nucleus OS=Homo sapiens GN=ACIN1 PE=1 SV=1	KSSS[166.9984]ISEEKGDS[166.9984]DDEKPR	1.84	6.2E-01	-1.68	6.5E-01	1.09	9.0E-01
ACINU_HUMAN	Apoptotic chromatin condensation inducer in the nucleus OS=Homo sapiens GN=ACIN1 PE=1 SV=1	KSSSISEEKGDS[166.9984]DDEKPR	1.43	6.2E-01	-1.34	7.7E-01	1.07	8.4E-01
ACINU_HUMAN	Apoptotic chromatin condensation inducer in the nucleus OS=Homo sapiens GN=ACIN1 PE=1 SV=1	RLS[166.9984]QPESAEK	1.01	9.8E-01	-1.05	9.6E-01	-1.04	9.9E-01
ACINU_HUMAN	Apoptotic chromatin condensation inducer in the nucleus OS=Homo sapiens GN=ACIN1 PE=1 SV=1	SSSISEEKGDS[166.9984]DDEKPR	-1.26	8.3E-01	1.09	9.2E-01	-1.15	9.0E-01
ACINU_HUMAN	Apoptotic chromatin condensation inducer in the nucleus OS=Homo sapiens GN=ACIN1 PE=1 SV=1	TAQVPS[166.9984]PPR	-2.93	2.3E-02	1.94	6.2E-01	-1.51	5.7E-01
ACM2_HUMAN	Muscarinic acetylcholine receptor M2 OS=Homo sapiens GN=CHRM2 PE=2 SV=1	DKKEPVANQDPVS[166.9984]PSLVQGR	1.18	9.1E-01	1.17	9.6E-01	1.37	8.9E-01
ACM2_HUMAN	Muscarinic acetylcholine receptor M2 OS=Homo sapiens GN=CHRM2 PE=2 SV=1	EPVANQDPVSPS[166.9984]LVQGR	-1.29	9.0E-01	1.50	7.5E-01	1.16	9.2E-01
ADA17_HUMAN	ADAM 17 OS=Homo sapiens GN=ADAM17 PE=1 SV=1	S[166.9984]FEDLTDHPVTR	-1.32	6.6E-01	1.10	8.6E-01	-1.20	7.7E-01
ADDB_HUMAN	Beta-adducin OS=Homo sapiens GN=ADD2 PE=1 SV=3	GLS[166.9984]QM[147.0354]T[181.014]T[181.014]S[166.9984]ADT DVDTSKDKTESVTSGPM[147.0354]SPEGSPS[166.9984]K	-2.79	5.9E-02	-1.60	8.0E-01	-4.46	7.0E-03
AFF4_HUMAN	AF4/FMR2 family member 4 OS=Homo sapiens GN=AFF4 PE=1 SV=1	SSS[166.9984]PGKPQAVSSLNSSHSR	-1.02	9.1E-01	-1.27	7.7E-01	-1.29	5.7E-01
AG2_HUMAN	Protein Ag2 homolog OS=Homo sapiens GN=AG2 PE=1 SV=2	S[166.9984]TQSLSLQR	2.25	1.3E-01	-1.52	5.1E-01	1.48	6.8E-01
AG2_HUMAN	Protein Ag2 homolog OS=Homo sapiens GN=AG2 PE=1 SV=2	SFLQS[166.9984]LEC[160.0307]LR	1.99	3.8E-01	-2.15	2.9E-01	-1.08	9.0E-01
AHNK_HUMAN	Neuroblast differentiation-associated protein AHNAK - Homo sapiens (Human)	KGDRS[166.9984]PEPGQTWTR	1.10	9.4E-01	2.14	8.0E-02	2.35	3.0E-02
AHNK_HUMAN	Neuroblast differentiation-associated protein AHNAK - Homo sapiens (Human)	LKS[166.9984]EDGVEGDLGETQSR	-1.07	9.4E-01	1.29	8.7E-01	1.20	9.1E-01
AKA12_HUMAN	A-kinase anchor protein 12 OS=Homo sapiens GN=AKAP12 PE=1 SV=3	DSEDVSERDS[166.9984]DKEM[147.0354]ATK	4.43	3.0E-03	-1.02	9.8E-01	4.33	8.0E-03
AKA12_HUMAN	A-kinase anchor protein 12 OS=Homo sapiens GN=AKAP12 PE=1 SV=3	EGVTPWAS[166.9984]FKK	1.99	2.9E-01	1.17	6.3E-01	2.34	2.0E-03
AKA12_HUMAN	A-kinase anchor protein 12 OS=Homo sapiens GN=AKAP12 PE=1 SV=3	RPS[166.9984]ESDKEDELDK	2.57	6.1E-01	-1.57	8.6E-01	1.64	7.2E-01
AKA12_HUMAN	A-kinase anchor protein 12 OS=Homo sapiens GN=AKAP12 PE=1 SV=3	RPS[166.9984]ESDKEDELDKVK	1.45	8.4E-01	1.10	9.3E-01	1.60	6.9E-01
AKA12_HUMAN	A-kinase anchor protein 12 OS=Homo sapiens GN=AKAP12 PE=1 SV=3	SPPS[166.9984]PVER	2.15	1.5E-01	-1.41	6.6E-01	1.53	7.9E-01
AKAP2_HUMAN	A-kinase anchor protein 2 OS=Homo sapiens GN=AKAP2 PE=1 SV=2	VKPPPS[166.9984]PTTEGPSLQPDLAPEEAAGTQRPK	-1.01	9.9E-01	1.80	3.9E-01	1.79	3.3E-01
AKTS1_HUMAN	Proline-rich AKT1 substrate 1 OS=Homo sapiens GN=AKT1S1 PE=1 SV=1	AATAARPPAPPPAPQPPS[166.9984]PTPS[166.9984]PPRPTLAR	4.67	5.3E-02	-2.18	7.0E-01	2.14	5.3E-01
AKTS1_HUMAN	Proline-rich AKT1 substrate 1 OS=Homo sapiens GN=AKT1S1 PE=1 SV=1	S[166.9984]LPVSVPVWGFK	1.12	7.1E-01	-1.10	8.0E-01	1.02	1.0E+00
ALBU_HUMAN	Serum albumin precursor - Homo sapiens (Human)	TC[160.0307]VADES[166.9984]AENC[160.0307]DK	1.27	7.1E-01	2.34	2.6E-05	2.99	1.3E-07
ALDOA_HUMAN	Fructose-bisphosphate aldolase A - Homo sapiens (Human)	GILAADES[166.9984]TGSIAK	1.43	9.0E-01	-1.85	5.9E-01	-1.29	6.0E-01
ALDOA_HUMAN	Fructose-bisphosphate aldolase A - Homo sapiens (Human)	GILAADESTGS[166.9984]IAK	-1.38	5.0E-01	1.02	8.5E-01	-1.35	5.9E-01
ALDOA_HUMAN	Fructose-bisphosphate aldolase A - Homo sapiens (Human)	GILAADESTGS[166.9984]IAKR	-1.86	1.2E-01	1.00	8.2E-01	-1.85	1.7E-01
ANK1_HUMAN	Ankyrin-1 OS=Homo sapiens GN=ANK1 PE=1 SV=3	EADAATS[166.9984]FLR	-1.35	7.0E-01	-1.19	8.6E-01	-1.61	4.9E-01
ANKR2_HUMAN	Ankyrin repeat domain-containing protein 2 OS=Homo sapiens GN=ANKRD2 PE=1 SV=3	KTS[166.9984]LDLR	1.32	8.9E-01	1.42	5.8E-01	1.87	3.7E-01
ANT3_HUMAN	Antithrombin-III OS=Homo sapiens GN=SERPINC1 PE=1 SV=1	ATEDEGS[166.9984]EQKIPEATNR	-1.40	2.9E-01	2.77	1.2E-01	1.98	5.6E-01
ANT3_HUMAN	Antithrombin-III OS=Homo sapiens GN=SERPINC1 PE=1 SV=1	KATEDEGS[166.9984]EQKIPEATNR	-1.50	3.4E-01	2.50	2.1E-01	1.66	6.6E-01
APOA1_HUMAN	Apolipoprotein A-I OS=Homo sapiens GN=APOA1 PE=1 SV=1	DYVS[166.9984]QFEGS[166.9984]ALGK	1.06	9.2E-01	3.63	2.0E-03	3.86	1.0E-03
AQP1_HUMAN	Aquaporin-1 OS=Homo sapiens GN=AQP1 PE=1 SV=3	VWTSGQVEEYDLDADDINS[166.9984]RVEM[147.0354]KPK	2.35	3.5E-01	-2.11	4.9E-01	1.11	9.9E-01
ARGL1_HUMAN	Arginine and glutamate-rich protein 1 OS=Homo sapiens GN=ARGLU1 PE=1 SV=1	ASS[166.9984]PPDRIDIFGR	1.42	7.9E-01	-2.07	4.2E-01	-1.45	6.6E-01
ARHG5_HUMAN	Rho guanine nucleotide exchange factor 5 OS=Homo sapiens GN=ARHGEF5 PE=1 SV=2	C[160.0307]S[166.9984]HQPISLLGSFLT[181.014]EESPDK	1.31	4.8E-01	-1.55	2.7E-01	-1.18	7.7E-01
AT11C_HUMAN	Probable phospholipid-transporting ATPase IG OS=Homo sapiens GN=ATP11C PE=1 SV=3	M[147.0354]QMVPSLPPAS[166.9984]EC[160.0307]AGEEKRVGTR	1.16	8.9E-01	2.74	4.5E-04	3.17	4.7E-06
B4GT5_HUMAN	Beta-1,4-galactosyltransferase 5 OS=Homo sapiens GN=B4GALT5 PE=2 SV=1	VQNAGYSVSRPEGDT[181.014]GK	1.59	5.8E-01	-1.41	8.0E-01	1.13	7.4E-01
BAG3_HUMAN	BAG family molecular chaperone regulator 3 OS=Homo sapiens GN=BAG3 PE=1 SV=3	SSVQGASS[166.9984]REGS[166.9984]PAR	6.71	3.0E-03	-1.68	7.4E-01	4.00	5.4E-02
BAG3_HUMAN	BAG family molecular chaperone regulator 3 OS=Homo sapiens GN=BAG3 PE=1 SV=3	VPPAPVPC[160.0307]PPPS[166.9984]PGPSAVPSSPK	1.33	7.3E-01	1.37	6.8E-01	1.82	3.2E-01
BAG4_HUMAN	BAG family molecular chaperone regulator 4 OS=Homo sapiens GN=BAG4 PE=1 SV=1	ELLELDSVETGGQDS[166.9984]VR	1.43	8.8E-01	1.70	7.6E-01	2.44	5.9E-01
BASI_HUMAN	Basigin precursor - Homo sapiens (Human)	KPEDVLDDDDAGSAPLKSS[166.9984]GQHQNDK	1.27	6.1E-01	-1.66	2.2E-01	-1.31	7.3E-01
BASI_HUMAN	Basigin precursor - Homo sapiens (Human)	RKPEDVLDDDDAGS[166.9984]APLK	-7.27	1.3E-05	4.62	4.6E-04	-1.57	6.8E-01
BASI_HUMAN	Basigin precursor - Homo sapiens (Human)	RKPEDVLDDDDAGSAPLKS[166.9984]SGQHQNDK	-4.36	3.6E-02	2.56	9.4E-02	-1.70	8.6E-01
BASI_HUMAN	Basigin precursor - Homo sapiens (Human)	RKPEDVLDDDDAGSAPLKSS[166.9984]GQHQNDK	-5.36	2.2E-04	2.48	5.9E-02	-2.16	5.7E-01
BCLF1_HUMAN	Bcl-2-associated transcription factor 1 OS=Homo sapiens GN=BCLAF1 PE=1 SV=2	FNDS[166.9984]EGDDTEETEDYR	1.89	4.4E-01	-1.65	5.6E-01	1.14	9.1E-01
BCLF1_HUMAN	Bcl-2-associated transcription factor 1 OS=Homo sapiens GN=BCLAF1 PE=1 SV=2	IDIS[166.9984]PSTLR	1.10	9.0E-01	-1.06	9.2E-01	1.04	9.6E-01
BCLF1_HUMAN	Bcl-2-associated transcription factor 1 OS=Homo sapiens GN=BCLAF1 PE=1 SV=2	KAEGEPQEES[166.9984]PLK	1.51	2.5E-01	-1.55	2.2E-01	-1.02	8.8E-01
BCLF1_HUMAN	Bcl-2-associated transcription factor 1 OS=Homo sapiens GN=BCLAF1 PE=1 SV=2	KAEGEPQEES[166.9984]PLKSK	1.69	3.5E-01	-1.73	2.7E-01	-1.02	9.7E-01
BCLF1_HUMAN	Bcl-2-associated transcription factor 1 OS=Homo sapiens GN=BCLAF1 PE=1 SV=2	LKDLFDYS[166.9984]PPLHK	1.30	7.1E-01	-1.36	6.5E-01	-1.05	9.1E-01
BCLF1_HUMAN	Bcl-2-associated transcription factor 1 OS=Homo sapiens GN=BCLAF1 PE=1 SV=2	RIDIS[166.9984]PSTLR	1.35	6.2E-01	-1.51	3.9E-01	-1.12	9.0E-01
BCLF1_HUMAN	Bcl-2-associated transcription factor 1 OS=Homo sapiens GN=BCLAF1 PE=1 SV=2	STFREES[166.9984]PLR	1.47	4.4E-01	-1.76	1.5E-01	-1.19	6.7E-01
BCLF1_HUMAN	Bcl-2-associated transcription factor 1 OS=Homo sapiens GN=BCLAF1 PE=1 SV=2	Y[243.0297]SPSQNS[166.9984]PIHHIPSR	2.05	1.5E-01	-2.54	3.8E-02	-1.24	7.0E-01
BCLF1_HUMAN	Bcl-2-associated transcription factor 1 OS=Homo sapiens GN=BCLAF1 PE=1 SV=2	YS[166.9984]PSQNS[166.9984]PIHHIPSR	1.30	9.6E-01	-1.71	5.9E-01	-1.32	3.6E-01
BNI3L_HUMAN	BCL2/adenovirus E1B 19 kDa protein-interacting protein 3-like OS=Homo sapiens GN=BNIP3L PE=1 SV=1	DHSSQS[166.9984]EEEVVEGEKEVEALKK	1.14	8.4E-01	1.26	8.1E-01	1.44	6.5E-01
BNIP2_HUMAN	BCL2/adenovirus E1B 19 kDa protein-interacting protein 2 OS=Homo sapiens GN=BNIP2 PE=1 SV=1	KGS[166.9984]ITEYTAAEEK	1.32	7.1E-01	1.40	7.0E-01	1.85	3.2E-01
BORG4_HUMAN	Cdc42 effector protein 4 OS=Homo sapiens GN=CDC42EP4 PE=1 SV=1	AGPDLPSLPSHALEDEGWAAAAPS[166.9984]PGSAR	2.09	1.4E-01	-1.68	4.8E-01	1.24	8.3E-01
CA144_HUMAN	UPF0485 protein C1orf144 OS=Homo sapiens GN=C1orf144 PE=1 SV=1	SKS[166.9984]PPKVPIVIQDDSLPAGPPPQIR	1.06	9.4E-01	1.07	9.1E-01	1.14	8.6E-01
CA144_HUMAN	UPF0485 protein C1orf144 OS=Homo sapiens GN=C1orf144 PE=1 SV=1	SKSPPKVPIVIQDDS[166.9984]LPAGPPPQIR	-1.06	9.4E-01	1.03	9.4E-01	-1.03	9.9E-01
CA198_HUMAN	Uncharacterized protein C1orf198 OS=Homo sapiens GN=C1orf198 PE=1 SV=1	SSS[166.9984]LDALGPTR	1.60	5.8E-01	1.24	6.5E-01	1.98	4.4E-01
CACB2_HUMAN	Voltage-dependent L-type calcium channel subunit beta-2 OS=Homo sapiens GN=CACNB2 PE=1 SV=3	SAS[166.9984]QAEEEPSVEPVKK	-1.54	5.8E-01	1.17	1.0E+00	-1.31	7.1E-01
CALD1_HUMAN	Caldesmon OS=Homo sapiens GN=CALD1 PE=1 SV=2	RGS[166.9984]IGENQVEVM[147.0354]VEEK	1.62	4.0E-01	-1.28	6.0E-01	1.26	9.7E-01
CALD1_HUMAN	Caldesmon OS=Homo sapiens GN=CALD1 PE=1 SV=2	TPDGNKS[166.9984]PAPKPSDLRPGDVSSK	1.79	6.4E-01	-5.25	1.6E-02	-2.93	1.9E-02
CALX_HUMAN	Calnexin precursor - Homo sapiens (Human)	AEEDEILNRS[166.9984]PR	1.85	1.2E-05	-1.14	4.7E-01	1.62	2.0E-03
CALX_HUMAN	Calnexin precursor - Homo sapiens (Human)	SDAEEDGGTVS[166.9984]QEEEDRKPK	2.24	1.3E-01	-1.95	2.8E-01	1.15	8.2E-01
CAPZB_HUMAN	F-actin-capping protein subunit beta - Homo sapiens (Human)	ELS[166.9984]QVLTQR	-2.24	6.0E-03	1.27	7.6E-01	-1.76	1.4E-01
CAR11_HUMAN	Caspase recruitment domain-containing protein 11 OS=Homo sapiens GN=CARD11 PE=2 SV=3	S[166.9984]S[166.9984]IM[147.0354]S[166.9984]ITAEPPGNDSIVR	1.18	9.2E-01	1.37	7.0E-01	1.63	5.0E-01
CASA1_BOVIN	Alpha-S1-casein precursor [Contains: Antioxidant peptide] - Bos taurus (Bovine)	DIGS[166.9984]ES[166.9984]TEDQAM[147.0354]EDIK	1.64	6.1E-01	-1.03	9.6E-01	1.59	7.2E-01
CASA1_BOVIN	Alpha-S1-casein precursor [Contains: Antioxidant peptide] - Bos taurus (Bovine)	DIGS[166.9984]ES[166.9984]TEDQAMEDIK	1.79	6.7E-01	-1.20	9.4E-01	1.49	7.4E-01
CASA1_BOVIN	Alpha-S1-casein precursor [Contains: Antioxidant peptide] - Bos taurus (Bovine)	DIGSES[166.9984]T[181.014]EDQAM[147.0354]EDIK	1.31	2.2E-01	-1.09	3.2E-01	1.20	8.5E-01
CASA1_BOVIN	Alpha-S1-casein precursor [Contains: Antioxidant peptide] - Bos taurus (Bovine)	DIGSES[166.9984]T[181.014]EDQAMEDIK	1.30	3.5E-02	-1.20	3.6E-01	1.08	8.8E-01
CASA1_BOVIN	Alpha-S1-casein precursor [Contains: Antioxidant peptide] - Bos taurus (Bovine)	VPQLEIVPNS[166.9984]AEER	-1.44	8.9E-01	1.42	9.2E-01	-1.02	9.8E-01
CASA1_BOVIN	Alpha-S1-casein precursor [Contains: Antioxidant peptide] - Bos taurus (Bovine)	YKVPQLEIVPNS[166.9984]AEER	-1.67	8.0E-01	1.46	9.2E-01	-1.14	9.3E-01
CASA2_BOVIN	Alpha-S2-casein precursor [Contains: Casocidin-1 - Bos taurus (Bovine)	EQLS[166.9984]TS[166.9984]EENSKK	2.21	8.0E-01	1.09	9.1E-01	2.41	6.0E-01
CASA2_BOVIN	Alpha-S2-casein precursor [Contains: Casocidin-1 - Bos taurus (Bovine)	EQLSTS[166.9984]EENSK	1.21	9.4E-01	1.50	6.5E-01	1.81	4.5E-01
CASA2_BOVIN	Alpha-S2-casein precursor [Contains: Casocidin-1 - Bos taurus (Bovine)	NM[147.0354]AINPS[166.9984]KENLC[160.0307]STFC[160.0307]K	1.52	9.2E-01	-1.02	9.5E-01	1.48	8.4E-01
CASA2_BOVIN	Alpha-S2-casein precursor [Contains: Casocidin-1 - Bos taurus (Bovine)	NMAINPS[166.9984]KENLC[160.0307]STFC[160.0307]K	1.21	8.9E-01	1.10	9.4E-01	1.33	8.2E-01
CASA2_BOVIN	Alpha-S2-casein precursor [Contains: Casocidin-1 - Bos taurus (Bovine)	TVDM[147.0354]ES[166.9984]TEVFTK	-1.64	8.9E-01	1.65	8.2E-01	1.01	9.6E-01
CASA2_BOVIN	Alpha-S2-casein precursor [Contains: Casocidin-1 - Bos taurus (Bovine)	TVDMES[166.9984]TEVFTK	-1.97	7.8E-01	1.58	9.2E-01	-1.25	9.2E-01
CASQ2_HUMAN	Calsequestrin-2 OS=Homo sapiens GN=CASQ2 PE=1 SV=2	KYDLLC[160.0307]LYYHEPVS[166.9984]SDKVTQK	-2.48	2.4E-01	-1.55	4.0E-01	-3.84	2.1E-02
CAV2_HUMAN	Caveolin-2 OS=Homo sapiens GN=CAV2 PE=1 SV=2	ADVQLFM[147.0354]DDDSY[243.0297]SHHS[166.9984]GLEYADPEK	2.60	3.2E-01	-3.06	1.9E-01	-1.18	9.1E-01
CBAA1_HUMAN	Calcium-binding atopy-related autoantigen 1 OS=Homo sapiens GN=CBARA1 PE=1 SV=1	MFRLNS[166.9984]LSALAELAVGSR	-1.64	5.4E-01	1.45	6.9E-01	-1.13	9.3E-01
CBX1_HUMAN	Chromobox protein homolog 1 - Homo sapiens (Human)	KADS[166.9984]DSEDKGEESKPK	1.11	9.4E-01	-1.85	2.7E-01	-1.66	3.3E-01
CC85A_HUMAN	Coiled-coil domain-containing protein 85A OS=Homo sapiens GN=CCDC85A PE=2 SV=2	SAS[166.9984]PEHPQKPR	1.17	4.0E-03	-2.72	8.9E-05	-2.32	4.9E-01
CCD18_HUMAN	Coiled-coil domain-containing protein 18 OS=Homo sapiens GN=CCDC18 PE=1 SV=1	LEQSQKM[147.0354]VIEKEQS[166.9984]LQES[166.9984]K	2.24	8.9E-01	-5.88	3.2E-01	-2.63	3.2E-01
CCD86_HUMAN	Coiled-coil domain-containing protein 86 OS=Homo sapiens GN=CCDC86 PE=1 SV=1	LGGLRPES[166.9984]PESLTSVSR	2.02	2.8E-01	-1.76	4.8E-01	1.15	8.8E-01
CCNY_HUMAN	Cyclin-Y OS=Homo sapiens GN=CCNY PE=1 SV=2	SAS[166.9984]ADNLTLPR	1.36	7.1E-01	1.29	8.0E-01	1.76	4.7E-01
CD44_HUMAN	CD44 antigen precursor - Homo sapiens (Human)	KPSGLNGEASKS[166.9984]QEMVHLVNK	-1.68	6.9E-01	1.58	7.6E-01	-1.06	9.7E-01
CDN1B_HUMAN	Cyclin-dependent kinase inhibitor 1B OS=Homo sapiens GN=CDKN1B PE=1 SV=1	VSNGS[166.9984]PSLER	-1.23	9.2E-01	-1.13	6.8E-01	-1.38	6.4E-01
CE042_HUMAN	Uncharacterized protein C5orf42 OS=Homo sapiens GN=C5orf42 PE=2 SV=2	KT[181.014]LAS[166.9984]KTISIS[166.9984]EEVR	1.41	2.1E-01	-1.41	8.3E-02	-1.00	8.0E-01
CE110_HUMAN	Centrosomal protein of 110 kDa OS=Homo sapiens GN=CEP110 PE=1 SV=3	S[166.9984]FQS[166.9984]EAPLKRGIVSAQDAS[166.9984]LQER	-1.09	9.4E-01	-1.98	2.6E-04	-2.16	2.0E-03
CF142_HUMAN	Uncharacterized protein C6orf142 OS=Homo sapiens GN=C6orf142 PE=2 SV=2	IPEESSDKS[166.9984]PETVNR	1.54	6.0E-01	2.27	2.1E-01	3.51	4.0E-03
CF142_HUMAN	Uncharacterized protein C6orf142 OS=Homo sapiens GN=C6orf142 PE=2 SV=2	YANLSS[166.9984]PTSTVSESQLTKPGVIRPVPVK	-1.13	8.6E-01	1.45	4.7E-01	1.28	6.6E-01
CF203_HUMAN	Uncharacterized protein C6orf203 OS=Homo sapiens GN=C6orf203 PE=1 SV=1	VDEEDSDEES[166.9984]HHDEM[147.0354]SEQEEELEDDPTVVK	2.24	5.7E-01	-3.56	2.2E-01	-1.59	5.7E-01
CHD9_HUMAN	Chromodomain-helicase-DNA-binding protein 9 OS=Homo sapiens GN=CHD9 PE=1 SV=1	NM[147.0354]AAM[147.0354]FPM[147.0354]LLS GM[147.0354]AGLPNLLGMGGLLTKPTESGT[181.014]EDK	2.22	7.8E-01	-4.15	3.3E-01	-1.87	4.4E-01
CHSP1_HUMAN	Calcium-regulated heat stable protein 1 - Homo sapiens (Human)	ERS[166.9984]PS[166.9984]PLRGNVVPS[166.9984]PLPTR	2.19	7.3E-02	-1.78	3.2E-01	1.23	6.8E-01
CHSP1_HUMAN	Calcium-regulated heat stable protein 1 - Homo sapiens (Human)	ERS[166.9984]PS[166.9984]PLRGNVVPSPLPTR	1.77	2.9E-01	-1.76	3.1E-01	1.01	1.0E+00
CHSP1_HUMAN	Calcium-regulated heat stable protein 1 - Homo sapiens (Human)	GNVVPS[166.9984]PLPTR	1.87	2.7E-01	-2.23	1.4E-01	-1.19	6.9E-01
CHSP1_HUMAN	Calcium-regulated heat stable protein 1 - Homo sapiens (Human)	TFS[166.9984]ATVR	-2.29	4.1E-02	1.63	3.6E-01	-1.41	5.8E-01
CI078_HUMAN	Uncharacterized protein C9orf78 OS=Homo sapiens GN=C9orf78 PE=1 SV=1	VGDTEKPEPERS[166.9984]PPNR	1.53	4.4E-01	-1.41	6.3E-01	1.09	8.9E-01
CI139_HUMAN	Uncharacterized protein C9orf139 OS=Homo sapiens GN=C9orf139 PE=2 SV=1	GFC[160.0307]PEM[147.0354]GQNESLS[166.9984]EERK	2.11	4.3E-01	-1.31	8.4E-01	1.62	4.9E-01
CK046_HUMAN	Uncharacterized protein C11orf46 OS=Homo sapiens GN=C11orf46 PE=2 SV=1	KPES[166.9984]DGRTAKALR	1.25	8.5E-01	-1.47	5.8E-01	-1.18	7.2E-01
CKAP5_HUMAN	Cytoskeleton-associated protein 5 OS=Homo sapiens GN=CKAP5 PE=1 SV=3	WNFTT[181.014]PRDEY[243.0297]IEQLKT[181.014]QM[147.0354]SS[166.9984]C[160.0307]VAK	-1.38	6.7E-01	1.37	5.9E-01	-1.01	9.0E-01
CLC14_HUMAN	C-type lectin domain family 14 member A OS=Homo sapiens GN=CLEC14A PE=1 SV=1	KES[166.9984]M[147.0354]GPPGLESDPEPAALGSSSAHC[160.0307]TNNGVK	-1.74	3.6E-01	1.68	5.3E-01	-1.03	9.5E-01
CLC14_HUMAN	C-type lectin domain family 14 member A OS=Homo sapiens GN=CLEC14A PE=1 SV=1	KES[166.9984]MGPPGLESDPEPAALGSSSAHC[160.0307]TNNGVK	1.50	7.3E-01	-1.61	6.4E-01	-1.07	9.0E-01
CLIP3_HUMAN	CAP-Gly domain-containing linker protein 3 OS=Homo sapiens GN=CLIP3 PE=1 SV=3	QGLFASVSKISKAVDAPPSS[166.9984]VT[181.014]S[166.9984]TPR	-1.20	8.6E-01	-1.12	8.4E-01	-1.34	6.8E-01
CLUL1_HUMAN	Clusterin-like protein 1 OS=Homo sapiens GN=CLUL1 PE=2 SV=1	APDHGGLIS[166.9984]KM[147.0354]LPGQDR	1.92	2.4E-01	-1.78	3.6E-01	1.08	9.3E-01
CMYA5_HUMAN	Cardiomyopathy-associated protein 5 OS=Homo sapiens GN=CMYA5 PE=1 SV=3	KRNS[166.9984]FESQDVPTNK	-1.54	6.0E-01	-1.55	5.8E-01	-2.39	2.0E-01
COBL_HUMAN	Protein cordon-bleu OS=Homo sapiens GN=COBL PE=1 SV=2	KSS[166.9984]LGNDETDKEK	1.40	7.8E-01	1.29	6.7E-01	1.80	2.0E-01
COBL_HUMAN	Protein cordon-bleu OS=Homo sapiens GN=COBL PE=1 SV=2	RAPAPPPPQPPPPS[166.9984]PLIPNR	1.19	9.0E-01	1.26	7.7E-01	1.51	5.1E-01
COBL_HUMAN	Protein cordon-bleu OS=Homo sapiens GN=COBL PE=1 SV=2	VSLGS[166.9984]QIDLQK	1.76	3.7E-01	-1.40	4.3E-01	1.26	8.2E-01
CPEB4_HUMAN	Cytoplasmic polyadenylation element-binding protein 4 OS=Homo sapiens GN=CPEB4 PE=1 SV=1	GLNGGIT[181.014]PLNSIS[166.9984]PLK	-1.62	2.7E-01	1.61	3.5E-01	-1.01	9.7E-01
CPZIP_HUMAN	Capz-interacting protein - Homo sapiens (Human)	AM[147.0354]VS[166.9984]PFHS[166.9984]PPSTPSSPGVR	1.75	3.6E-01	3.08	2.4E-01	5.39	2.2E-02
CPZIP_HUMAN	Capz-interacting protein - Homo sapiens (Human)	AM[147.0354]VSPFHS[166.9984]PPSTPSSPGVR	1.14	9.2E-01	2.09	3.0E-01	2.38	1.4E-01
CPZIP_HUMAN	Capz-interacting protein - Homo sapiens (Human)	AMVS[166.9984]PFHS[166.9984]PPSTPSSPGVR	1.19	7.3E-01	1.43	4.4E-01	1.71	1.7E-01
CPZIP_HUMAN	Capz-interacting protein - Homo sapiens (Human)	AMVSPFHS[166.9984]PPSTPSSPGVR	1.58	4.3E-01	-1.03	9.2E-01	1.53	9.0E-02
CPZIP_HUMAN	Capz-interacting protein - Homo sapiens (Human)	APGS[166.9984]PLSSEGAAGEGVR	-1.11	9.4E-01	1.50	7.0E-01	1.35	7.3E-01
CPZIP_HUMAN	Capz-interacting protein - Homo sapiens (Human)	RSS[166.9984]EEVDGQHPAQEEVPESPQTSGPEAENR	3.39	1.2E-01	-4.14	6.6E-02	-1.22	7.3E-01
CPZIP_HUMAN	Capz-interacting protein - Homo sapiens (Human)	SKAPGS[166.9984]PLSSEGAAGEGVR	1.08	8.8E-01	-1.39	4.5E-01	-1.29	5.7E-01
CPZIP_HUMAN	Capz-interacting protein - Homo sapiens (Human)	SQS[166.9984]DC[160.0307]GELGDFR	1.37	9.5E-01	-1.80	7.8E-01	-1.31	7.7E-01
CPZIP_HUMAN	Capz-interacting protein - Homo sapiens (Human)	VDLGQNGEEKS[166.9984]PPNASHPPK	-2.54	3.5E-02	1.62	5.1E-01	-1.57	4.5E-01
CPZIP_HUMAN	Capz-interacting protein - Homo sapiens (Human)	VKSS[166.9984]PLIEK	-1.50	8.0E-01	-1.28	7.1E-01	-1.92	6.2E-01

CR025_HUMAN	Uncharacterized protein C18orf25 OS=Homo sapiens GN=C18orf25 PE=1 SV=2	RDS[166.9984]SESQLASTESDKPTTGR	-1.41	8.3E-01	1.05	7.0E-01	-1.35	9.5E-01
CR025_HUMAN	Uncharacterized protein C18orf25 OS=Homo sapiens GN=C18orf25 PE=1 SV=2	RDSSES[166.9984]QLASTESDKPTTGR	-1.12	8.9E-01	-1.28	8.0E-01	-1.43	5.9E-01
CRIP2_HUMAN	Cysteine-rich protein 2 - Homo sapiens (Human)	ASS[166.9984]VTTFTGEPNTC[160.0307]PR	-1.61	5.2E-04	1.36	6.4E-01	-1.19	6.8E-01
CRYAB_HUMAN	Alpha-crystallin B chain OS=Homo sapiens GN=CRYAB PE=1 SV=2	RPFFPFHSPS[166.9984]R	-1.64	6.3E-01	1.11	9.8E-01	-1.48	7.4E-01
CSDC2_HUMAN	Cold shock domain-containing protein C2 OS=Homo sapiens GN=CSDC2 PE=1 SV=1	DLPS[166.9984]PLPTK	2.69	3.7E-01	-1.77	6.4E-01	1.52	9.1E-01
CSDC2_HUMAN	Cold shock domain-containing protein C2 OS=Homo sapiens GN=CSDC2 PE=1 SV=1	DLPS[166.9984]PLPTKR	1.60	5.9E-01	-1.15	7.6E-01	1.39	9.0E-01
CSPG2_HUMAN	Versican core protein OS=Homo sapiens GN=VCAN PE=1 SV=3	TDGQVS[166.9984]GEAIK	2.31	1.1E-01	-1.05	9.1E-01	2.20	3.6E-01
CSRP3_HUMAN	Cysteine and glycine-rich protein 3 OS=Homo sapiens GN=CSRP3 PE=1 SV=1	FGES[166.9984]EKC[160.0307]PR	-1.13	7.8E-01	-1.05	8.5E-01	-1.18	6.6E-01
CSRP3_HUMAN	Cysteine and glycine-rich protein 3 OS=Homo sapiens GN=CSRP3 PE=1 SV=1	GIGYGQGAGC[160.0307]LST[181.014]DTGEHLGLQFQQSPKPAR	-1.12	9.9E-01	-1.10	7.0E-01	-1.23	7.7E-01
CSRP3_HUMAN	Cysteine and glycine-rich protein 3 OS=Homo sapiens GN=CSRP3 PE=1 SV=1	GIGYGQGAGC[160.0307]LSTDTGEHLGLQFQQS[166.9984]PKPAR	-1.25	3.4E-01	-1.04	1.0E+00	-1.29	3.4E-01
CSRP3_HUMAN	Cysteine and glycine-rich protein 3 OS=Homo sapiens GN=CSRP3 PE=1 SV=1	S[166.9984]LESTNVTDKDGELYC[160.0307]K	-1.65	3.1E-01	1.02	1.0E+00	-1.62	2.9E-01
CX026_HUMAN	UPF0368 protein Cxorf26 OS=Homo sapiens GN=CXorf26 PE=1 SV=1	GADS[166.9984]GEEKEEGINR	-1.04	8.9E-01	-1.16	8.2E-01	-1.21	6.1E-01
CX026_HUMAN	UPF0368 protein Cxorf26 OS=Homo sapiens GN=CXorf26 PE=1 SV=1	GADS[166.9984]GEEKEEGINREDK	-1.08	9.1E-01	-1.12	8.8E-01	-1.21	7.3E-01
CXA1_HUMAN	Gap junction alpha-1 protein OS=Homo sapiens GN=GJA1 PE=1 SV=2	KLAAGHELQPLAIVDQRPSS[166.9984]R	-1.11	9.6E-01	1.30	7.9E-01	1.17	8.8E-01
CXA1_HUMAN	Gap junction alpha-1 protein OS=Homo sapiens GN=GJA1 PE=1 SV=2	KLAAGHELQPLAIVDQRPSS[166.9984]RAS[166.9984]S[166.9984]R	1.62	7.4E-01	-1.92	5.9E-01	-1.18	8.1E-01
CXA1_HUMAN	Gap junction alpha-1 protein OS=Homo sapiens GN=GJA1 PE=1 SV=2	LAAGHELQPLAIVDQRPS[166.9984]SR	-1.12	8.6E-01	-1.16	7.0E-01	-1.29	6.3E-01
CXA1_HUMAN	Gap junction alpha-1 protein OS=Homo sapiens GN=GJA1 PE=1 SV=2	LAAGHELQPLAIVDQRPS[166.9984]SRAS[166.9984]S[166.9984]R AS[166.9984]S[166.9984]RPRPDDLEI	1.68	8.0E-01	-2.51	5.3E-01	-1.49	6.1E-01
CXA1_HUMAN	Gap junction alpha-1 protein OS=Homo sapiens GN=GJA1 PE=1 SV=2	LAAGHELQPLAIVDQRPSS[166.9984]RAS[166.9984]S[166.9984]R	-2.04	9.6E-02	1.29	6.8E-01	-1.58	4.4E-01
CXA1_HUMAN	Gap junction alpha-1 protein OS=Homo sapiens GN=GJA1 PE=1 SV=2	LVTGDRNNS[166.9984]SC[160.0307]R	1.02	9.5E-01	-1.66	5.7E-01	-1.63	3.6E-01
CXA1_HUMAN	Gap junction alpha-1 protein OS=Homo sapiens GN=GJA1 PE=1 SV=2	M[147.0354]GQAGS[166.9984]TISNS[166.9984]HAQPFDFPDDNQNSK	-2.19	5.2E-01	1.21	9.6E-01	-1.82	6.8E-01
CXA1_HUMAN	Gap junction alpha-1 protein OS=Homo sapiens GN=GJA1 PE=1 SV=2	M[147.0354]GQAGSTISNS[166.9984]HAQPFDFPDDNQNSK	-2.23	6.0E-01	1.09	8.0E-01	-2.04	6.2E-01
CXA1_HUMAN	Gap junction alpha-1 protein OS=Homo sapiens GN=GJA1 PE=1 SV=2	MGQAGS[166.9984]TIS[166.9984]NSHAQPFDFPDDNQNSK	-1.81	9.1E-01	-1.25	7.1E-01	-2.26	1.2E-01
CXA1_HUMAN	Gap junction alpha-1 protein OS=Homo sapiens GN=GJA1 PE=1 SV=2	MGQAGST[181.014]IS[166.9984]NSHAQPFDFPDDNQNSK	-1.22	8.9E-01	-2.31	4.5E-02	-2.81	4.3E-02
CXA1_HUMAN	Gap junction alpha-1 protein OS=Homo sapiens GN=GJA1 PE=1 SV=2	MGQAGSTIS[166.9984]NSHAQPFDFPDDNQNSK	-2.57	4.4E-01	-1.13	5.3E-01	-2.90	3.0E-01
CXA1_HUMAN	Gap junction alpha-1 protein OS=Homo sapiens GN=GJA1 PE=1 SV=2	SDPYHATS[166.9984]GALSPAK	-1.02	9.7E-01	-3.89	2.0E-03	-3.96	7.0E-03
CYC_HUMAN	Cytochrome c - Homo sapiens (Human)	KTGQAPGYS[166.9984]YTAANK	1.78	1.7E-01	-1.84	1.3E-01	-1.03	9.8E-01
CYC_HUMAN	Cytochrome c - Homo sapiens (Human)	KTGQAPGYSYT[181.014]AANK	1.05	9.4E-01	-1.41	6.5E-01	-1.35	8.3E-01
CYC_HUMAN	Cytochrome c - Homo sapiens (Human)	TGQAPGYS[166.9984]YTAANK	1.33	4.0E-01	-2.60	2.0E-03	-1.95	7.0E-01
DAP1_HUMAN	Death-associated protein 1 OS=Homo sapiens GN=DAP PE=1 SV=3	DKDDQEWESPS[166.9984]PPKPTVFISGVIAR	-1.09	8.8E-01	-1.01	9.9E-01	-1.10	8.9E-01
DESM_HUMAN	Desmin - Homo sapiens (Human)	TFGGAPGFPLGS[166.9984]PLSS[166.9984]PVFPR	1.41	7.7E-01	1.82	3.6E-01	2.55	6.2E-02
DESM_HUMAN	Desmin - Homo sapiens (Human)	TFGGAPGFPLGS[166.9984]PLSSPVFPR	2.16	3.5E-01	-1.25	9.7E-01	1.72	1.5E-01
DESM_HUMAN	Desmin - Homo sapiens (Human)	TFGGAPGFPLGSPLSS[166.9984]PVFPR	1.50	2.5E-01	-1.18	8.3E-01	1.27	6.2E-01
DESP_HUMAN	Desmoplakin OS=Homo sapiens GN=DSP PE=1 SV=3	SS[166.9984]SFSDTLEESSPIAAIFDTENLEK	1.06	9.4E-01	1.08	9.4E-01	1.14	8.9E-01
DNJB6_HUMAN	DnaJ homolog subfamily B member 6 OS=Homo sapiens GN=DNAJB6 PE=1 SV=2	HAPHC[160.0307]LS[166.9984]EEEGEQDRPR	1.89	3.0E-03	-1.46	6.8E-02	1.29	3.4E-01
DNJC5_HUMAN	DnaJ homolog subfamily C member 5 - Homo sapiens (Human)	SLS[166.9984]TSGESLYHVLGLDK	1.56	3.7E-01	-1.89	1.8E-01	-1.22	9.7E-01
DOCK8_HUMAN	Dedicator of cytokinesis protein 8 OS=Homo sapiens GN=DOCK8 PE=2 SV=3	LPPNY[243.0297]SM[147.0354]HSAEKVPLQNPPIK	-1.30	4.5E-01	-1.15	8.2E-01	-1.50	3.3E-01
DSG2_HUMAN	Desmoglein-2 OS=Homo sapiens GN=DSG2 PE=1 SV=2	VVPSFLPVDQGGS[166.9984]LVGR	2.33	1.7E-01	-2.29	2.1E-01	1.02	8.7E-01
DSG2_HUMAN	Desmoglein-2 OS=Homo sapiens GN=DSG2 PE=1 SV=2	WEEHRS[166.9984]LLSGR	1.77	5.6E-01	-2.81	1.8E-01	-1.58	6.4E-01
DTD1_HUMAN	D-tyrosyl-tRNA(Tyr) deacylase 1 OS=Homo sapiens GN=DTD1 PE=1 SV=2	SASS[166.9984]GAEGDVSSEREP	1.21	9.0E-01	-1.45	6.4E-01	-1.20	6.8E-01
DTL_HUMAN	Denticleless protein homolog OS=Homo sapiens GN=DTL PE=1 SV=2	GLAPSVDFQQSVT[181.014]VVLFQDENT[181.014]LVS[166.9984]AGAVDGIIK	1.65	7.2E-01	-3.53	1.8E-01	-2.13	1.5E-01
DUS27_HUMAN	Inactive dual specificity phosphatase 27 OS=Homo sapiens GN=DUSP27 PE=2 SV=1	KVGS[166.9984]ENKEEVVELSK	-1.39	7.7E-01	1.36	8.3E-01	-1.03	9.7E-01
E41L2_HUMAN	Band 4.1-like protein 2 OS=Homo sapiens GN=EPB41L2 PE=1 SV=1	S[166.9984]YTLVVAK	1.56	6.7E-01	1.37	7.8E-01	2.14	4.0E-01
EAN57_HUMAN	Protein EAN57 OS=Homo sapiens GN=EAN57 PE=2 SV=2	S[166.9984]VRDLEHWHGRK	2.12	3.9E-01	-1.91	5.8E-01	1.11	8.2E-01
EF1B_HUMAN	Elongation factor 1-beta - Homo sapiens (Human)	YGPADVEDTTGSGATDSKDDDDIDLFGS[166.9984]DDEEESEEAKR	1.08	9.1E-01	-1.68	3.3E-01	-1.56	4.0E-01
EF1D_HUMAN	Elongation factor 1-delta - Homo sapiens (Human)	ATAPQTQHVS[166.9984]PMR	-1.28	9.1E-01	1.19	9.6E-01	-1.08	9.5E-01
EF1D_HUMAN	Elongation factor 1-delta - Homo sapiens (Human)	KPATPAEDDEDDDIDLFGS[166.9984]DNEEEDKEAAQLR	1.60	2.3E-01	-1.89	8.5E-02	-1.18	6.5E-01
EF1D_HUMAN	Elongation factor 1-delta - Homo sapiens (Human)	KPATPAEDDEDDDIDLFGS[166.9984]DNEEEDKEAAQLREER	-1.61	5.3E-02	1.22	5.8E-01	-1.32	4.9E-01
EIF3G_HUMAN	Eukaryotic translation initiation factor 3 subunit G OS=Homo sapiens GN=EIF3G PE=1 SV=2	GIPLATGDT[181.014]SPEPELLPGAPLPPPK	2.00	4.0E-01	-1.74	5.8E-01	1.15	9.0E-01
EIF3G_HUMAN	Eukaryotic translation initiation factor 3 subunit G OS=Homo sapiens GN=EIF3G PE=1 SV=2	GIPLATGDTS[166.9984]PEPELLPGAPLPPPK	1.39	6.2E-01	-1.52	5.8E-01	-1.09	9.0E-01
EMD_HUMAN	Emerin OS=Homo sapiens GN=EMD PE=1 SV=1	DSAYQSITHYRPVS[166.9984]ASR	2.24	4.3E-02	-1.42	4.7E-01	1.58	4.4E-01
ESAM_HUMAN	Endothelial cell-selective adhesion molecule OS=Homo sapiens GN=ESAM PE=1 SV=1	ALRPPHGPPRPGALT[181.014]PTPS[166.9984]LSSQALPSPR	1.35	5.8E-01	-1.15	9.1E-01	1.18	6.8E-01
ESAM_HUMAN	Endothelial cell-selective adhesion molecule OS=Homo sapiens GN=ESAM PE=1 SV=1	ALRPPHGPPRPGALTPT[181.014]PS[166.9984]LS[166.9984]SQALPSPR	1.41	4.7E-01	-1.23	7.1E-01	1.15	8.8E-01
EVL_HUMAN	Ena/VASP-like protein - Homo sapiens (Human)	SNS[166.9984]VEKPVSSILSR	1.26	9.2E-01	-1.23	8.9E-01	1.02	9.1E-01
F10A1_HUMAN	Hsc70-interacting protein - Homo sapiens (Human)	KVEEDLKADEPS[166.9984]S[166.9984]EES[166.9984]DLEIDK	-1.54	2.5E-01	1.24	6.7E-01	-1.24	6.0E-01
F122A_HUMAN	Protein FAM122A OS=Homo sapiens GN=FAM122A PE=1 SV=1	RIDFIPVS[166.9984]PAPSPT[181.014]R	1.11	9.2E-01	-1.13	9.0E-01	-1.02	9.9E-01
F122A_HUMAN	Protein FAM122A OS=Homo sapiens GN=FAM122A PE=1 SV=1	RNS[166.9984]TTFPSR	1.42	6.8E-01	-1.75	3.5E-01	-1.23	7.3E-01
F122A_HUMAN	Protein FAM122A OS=Homo sapiens GN=FAM122A PE=1 SV=1	SNS[166.9984]APLIHGLSDTSPVFQAEAPSAR	1.12	8.9E-01	-1.39	6.0E-01	-1.24	7.3E-01
F122B_HUMAN	Protein FAM122B OS=Homo sapiens GN=FAM122B PE=1 SV=2	SSS[166.9984]APLIHGLSDLSQVFQPYTLR	-1.02	9.6E-01	-1.20	9.1E-01	-1.23	8.6E-01
F262_HUMAN	6-phosphofructo-2-kinase/fructose-2,6-biphosphatase 2 OS=Homo sapiens GN=PFKFB2 PE=1 SV=2	NYS[166.9984]VGSRPLKPLSPLR	1.58	6.5E-01	-1.39	7.4E-01	1.13	9.8E-01
F90AO_HUMAN	Putative protein FAM90A24 OS=Homo sapiens GN=FAM90A24P PE=5 SV=1	EVPQAAS[166.9984]KTHGLLQAS[166.9984]RPQAQDK	1.28	1.0E+00	1.55	4.3E-01	1.99	2.0E-01
FA49A_HUMAN	Protein FAM49A OS=Homo sapiens GN=FAM49A PE=1 SV=1	FY[243.0297]EFS[166.9984]IRLEKALQSLLESL TC[160.0307]PPY[243.0297]T[181.014]PT[181.014]QHLER	1.22	9.1E-01	-1.60	6.3E-01	-1.32	7.3E-01
FA54B_HUMAN	Protein FAM54B OS=Homo sapiens GN=FAM54B PE=1 SV=2	NAS[166.9984]VPNLR	2.51	1.0E-03	-4.67	9.6E-09	-1.86	3.2E-02
FBN1_HUMAN	Fibrillin-1 OS=Homo sapiens GN=FBN1 PE=1 SV=1	GNPEPPVS[166.9984]GEM[147.0354]DDNSLSPEAC[160.0307]YEC[160.0307]K	3.80	7.4E-04	-2.39	7.3E-02	1.59	6.4E-01
FCGRN_HUMAN	IgG receptor FcRn large subunit p51 OS=Homo sapiens GN=FCGRT PE=1 SV=1	S[166.9984]GLPAPWISLRGDDTGVLLPT[181.014]PGEAQDADLK	-1.09	9.2E-01	3.36	2.7E-01	3.10	2.2E-01
FETUA_HUMAN	Alpha-2-HS-glycoprotein precursor - Homo sapiens (Human)	C[160.0307]DSSPDS[166.9984]AEDVR	-2.32	7.8E-01	20.09	2.3E-11	8.67	2.4E-06
FETUA_HUMAN	Alpha-2-HS-glycoprotein precursor - Homo sapiens (Human)	C[160.0307]DSSPDS[166.9984]AEDVRK	2.41	9.2E-01	2.14	3.5E-01	5.15	5.9E-02
FETUA_HUMAN	Alpha-2-HS-glycoprotein precursor - Homo sapiens (Human)	HTFM[147.0354]GVVSLGSPS[166.9984]GEVSHPR	-1.88	7.6E-01	7.95	7.2E-04	4.24	1.1E-01
FETUA_HUMAN	Alpha-2-HS-glycoprotein precursor - Homo sapiens (Human)	HTFMGVVSLGSPS[166.9984]GEVSHPR	-2.09	3.9E-01	4.22	3.1E-02	2.02	5.4E-01
FETUA_HUMAN	Alpha-2-HS-glycoprotein precursor - Homo sapiens (Human)	HTFMGVVSLGSPSGEVS[166.9984]HPR	-2.17	3.7E-01	3.48	1.3E-01	1.60	7.4E-01
FGFR3_HUMAN	Fibroblast growth factor receptor 3 OS=Homo sapiens GN=FGFR3 PE=1 SV=1	VVGRAAEVPGPEPGQQEQLVFGS[166.9984]GDA VELS[166.9984]C[160.0307]PPPGGGPMGPTVWVK	1.53	5.8E-01	-3.84	7.0E-03	-2.51	3.0E-03
FHL2_HUMAN	Four and a half LIM domains protein 2 OS=Homo sapiens GN=FHL2 PE=1 SV=3	YIS[166.9984]FEER	-3.34	1.3E-02	-1.04	9.2E-01	-3.49	3.5E-02
FOXA1_HUMAN	Hepatocyte nuclear factor 3-alpha OS=Homo sapiens GN=FOXA1 PE=1 SV=1	SY[243.0297]PHAKPPYSYISLITM[147.0354]AIQRAPSKMLT[181.014]LSEI YQWIM[147.0354]DLFPY[243.0297]Y[243.0297]R	-1.57	2.1E-01	-2.27	1.4E-01	-3.55	1.1E-05
FRIH_HUMAN	Ferritin heavy chain OS=Homo sapiens GN=FTH1 PE=1 SV=2	KMGAPESGLAEYLFDKHTLGDS[166.9984]DNES	-1.39	7.1E-01	-1.17	9.6E-01	-1.63	6.7E-01
FTCD_HUMAN	Formimidoyltransferase-cyclodeaminase OS=Homo sapiens GN=FTCD PE=1 SV=2	AFVGEVGARSAAPGGGSVAAAAAAMGAA LGS[166.9984]M[147.0354]VGLMTY[243.0297]GRR	-1.75	7.5E-02	1.48	7.7E-01	-1.19	5.0E-01
FUND1_HUMAN	FUN14 domain-containing protein 1 OS=Homo sapiens GN=FUNDC1 PE=1 SV=1	NPPPQDYESDDDS[166.9984]YEVLDLTEYAR	-1.04	9.7E-01	-1.22	8.8E-01	-1.27	8.6E-01
G3BP1_HUMAN	Ras GTPase-activating protein-binding protein 1 OS=Homo sapiens GN=G3BP1 PE=1 SV=1	SSS[166.9984]PAPADIAQTVQEDLR	1.36	6.9E-01	-1.31	8.0E-01	1.04	9.8E-01
G3P_HUMAN	Glyceraldehyde-3-phosphate dehydrogenase - Homo sapiens (Human)	GALQNIIPAS[166.9984]TGAAK	-1.56	6.7E-01	1.78	4.9E-01	1.14	9.0E-01
G3P_HUMAN	Glyceraldehyde-3-phosphate dehydrogenase - Homo sapiens (Human)	IISNASC[160.0307]T[181.014]TNC[160.0307]LAPLAK	-1.27	8.0E-01	1.17	8.1E-01	-1.09	9.5E-01
G3P_HUMAN	Glyceraldehyde-3-phosphate dehydrogenase - Homo sapiens (Human)	VIHDNFGIVEGLM[147.0354]TTVHAITAT[181.014]QK	-1.32	6.3E-01	-1.35	7.7E-01	-1.79	1.4E-01
G3P_HUMAN	Glyceraldehyde-3-phosphate dehydrogenase - Homo sapiens (Human)	VIHDNFGIVEGLMTTVHAITAT[181.014]QK	-1.16	5.3E-01	-1.61	9.2E-01	-1.86	3.2E-01
GATA6_HUMAN	Transcription factor GATA-6 OS=Homo sapiens GN=GATA6 PE=1 SV=2	NT[181.014]S[166.9984]PT[181.014]TQPTAS GAGAPVM[147.0354]TGAGESTNPENSELK	-2.11	4.9E-02	-1.11	9.7E-01	-2.35	1.4E-02
GBP4_HUMAN	Guanylate-binding protein 4 OS=Homo sapiens GN=GBP4 PE=1 SV=2	KS[166.9984]EQLNK	2.24	2.4E-01	-1.29	7.6E-01	1.73	2.9E-01
GGT5_HUMAN	Gamma-glutamyltransferase 5 OS=Homo sapiens GN=GGT5 PE=2 SV=2	SPSSM[147.0354]VPSILINKAQGS[166.9984]K	-1.15	9.7E-01	-1.48	8.0E-01	-1.71	7.7E-01
GNPAT_HUMAN	Dihydroxyacetone phosphate acyltransferase OS=Homo sapiens GN=GNPAT PE=1 SV=1	FTSQLLDQGTS[166.9984]QC[160.0307]Y[243.0297]DVLSSDVQK	3.49	2.0E-01	-1.09	9.5E-01	3.19	2.8E-02
GPSM1_HUMAN	G-protein-signaling modulator 1 OS=Homo sapiens GN=GPSM1 PE=1 SV=1	LDDQRASVGS[166.9984]LPGLR	-2.20	6.6E-01	-2.23	5.7E-01	-4.92	1.4E-01
GPTC8_HUMAN	G patch domain-containing protein 8 OS=Homo sapiens GN=GPATCH8 PE=1 SV=1	GPKPEPPGS[166.9984]GSPAPPR	-1.86	1.5E-01	1.92	1.2E-01	1.03	9.6E-01
GRAM4_HUMAN	GRAM domain-containing protein 4 OS=Homo sapiens GN=GRAMD4 PE=1 SV=1	RLQT[181.014]TSSRS[166.9984]Y[243.0297]VPSAPAGLGK	-1.34	8.5E-01	1.07	9.9E-01	-1.26	8.5E-01
GSK3A_HUMAN	Glycogen synthase kinase-3 alpha OS=Homo sapiens GN=GSK3A PE=1 SV=2	GEPNVSY[243.0297]IC[160.0307]SR	1.53	5.5E-01	-1.14	8.8E-01	1.34	7.1E-01
H12_HUMAN	Histone H1.2 - Homo sapiens (Human)	S[166.9984]ETAPAAPAAAPPAEK	1.31	8.6E-01	-1.56	6.0E-01	-1.20	7.7E-01
H31_HUMAN	Histone H3.1 OS=Homo sapiens GN=HIST1H3A PE=1 SV=2	ST[181.014]ELLIR	1.39	8.0E-01	-2.13	2.9E-01	-1.53	6.5E-01
HABP4_HUMAN	Intracellular hyaluronan-binding protein 4 OS=Homo sapiens GN=HABP4 PE=1 SV=1	KSLPAPVAQRPDS[166.9984]PGGGLQAPGQK	1.30	6.6E-01	1.12	8.7E-01	1.45	4.8E-01
HABP4_HUMAN	Intracellular hyaluronan-binding protein 4 OS=Homo sapiens GN=HABP4 PE=1 SV=1	SLPAPVAQRPDS[166.9984]PGGGLQAPGQK	2.05	2.8E-01	-1.46	6.5E-01	1.41	6.6E-01
HAP28_HUMAN	28 kDa heat- and acid-stable phosphoprotein OS=Homo sapiens GN=PDAP1 PE=1 SV=1	KSLDS[166.9984]DES[166.9984]EDEEDDYQQK	1.03	9.5E-01	-1.23	6.2E-01	-1.19	6.8E-01
HAP28_HUMAN	28 kDa heat- and acid-stable phosphoprotein OS=Homo sapiens GN=PDAP1 PE=1 SV=1	S[166.9984]LDSDES[166.9984]EDEEDDYQQK	-1.18	7.5E-01	-1.11	8.0E-01	-1.31	4.8E-01
HAP28_HUMAN	28 kDa heat- and acid-stable phosphoprotein OS=Homo sapiens GN=PDAP1 PE=1 SV=1	SLDS[166.9984]DES[166.9984]EDEEDDYQQKR	1.01	9.4E-01	-1.41	7.0E-01	-1.39	4.8E-01
HDAC2_HUMAN	Histone deacetylase 2 OS=Homo sapiens GN=HDAC2 PE=1 SV=2	M[147.0354]LPHAPGVQM[147.0354]QAIPEDAVHEDS[166.9984]GDEDGEDPDKR	2.76	3.4E-01	-2.79	3.5E-01	-1.01	9.0E-01
HDAC2_HUMAN	Histone deacetylase 2 OS=Homo sapiens GN=HDAC2 PE=1 SV=2	MLPHAPGVQMQAIPEDAVHEDS[166.9984]GDEDGEDPDKR	-1.50	8.3E-01	1.24	9.2E-01	-1.21	9.3E-01
HDGF_HUMAN	Hepatoma-derived growth factor OS=Homo sapiens GN=HDGF PE=1 SV=1	AGDLLEDS[166.9984]PK	1.65	5.8E-01	-1.31	7.6E-01	1.27	9.4E-01
HDGF_HUMAN	Hepatoma-derived growth factor OS=Homo sapiens GN=HDGF PE=1 SV=1	AGDLLEDS[166.9984]PKRPK	1.19	8.2E-01	1.06	9.8E-01	1.26	7.7E-01
HDGF_HUMAN	Hepatoma-derived growth factor OS=Homo sapiens GN=HDGF PE=1 SV=1	GNAEGS[166.9984]S[166.9984]DEEGKLVIDEPAK	1.64	4.6E-01	-1.44	5.8E-01	1.14	9.9E-01
HDGF_HUMAN	Hepatoma-derived growth factor OS=Homo sapiens GN=HDGF PE=1 SV=1	GNAEGS[166.9984]S[166.9984]DEEGKLVIDEPAKEK	1.61	1.6E-01	-1.44	3.6E-01	1.12	8.9E-01
HDGF_HUMAN	Hepatoma-derived growth factor OS=Homo sapiens GN=HDGF PE=1 SV=1	GNAEGSS[166.9984]DEEGKLVIDEPAK	-1.37	9.3E-04	1.21	2.4E-04	-1.13	9.3E-01
HDGF_HUMAN	Hepatoma-derived growth factor OS=Homo sapiens GN=HDGF PE=1 SV=1	KGNAEGS[166.9984]S[166.9984]DEEGKLVIDEPAK	-1.10	9.0E-01	-1.05	8.6E-01	-1.15	6.8E-01
HDGF_HUMAN	Hepatoma-derived growth factor OS=Homo sapiens GN=HDGF PE=1 SV=1	NSTPS[166.9984]EPGSGR	-1.19	5.2E-01	1.02	9.3E-01	-1.16	6.0E-01
HDGF_HUMAN	Hepatoma-derived growth factor OS=Homo sapiens GN=HDGF PE=1 SV=1	RAGDLLEDS[166.9984]PK	-1.31	4.0E-01	1.04	9.4E-01	-1.25	4.4E-01
HDGF_HUMAN	Hepatoma-derived growth factor OS=Homo sapiens GN=HDGF PE=1 SV=1	RAGDLLEDS[166.9984]PKRPK	-1.75	1.6E-02	1.10	9.6E-01	-1.59	3.1E-02
HDGR2_HUMAN	Hepatoma-derived growth factor-related protein 2 OS=Homo sapiens GN=HDGFRP2 PE=1 SV=1	KRS[166.9984]EGFSMDR	-6.87	1.3E-02	2.88	4.3E-01	-2.39	5.5E-01
HEAT1_HUMAN	HEAT repeat-containing protein 1 OS=Homo sapiens GN=HEATR1 PE=1 SV=3	TVKMVIPALIQS[166.9984]DS[166.9984]GDSIEVS[166.9984]R	1.04	9.2E-01	1.96	5.3E-02	2.04	2.2E-02
HMGA1_HUMAN	High mobility group protein HMG-I/HMG-Y OS=Homo sapiens GN=HMGA1 PE=1 SV=3	KLEKEEEEGIS[166.9984]QES[166.9984]S[166.9984]EEEQ	1.01	9.7E-01	-1.37	6.3E-01	-1.36	4.7E-01
HMGA1_HUMAN	High mobility group protein HMG-I/HMG-Y OS=Homo sapiens GN=HMGA1 PE=1 SV=3	KLEKEEEEGISQES[166.9984]S[166.9984]EEEQ	1.09	9.2E-01	-1.52	4.6E-01	-1.39	4.2E-01
HN1_HUMAN	Hematological and neurological expressed 1 protein OS=Homo sapiens GN=HN1 PE=1 SV=3	RNS[166.9984]SEASSGDFLDLK	-1.18	9.2E-01	-1.20	8.2E-01	-1.41	7.2E-01
HNRL2_HUMAN	Heterogeneous nuclear ribonucleoprotein U-like protein 2 OS=Homo sapiens GN=HNRNPUL2 PE=1 SV=1	REEDEPEERS[166.9984]GDETPGSEVPGDK	1.88	1.7E-01	1.04	9.4E-01	1.96	9.4E-02
HNRPD_HUMAN	Heterogeneous nuclear ribonucleoprotein D0 - Homo sapiens (Human)	IDASKNEEDEGHSNS[166.9984]SPR	1.86	1.5E-01	-1.43	4.3E-01	1.30	6.7E-01
HNRPD_HUMAN	Heterogeneous nuclear ribonucleoprotein D0 - Homo sapiens (Human)	IDASKNEEDEGHSNSS[166.9984]PR	1.60	5.2E-01	-1.28	9.4E-01	1.25	9.8E-02
HNRPG_HUMAN	Heterogeneous nuclear ribonucleoprotein G - Homo sapiens (Human)	DVYLS[166.9984]PR	2.28	4.6E-01	-1.09	9.3E-01	2.09	1.3E-01
HNRPU_HUMAN	Heterogeneous nuclear ribonucleoprotein U - Homo sapiens (Human)	AKS[166.9984]PQPPVEEEDEHFDDTVVC[160.0307]LDTYNC[160.0307]DLHFK	1.33	5.5E-01	-1.14	8.5E-01	1.16	6.8E-01
HP1B3_HUMAN	Heterochromatin protein 1-binding protein 3 - Homo sapiens (Human)	TVNSTRET[181.014]PPK	1.45	5.8E-01	-1.52	4.3E-01	-1.05	9.0E-01
HS90A_HUMAN	Heat shock protein HSP 90-alpha - Homo sapiens (Human)	ES[166.9984]EDKPEIEDVGSDEEEEKK	-1.09	7.1E-01	1.80	8.6E-02	1.65	3.2E-01
HS90A_HUMAN	Heat shock protein HSP 90-alpha - Homo sapiens (Human)	ES[166.9984]EDKPEIEDVGSDEEEEKKDGDK	1.07	9.8E-01	1.24	6.8E-01	1.33	5.3E-01
HS90A_HUMAN	Heat shock protein HSP 90-alpha - Homo sapiens (Human)	ESEDKPEIEDVGS[166.9984]DEEEEKKDGDK	-3.42	1.7E-04	3.74	6.5E-06	1.09	9.0E-01
HS90B_HUMAN	Heat shock protein HSP 90-beta - Homo sapiens (Human)	IEDVGS[166.9984]DEEDDSGK	2.71	8.5E-02	2.76	4.3E-01	7.48	6.0E-03
HS90B_HUMAN	Heat shock protein HSP 90-beta - Homo sapiens (Human)	IEDVGS[166.9984]DEEDDSGKDK	1.29	9.1E-01	-1.03	9.9E-01	1.25	8.9E-01
HS90B_HUMAN	Heat shock protein HSP 90-beta - Homo sapiens (Human)	IEDVGS[166.9984]DEEDDSGKDKK	1.55	4.6E-01	-1.27	8.1E-01	1.22	7.2E-01
HSPB1_HUMAN	Heat shock protein beta-1 - Homo sapiens (Human)	GPS[166.9984]WDPFR	-3.99	3.6E-01	4.53	7.7E-04	1.14	6.1E-01
HSPB1_HUMAN	Heat shock protein beta-1 - Homo sapiens (Human)	GPS[166.9984]WDPFRDWYPHSR	-4.01	1.5E-04	1.76	4.8E-01	-2.28	7.2E-02
HSPB1_HUMAN	Heat shock protein beta-1 - Homo sapiens (Human)	QLS[166.9984]SGVSEIR	-3.32	5.1E-06	-1.07	8.0E-01	-3.56	1.4E-04
HSPB7_HUMAN	Heat shock protein beta-7 OS=Homo sapiens GN=HSPB7 PE=1 SV=1	AERS[166.9984]FHSSSSSSSSTSSSASR	-3.08	1.4E-01	5.03	1.0E-01	1.63	8.3E-01
HSPB7_HUMAN	Heat shock protein beta-7 OS=Homo sapiens GN=HSPB7 PE=1 SV=1	AERSFHSSSSSSSSS[166.9984]TSSSASR	-2.80	8.8E-02	3.83	5.2E-02	1.36	8.4E-01
HSPB7_HUMAN	Heat shock protein beta-7 OS=Homo sapiens GN=HSPB7 PE=1 SV=1	S[166.9984]FHSSSSSSSSTSSSASR	-2.56	2.0E-03	2.27	1.7E-01	-1.13	6.9E-01
HSPB7_HUMAN	Heat shock protein beta-7 OS=Homo sapiens GN=HSPB7 PE=1 SV=1	SFHS[166.9984]S[166.9984]SSSSSSSTSSSASR	-3.46	6.0E-03	2.83	5.1E-02	-1.22	8.2E-01
HSPB7_HUMAN	Heat shock protein beta-7 OS=Homo sapiens GN=HSPB7 PE=1 SV=1	SFHS[166.9984]SSSSSSSSTSSSASR	-3.20	1.5E-01	2.51	3.6E-01	-1.27	8.8E-01
HSPB8_HUMAN	Heat shock protein beta-8 OS=Homo sapiens GN=HSPB8 PE=1 SV=1	DPFRDS[166.9984]PLSSR	1.45	6.0E-01	-1.04	1.0E+00	1.39	6.6E-01
HSPB8_HUMAN	Heat shock protein beta-8 OS=Homo sapiens GN=HSPB8 PE=1 SV=1	FGVPAEGRT[181.014]PPPFPGEPWK	5.28	2.4E-05	-5.87	3.5E-06	-1.11	8.6E-01
HTSF1_HUMAN	HIV Tat-specific factor 1 OS=Homo sapiens GN=HTATSF1 PE=1 SV=1	DLDEEGS[166.9984]EKELHENVLDK	-1.06	9.5E-01	1.11	9.7E-01	1.05	9.8E-01
HTSF1_HUMAN	HIV Tat-specific factor 1 OS=Homo sapiens GN=HTATSF1 PE=1 SV=1	ESS[166.9984]PEKEAEEGC[160.0307]PEKESEEGC[160.0307]PK	-1.08	9.2E-01	-1.06	8.0E-01	-1.14	7.3E-01
HTSF1_HUMAN	HIV Tat-specific factor 1 OS=Homo sapiens GN=HTATSF1 PE=1 SV=1	VFDDES[166.9984]DEKEDEEYADEK	-1.78	3.9E-01	1.54	6.9E-01	-1.16	8.3E-01

HUG1_HUMAN	Protein HUG-1 OS=Homo sapiens GN=HUG1 PE=2 SV=1	LRRPET[181.014]S[166.9984]GISDAGGAR	1.62	7.3E-01	-1.90	6.8E-01	-1.17	9.7E-01
I23O_HUMAN	Indoleamine 2,3-dioxygenase OS=Homo sapiens GN=INDO PE=1 SV=1	ENKTS[166.9984]EDPSK	1.21	9.2E-01	-1.48	5.9E-01	-1.22	6.7E-01
ICAL_HUMAN	Calpastatin OS=Homo sapiens GN=CAST PE=1 SV=4	EGITGPPADSSKPIGPDDAIDALSSDFTC[160.0307]GS[166.9984]PTAAGK	1.20	6.7E-01	-1.54	4.4E-01	-1.28	9.8E-01
ICAL_HUMAN	Calpastatin OS=Homo sapiens GN=CAST PE=1 SV=4	EGITGPPADSSKPIGPDDAIDALSSDFTC[160.0307]GSPT[181.014]AAGK	-1.16	9.7E-01	-1.63	3.6E-01	-1.89	6.7E-01
ICAL_HUMAN	Calpastatin OS=Homo sapiens GN=CAST PE=1 SV=4	KEGITGPPADSSKPIGPDDAIDALSSDFTC[160.0307]GS[166.9984]PTAAGK	-1.07	9.2E-01	-1.86	2.9E-01	-2.00	6.6E-01
ICLN_HUMAN	Methylosome subunit pICln OS=Homo sapiens GN=CLNS1A PE=1 SV=1	FEEESKEPVADEEEEDS[166.9984]DDDVEPITEFR	1.06	9.2E-01	-1.42	5.0E-01	-1.33	4.9E-01
IF2P_HUMAN	Eukaryotic translation initiation factor 5B OS=Homo sapiens GN=EIF5B PE=1 SV=3	KQS[166.9984]FDDNDS[166.9984]EELEDKDSK	1.10	9.0E-01	1.01	9.6E-01	1.11	9.4E-01
IF2P_HUMAN	Eukaryotic translation initiation factor 5B OS=Homo sapiens GN=EIF5B PE=1 SV=3	NKPGPNIES[166.9984]GNEDDDASFK	-1.19	5.7E-01	-1.28	5.8E-01	-1.53	7.5E-02
IF2P_HUMAN	Eukaryotic translation initiation factor 5B OS=Homo sapiens GN=EIF5B PE=1 SV=3	VEMYS[166.9984]GS[166.9984]DDDDDFNKLPK	-3.89	8.3E-02	2.14	5.5E-01	-1.81	6.8E-01
IF2P_HUMAN	Eukaryotic translation initiation factor 5B OS=Homo sapiens GN=EIF5B PE=1 SV=3	WDGS[166.9984]EEDEDNSKK	-1.61	3.7E-01	-1.15	8.0E-01	-1.86	1.4E-01
IF4B_HUMAN	Eukaryotic translation initiation factor 4B OS=Homo sapiens GN=EIF4B PE=1 SV=2	SLENETLNKEEDC[160.0307]HS[166.9984]PTSKPPKPDQPLK	-1.11	6.3E-02	2.29	1.5E-01	2.06	5.3E-01
IF4B_HUMAN	Eukaryotic translation initiation factor 4B OS=Homo sapiens GN=EIF4B PE=1 SV=2	SQS[166.9984]SDTEQQSPTSGGGK	1.16	6.8E-01	1.34	5.6E-01	1.55	2.9E-01
IF4B_HUMAN	Eukaryotic translation initiation factor 4B OS=Homo sapiens GN=EIF4B PE=1 SV=2	TGS[166.9984]ESSQTGTSTTSSR	-1.98	1.1E-01	1.14	2.6E-02	-1.74	2.8E-01
IFT52_HUMAN	Intraflagellar transport protein 52 homolog OS=Homo sapiens GN=IFT52 PE=2 SV=3	Y[243.0297]LDTGGDVFVMLGEGGES[166.9984]R	-1.34	9.0E-01	-1.53	2.8E-01	-2.04	2.8E-01
IL6RB_HUMAN	Interleukin-6 receptor subunit beta OS=Homo sapiens GN=IL6ST PE=1 SV=2	SHIAQWS[166.9984]PHTPPR	1.43	5.7E-01	-2.23	9.2E-02	-1.56	3.4E-01
IPO4_HUMAN	Importin-4 OS=Homo sapiens GN=IPO4 PE=1 SV=2	S[166.9984]FAVGTLAETIQGLGAAS[166.9984]AQFVSR	-1.13	7.2E-01	-3.21	1.6E-04	-3.64	3.4E-06
ITA5_HUMAN	Integrin alpha-5 OS=Homo sapiens GN=ITGA5 PE=1 SV=2	LLESSLS[166.9984]SSEGEEPVEYK	1.25	6.1E-01	-1.56	2.7E-01	-1.24	6.4E-01
ITA5_HUMAN	Integrin alpha-5 OS=Homo sapiens GN=ITGA5 PE=1 SV=2	LLESSLSS[166.9984]SEGEEPVEYK	1.04	9.2E-01	-1.43	3.6E-01	-1.38	4.1E-01
ITFG3_HUMAN	Protein ITFG3 OS=Homo sapiens GN=ITFG3 PE=2 SV=1	KS[166.9984]QENLGNPSKNEDNVK	1.23	8.8E-01	-1.17	8.8E-01	1.05	9.8E-01
IWS1_HUMAN	Protein IWS1 homolog OS=Homo sapiens GN=IWS1 PE=1 SV=2	AAVLS[166.9984]DS[166.9984]EDEEKASAK	1.24	7.2E-01	-1.23	6.5E-01	1.01	8.8E-01
IWS1_HUMAN	Protein IWS1 homolog OS=Homo sapiens GN=IWS1 PE=1 SV=2	TIAS[166.9984]DS[166.9984]EEEAGKELSDK	-2.16	3.6E-02	1.76	2.7E-01	-1.23	6.8E-01
JADE3_HUMAN	Protein Jade-3 OS=Homo sapiens GN=PHF16 PE=1 SV=1	FAKS[166.9984]NGLEGSWSGNVTQK	1.41	6.5E-01	-1.13	1.0E-01	1.25	1.1E-02
JPH2_HUMAN	Junctophilin-2 OS=Homo sapiens GN=JPH2 PE=1 SV=2	ETPRPEGGSPS[166.9984]PAGT[181.014]PPQPK	2.26	2.0E-01	-1.42	6.7E-01	1.59	3.3E-01
JPH2_HUMAN	Junctophilin-2 OS=Homo sapiens GN=JPH2 PE=1 SV=2	GAGAAGLPQPPRES[166.9984]PQLHER	1.41	6.5E-01	-1.02	9.8E-01	1.38	4.6E-01
K0831_HUMAN	Uncharacterized protein KIAA0831 OS=Homo sapiens GN=KIAA0831 PE=1 SV=1	M[147.0354]AS[166.9984]PSGKGAR	1.04	1.0E+00	1.06	9.4E-01	1.10	9.2E-01
K1143_HUMAN	Uncharacterized protein KIAA1143 OS=Homo sapiens GN=KIAA1143 PE=1 SV=2	IQPQPPDEDGDHS[166.9984]DKEDEQPQVVVLK	-1.13	8.2E-01	-1.14	6.5E-01	-1.28	4.3E-01
K1383_HUMAN	Uncharacterized protein KIAA1383 OS=Homo sapiens GN=KIAA1383 PE=1 SV=2	SKAEC[160.0307]DNVGS[166.9984]VENGKTNSVVTC[160.0307]S[166.9984]GAGNGR	1.13	8.3E-01	1.58	6.2E-01	1.80	4.1E-01
K1704_HUMAN	Uncharacterized protein KIAA1704 OS=Homo sapiens GN=KIAA1704 PE=1 SV=1	KQDDS[166.9984]PPRPIIGPALPPGFIK	1.40	4.9E-01	-1.54	3.2E-01	-1.09	9.1E-01
K2C4_HUMAN	Keratin, type II cytoskeletal 4 - Homo sapiens (Human)	RGAFSSVS[166.9984]M[147.0354]SGGAGR	1.49	2.2E-01	-1.67	3.7E-01	-1.12	9.5E-01
KAD1_HUMAN	Adenylate kinase isoenzyme 1 OS=Homo sapiens GN=AK1 PE=1 SV=3	KVNAEGS[166.9984]VDSVFSQVC[160.0307]THLDALK	-1.06	9.4E-01	-1.01	9.6E-01	-1.08	9.6E-01
KAD1_HUMAN	Adenylate kinase isoenzyme 1 OS=Homo sapiens GN=AK1 PE=1 SV=3	VNAEGS[166.9984]VDSVFSQVC[160.0307]THLDALK	1.46	6.7E-01	-1.61	6.0E-01	-1.10	9.0E-01
KAD1_HUMAN	Adenylate kinase isoenzyme 1 OS=Homo sapiens GN=AK1 PE=1 SV=3	YGYTHLS[166.9984]TGDLLR	1.58	5.7E-01	-2.00	3.1E-01	-1.27	7.4E-01
KAP0_HUMAN	cAMP-dependent protein kinase type I-alpha regulatory subunit OS=Homo sapiens GN=PRKAR1A PE=1 SV=1	T[181.014]DSREDEISPPPPNPVVK	1.28	8.8E-01	1.35	3.5E-01	1.73	8.3E-02
KAP0_HUMAN	cAMP-dependent protein kinase type I-alpha regulatory subunit OS=Homo sapiens GN=PRKAR1A PE=1 SV=1	TDS[166.9984]REDEIS[166.9984]PPPPNPVVK	1.44	4.5E-01	1.13	9.1E-01	1.63	3.3E-01
KAP0_HUMAN	cAMP-dependent protein kinase type I-alpha regulatory subunit OS=Homo sapiens GN=PRKAR1A PE=1 SV=1	TDSREDEIS[166.9984]PPPPNPVVK	-2.81	4.0E-03	-1.77	3.5E-01	-4.98	8.9E-08
KAP2_HUMAN	cAMP-dependent protein kinase type II-alpha regulatory subunit OS=Homo sapiens GN=PRKAR2A PE=1 SV=2	RVS[166.9984]VC[160.0307]AETYNPDEEEEDTDPR	1.32	5.7E-01	-1.19	7.3E-01	1.11	8.6E-01
KAP2_HUMAN	cAMP-dependent protein kinase type II-alpha regulatory subunit OS=Homo sapiens GN=PRKAR2A PE=1 SV=2	VADAKGDS[166.9984]ES[166.9984]EEDEDLEVPVPSR	-1.04	9.2E-01	-1.12	9.8E-01	-1.15	9.0E-01
KAP3_HUMAN	cAMP-dependent protein kinase type II-beta regulatory subunit OS=Homo sapiens GN=PRKAR2B PE=1 SV=3	RAS[166.9984]VC[160.0307]AEAYNPDEEEDDAESR	1.39	7.6E-01	1.01	9.9E-01	1.40	7.9E-01
KAPCA_HUMAN	cAMP-dependent protein kinase catalytic subunit alpha OS=Homo sapiens GN=PRKACA PE=1 SV=2	TWT[181.014]LC[160.0307]GTPEYLAPEIILSK	-1.06	9.5E-01	1.03	9.1E-01	-1.03	9.8E-01
KAPCB_HUMAN	cAMP-dependent protein kinase catalytic subunit beta OS=Homo sapiens GN=PRKACB PE=1 SV=2	T[181.014]WTLC[160.0307]GTPEYLAPEIILSK	1.34	9.5E-01	-1.21	9.6E-01	1.11	9.0E-01
KCRM_HUMAN	Creatine kinase M-type OS=Homo sapiens GN=CKM PE=1 SV=2	GQS[166.9984]IDDMIPAQK	-1.19	9.4E-01	-2.11	3.9E-01	-2.52	3.8E-01
KCRM_HUMAN	Creatine kinase M-type OS=Homo sapiens GN=CKM PE=1 SV=2	GTGGVDTAAVGS[166.9984]VFDVSNADR	-1.24	8.5E-01	-2.04	2.7E-01	-2.52	5.9E-02
KCRM_HUMAN	Creatine kinase M-type OS=Homo sapiens GN=CKM PE=1 SV=2	RGT[181.014]GGVDTAAVGSVFDVSNADR	-2.11	3.7E-02	-1.79	5.8E-01	-3.77	8.4E-05
KCRM_HUMAN	Creatine kinase M-type OS=Homo sapiens GN=CKM PE=1 SV=2	RGTGGVDTAAVGS[166.9984]VFDVSNADR	-1.42	4.6E-01	-3.67	5.0E-03	-5.20	1.5E-08
KCRS_HUMAN	Creatine kinase, sarcomeric mitochondrial OS=Homo sapiens GN=CKMT2 PE=1 SV=2	LGYILTC[160.0307]PS[166.9984]NLGTGLR	1.30	9.4E-01	-2.28	6.8E-01	-1.76	4.6E-01
KCRU_HUMAN	Creatine kinase, ubiquitous mitochondrial precursor - Homo sapiens (Human)	LGYILTC[160.0307]PS[166.9984]NLGTGLR	1.02	9.4E-01	-1.62	6.8E-01	-1.60	4.6E-01
KNG1_HUMAN	Kininogen-1 OS=Homo sapiens GN=KNG1 PE=1 SV=2	ETTC[160.0307]S[166.9984]KESNEELTESC[160.0307]ETK	-1.39	6.3E-01	4.07	3.3E-05	2.93	2.4E-02
KNG1_HUMAN	Kininogen-1 OS=Homo sapiens GN=KNG1 PE=1 SV=2	ETTC[160.0307]S[166.9984]KESNEELTESC[160.0307]ETKK	-1.05	9.6E-01	2.47	5.3E-02	2.36	1.4E-01
KNG1_HUMAN	Kininogen-1 OS=Homo sapiens GN=KNG1 PE=1 SV=2	ETTC[160.0307]SKES[166.9984]NEELTESC[160.0307]ETK	-1.10	7.0E-01	2.16	8.0E-03	1.96	5.4E-02
KNG1_HUMAN	Kininogen-1 OS=Homo sapiens GN=KNG1 PE=1 SV=2	ETTC[160.0307]SKES[166.9984]NEELTESC[160.0307]ETKK	-1.30	1.4E-01	2.52	1.0E-03	1.93	6.2E-02
KPCG_HUMAN	Protein kinase C gamma type OS=Homo sapiens GN=PRKCG PE=1 SV=3	LHISDFSFLM[147.0354]VLGKGS[166.9984]FGK	-1.10	7.7E-01	-1.07	9.4E-01	-1.18	6.4E-01
LA_HUMAN	Lupus La protein OS=Homo sapiens GN=SSB PE=1 SV=2	FAS[166.9984]DDEHDEHDENGATGPVK	1.14	7.9E-01	-1.19	6.8E-01	-1.05	9.2E-01
LA_HUMAN	Lupus La protein OS=Homo sapiens GN=SSB PE=1 SV=2	FAS[166.9984]DDEHDEHDENGATGPVKR	1.35	7.1E-01	-1.45	5.9E-01	-1.07	7.7E-01
LAP2A_HUMAN	Lamina-associated polypeptide 2 isoform alpha - Homo sapiens (Human)	GPPDFS[166.9984]S[166.9984]DEEREPTPVLGSGAAAAGR	1.76	1.1E-01	-1.13	8.3E-01	1.55	2.4E-01
LAP2A_HUMAN	Lamina-associated polypeptide 2 isoform alpha - Homo sapiens (Human)	GPPDFSSDEEREPT[181.014]PVLGSGAAAAGR	2.04	6.2E-04	-1.58	7.2E-02	1.30	5.5E-01
LAP2A_HUMAN	Lamina-associated polypeptide 2 isoform alpha - Homo sapiens (Human)	SST[181.014]PLPTISSSAENTR	2.19	6.1E-02	-1.87	2.2E-01	1.17	8.2E-01
LARP7_HUMAN	La-related protein 7 OS=Homo sapiens GN=LARP7 PE=1 SV=1	KRSS[166.9984]S[166.9984]EDAESLAPR	1.18	8.6E-01	-1.03	9.4E-01	1.14	9.0E-01
LARP7_HUMAN	La-related protein 7 OS=Homo sapiens GN=LARP7 PE=1 SV=1	S[166.9984]RPTS[166.9984]EGSDIESTEPQK	1.09	9.5E-01	-1.28	7.7E-01	-1.18	7.9E-01
LARP7_HUMAN	La-related protein 7 OS=Homo sapiens GN=LARP7 PE=1 SV=1	SRPT[181.014]S[166.9984]EGSDIESTEPQK	1.19	8.8E-01	-1.42	6.5E-01	-1.19	8.1E-01
LARP7_HUMAN	La-related protein 7 OS=Homo sapiens GN=LARP7 PE=1 SV=1	SRPT[181.014]SEGSDIESTEPQK	-4.46	1.0E-02	3.48	7.0E-03	-1.28	8.9E-01
LASP1_HUMAN	LIM and SH3 domain protein 1 - Homo sapiens (Human)	MGPSGGEGM[147.0354]EPERRDSQDGS[166.9984]SYR	1.59	6.8E-01	1.02	1.0E+00	1.61	7.0E-01
LASP1_HUMAN	LIM and SH3 domain protein 1 - Homo sapiens (Human)	MGPSGGEGMEPERRDS[166.9984]QDGSSYR	-2.12	7.1E-01	2.85	5.2E-01	1.34	9.0E-01
LBH_HUMAN	Protein LBH OS=Homo sapiens GN=LBH PE=1 SV=1	LPS[166.9984]IVVEPTEGEVESGELR	-1.65	5.4E-01	1.18	9.1E-01	-1.40	7.5E-01
LEO1_HUMAN	RNA polymerase-associated protein LEO1 OS=Homo sapiens GN=LEO1 PE=1 SV=1	KLT[181.014]SDEEGEPSGK	1.14	9.2E-01	-1.34	7.3E-01	-1.17	7.7E-01
LETM2_HUMAN	LETM1 domain-containing protein LETM2, mitochondrial OS=Homo sapiens GN=LETM2 PE=2 SV=2	QVQTGHKPS[166.9984]TKEIVR	1.63	7.9E-01	2.31	3.9E-01	3.76	1.0E-01
LIGO2_HUMAN	Leucine-rich repeat and immunoglobulin-like domain-containing nogo receptor-interacting protein 2 OS=Homo sapiens GN=LINGO2 PE=2 SV=1	ATVLGDGTLEIRFAQDQDSGM[147.0354]YVC[160.0307]IASN AAGNDT[181.014]FT[181.014]AS[166.9984]LT[181.014]VKGFAS[166.9984]DR	-1.48	6.7E-01	-1.62	3.8E-01	-2.39	2.2E-01
LIMA1_HUMAN	LIM domain and actin-binding protein 1 - Homo sapiens (Human)	ETPHS[166.9984]PGVEDAPIAK	1.98	4.5E-01	1.44	6.9E-01	2.85	1.0E-01
LIMC1_HUMAN	LIM and calponin homology domains-containing protein 1 OS=Homo sapiens GN=LIMCH1 PE=1 SV=3	GSSDGRGS[166.9984]DSESDLPHR	1.05	9.5E-01	1.46	8.2E-01	1.54	6.8E-01
LIMC1_HUMAN	LIM and calponin homology domains-containing protein 1 OS=Homo sapiens GN=LIMCH1 PE=1 SV=3	S[166.9984]PEPEATLTFPFLDK	1.48	6.4E-01	-1.07	9.2E-01	1.38	8.3E-01
LMNA_HUMAN	Lamin-A/C - Homo sapiens (Human)	LRLS[166.9984]PS[166.9984]PTSQR	2.54	7.7E-02	-1.69	4.0E-01	1.50	6.2E-01
LMNA_HUMAN	Lamin-A/C - Homo sapiens (Human)	SGAQASSTPLS[166.9984]PTR	1.50	7.0E-01	-1.20	8.6E-01	1.25	8.7E-01
LMO7_HUMAN	LIM domain only protein 7 OS=Homo sapiens GN=LMO7 PE=1 SV=2	RGES[166.9984]LDNLDSPR	2.79	3.4E-02	1.32	7.8E-01	3.67	4.0E-03
LMO7_HUMAN	LIM domain only protein 7 OS=Homo sapiens GN=LMO7 PE=1 SV=2	SAS[166.9984]VNKEPVSLPGIM[147.0354]R	2.50	2.1E-01	1.08	9.1E-01	2.71	1.5E-01
LMO7_HUMAN	LIM domain only protein 7 OS=Homo sapiens GN=LMO7 PE=1 SV=2	SAS[166.9984]VNKEPVSLPGIMR	2.73	7.5E-01	-1.68	1.0E+00	1.63	5.7E-01
LMO7_HUMAN	LIM domain only protein 7 OS=Homo sapiens GN=LMO7 PE=1 SV=2	SHS[166.9984]PSASQSGSQLR	-1.11	9.1E-01	-1.27	6.5E-01	-1.41	8.3E-01
LMOD1_HUMAN	Leiomodin-1 OS=Homo sapiens GN=LMOD1 PE=1 SV=2	GS[166.9984]PKPS[166.9984]PQPSPKPS[166.9984]PK	9.66	1.5E-06	-1.92	1.1E-04	5.04	8.0E-03
LMOD1_HUMAN	Leiomodin-1 OS=Homo sapiens GN=LMOD1 PE=1 SV=2	GSPKPSPQPS[166.9984]PKPSPK	9.18	1.3E-05	-4.93	3.0E-03	1.86	7.8E-01
LMOD1_HUMAN	Leiomodin-1 OS=Homo sapiens GN=LMOD1 PE=1 SV=2	NSLS[166.9984]PATQR	4.40	4.0E-02	-6.84	2.0E-03	-1.56	7.1E-01
LMOD2_HUMAN	Leiomodin-2 OS=Homo sapiens GN=LMOD2 PE=2 SV=2	GTPSSSPYVS[166.9984]PR	-1.88	6.0E-01	2.34	6.7E-01	1.24	9.9E-01
LMOD2_HUMAN	Leiomodin-2 OS=Homo sapiens GN=LMOD2 PE=2 SV=2	S[166.9984]RPLS[166.9984]PVATPPPPPPPPPPPPSSQR	1.41	6.1E-01	-1.21	8.1E-01	1.16	9.1E-01
LMOD2_HUMAN	Leiomodin-2 OS=Homo sapiens GN=LMOD2 PE=2 SV=2	S[166.9984]RPLSPVATPPPPPPPPPPPPSSQR	1.59	4.6E-01	-1.39	6.8E-01	1.14	9.4E-01
LMOD2_HUMAN	Leiomodin-2 OS=Homo sapiens GN=LMOD2 PE=2 SV=2	SRPLS[166.9984]PVAT[181.014]PPPPPPPPPPPPSSQR	1.28	6.6E-01	-1.15	8.0E-01	1.11	8.8E-01
LMOD2_HUMAN	Leiomodin-2 OS=Homo sapiens GN=LMOD2 PE=2 SV=2	SRPLS[166.9984]PVATPPPPPPPPPPPPSSQR	1.66	4.5E-01	-1.57	6.0E-01	1.06	9.1E-01
LMOD2_HUMAN	Leiomodin-2 OS=Homo sapiens GN=LMOD2 PE=2 SV=2	YES[166.9984]IDEDELLASLSAEELKELER	1.25	7.1E-01	-1.25	7.0E-01	1.00	1.0E+00
LRC57_HUMAN	Leucine-rich repeat-containing protein 57 OS=Homo sapiens GN=LRRC57 PE=1 SV=1	DRGLT[181.014]EFPADLQKLT[181.014]S[166.9984]NLR	-5.13	7.0E-01	1.93	9.8E-01	-2.66	7.2E-01
LRP1_HUMAN	Prolow-density lipoprotein receptor-related protein 1 OS=Homo sapiens GN=LRP1 PE=1 SV=1	HS[166.9984]LASTDEKR	-2.68	3.4E-01	2.26	2.2E-01	-1.19	9.1E-01
LRP2_HUMAN	Low-density lipoprotein receptor-related protein 2 OS=Homo sapiens GN=LRP2 PE=1 SV=3	TKLGWPAGVTLDM[147.0354]IS[166.9984]KR	-1.71	5.4E-01	-1.12	9.7E-01	-1.92	4.3E-01
LRRF1_HUMAN	Leucine-rich repeat flightless-interacting protein 1 - Homo sapiens (Human)	NMPGLSAATLASLGGTSS[166.9984]R	-1.33	8.4E-01	-1.46	7.9E-01	-1.93	6.6E-01
LRRF2_HUMAN	Leucine-rich repeat flightless-interacting protein 2 OS=Homo sapiens GN=LRRFIP2 PE=1 SV=1	RGS[166.9984]GDTSSLIDPDTSLSELR	1.19	8.8E-01	-1.10	9.2E-01	1.07	9.8E-01
LTMD1_HUMAN	LETM1 domain-containing protein 1 OS=Homo sapiens GN=LETMD1 PE=1 SV=1	ALSRAMLLT[181.014]S[166.9984]YLPPPLLR	-2.62	1.4E-01	3.73	1.6E-01	1.42	8.2E-01
LU_HUMAN	Lutheran blood group glycoprotein precursor - Homo sapiens (Human)	GAPPPGEPGLS[166.9984]HSGSEQPEQTGLLM[147.0354]GGASGGAR	1.18	9.6E-01	-1.00	8.4E-01	1.18	8.8E-01
LU_HUMAN	Lutheran blood group glycoprotein precursor - Homo sapiens (Human)	GAPPPGEPGLSHS[166.9984]GSEQPEQTGLLMGGASGGAR	-2.76	1.7E-01	1.23	9.6E-01	-2.24	4.3E-01
LYRIC_HUMAN	Protein LYRIC OS=Homo sapiens GN=MTDH PE=1 SV=2	LSSQIS[166.9984]AGEEK	-1.77	2.2E-01	1.55	5.4E-01	-1.15	8.1E-01
LYRIC_HUMAN	Protein LYRIC OS=Homo sapiens GN=MTDH PE=1 SV=2	SQEPIPDDQKVS[166.9984]DDDKEK	-7.29	6.0E-14	3.31	1.7E-04	-2.21	8.0E-03
MA7D1_HUMAN	MAP7 domain-containing protein 1 OS=Homo sapiens GN=MAP7D1 PE=1 SV=1	RSS[166.9984]QPSPTAVPASDSPPTK	1.12	8.7E-01	-1.00	9.1E-01	1.11	9.8E-01
MAP1B_HUMAN	Microtubule-associated protein 1B OS=Homo sapiens GN=MAP1B PE=1 SV=1	KES[166.9984]KEETPEVTK	2.00	4.7E-01	1.14	9.2E-01	2.29	2.4E-01
MAP1B_HUMAN	Microtubule-associated protein 1B OS=Homo sapiens GN=MAP1B PE=1 SV=1	KLGDVS[166.9984]PTQIDVSQFGSFK	1.99	4.7E-01	-1.22	9.3E-01	1.64	5.5E-01
MAP1B_HUMAN	Microtubule-associated protein 1B OS=Homo sapiens GN=MAP1B PE=1 SV=1	SPSLSPSPPS[166.9984]PLEK	1.68	7.1E-01	-1.40	8.3E-01	1.20	9.0E-01
MAP4_HUMAN	Microtubule-associated protein 4 - Homo sapiens (Human)	DM[147.0354]ES[166.9984]PTKLDVTLAK	3.80	1.5E-01	1.50	9.7E-01	5.68	6.4E-02
MAP4_HUMAN	Microtubule-associated protein 4 - Homo sapiens (Human)	SKVGS[166.9984]TENIK	3.32	1.9E-02	1.37	5.6E-01	4.55	6.2E-06
MAP4_HUMAN	Microtubule-associated protein 4 - Homo sapiens (Human)	VGS[166.9984]LDNVGHLPAGGAVK	4.53	2.5E-01	-1.30	9.4E-01	3.49	5.4E-02
MARCS_HUMAN	Myristoylated alanine-rich C-kinase substrate - Homo sapiens (Human)	AEDGATPSPSNET[181.014]PK	1.84	4.5E-01	1.75	2.4E-01	3.23	2.0E-03
MARCS_HUMAN	Myristoylated alanine-rich C-kinase substrate - Homo sapiens (Human)	AEDGATPSPSNET[181.014]PKK	1.61	2.0E-07	1.62	2.9E-02	2.61	1.8E-01
MARCS_HUMAN	Myristoylated alanine-rich C-kinase substrate - Homo sapiens (Human)	EAPAEGEAAEPGS[166.9984]PTAAEGEAASAASSTSSPK	2.15	3.2E-01	-1.00	8.6E-01	2.15	4.4E-02
MARCS_HUMAN	Myristoylated alanine-rich C-kinase substrate - Homo sapiens (Human)	GEPAAAAAPEAGAS[166.9984]PVEK	1.89	2.4E-01	-1.27	7.4E-01	1.50	2.9E-01
MARCS_HUMAN	Myristoylated alanine-rich C-kinase substrate - Homo sapiens (Human)	LSGFS[166.9984]FK	-2.16	4.5E-01	2.66	6.1E-02	1.23	7.7E-01
MARCS_HUMAN	Myristoylated alanine-rich C-kinase substrate - Homo sapiens (Human)	LSGFS[166.9984]FKK	-1.99	3.1E-01	1.39	6.3E-01	-1.43	7.5E-01
MARE2_HUMAN	Microtubule-associated protein RP/EB family member 2 OS=Homo sapiens GN=MAPRE2 PE=1 SV=1	SSPAAKPGSTPS[166.9984]RPS[166.9984]SAKR	1.48	8.4E-01	-2.20	4.9E-01	-1.48	6.6E-01
MATR3_HUMAN	Matrin-3 - Homo sapiens (Human)	RDS[166.9984]FDDRGPSLNPVLDYDHGSR	2.61	6.0E-03	-1.11	2.5E-04	2.36	7.5E-02
MATR3_HUMAN	Matrin-3 - Homo sapiens (Human)	SYS[166.9984]PDGKESPSDKK	-2.99	7.8E-02	1.78	5.8E-01	-1.68	6.1E-01
MECP2_HUMAN	Methyl-CpG-binding protein 2 OS=Homo sapiens GN=MECP2 PE=1 SV=1	AETSEGSGSAPAVPEASAS[166.9984]PK	1.72	2.1E-01	-1.64	3.5E-01	1.05	8.9E-01
MEGF8_HUMAN	Multiple epidermal growth factor-like domains 8 OS=Homo sapiens GN=MEGF8 PE=1 SV=1	S[166.9984]FHAAAYVPAGRGAM[147.0354]YLL GGLT[181.014]AGGVT[181.014]RDFWVLNLTTLQWR	-1.61	7.1E-01	-1.13	7.1E-01	-1.81	5.0E-01
MICA3_HUMAN	Protein MICAL-3 OS=Homo sapiens GN=MICAL3 PE=1 SV=1	Y[243.0297]M[147.0354]ATQLLAK	-1.17	9.6E-01	1.74	7.8E-01	1.48	8.5E-01
MLRV_HUMAN	Myosin regulatory light chain 2, ventricular/cardiac muscle isoform OS=Homo sapiens GN=MYL2 PE=1 SV=3	AGGANS[166.9984]NVFSM[147.0354]FEQTQIQEFK	2.86	3.4E-01	-1.86	7.5E-01	1.53	4.1E-01
MLRV_HUMAN	Myosin regulatory light chain 2, ventricular/cardiac muscle isoform OS=Homo sapiens GN=MYL2 PE=1 SV=3	AGGANS[166.9984]NVFSMFEQTQIQEFK	3.01	2.0E-01	-1.98	5.5E-01	1.52	7.0E-01
MLRV_HUMAN	Myosin regulatory light chain 2, ventricular/cardiac muscle isoform OS=Homo sapiens GN=MYL2 PE=1 SV=3	RAGGANS[166.9984]NVFSM[147.0354]FEQTQIQEFK	-2.29	3.5E-01	1.88	5.6E-01	-1.22	8.8E-01
MLRV_HUMAN	Myosin regulatory light chain 2, ventricular/cardiac muscle isoform OS=Homo sapiens GN=MYL2 PE=1 SV=3	RAGGANS[166.9984]NVFSMFEQTQIQEFK	-3.53	6.0E-03	1.70	7.8E-01	-2.08	8.8E-02
MPRI_HUMAN	Cation-independent mannose-6-phosphate receptor OS=Homo sapiens GN=IGF2R PE=1 SV=2	ALSS[166.9984]LHGDDQDSEDEVLTIPEVK	1.18	8.2E-01	-1.04	9.7E-01	1.14	8.1E-01
MPRI_HUMAN	Cation-independent mannose-6-phosphate receptor OS=Homo sapiens GN=IGF2R PE=1 SV=2	ALSSLHGDDQDS[166.9984]EDEVLTIPEVK	-1.02	9.8E-01	-1.34	6.5E-01	-1.36	5.4E-01
MPRI_HUMAN	Cation-independent mannose-6-phosphate receptor OS=Homo sapiens GN=IGF2R PE=1 SV=2	LVS[166.9984]FHDDS[166.9984]DEDLLHI	1.30	5.3E-01	-2.01	1.0E-02	-1.55	2.9E-01
MPRI_HUMAN	Cation-independent mannose-6-phosphate receptor OS=Homo sapiens GN=IGF2R PE=1 SV=2	LVSFHDDS[166.9984]DEDLLHI	1.08	9.0E-01	-1.71	1.9E-01	-1.58	2.5E-01
MPRI_HUMAN	Cation-independent mannose-6-phosphate receptor OS=Homo sapiens GN=IGF2R PE=1 SV=2	S[166.9984]LQLSTEGFITLTY[243.0297]K	-2.64	1.2E-02	1.03	9.4E-01	-2.58	3.6E-02
MRLC2_HUMAN	Myosin regulatory light chain MRLC2 OS=Homo sapiens GN=MYLC2B PE=1 SV=2	AT[181.014]SNVFAM[147.0354]FDQSQIQEFK	4.47	4.2E-01	-6.46	1.9E-01	-1.45	5.8E-01
MRLC2_HUMAN	Myosin regulatory light chain MRLC2 OS=Homo sapiens GN=MYLC2B PE=1 SV=2	AT[181.014]SNVFAMFDQSQIQEFK	3.22	3.5E-01	-6.30	3.9E-02	-1.96	1.3E-01
MRLC2_HUMAN	Myosin regulatory light chain MRLC2 OS=Homo sapiens GN=MYLC2B PE=1 SV=2	ATS[166.9984]NVFAM[147.0354]FDQSQIQEFK	-2.19	5.9E-01	1.04	7.8E-01	-2.11	5.0E-01
MTA2_HUMAN	Metastasis-associated protein MTA2 OS=Homo sapiens GN=MTA2 PE=1 SV=1	RPY[243.0297]APINANAIKAEC[160.0307]SIR	-1.01	9.5E-01	-1.44	6.8E-01	-1.45	8.2E-01
MUC5B_HUMAN	Mucin-5B OS=Homo sapiens GN=MUC5B PE=1 SV=2	ATSSSSPRT[181.014]AT[181.014]T[181.014]LPVLT[181.014]STATK	1.57	8.4E-01	3.31	7.0E-02	5.21	4.0E-03
MYCT1_HUMAN	Myc target protein 1 OS=Homo sapiens GN=MYCT1 PE=2 SV=1	SS[166.9984]YTHGLNR	1.19	7.0E-01	2.49	2.1E-01	2.95	6.6E-02
MYH14_HUMAN	Myosin-14 - Homo sapiens (Human)	QLVS[166.9984]TLEK	1.07	9.9E-01	2.24	5.8E-01	2.40	5.2E-01
MYH15_HUMAN	Myosin-15 OS=Homo sapiens GN=MYH15 PE=1 SV=3	EKRT[181.014]TEHK	-1.02	9.2E-01	-3.25	5.6E-02	-3.33	2.0E-01
MYOZ2_HUMAN	Myozenin-2 OS=Homo sapiens GN=MYOZ2 PE=1 SV=1	DIMLEELSHLS[166.9984]NR	-1.53	1.8E-01	3.02	2.5E-04	1.97	3.0E-03
MYOZ2_HUMAN	Myozenin-2 OS=Homo sapiens GN=MYOZ2 PE=1 SV=1	S[166.9984]PPNPDNIAPGYSGPLK	1.60	8.5E-01	-1.22	1.0E+00	1.31	8.2E-01
MYOZ2_HUMAN	Myozenin-2 OS=Homo sapiens GN=MYOZ2 PE=1 SV=1	VDGSNLEGGS[166.9984]QQAPLTPPNTPDPR	-1.26	9.5E-01	1.55	1.8E-01	1.23	4.3E-01
MYOZ2_HUMAN	Myozenin-2 OS=Homo sapiens GN=MYOZ2 PE=1 SV=1	VDGSNLEGGSQQAPLT[181.014]PPNT[181.014]PDPR	1.20	5.4E-01	-1.48	2.4E-01	-1.23	4.5E-01
MYOZ2_HUMAN	Myozenin-2 OS=Homo sapiens GN=MYOZ2 PE=1 SV=1	VDGSNLEGGSQQAPLT[181.014]PPNTPDPR	-1.03	9.8E-01	-1.24	4.5E-01	-1.28	2.2E-01
MYPC3_HUMAN	Myosin-binding protein C, cardiac-type OS=Homo sapiens GN=MYBPC3 PE=1 SV=3	IS[166.9984]DSHEDTGILDFSSLLK	1.26	9.2E-01	-1.26	8.2E-01	1.00	8.2E-01
MYPC3_HUMAN	Myosin-binding protein C, cardiac-type OS=Homo sapiens GN=MYBPC3 PE=1 SV=3	RIS[166.9984]DSHEDTGILDFSSLLK	-1.07	9.4E-01	-1.10	9.9E-01	-1.18	9.1E-01
MYPT1_HUMAN	Protein phosphatase 1 regulatory subunit 12A OS=Homo sapiens GN=PPP1R12A PE=1 SV=1	KTGS[166.9984]YGALAEITASK	1.41	7.0E-01	1.31	6.5E-01	1.84	1.9E-01
MYPT1_HUMAN	Protein phosphatase 1 regulatory subunit 12A OS=Homo sapiens GN=PPP1R12A PE=1 SV=1	KTGSY[243.0297]GALAEITASK	1.21	7.8E-01	1.21	9.1E-01	1.46	6.7E-01

MYPT1_HUMAN	Protein phosphatase 1 regulatory subunit 12A OS=Homo sapiens GN=PPP1R12A PE=1 SV=1	RS[166.9984]TQGVTLTDLQEAEK	-1.02	8.9E-01	1.28	7.5E-01	1.25	8.1E-01
MYPT1_HUMAN	Protein phosphatase 1 regulatory subunit 12A OS=Homo sapiens GN=PPP1R12A PE=1 SV=1	RST[181.014]QGVTLTDLQEAEK	-1.92	3.2E-01	1.20	8.2E-01	-1.60	4.3E-01
MYPT1_HUMAN	Protein phosphatase 1 regulatory subunit 12A OS=Homo sapiens GN=PPP1R12A PE=1 SV=1	S[166.9984]YLTPVRDEESESQR	-2.68	2.6E-02	1.25	6.5E-01	-2.15	1.1E-01
MYPT2_HUMAN	Protein phosphatase 1 regulatory subunit 12B OS=Homo sapiens GN=PPP1R12B PE=1 SV=2	DEDET[181.014]DGSEEVKETWHER	2.24	5.9E-01	-3.41	1.7E-01	-1.52	3.4E-01
MYPT2_HUMAN	Protein phosphatase 1 regulatory subunit 12B OS=Homo sapiens GN=PPP1R12B PE=1 SV=2	S[166.9984]LDEEPIC[160.0307]HR	-2.20	1.1E-01	-1.37	6.5E-01	-3.03	4.0E-03
NCAM1_HUMAN	Neural cell adhesion molecule 1 OS=Homo sapiens GN=NCAM1 PE=1 SV=3	AAFSKDES[166.9984]KEPIVEVR	-1.09	9.0E-01	-1.66	5.8E-01	-1.81	3.9E-01
NCK1_HUMAN	Cytoplasmic protein NCK1 OS=Homo sapiens GN=NCK1 PE=1 SV=1	RKPS[166.9984]VPDSASPADDSFVDPGER	-1.20	8.5E-01	1.22	8.2E-01	1.01	9.8E-01
NDK8_HUMAN	Putative nucleoside diphosphate kinase - Homo sapiens (Human)	VMLGET[181.014]NPADSKPGTIR	-1.66	6.3E-01	1.23	9.2E-01	-1.36	8.2E-01
NDRG1_HUMAN	Protein NDRG1 OS=Homo sapiens GN=NDRG1 PE=1 SV=1	SHT[181.014]SEGAHLDITPNSGAAGNSAGPK	1.39	7.1E-01	1.11	9.2E-01	1.54	5.5E-01
NDRG3_HUMAN	Protein NDRG3 OS=Homo sapiens GN=NDRG3 PE=1 SV=2	C[160.0307]S[166.9984]TLLVVGDNSPAVEAVVEC[160.0307]NS[166.9984]R	1.53	7.3E-01	-1.34	8.5E-01	1.15	8.8E-01
NEBL_HUMAN	Nebulette OS=Homo sapiens GN=NEBL PE=1 SV=1	TDPGS[166.9984]IFDLDPLEDNIQSR	1.58	7.1E-01	1.75	6.5E-01	2.78	1.8E-01
NEUG_HUMAN	Neurogranin OS=Homo sapiens GN=NRGN PE=1 SV=1	IQAS[166.9984]FR	1.27	8.7E-01	1.20	5.1E-01	1.52	6.8E-01
NEXN_HUMAN	Nexilin OS=Homo sapiens GN=NEXN PE=1 SV=1	EM[147.0354]LAS[166.9984]DDEEDVSSK	2.79	1.4E-01	-1.15	9.2E-01	2.42	2.0E-03
NEXN_HUMAN	Nexilin OS=Homo sapiens GN=NEXN PE=1 SV=1	EM[147.0354]LAS[166.9984]DDEEDVSSKVEK	5.55	1.5E-01	-5.85	1.8E-01	-1.05	7.2E-01
NEXN_HUMAN	Nexilin OS=Homo sapiens GN=NEXN PE=1 SV=1	TIS[166.9984]QEFLTPGK	13.46	5.3E-04	-16.05	3.1E-04	-1.19	6.9E-01
NFAC4_HUMAN	Nuclear factor of activated T-cells, cytoplasmic 4 OS=Homo sapiens GN=NFATC4 PE=1 SV=2	DPGS[166.9984]PGPFDYVGAPPAES[166.9984]IPQKT[181.014]RR	1.50	6.6E-01	-1.41	7.2E-01	1.06	9.7E-01
NFM_HUMAN	Neurofilament medium polypeptide OS=Homo sapiens GN=NEFM PE=1 SV=2	GKS[166.9984]PVPKS[166.9984]PVEEK	-1.61	4.6E-01	1.22	5.8E-01	-1.31	9.0E-01
NFYC_HUMAN	Nuclear transcription factor Y subunit gamma OS=Homo sapiens GN=NFYC PE=1 SV=3	S[166.9984]T[181.014]EGGFGGT[181.014]SSSDA QQSLQS[166.9984]FWPRVM[147.0354]EEIR	-2.73	9.4E-02	1.45	7.9E-01	-1.88	4.9E-01
NOC3L_HUMAN	Nucleolar complex protein 3 homolog OS=Homo sapiens GN=NOC3L PE=1 SV=1	LKLHT[181.014]ET[181.014]LNIVFVT[181.014]YFR	-1.73	1.8E-01	-1.03	7.4E-01	-1.78	1.3E-01
NOLC1_HUMAN	Nucleolar phosphoprotein p130 OS=Homo sapiens GN=NOLC1 PE=1 SV=2	GKGS[166.9984]PRPQAPK	1.70	8.5E-01	-4.87	1.3E-01	-2.86	4.3E-01
NOS1_HUMAN	Nitric oxide synthase, brain OS=Homo sapiens GN=NOS1 PE=1 SV=2	NTALGVIS[166.9984]NWTDELR	1.35	9.0E-01	1.87	3.5E-01	2.52	1.2E-01
NP1L4_HUMAN	Nucleosome assembly protein 1-like 4 OS=Homo sapiens GN=NAP1L4 PE=1 SV=1	EFITGDVEPTDAESEWHS[166.9984]ENEEEEK	-1.12	5.7E-01	1.44	3.9E-01	1.28	5.9E-01
NP1L4_HUMAN	Nucleosome assembly protein 1-like 4 OS=Homo sapiens GN=NAP1L4 PE=1 SV=1	EFITGDVEPTDAESEWHS[166.9984]ENEEEEKLAGDMK	-1.46	8.8E-01	1.36	7.7E-01	-1.07	9.6E-01
NSF1C_HUMAN	NSFL1 cofactor p47 OS=Homo sapiens GN=NSFL1C PE=1 SV=2	KKS[166.9984]PNELVDDLFK	1.36	8.5E-01	1.03	8.7E-01	1.39	4.8E-01
NUCB1_HUMAN	Nucleobindin-1 OS=Homo sapiens GN=NUCB1 PE=1 SV=4	AQRLS[166.9984]QETEALGR	1.78	2.1E-01	-1.60	4.3E-01	1.11	9.0E-01
NUCKS_HUMAN	Nuclear ubiquitous casein and cyclin-dependent kinases substrate OS=Homo sapiens GN=NUCKS1 PE=1 SV=1	KVVDYSQFQES[166.9984]DDADEDYGR	-1.07	9.1E-01	-1.33	6.4E-01	-1.42	3.3E-01
NUCKS_HUMAN	Nuclear ubiquitous casein and cyclin-dependent kinases substrate OS=Homo sapiens GN=NUCKS1 PE=1 SV=1	TPSPKEEDEEPES[166.9984]PPEKK	-1.33	2.3E-01	-1.41	7.8E-01	-1.89	1.1E-02
OCAD1_HUMAN	OCIA domain-containing protein 1 OS=Homo sapiens GN=OCIAD1 PE=1 SV=1	RSS[166.9984]PPGHYYQK	3.33	1.4E-02	-3.33	5.1E-02	-1.00	9.1E-01
ODBA_HUMAN	2-oxoisovalerate dehydrogenase subunit alpha, mitochondrial OS=Homo sapiens GN=BCKDHA PE=1 SV=2	IGHHS[166.9984]TSDDSSAY[243.0297]RSVDEVNYWDK	-1.22	8.7E-01	1.68	3.6E-01	1.38	6.8E-01
ODBA_HUMAN	2-oxoisovalerate dehydrogenase subunit alpha, mitochondrial OS=Homo sapiens GN=BCKDHA PE=1 SV=2	IGHHS[166.9984]TSDDSSAYR	-2.64	3.9E-02	3.07	2.2E-01	1.16	9.6E-01
ODBA_HUMAN	2-oxoisovalerate dehydrogenase subunit alpha, mitochondrial OS=Homo sapiens GN=BCKDHA PE=1 SV=2	IGHHS[166.9984]TSDDSSAYRS[166.9984]VDEVNYWDK	1.07	8.9E-01	1.08	1.0E+00	1.15	9.1E-01
ODBA_HUMAN	2-oxoisovalerate dehydrogenase subunit alpha, mitochondrial OS=Homo sapiens GN=BCKDHA PE=1 SV=2	IGHHSTSDDS[166.9984]SAY[243.0297]RSVDEVNYWDKQDHPISR	-1.36	8.3E-01	1.52	6.5E-01	1.11	9.1E-01
ODBA_HUMAN	2-oxoisovalerate dehydrogenase subunit alpha, mitochondrial OS=Homo sapiens GN=BCKDHA PE=1 SV=2	S[166.9984]VDEVNYWDK	-1.52	7.7E-01	1.68	6.9E-01	1.10	9.5E-01
ODBA_HUMAN	2-oxoisovalerate dehydrogenase subunit alpha, mitochondrial OS=Homo sapiens GN=BCKDHA PE=1 SV=2	S[166.9984]VDEVNYWDKQDHPISR	1.19	9.1E-01	-2.72	3.1E-01	-2.29	3.3E-01
ODPA_HUMAN	ODPA_HUMAN	YGM[147.0354]GT[181.014]SVER	123.00	6.0E-14	-61.57	8.9E-14	2.00	7.0E-01
ODPA_HUMAN	Pyruvate dehydrogenase E1 component subunit alpha, somatic form, mitochondrial OS=Homo sapiens GN=PDHA1 PE=1 SV=3	YHGHS[166.9984]M[147.0354]SDPGVS[166.9984]YR	1.16	5.7E-01	-1.15	6.5E-01	1.01	9.4E-01
ODPA_HUMAN	Pyruvate dehydrogenase E1 component subunit alpha, somatic form, mitochondrial OS=Homo sapiens GN=PDHA1 PE=1 SV=3	YHGHS[166.9984]M[147.0354]SDPGVSY[243.0297]R	32.46	1.8E-10	-32.49	2.3E-11	-1.00	9.4E-01
ODPA_HUMAN	Pyruvate dehydrogenase E1 component subunit alpha, somatic form, mitochondrial OS=Homo sapiens GN=PDHA1 PE=1 SV=3	YHGHS[166.9984]M[147.0354]SDPGVSYR	-1.64	9.0E-01	1.56	8.4E-01	-1.05	9.7E-01
ODPA_HUMAN	Pyruvate dehydrogenase E1 component subunit alpha, somatic form, mitochondrial OS=Homo sapiens GN=PDHA1 PE=1 SV=3	YHGHS[166.9984]MSDPGVSYR	25.87	1.1E-13	-27.30	6.0E-14	-1.06	9.1E-01
ODPA_HUMAN	Pyruvate dehydrogenase E1 component subunit alpha, somatic form, mitochondrial OS=Homo sapiens GN=PDHA1 PE=1 SV=3	YHGHSMS[166.9984]DPGVSYR	-2.20	5.9E-01	1.89	6.4E-01	-1.17	9.5E-01
OR5K3_HUMAN	Olfactory receptor 5K3 OS=Homo sapiens GN=OR5K3 PE=2 SV=1	LHTPM[147.0354]Y[243.0297]IFLGNLVLMDS[166.9984]C[160.0307] C[160.0307]S[166.9984]S[166.9984]AIT[181.014]PKM[147.0354]LENFFSEDKR	-1.79	1.3E-01	1.37	4.9E-01	-1.31	7.0E-01
P3C2A_HUMAN	Phosphatidylinositol-4-phosphate 3-kinase C2 domain-containing alpha polypeptide OS=Homo sapiens GN=PIK3C2A PE=1 SV=2	TKFPY[243.0297]TNHRT[181.014]NPGY[243.0297]LLSPVTAQR	-1.83	4.6E-01	5.97	4.5E-07	3.27	2.0E-02
P3C2G_HUMAN	Phosphatidylinositol-4-phosphate 3-kinase C2 domain-containing gamma polypeptide OS=Homo sapiens GN=PIK3C2G PE=1 SV=1	DLNHY[243.0297]MEQILNVSHEVTNSDC[160.0307]VLSF FLS[166.9984]EAVQQT[181.014]VES[166.9984]SPVYLGEK	-1.06	9.2E-01	1.39	7.8E-01	1.31	8.3E-01
PACN3_HUMAN	Protein kinase C and casein kinase substrate in neurons protein 3 OS=Homo sapiens GN=PACSIN3 PE=1 SV=2	DGTAPPPQSPGSPGTGQDEEWS[166.9984]DEESPRK	1.38	5.7E-01	-1.02	9.6E-01	1.35	6.0E-01
PACN3_HUMAN	Protein kinase C and casein kinase substrate in neurons protein 3 OS=Homo sapiens GN=PACSIN3 PE=1 SV=2	GGRS[166.9984]PDEVTLTSIVPTR	-1.01	9.8E-01	1.13	9.5E-01	1.12	9.3E-01
PALMD_HUMAN	Palmdelphin OS=Homo sapiens GN=PALMD PE=1 SV=1	NS[166.9984]KSPTEYHEPVYANPFYRPTTPQR	1.22	8.0E-01	-1.03	9.5E-01	1.18	8.7E-01
PALMD_HUMAN	Palmdelphin OS=Homo sapiens GN=PALMD PE=1 SV=1	SEHQNS[166.9984]SPTC[160.0307]QEDEEDVR	1.79	4.2E-01	-1.55	6.3E-01	1.15	8.2E-01
PALMD_HUMAN	Palmdelphin OS=Homo sapiens GN=PALMD PE=1 SV=1	SPTEYHEPVYANPFYRPTT[181.014]PQR	2.05	3.0E-02	-2.10	1.1E-01	-1.02	9.5E-01
PARD3_HUMAN	Partitioning-defective 3 homolog OS=Homo sapiens GN=PARD3 PE=1 SV=2	ENHADLGIFVKS[166.9984]IINGGAAS[166.9984]KDGR	1.55	6.0E-01	-1.46	6.7E-01	1.06	9.8E-01
PCDH1_HUMAN	Protocadherin-1 OS=Homo sapiens GN=PCDH1 PE=1 SV=2	SNSPLPSIQLQPQSPSAS[166.9984]KK	2.11	3.2E-01	-4.46	9.0E-03	-2.11	5.4E-02
PCDH7_HUMAN	Protocadherin-7 OS=Homo sapiens GN=PCDH7 PE=1 SV=1	LSDS[166.9984]PSM[147.0354]GR	6.23	4.1E-02	-8.23	1.4E-01	-1.32	9.0E-01
PCNP_HUMAN	PEST proteolytic signal-containing nuclear protein OS=Homo sapiens GN=PCNP PE=1 SV=2	NIGRDT[181.014]PTSAGPNSFNK	1.95	2.5E-01	-2.53	8.1E-02	-1.30	2.9E-01
PCY1A_HUMAN	Choline-phosphate cytidylyltransferase A OS=Homo sapiens GN=PCYT1A PE=1 SV=2	SPS[166.9984]PSFR	-1.65	6.7E-01	2.27	3.0E-01	1.37	7.6E-01
PCY1A_HUMAN	Choline-phosphate cytidylyltransferase A OS=Homo sapiens GN=PCYT1A PE=1 SV=2	T[181.014]SPPC[160.0307]SPANLSR	-1.01	9.1E-01	-1.43	7.0E-01	-1.44	4.7E-01
PCY1B_HUMAN	Choline-phosphate cytidylyltransferase B OS=Homo sapiens GN=PCYT1B PE=1 SV=1	MLQALS[166.9984]PK	1.11	9.1E-01	1.50	7.4E-01	1.66	6.0E-01
PDCD4_HUMAN	Programmed cell death protein 4 OS=Homo sapiens GN=PDCD4 PE=1 SV=1	SGLTVPTS[166.9984]PK	2.02	4.7E-01	1.11	9.4E-01	2.23	2.8E-01
PDE1C_HUMAN	Calcium/calmodulin-dependent 3',5'-cyclic nucleotide phosphodiesterase 1C OS=Homo sapiens GN=PDE1C PE=2 SV=1	RSS[166.9984]LNSISSSDAK	1.10	9.7E-01	1.14	9.1E-01	1.26	8.7E-01
PDE3A_HUMAN	cGMP-inhibited 3',5'-cyclic phosphodiesterase A OS=Homo sapiens GN=PDE3A PE=1 SV=3	RTS[166.9984]LPC[160.0307]IPR	1.79	5.7E-01	1.40	8.5E-01	2.51	2.9E-01
PDLI2_HUMAN	PDZ and LIM domain protein 2 OS=Homo sapiens GN=PDLIM2 PE=1 SV=1	SSRPSM[147.0354]DSEGGS[166.9984]LLLDEDSEVFKMLQENR	1.18	7.7E-01	-1.29	6.8E-01	-1.10	9.6E-01
PEBP1_HUMAN	Phosphatidylethanolamine-binding protein 1 - Homo sapiens (Human)	NRPTS[166.9984]ISWDGLDSGK	-1.00	1.0E+00	-1.14	7.4E-01	-1.14	7.3E-01
PEBP1_HUMAN	Phosphatidylethanolamine-binding protein 1 - Homo sapiens (Human)	NRPTSIS[166.9984]WDGLDSGK	-1.01	9.8E-01	-1.96	3.2E-01	-1.99	1.5E-01
PGM1_HUMAN	Phosphoglucomutase-1 OS=Homo sapiens GN=PGM1 PE=1 SV=3	AIGGIILTAS[166.9984]HNPGGPNGDFGIK	-1.21	9.0E-01	-1.70	6.2E-01	-2.06	2.8E-01
PGM2_HUMAN	Phosphoglucomutase-2 OS=Homo sapiens GN=PGM2 PE=1 SV=4	KAVLPTS[166.9984]K	1.74	5.5E-01	1.25	7.0E-01	2.16	1.1E-01
PGM2_HUMAN	Phosphoglucomutase-2 OS=Homo sapiens GN=PGM2 PE=1 SV=4	SALKDTY[243.0297]M[147.0354]LSST[181.014]VS[166.9984]SKILR	1.45	6.9E-01	-1.51	7.0E-01	-1.04	9.9E-01
PGM52_HUMAN	Putative PGM5-like protein 2 OS=Homo sapiens PE=5 SV=2	AAGGIILTAS[166.9984]HC[160.0307]PGGPGGEFGVK	1.15	9.3E-01	-1.17	9.4E-01	-1.02	9.8E-01
PGRC1_HUMAN	Membrane-associated progesterone receptor component 1 - Homo sapiens (Human)	EGEEPTVYS[166.9984]DEEEPKDESAR	2.22	5.9E-02	-1.43	7.1E-01	1.55	1.0E-01
PGRC1_HUMAN	Membrane-associated progesterone receptor component 1 - Homo sapiens (Human)	LLKEGEEPTVYS[166.9984]DEEEPKDESAR	1.33	3.7E-01	1.09	9.4E-01	1.45	2.7E-01
PGRC2_HUMAN	Membrane-associated progesterone receptor component 2 - Homo sapiens (Human)	LLKPGEEPS[166.9984]EYTDEEDTKDHNKQD	1.32	1.6E-01	-1.22	4.0E-01	1.08	9.3E-01
PGRC2_HUMAN	Membrane-associated progesterone receptor component 2 - Homo sapiens (Human)	LLKPGEEPSEYT[181.014]DEEDTK	-1.17	8.7E-01	1.25	8.0E-01	1.07	9.5E-01
PGRC2_HUMAN	Membrane-associated progesterone receptor component 2 - Homo sapiens (Human)	LLKPGEEPSEYT[181.014]DEEDTKDHNK	1.15	4.1E-01	-1.13	5.9E-01	1.02	9.8E-01
PGRC2_HUMAN	Membrane-associated progesterone receptor component 2 - Homo sapiens (Human)	LLKPGEEPSEYT[181.014]DEEDTKDHNKQD	-1.53	4.5E-01	1.46	6.3E-01	-1.05	9.0E-01
PHF6_HUMAN	PHD finger protein 6 OS=Homo sapiens GN=PHF6 PE=1 SV=1	DRS[166.9984]PHRS[166.9984]SPSDTRPK	1.04	8.9E-01	1.06	9.1E-01	1.11	9.8E-01
PININ_HUMAN	Pinin - Homo sapiens (Human)	SLS[166.9984]PGKENVSALDM[147.0354]EK	2.16	7.0E-01	-3.54	3.9E-01	-1.64	5.6E-01
PKP2_HUMAN	Plakophilin-2 OS=Homo sapiens GN=PKP2 PE=1 SV=1	RLEIS[166.9984]PDS[166.9984]SPER	1.50	3.9E-01	-1.09	8.4E-01	1.37	5.0E-01
PLCL1_HUMAN	Inactive phospholipase C-like protein 1 OS=Homo sapiens GN=PLCL1 PE=1 SV=2	KTVSFS[166.9984]SMPSEKK	2.00	2.9E-01	-1.13	9.0E-01	1.76	6.6E-01
PLEC1_HUMAN	Plectin-1 - Homo sapiens (Human)	DPYSGS[166.9984]TISLFQAM[147.0354]QK	1.32	7.3E-01	-2.78	8.3E-01	-2.11	3.1E-01
PLM_HUMAN	Phospholemman OS=Homo sapiens GN=FXYD1 PE=1 SV=2	TGEPDEEEGTFRSS[166.9984]IR	1.40	8.1E-01	-2.44	2.0E-01	-1.75	3.3E-01
PMGT1_HUMAN	Protein O-linked-mannose beta-1,2-N-acetylglucosaminyltransferase 1 OS=Homo sapiens GN=POMGNT1 PE=1 SV=1	NPC[160.0307]EDSFLPDTEGHTYVAFIRM[147.0354]EKDDDFT[181.014]T[181.014]WTQLAK	1.17	8.9E-01	-1.87	1.2E-01	-1.59	1.4E-01
POLH_HUMAN	DNA polymerase eta OS=Homo sapiens GN=POLH PE=1 SV=1	RLSS[166.9984]LR	-1.04	8.6E-01	-1.59	5.7E-01	-1.65	2.1E-01
POPD1_HUMAN	Blood vessel epicardial substance OS=Homo sapiens GN=BVES PE=2 SV=1	GTSS[166.9984]MS[166.9984]SLHVSSPHQR	2.59	5.4E-01	-2.02	7.0E-01	1.28	8.9E-01
POPD1_HUMAN	Blood vessel epicardial substance OS=Homo sapiens GN=BVES PE=2 SV=1	GTSSM[147.0354]S[166.9984]SLHVSSPHQR	1.41	5.6E-01	-1.71	3.9E-01	-1.21	7.2E-01
POPD1_HUMAN	Blood vessel epicardial substance OS=Homo sapiens GN=BVES PE=2 SV=1	GTSSMSS[166.9984]LHVSSPHQR	-1.69	7.8E-01	1.37	9.2E-01	-1.24	9.1E-01
POPD1_HUMAN	Blood vessel epicardial substance OS=Homo sapiens GN=BVES PE=2 SV=1	M[147.0354]KPIEEGAEDDDDVFEPAS[166.9984]PNTLK	1.59	5.8E-01	-2.23	8.0E-02	-1.40	1.6E-01
POPD1_HUMAN	Blood vessel epicardial substance OS=Homo sapiens GN=BVES PE=2 SV=1	MKPIEEGAEDDDDVFEPAS[166.9984]PNTLK	1.68	9.4E-01	-2.65	7.5E-01	-1.58	7.5E-01
POPD1_HUMAN	Blood vessel epicardial substance OS=Homo sapiens GN=BVES PE=2 SV=1	NS[166.9984]IASSSDSDDGLHQFLR	-8.02	1.2E-02	2.03	9.4E-01	-3.96	1.7E-01
POPD1_HUMAN	Blood vessel epicardial substance OS=Homo sapiens GN=BVES PE=2 SV=1	NSIASSS[166.9984]DSDDGLHQFLR	-5.94	8.4E-02	1.35	7.0E-01	-4.40	1.3E-01
POPD1_HUMAN	Blood vessel epicardial substance OS=Homo sapiens GN=BVES PE=2 SV=1	NSIASSSDS[166.9984]DDGLHQFLR	-10.06	6.0E-14	1.30	1.0E+00	-7.72	7.9E-11
POPD2_HUMAN	Popeye domain-containing protein 2 OS=Homo sapiens GN=POPDC2 PE=2 SV=2	GQAPLAPTHT[181.014]PEL	-1.34	4.3E-01	3.21	5.0E-03	2.39	5.8E-02
POPD2_HUMAN	Popeye domain-containing protein 2 OS=Homo sapiens GN=POPDC2 PE=2 SV=2	IPLQSYS[166.9984]QVISR	1.62	4.4E-01	-1.78	3.1E-01	-1.10	9.0E-01
POPD2_HUMAN	Popeye domain-containing protein 2 OS=Homo sapiens GN=POPDC2 PE=2 SV=2	LSRPDS[166.9984]GILASR	-1.42	8.0E-01	1.06	1.0E+00	-1.34	8.3E-01
PP16A_HUMAN	Protein phosphatase 1 regulatory subunit 16A OS=Homo sapiens GN=PPP1R16A PE=1 SV=1	M[147.0354]S[166.9984]TQERLKHAQK	-2.81	4.1E-02	1.22	7.0E-01	-2.30	2.4E-01
PPHLN_HUMAN	Periphilin-1 OS=Homo sapiens GN=PPHLN1 PE=1 SV=2	DTS[166.9984]PSSGSAVSSSK	1.13	8.0E-01	1.09	9.1E-01	1.24	8.9E-01
PPLA_HUMAN	Cardiac phospholamban OS=Homo sapiens GN=PLN PE=1 SV=1	RAS[166.9984]TIEMPQQAR	2.25	8.4E-01	-1.13	8.9E-01	2.00	5.6E-01
PPLA_HUMAN	Cardiac phospholamban OS=Homo sapiens GN=PLN PE=1 SV=1	RAST[181.014]IEM[147.0354]PQQAR	-2.59	2.5E-01	-1.24	6.1E-01	-3.21	6.6E-02
PPR1C_HUMAN	Protein phosphatase 1 regulatory subunit 1C OS=Homo sapiens GN=PPP1R1C PE=2 SV=1	RGPNTQGELQNAS[166.9984]PK	-1.16	9.1E-01	1.23	9.5E-01	1.07	9.6E-01
PR38B_HUMAN	Pre-mRNA-splicing factor 38B OS=Homo sapiens GN=PRPF38B PE=1 SV=1	RRS[166.9984]QS[166.9984]IEQESQEK	-1.16	9.2E-01	1.00	9.5E-01	-1.16	8.8E-01
PRKRA_HUMAN	Interferon-inducible double stranded RNA-dependent protein kinase activator A OS=Homo sapiens GN=PRKRA PE=1 SV=1	EDS[166.9984]GTFSLGK	1.63	7.0E-01	-1.08	9.5E-01	1.51	7.3E-01
PRKRA_HUMAN	Interferon-inducible double stranded RNA-dependent protein kinase activator A OS=Homo sapiens GN=PRKRA PE=1 SV=1	HRAEAPPLEREDS[166.9984]GTFSLGK	-1.56	6.6E-01	1.20	8.2E-01	-1.29	8.3E-01
PRP4B_HUMAN	Serine/threonine-protein kinase PRP4 homolog OS=Homo sapiens GN=PRPF4B PE=1 SV=2	KKS[166.9984]PIINESR	1.40	9.8E-01	-1.09	8.5E-01	1.29	7.7E-01
PRP4B_HUMAN	Serine/threonine-protein kinase PRP4 homolog OS=Homo sapiens GN=PRPF4B PE=1 SV=2	RRS[166.9984]LS[166.9984]PKPR	1.98	5.0E-01	-2.35	3.2E-01	-1.19	6.8E-01
PRP4B_HUMAN	Serine/threonine-protein kinase PRP4 homolog OS=Homo sapiens GN=PRPF4B PE=1 SV=2	S[166.9984]RS[166.9984]PLLNDR	1.03	9.2E-01	-1.31	6.2E-01	-1.27	7.6E-01
PRP4B_HUMAN	Serine/threonine-protein kinase PRP4 homolog OS=Homo sapiens GN=PRPF4B PE=1 SV=2	S[166.9984]RS[166.9984]PLLNDRR	1.03	9.4E-01	-1.55	6.5E-01	-1.51	5.6E-01
PSA3_HUMAN	Proteasome subunit alpha type-3 - Homo sapiens (Human)	ESLKEEDES[166.9984]DDDNM	1.41	9.0E-01	-1.64	9.8E-01	-1.17	8.2E-01
PSA3_HUMAN	Proteasome subunit alpha type-3 - Homo sapiens (Human)	ESLKEEDES[166.9984]DDDNM[147.0354]	-1.96	9.4E-01	1.26	8.0E-01	-1.56	8.2E-01
PTN13_HUMAN	Tyrosine-protein phosphatase non-receptor type 13 OS=Homo sapiens GN=PTPN13 PE=1 SV=2	AIST[181.014]GSLASS[166.9984]TLNK	2.22	4.0E-03	1.01	9.2E-01	2.24	3.0E-02
PTPRG_HUMAN	Receptor-type tyrosine-protein phosphatase gamma OS=Homo sapiens GN=PTPRG PE=1 SV=3	VGLAPLPGM[147.0354]KGT[181.014]DYI NAS[166.9984]Y[243.0297]IMGYY[243.0297]R	1.39	5.5E-01	-1.02	1.0E+00	1.36	5.0E-01
PTRF_HUMAN	Polymerase I and transcript release factor OS=Homo sapiens GN=PTRF PE=1 SV=1	ES[166.9984]EALPEKEGEELGEGERPEEDAAALELS[166.9984]SDEAVEVEEVIEESR	1.08	8.9E-01	1.54	2.7E-01	1.67	1.1E-01
PTRF_HUMAN	Polymerase I and transcript release factor OS=Homo sapiens GN=PTRF PE=1 SV=1	ES[166.9984]EALPEKEGEELGEGERPEEDAAALELSS[166.9984]DEAVEVEEVIEESR	1.53	5.2E-01	-1.10	9.2E-01	1.38	5.8E-01
PTRF_HUMAN	Polymerase I and transcript release factor OS=Homo sapiens GN=PTRF PE=1 SV=1	KVS[166.9984]VNVK	-1.25	8.2E-01	1.39	7.0E-01	1.11	9.1E-01
PTRF_HUMAN	Polymerase I and transcript release factor OS=Homo sapiens GN=PTRF PE=1 SV=1	LPAKLS[166.9984]ISK	1.27	1.3E-01	-1.18	6.5E-01	1.08	8.6E-01
PTRF_HUMAN	Polymerase I and transcript release factor OS=Homo sapiens GN=PTRF PE=1 SV=1	RGS[166.9984]S[166.9984]PDVHALLEITEESDAVLVDK	2.28	6.3E-01	-2.22	6.5E-01	1.03	9.2E-01
PTRF_HUMAN	Polymerase I and transcript release factor OS=Homo sapiens GN=PTRF PE=1 SV=1	S[166.9984]FTPDHVVYAR	-1.12	1.0E+00	1.12	8.6E-01	-1.00	9.0E-01
PTRF_HUMAN	Polymerase I and transcript release factor OS=Homo sapiens GN=PTRF PE=1 SV=1	S[166.9984]LKESEALPEK	-1.04	9.8E-01	-1.03	9.7E-01	-1.06	1.0E+00
PTRF_HUMAN	Polymerase I and transcript release factor OS=Homo sapiens GN=PTRF PE=1 SV=1	VM[147.0354]IYQDEVKLPAKLSIS[166.9984]K	2.15	2.2E-01	-2.38	2.3E-01	-1.11	9.4E-01
PTRFL_HUMAN	PTRF/SDPR family protein OS=Homo sapiens PE=2 SV=2	EIPT[181.014]PEPLK	9.33	1.3E-12	-2.60	1.2E-02	3.59	1.4E-01
PTRFL_HUMAN	PTRF/SDPR family protein OS=Homo sapiens PE=2 SV=2	LS[166.9984]SVTEDEDQDAALTIVTVLDK	-1.67	5.7E-01	3.68	1.3E-07	2.20	2.7E-02
PTRFL_HUMAN	PTRF/SDPR family protein OS=Homo sapiens PE=2 SV=2	S[166.9984]ESLGPISELYSDELSEPEHEAARPVYPPHEGR	-1.20	9.6E-01	2.34	5.2E-02	1.94	1.4E-01
PTRFL_HUMAN	PTRF/SDPR family protein OS=Homo sapiens PE=2 SV=2	S[166.9984]GKEHIDNIK	-1.44	6.4E-01	2.00	4.2E-02	1.39	4.2E-01
PTRFL_HUMAN	PTRF/SDPR family protein OS=Homo sapiens PE=2 SV=2	S[166.9984]GKEHIDNIKK	-1.41	6.4E-01	1.89	2.7E-02	1.33	4.4E-01
PTRFL_HUMAN	PTRF/SDPR family protein OS=Homo sapiens PE=2 SV=2	SES[166.9984]LGPISELYSDELS[166.9984]EPEHEAARPVYPPHEGR	-1.07	9.4E-01	-1.31	7.2E-01	-1.39	6.8E-01
PTRFL_HUMAN	PTRF/SDPR family protein OS=Homo sapiens PE=2 SV=2	SES[166.9984]LGPISELYSDELSEPEHEAARPVYPPHEGR	-1.58	6.6E-01	-1.27	8.5E-01	-2.01	4.9E-01
PXDC2_HUMAN	Plexin domain-containing protein 2 OS=Homo sapiens GN=PLXDC2 PE=1 SV=1	RGS[166.9984]GHPAYAEVEPVGEK	1.36	8.4E-01	3.15	4.2E-02	4.28	7.0E-03
QCR6_HUMAN	Cytochrome b-c1 complex subunit 6, mitochondrial precursor - Homo sapiens (Human)	S[166.9984]HTEEDC[160.0307]TEELFDFLHAR	-1.38	8.8E-01	-1.30	8.2E-01	-1.80	6.9E-01
QCR6_HUMAN	Cytochrome b-c1 complex subunit 6, mitochondrial precursor - Homo sapiens (Human)	SHT[181.014]EEDC[160.0307]TEELFDFLHAR	-1.08	9.8E-01	-1.81	4.6E-01	-1.95	7.0E-01
QSOX2_HUMAN	Sulfhydryl oxidase 2 OS=Homo sapiens GN=QSOX2 PE=2 SV=3	QHYGRDNLLDTYSADQGDS[166.9984]SEGGTLARGEEEEK	1.70	7.3E-01	-1.03	9.1E-01	1.66	4.3E-01

RA1L3_HUMAN	Putative heterogeneous nuclear ribonucleoprotein A1-like protein 3 OS=Homo sapiens GN=HNRPA1L3 PE=5 SV=1	SES[166.9984]PKEPEQLR	2.48	2.9E-01	-1.62	6.8E-01	1.53	4.3E-01
RA1L3_HUMAN	Putative heterogeneous nuclear ribonucleoprotein A1-like protein 3 OS=Homo sapiens GN=HNRPA1L3 PE=5 SV=1	SES[166.9984]PKEPEQLRK	1.37	8.2E-02	-1.14	5.8E-01	1.20	2.9E-01
RAD_HUMAN	GTP-binding protein RAD OS=Homo sapiens GN=RRAD PE=1 SV=2	RGS[166.9984]TPWGPAPPLHR	1.89	2.3E-02	-1.51	3.4E-01	1.25	7.3E-01
RAD_HUMAN	GTP-binding protein RAD OS=Homo sapiens GN=RRAD PE=1 SV=2	RRGST[181.014]PWGPAPPLHR	1.85	9.3E-01	-1.58	8.5E-01	1.17	7.4E-01
RAD_HUMAN	GTP-binding protein RAD OS=Homo sapiens GN=RRAD PE=1 SV=2	SKS[166.9984]C[160.0307]HDLSVL	1.04	6.4E-01	1.04	9.2E-01	1.08	4.8E-01
RALY_HUMAN	RNA-binding protein Raly OS=Homo sapiens GN=RALY PE=1 SV=1	GRLS[166.9984]PVPVPR	2.58	2.2E-01	-1.77	5.0E-01	1.45	6.7E-01
RBBP6_HUMAN	Retinoblastoma-binding protein 6 - Homo sapiens (Human)	LEVTEIVKPS[166.9984]PK	1.57	4.7E-01	-1.67	4.4E-01	-1.06	8.8E-01
RBM10_HUMAN	RNA-binding protein 10 OS=Homo sapiens GN=RBM10 PE=1 SV=3	LASDDRPS[166.9984]PPR	2.08	2.6E-01	-1.31	8.0E-01	1.59	6.3E-01
RBM25_HUMAN	Probable RNA-binding protein 25 OS=Homo sapiens GN=RBM25 PE=1 SV=2	LGASNS[166.9984]PGQPNSVK	2.27	6.8E-02	-1.56	4.9E-01	1.46	7.2E-01
RBM39_HUMAN	RNA-binding protein 39 - Homo sapiens (Human)	DKS[166.9984]PVREPIDNLTPEER	1.57	5.2E-01	-1.29	7.5E-01	1.22	6.9E-01
RBP1_HUMAN	RalA-binding protein 1 OS=Homo sapiens GN=RALBP1 PE=1 SV=3	AGKEPAKPS[166.9984]PSR	2.30	1.7E-01	1.22	6.2E-01	2.81	2.1E-02
RCAS1_HUMAN	Receptor-binding cancer antigen expressed on SiSo cells OS=Homo sapiens GN=EBAG9 PE=1 SV=1	KLSGDQIT[181.014]LPTTVDYSSVPK	1.23	6.3E-01	-1.80	8.5E-02	-1.46	4.3E-01
RFPL1_HUMAN	Ret finger protein-like 1 OS=Homo sapiens GN=RFPL1 PE=2 SV=2	FQVDMTLDADT[181.014]ANNFLLISDDLR	1.72	8.6E-01	-2.33	6.3E-01	-1.36	5.5E-01
RHG22_HUMAN	Rho GTPase-activating protein 22 OS=Homo sapiens GN=ARHGAP22 PE=2 SV=1	S[166.9984]LDLDHSM[147.0354]DEAGAGASNSEPSEPDSPTREHAR	1.03	9.7E-01	1.22	7.9E-01	1.25	7.1E-01
RIC3_HUMAN	Protein RIC-3 OS=Homo sapiens GN=RIC3 PE=1 SV=1	SHLAEAFAKAKGS[166.9984]GGGAGGGGS[166.9984]GR	-1.11	8.4E-01	1.24	8.1E-01	1.11	9.6E-01
RL1D1_HUMAN	Ribosomal L1 domain-containing protein 1 OS=Homo sapiens GN=RSL1D1 PE=1 SV=3	AT[181.014]NESEDEIPQLVPIGK	1.21	7.5E-01	-1.23	7.0E-01	-1.01	9.8E-01
RL1D1_HUMAN	Ribosomal L1 domain-containing protein 1 OS=Homo sapiens GN=RSL1D1 PE=1 SV=3	ATNES[166.9984]EDEIPQLVPIGK	1.49	3.7E-01	-1.58	3.4E-01	-1.06	9.5E-01
RLA2_HUMAN	60S acidic ribosomal protein P2 OS=Homo sapiens GN=RPLP2 PE=1 SV=1	KEES[166.9984]EES[166.9984]DDDM[147.0354]GFGLFD	3.57	5.3E-01	-5.06	3.0E-01	-1.42	8.5E-01
RLA2_HUMAN	60S acidic ribosomal protein P2 OS=Homo sapiens GN=RPLP2 PE=1 SV=1	KEES[166.9984]EES[166.9984]DDDMGFGLFD	-3.84	1.1E-01	1.35	9.4E-01	-2.84	3.8E-01
RMP_HUMAN	Unconventional prefoldin RPB5 interactor OS=Homo sapiens GN=RMP PE=1 SV=2	KNS[166.9984]TGSGHSAQELPTIR	-1.30	8.3E-01	-1.03	9.5E-01	-1.35	7.5E-01
RNPS1_HUMAN	RNA-binding protein with serine-rich domain 1 OS=Homo sapiens GN=RNPS1 PE=1 SV=1	RFS[166.9984]PPR	1.43	6.7E-01	-1.21	8.0E-01	1.18	9.5E-01
ROA3_HUMAN	Heterogeneous nuclear ribonucleoprotein A3 - Homo sapiens (Human)	SSGS[166.9984]PYGGGYGSGGGSGGYGSR	1.53	4.9E-01	-1.19	8.0E-01	1.29	7.4E-01
RP1_HUMAN	Oxygen-regulated protein 1 OS=Homo sapiens GN=RP1 PE=1 SV=1	S[166.9984]VIGS[166.9984]VTLVSETEVQEK	-1.57	1.2E-01	-1.45	8.0E-01	-2.28	7.0E-03
RRAS2_HUMAN	Ras-related protein R-Ras2 OS=Homo sapiens GN=RRAS2 PE=1 SV=1	KFQEQEC[160.0307]PPS[166.9984]PEPTR	1.68	2.2E-01	1.26	9.0E-01	2.11	3.7E-01
RRAS2_HUMAN	Ras-related protein R-Ras2 OS=Homo sapiens GN=RRAS2 PE=1 SV=1	KFQEQEC[160.0307]PPS[166.9984]PEPTRK	-1.05	9.2E-01	1.92	4.2E-01	1.83	5.6E-01
RSRC2_HUMAN	Arginine/serine-rich coiled-coil protein 2 OS=Homo sapiens GN=RSRC2 PE=1 SV=1	EQSEVSVS[166.9984]PR	1.39	6.6E-01	-1.56	4.5E-01	-1.12	8.2E-01
RTKN_HUMAN	Rhotekin OS=Homo sapiens GN=RTKN PE=1 SV=2	LSSSLGRS[166.9984]SGR	1.31	7.1E-01	1.23	9.6E-01	1.61	6.8E-01
RTN1_HUMAN	Reticulon-1 OS=Homo sapiens GN=RTN1 PE=1 SV=1	DTDIS[166.9984]IKPEGVR	2.91	3.6E-04	-3.14	4.6E-04	-1.08	9.6E-01
RU17_HUMAN	U1 small nuclear ribonucleoprotein 70 kDa - Homo sapiens (Human)	YDERPGPS[166.9984]PLPHR	2.17	2.8E-01	-1.30	7.9E-01	1.68	3.3E-01
S12A4_HUMAN	Solute carrier family 12 member 4 OS=Homo sapiens GN=SLC12A4 PE=1 SV=2	M[147.0354]HTAVKLNEVIVTRS[166.9984]HDAR	1.16	9.2E-01	1.55	3.1E-05	1.81	8.0E-03
S38A1_HUMAN	Sodium-coupled neutral amino acid transporter 1 OS=Homo sapiens GN=SLC38A1 PE=1 SV=1	RS[166.9984]LTNSHLEK	-1.00	9.8E-01	-1.51	7.5E-01	-1.51	8.2E-01
SAFB1_HUMAN	Scaffold attachment factor B1 OS=Homo sapiens GN=SAFB PE=1 SV=4	SVVS[166.9984]FDK	1.93	2.5E-01	-1.73	3.4E-01	1.11	9.0E-01
SAFB1_HUMAN	Scaffold attachment factor B1 OS=Homo sapiens GN=SAFB PE=1 SV=4	SVVS[166.9984]FDKVK	1.43	8.0E-01	-1.32	8.2E-01	1.08	9.1E-01
SC61B_HUMAN	Protein transport protein Sec61 subunit beta OS=Homo sapiens GN=SEC61B PE=1 SV=2	PGPTPS[166.9984]GTNVGSSGRSPSK	1.46	4.4E-01	-1.73	2.3E-01	-1.19	3.3E-01
SC61B_HUMAN	Protein transport protein Sec61 subunit beta OS=Homo sapiens GN=SEC61B PE=1 SV=2	PGPTPSGTNVGSSGRS[166.9984]PSK	1.81	4.4E-01	-2.24	2.4E-01	-1.24	4.9E-01
SDPR_HUMAN	Serum deprivation-response protein OS=Homo sapiens GN=SDPR PE=1 SV=3	EELPDENKSLEETLHT[181.014]VDLS[166.9984]SDDDLPHDEEALEDSAEEKVEESR	1.39	9.8E-01	1.21	2.7E-01	1.68	1.9E-01
SDPR_HUMAN	Serum deprivation-response protein OS=Homo sapiens GN=SDPR PE=1 SV=3	IS[166.9984]S[166.9984]GKS[166.9984]SPFKVSPLTFGR	1.06	9.3E-01	1.31	3.2E-01	1.39	2.9E-01
SDPR_HUMAN	Serum deprivation-response protein OS=Homo sapiens GN=SDPR PE=1 SV=3	ISSGKS[166.9984]S[166.9984]PFKVS[166.9984]PLTFGR	1.26	1.0E+00	1.01	8.9E-01	1.27	8.7E-01
SDPR_HUMAN	Serum deprivation-response protein OS=Homo sapiens GN=SDPR PE=1 SV=3	S[166.9984]SPFKVS[166.9984]PLTFGR	1.45	6.5E-01	-1.21	8.6E-01	1.20	7.3E-01
SDPR_HUMAN	Serum deprivation-response protein OS=Homo sapiens GN=SDPR PE=1 SV=3	SLEETLHTVDLS[166.9984]S[166.9984]DDDLPHDEEALEDS[166.9984]AEEKVEESR	1.42	8.3E-01	-1.22	9.2E-01	1.16	9.0E-01
SDPR_HUMAN	Serum deprivation-response protein OS=Homo sapiens GN=SDPR PE=1 SV=3	SLEETLHTVDLS[166.9984]S[166.9984]DDDLPHDEEALEDSAEEK	1.04	9.2E-01	1.10	8.2E-01	1.14	7.7E-01
SDPR_HUMAN	Serum deprivation-response protein OS=Homo sapiens GN=SDPR PE=1 SV=3	SLEETLHTVDLS[166.9984]S[166.9984]DDDLPHDEEALEDSAEEKVEESR	-1.12	9.1E-01	1.21	6.3E-01	1.08	8.3E-01
SDPR_HUMAN	Serum deprivation-response protein OS=Homo sapiens GN=SDPR PE=1 SV=3	SLEETLHTVDLSS[166.9984]DDDLPHDEEALEDS[166.9984]AEEKVEESR	-1.02	9.5E-01	-1.12	1.0E+00	-1.13	9.4E-01
SDPR_HUMAN	Serum deprivation-response protein OS=Homo sapiens GN=SDPR PE=1 SV=3	SSPFKVS[166.9984]PLTFGR	-1.65	6.3E-01	1.40	3.6E-01	-1.17	9.9E-01
SDPR_HUMAN	Serum deprivation-response protein OS=Homo sapiens GN=SDPR PE=1 SV=3	VS[166.9984]PLTFGR	1.46	5.8E-01	-2.42	8.2E-02	-1.66	5.7E-01
SEC62_HUMAN	Translocation protein SEC62 OS=Homo sapiens GN=SEC62 PE=1 SV=1	VGPGNHGTEGSGGERHS[166.9984]DTDSDR	-1.49	7.7E-01	2.27	5.5E-01	1.52	7.3E-01
SEPT2_HUMAN	Septin-2 - Homo sapiens (Human)	IYHLPDAES[166.9984]DEDEDFKEQTR	1.05	8.0E-01	1.67	4.2E-02	1.75	7.0E-03
SFRS1_HUMAN	Splicing factor, arginine/serine-rich 1 - Homo sapiens (Human)	VDGPRS[166.9984]PS[166.9984]YGR	1.15	9.1E-01	-1.25	7.0E-01	-1.09	6.9E-01
SFRS1_HUMAN	Splicing factor, arginine/serine-rich 1 - Homo sapiens (Human)	VDGPRS[166.9984]PS[166.9984]YGRS[166.9984]R	1.30	8.2E-01	-1.57	5.7E-01	-1.20	8.4E-01
SFRS1_HUMAN	Splicing factor, arginine/serine-rich 1 - Homo sapiens (Human)	VDGPRS[166.9984]PSYGR	2.46	2.3E-01	-3.25	5.8E-02	-1.32	7.8E-01
SFRS1_HUMAN	Splicing factor, arginine/serine-rich 1 - Homo sapiens (Human)	VKVDGPRS[166.9984]PS[166.9984]YGR	1.34	4.3E-01	-1.82	1.0E-01	-1.36	3.1E-01
SFRS1_HUMAN	Splicing factor, arginine/serine-rich 1 - Homo sapiens (Human)	VKVDGPRS[166.9984]PSY[243.0297]GR	-1.36	6.7E-01	-1.38	8.6E-01	-1.88	3.8E-01
SFRS6_HUMAN	Splicing factor, arginine/serine-rich 6 - Homo sapiens (Human)	ARS[166.9984]VS[166.9984]PPPK	1.18	2.9E-06	-2.11	5.6E-09	-1.78	4.6E-01
SFRS6_HUMAN	Splicing factor, arginine/serine-rich 6 - Homo sapiens (Human)	ARS[166.9984]VS[166.9984]PPPKR	1.07	9.2E-01	-2.47	5.8E-01	-2.30	3.7E-01
SFRS9_HUMAN	Splicing factor, arginine/serine-rich 9 - Homo sapiens (Human)	GS[166.9984]PHYFSPFRPY	1.29	7.0E-01	-1.17	8.0E-01	1.10	9.0E-01
SGCA_HUMAN	Alpha-sarcoglycan OS=Homo sapiens GN=SGCA PE=1 SV=1	LPPRVDS[166.9984]AQVPLILDQH	-1.25	7.8E-01	-1.05	9.4E-01	-1.31	6.8E-01
SH3R2_HUMAN	Putative E3 ubiquitin-protein ligase SH3RF2 OS=Homo sapiens GN=SH3RF2 PE=2 SV=2	FQNYS[166.9984]PPPTK	-1.25	7.1E-01	1.15	7.0E-01	-1.08	9.5E-01
SIDT2_HUMAN	SID1 transmembrane family member 2 OS=Homo sapiens GN=SIDT2 PE=1 SV=2	KDFPSNS[166.9984]FYVVVVVK	2.06	4.8E-01	-1.92	5.3E-01	1.07	9.0E-01
SLTM_HUMAN	SAFB-like transcription modulator OS=Homo sapiens GN=SLTM PE=1 SV=2	AGAGM[147.0354]ITQHSSNAS[166.9984]PINR	-1.01	9.4E-01	1.37	7.5E-01	1.35	7.0E-01
SLTM_HUMAN	SAFB-like transcription modulator OS=Homo sapiens GN=SLTM PE=1 SV=2	AGAGMITQHSSNAS[166.9984]PINR	-1.22	8.4E-01	1.21	8.2E-01	-1.01	9.9E-01
SLTM_HUMAN	SAFB-like transcription modulator OS=Homo sapiens GN=SLTM PE=1 SV=2	DGQDAIAQS[166.9984]PEK	1.75	7.1E-01	-1.92	6.5E-01	-1.10	9.1E-01
SLTM_HUMAN	SAFB-like transcription modulator OS=Homo sapiens GN=SLTM PE=1 SV=2	DGQDAIAQS[166.9984]PEKESK	1.21	8.9E-01	-1.59	5.9E-01	-1.31	6.6E-01
SLTM_HUMAN	SAFB-like transcription modulator OS=Homo sapiens GN=SLTM PE=1 SV=2	S[166.9984]PGHMVILDQTK	-1.00	9.8E-01	-1.32	6.5E-01	-1.32	6.8E-01
SMAP_HUMAN	Small acidic protein OS=Homo sapiens GN=SMAP PE=1 SV=1	S[166.9984]ASPDDDLGSSNWEAADLGNEER	-1.04	9.4E-01	-1.35	7.1E-01	-1.40	5.3E-01
SMAP_HUMAN	Small acidic protein OS=Homo sapiens GN=SMAP PE=1 SV=1	SAS[166.9984]PDDDLGSSNWEAADLGNEERK	-1.08	9.2E-01	-1.45	2.2E-01	-1.56	9.4E-02
SMCA2_HUMAN	Probable global transcription activator SNF2L2 OS=Homo sapiens GN=SMARCA2 PE=1 SV=1	GRPPAEKLS[166.9984]PNPPK	1.13	9.2E-01	-1.07	9.3E-01	1.05	9.8E-01
SMTN_HUMAN	Smoothelin OS=Homo sapiens GN=SMTN PE=1 SV=5	STS[166.9984]FGVPNANSIK	-2.04	1.1E-01	-1.36	7.0E-01	-2.78	1.0E-02
SN_HUMAN	Sialoadhesin OS=Homo sapiens GN=SIGLEC1 PE=1 SV=2	VVATSLPSGGGC[160.0307]ST[181.014]C[160.0307]G GC[160.0307]S[166.9984]PRM[147.0354]KVTK	-1.21	7.8E-01	-1.10	1.0E+00	-1.33	7.6E-01
SNPC4_HUMAN	snRNA-activating protein complex subunit 4 OS=Homo sapiens GN=SNAPC4 PE=1 SV=1	LASS[166.9984]RVER	-1.50	3.7E-01	2.43	6.1E-01	1.62	9.3E-01
SOX4_HUMAN	Transcription factor SOX-4 OS=Homo sapiens GN=SOX4 PE=1 SV=1	VGGS[166.9984]GGGGHGGGGGGGS[166.9984]S[166.9984]NAG GGGGGAS[166.9984]GGGANS[166.9984]KPAQK	-1.79	2.3E-01	-1.21	7.3E-01	-2.15	4.1E-02
SP100_HUMAN	Nuclear autoantigen Sp-100 OS=Homo sapiens GN=SP100 PE=1 SV=3	LNEC[160.0307]IS[166.9984]PVANEM[147.0354]NHLPAHSHDLQR	1.45	2.6E-01	1.17	7.7E-01	1.70	6.4E-02
SP100_HUMAN	Nuclear autoantigen Sp-100 OS=Homo sapiens GN=SP100 PE=1 SV=3	LNEC[160.0307]IS[166.9984]PVANEMNHLPAHSHDLQR	-1.12	1.0E+00	1.24	9.2E-01	1.11	9.3E-01
SP100_HUMAN	Nuclear autoantigen Sp-100 OS=Homo sapiens GN=SP100 PE=1 SV=3	LPLQES[166.9984]EEEEREER	3.42	2.9E-01	-3.39	3.3E-01	1.01	8.8E-01
SPATL_HUMAN	Protein SPATIAL OS=Homo sapiens GN=SPATIAL PE=2 SV=2	ELKELAS[166.9984]RVAFLT[181.014]K	-3.45	5.5E-04	2.33	2.1E-02	-1.48	6.3E-01
SPRL1_HUMAN	SPARC-like protein 1 OS=Homo sapiens GN=SPARCL1 PE=1 SV=1	AEDEENEKETAVS[166.9984]TEDDSHHK	1.12	9.2E-01	-1.29	7.5E-01	-1.15	8.5E-01
SPRL1_HUMAN	SPARC-like protein 1 OS=Homo sapiens GN=SPARCL1 PE=1 SV=1	DQGNQEQDPNIS[166.9984]NGEEEEEKEPGEVGTHNDNQER	1.05	9.8E-01	-1.29	6.9E-01	-1.23	6.7E-01
SPRL1_HUMAN	SPARC-like protein 1 OS=Homo sapiens GN=SPARCL1 PE=1 SV=1	HIQETEWQS[166.9984]QEGK	1.27	7.1E-01	-1.89	5.0E-01	-1.49	5.0E-01
SPRL1_HUMAN	SPARC-like protein 1 OS=Homo sapiens GN=SPARCL1 PE=1 SV=1	SSS[166.9984]QELGLK	-1.13	9.2E-01	-1.65	5.5E-01	-1.87	3.0E-01
SPTC2_HUMAN	Serine palmitoyltransferase 2 OS=Homo sapiens GN=SPTLC2 PE=1 SV=1	KELIDY[243.0297]LR	5.10	4.0E-03	-2.02	5.0E-01	2.52	4.6E-01
SRBS2_HUMAN	Sorbin and SH3 domain-containing protein 2 OS=Homo sapiens GN=SORBS2 PE=1 SV=3	DAS[166.9984]SPVPPPHVPPPVPPLRPR	2.67	3.3E-01	2.68	4.4E-01	7.16	1.8E-02
SRBS2_HUMAN	Sorbin and SH3 domain-containing protein 2 OS=Homo sapiens GN=SORBS2 PE=1 SV=3	DASS[166.9984]PVPPPHVPPPVPPLRPR	2.64	1.4E-02	2.64	2.4E-01	6.98	6.8E-04
SRBS2_HUMAN	Sorbin and SH3 domain-containing protein 2 OS=Homo sapiens GN=SORBS2 PE=1 SV=3	GAEDYPDPPIPHS[166.9984]YSSDR	3.70	4.8E-05	1.04	1.0E+00	3.85	4.2E-04
SRBS2_HUMAN	Sorbin and SH3 domain-containing protein 2 OS=Homo sapiens GN=SORBS2 PE=1 SV=3	RKS[166.9984]EPAVGPPR	2.26	4.0E-03	1.52	3.4E-01	3.44	2.6E-05
SRBS2_HUMAN	Sorbin and SH3 domain-containing protein 2 OS=Homo sapiens GN=SORBS2 PE=1 SV=3	S[166.9984]EPAVGPPR	5.55	3.7E-08	-1.69	2.9E-01	3.28	3.5E-02
SRBS2_HUMAN	Sorbin and SH3 domain-containing protein 2 OS=Homo sapiens GN=SORBS2 PE=1 SV=3	SFTSSS[166.9984]PS[166.9984]SPSR	3.15	1.0E-03	-1.07	8.4E-01	2.95	6.0E-03
SRBS2_HUMAN	Sorbin and SH3 domain-containing protein 2 OS=Homo sapiens GN=SORBS2 PE=1 SV=3	SFTSSSPS[166.9984]SPSR	1.86	8.1E-02	1.39	3.8E-01	2.60	2.0E-03
SRBS2_HUMAN	Sorbin and SH3 domain-containing protein 2 OS=Homo sapiens GN=SORBS2 PE=1 SV=3	SHS[166.9984]DNSPNAFK	1.77	2.6E-02	1.25	8.0E-01	2.21	3.6E-02
SRBS2_HUMAN	Sorbin and SH3 domain-containing protein 2 OS=Homo sapiens GN=SORBS2 PE=1 SV=3	T[181.014]SPGRVDLPGSSTTLTK	-2.16	1.9E-01	3.77	2.9E-04	1.75	3.7E-01
SRBS2_HUMAN	Sorbin and SH3 domain-containing protein 2 OS=Homo sapiens GN=SORBS2 PE=1 SV=3	TSPGRVDLPGS[166.9984]STTLTK	-1.20	9.2E-01	1.25	8.9E-01	1.04	9.8E-01
SRC8_HUMAN	Src substrate cortactin - Homo sapiens (Human)	AKT[181.014]QTPPVS[166.9984]PAPQPTEER	1.05	9.0E-01	2.36	4.8E-02	2.48	3.3E-02
SRC8_HUMAN	Src substrate cortactin - Homo sapiens (Human)	AKTQT[181.014]PPVSPAPQPTEER	-1.30	5.3E-01	2.30	3.0E-03	1.76	5.9E-02
SRC8_HUMAN	Src substrate cortactin - Homo sapiens (Human)	ASAGHAVS[166.9984]IAQDDAGADDWETDPDFVNDVSEK	1.44	8.0E-01	1.11	8.8E-01	1.60	5.5E-01
SRC8_HUMAN	Src substrate cortactin - Homo sapiens (Human)	HC[160.0307]S[166.9984]QVDSVR	2.82	1.7E-02	-1.87	3.2E-01	1.51	4.0E-01
SRC8_HUMAN	Src substrate cortactin - Homo sapiens (Human)	LPS[166.9984]SPVYEDAASFK	1.63	2.9E-01	-1.08	9.2E-01	1.50	3.6E-01
SRC8_HUMAN	Src substrate cortactin - Homo sapiens (Human)	LPSS[166.9984]PVYEDAASFK	-1.14	7.2E-01	1.58	4.5E-01	1.39	5.8E-01
SRC8_HUMAN	Src substrate cortactin - Homo sapiens (Human)	T[181.014]QTPPVS[166.9984]PAPQPTEER	-1.61	2.6E-01	1.90	2.8E-01	1.18	8.8E-01
SRC8_HUMAN	Src substrate cortactin - Homo sapiens (Human)	TQT[181.014]PPVS[166.9984]PAPQPTEER	-5.46	6.8E-01	4.31	6.7E-01	-1.27	8.7E-01
SRC8_HUMAN	Src substrate cortactin - Homo sapiens (Human)	TQT[181.014]PPVSPAPQPTEER	-2.60	6.4E-02	1.47	6.8E-01	-1.77	4.5E-01
SRCH_HUMAN	Sarcoplasmic reticulum histidine-rich calcium-binding protein OS=Homo sapiens GN=HRC PE=2 SV=1	AEVGAPLS[166.9984]PDHS[166.9984]EEEEEEEEGLEEDEPR	-1.04	9.4E-01	1.68	7.6E-01	1.63	7.2E-01
SRCH_HUMAN	Sarcoplasmic reticulum histidine-rich calcium-binding protein OS=Homo sapiens GN=HRC PE=2 SV=1	AEVGAPLS[166.9984]PDHSEEEEEEEEGLEEDEPR	1.23	8.0E-01	-1.04	8.4E-01	1.18	9.5E-01
SRCH_HUMAN	Sarcoplasmic reticulum histidine-rich calcium-binding protein OS=Homo sapiens GN=HRC PE=2 SV=1	AEVGAPLSPDHS[166.9984]EEEEEEEEGLEEDEPR	1.81	2.2E-01	-1.65	3.7E-01	1.10	7.7E-01
SRCH_HUMAN	Sarcoplasmic reticulum histidine-rich calcium-binding protein OS=Homo sapiens GN=HRC PE=2 SV=1	DDSEEEKEKEEDPGS[166.9984]HEEDDESSEQGEK	1.18	1.7E-01	-1.34	2.2E-01	-1.13	4.9E-01
SRCH_HUMAN	Sarcoplasmic reticulum histidine-rich calcium-binding protein OS=Homo sapiens GN=HRC PE=2 SV=1	DEEEDEDVS[166.9984]TER	-1.26	4.4E-01	1.09	9.8E-01	-1.16	4.5E-01
SRCH_HUMAN	Sarcoplasmic reticulum histidine-rich calcium-binding protein OS=Homo sapiens GN=HRC PE=2 SV=1	EEAGGASS[166.9984]EEESGEDTGPQDAQEY GNYQPGSLC[160.0307]GYC[160.0307]SFC[160.0307]NR	-1.16	5.7E-01	-1.00	8.5E-01	-1.16	3.9E-01
SRCH_HUMAN	Sarcoplasmic reticulum histidine-rich calcium-binding protein OS=Homo sapiens GN=HRC PE=2 SV=1	EEDEEVS[166.9984]AELGHQAPSHR	-1.11	8.1E-01	-1.11	6.4E-01	-1.24	5.8E-01
SRCH_HUMAN	Sarcoplasmic reticulum histidine-rich calcium-binding protein OS=Homo sapiens GN=HRC PE=2 SV=1	EKEEDPGS[166.9984]HEEDDESSEQGEK	-1.07	1.0E+00	-1.16	4.7E-01	-1.25	6.9E-01
SRCH_HUMAN	Sarcoplasmic reticulum histidine-rich calcium-binding protein OS=Homo sapiens GN=HRC PE=2 SV=1	GHDGEDDEGEEEEEEEEEEEEAS[166.9984]TEYGHQAHR	-1.32	6.7E-01	-1.02	9.6E-01	-1.34	7.0E-01
SRCH_HUMAN	Sarcoplasmic reticulum histidine-rich calcium-binding protein OS=Homo sapiens GN=HRC PE=2 SV=1	GHGS[166.9984]EEDEDVSDGHHHHGPSHR	-1.30	8.0E-01	-1.11	9.2E-01	-1.44	6.6E-01
SRCH_HUMAN	Sarcoplasmic reticulum histidine-rich calcium-binding protein OS=Homo sapiens GN=HRC PE=2 SV=1	GHGSEDT[181.014]EDSAEHR	-1.73	5.8E-01	1.09	6.3E-01	-1.58	3.7E-01
SRCH_HUMAN	Sarcoplasmic reticulum histidine-rich calcium-binding protein OS=Homo sapiens GN=HRC PE=2 SV=1	GHGSEDTEDS[166.9984]AEHR	-1.88	1.7E-01	1.17	8.5E-01	-1.61	3.3E-01
SRCH_HUMAN	Sarcoplasmic reticulum histidine-rich calcium-binding protein OS=Homo sapiens GN=HRC PE=2 SV=1	GHKS[166.9984]DEEDFQDEYK	-1.92	4.5E-01	1.17	9.7E-01	-1.64	4.3E-01
SRCH_HUMAN	Sarcoplasmic reticulum histidine-rich calcium-binding protein OS=Homo sapiens GN=HRC PE=2 SV=1	HQGHEEDDDDDDDDDDDDDDDDVS[166.9984]IEYR	-1.37	4.4E-01	-1.20	8.1E-01	-1.64	2.2E-01
SRCH_HUMAN	Sarcoplasmic reticulum histidine-rich calcium-binding protein OS=Homo sapiens GN=HRC PE=2 SV=1	HQGHRDEEEDEDVS[166.9984]TER	-2.20	7.0E-02	1.20	1.0E+00	-1.84	1.1E-01
SRCH_HUMAN	Sarcoplasmic reticulum histidine-rich calcium-binding protein OS=Homo sapiens GN=HRC PE=2 SV=1	HRS[166.9984]HEEDDNDDDDVS[166.9984]TEYGHQAHR	-3.18	4.0E-03	1.65	4.9E-01	-1.92	3.1E-01
SRCH_HUMAN	Sarcoplasmic reticulum histidine-rich calcium-binding protein OS=Homo sapiens GN=HRC PE=2 SV=1	HRS[166.9984]HEEDDNDDDDVSTEYGHQAHR	-1.31	6.3E-01	-2.26	7.0E-03	-2.97	3.0E-03
SRCH_HUMAN	Sarcoplasmic reticulum histidine-rich calcium-binding protein OS=Homo sapiens GN=HRC PE=2 SV=1	SHEEDDNDDDDVS[166.9984]TEYGHQAHR	-1.28	7.5E-01	-2.61	6.5E-01	-3.34	1.9E-01
SRCH_HUMAN	Sarcoplasmic reticulum histidine-rich calcium-binding protein OS=Homo sapiens GN=HRC PE=2 SV=1	VGDEGVS[166.9984]GEEVFAEHGGQAR	-2.57	2.8E-02	-1.36	6.7E-01	-3.50	4.0E-03
SRCH_HUMAN	Sarcoplasmic reticulum histidine-rich calcium-binding protein OS=Homo sapiens GN=HRC PE=2 SV=1	VPREEDEEVS[166.9984]AELGHQAPSHR	-1.66	1.0E+00	-2.62	9.2E-07	-4.36	1.1E-05
SRRM1_HUMAN	Serine/arginine repetitive matrix protein 1 - Homo sapiens (Human)	AAS[166.9984]PS[166.9984]PQSVR	1.28	7.1E-01	-1.24	7.5E-01	1.04	9.2E-01
SRRM1_HUMAN	Serine/arginine repetitive matrix protein 1 - Homo sapiens (Human)	AAS[166.9984]PSPQSVR	1.16	9.7E-01	-1.15	9.2E-01	1.01	9.1E-01
SRRM1_HUMAN	Serine/arginine repetitive matrix protein 1 - Homo sapiens (Human)	APQTSSS[166.9984]PPPVR	1.10	8.7E-01	-1.11	7.8E-01	-1.01	9.0E-01
SRRM1_HUMAN	Serine/arginine repetitive matrix protein 1 - Homo sapiens (Human)	HRPS[166.9984]PPAT[181.014]PPPK	1.11	9.2E-01	-1.14	8.5E-01	-1.02	9.4E-01
SRRM1_HUMAN	Serine/arginine repetitive matrix protein 1 - Homo sapiens (Human)	KEKT[181.014]PELPEPSVK	1.13	9.7E-01	-1.16	9.2E-01	-1.03	8.2E-01
SRRM1_HUMAN	Serine/arginine repetitive matrix protein 1 - Homo sapiens (Human)	KET[181.014]ESEAEDNLDDLEK	1.20	9.4E-01	-1.24	8.2E-01	-1.03	8.4E-01
SRRM1_HUMAN	Serine/arginine repetitive matrix protein 1 - Homo sapiens (Human)	KETES[166.9984]EAEDNLDDLEK	1.60	5.9E-01	-1.66	4.5E-01	-1.03	9.1E-01
SRRM1_HUMAN	Serine/arginine repetitive matrix protein 1 - Homo sapiens (Human)	KPPAPPS[166.9984]PVQSQS[166.9984]PSTNWSPAVPVK	1.46	7.0E-01	-1.57	6.4E-01	-1.08	8.9E-01
SRRM1_HUMAN	Serine/arginine repetitive matrix protein 1 - Homo sapiens (Human)	KPPAPPS[166.9984]PVQSQS[166.9984]PSTNWSPAVPVKK	1.39	8.7E-01	-1.53	7.2E-01	-1.10	7.9E-01
SRRM1_HUMAN	Serine/arginine repetitive matrix protein 1 - Homo sapiens (Human)	KS[166.9984]RVSVS[166.9984]PGR	-1.12	9.6E-01	1.00	9.4E-01	-1.12	9.1E-01
SRRM1_HUMAN	Serine/arginine repetitive matrix protein 1 - Homo sapiens (Human)	KVELS[166.9984]ES[166.9984]EEDKGGK	1.31	8.3E-01	-1.46	6.5E-01	-1.12	6.2E-01
SRRM1_HUMAN	Serine/arginine repetitive matrix protein 1 - Homo sapiens (Human)	KVELS[166.9984]ESEEDKGGK	2.10	4.6E-01	-2.39	2.7E-01	-1.13	9.1E-01
SRRM1_HUMAN	Serine/arginine repetitive matrix protein 1 - Homo sapiens (Human)	RES[166.9984]PS[166.9984]PAPKPR	1.25	7.5E-01	-1.44	5.6E-01	-1.15	7.8E-01
SRRM1_HUMAN	Serine/arginine repetitive matrix protein 1 - Homo sapiens (Human)	RES[166.9984]PSPAPKPR	1.06	9.4E-01	-1.31	5.4E-01	-1.23	5.6E-01
SRRM1_HUMAN	Serine/arginine repetitive matrix protein 1 - Homo sapiens (Human)	RESPS[166.9984]PAPKPR	1.05	1.0E+00	-1.30	5.6E-01	-1.24	4.2E-01
SRRM1_HUMAN	Serine/arginine repetitive matrix protein 1 - Homo sapiens (Human)	RLS[166.9984]PSAS[166.9984]PPR	1.19	9.2E-01	-1.49	5.0E-01	-1.25	3.6E-01
SRRM1_HUMAN	Serine/arginine repetitive matrix protein 1 - Homo sapiens (Human)	RQS[166.9984]PSPST[181.014]RPIR	1.54	6.9E-01	-1.93	3.3E-01	-1.25	4.9E-01
SRRM1_HUMAN	Serine/arginine repetitive matrix protein 1 - Homo sapiens (Human)	RRAS[166.9984]PS[166.9984]PPPK	1.07	8.0E-01	-1.35	8.1E-01	-1.26	2.5E-01
SRRM1_HUMAN	Serine/arginine repetitive matrix protein 1 - Homo sapiens (Human)	RRS[166.9984]PS[166.9984]PAPPPR	1.23	8.6E-01	-1.58	4.9E-01	-1.29	7.0E-01
SRRM1_HUMAN	Serine/arginine repetitive matrix protein 1 - Homo sapiens (Human)	RRS[166.9984]PS[166.9984]PPPTR	-1.06	8.9E-01	-1.22	8.2E-01	-1.29	6.2E-01
SRRM1_HUMAN	Serine/arginine repetitive matrix protein 1 - Homo sapiens (Human)	RRT[181.014]AS[166.9984]PPPPPK	1.42	9.2E-01	-1.87	6.4E-01	-1.31	5.4E-01

SRRM1_HUMAN	Serine/arginine repetitive matrix protein 1 - Homo sapiens (Human)	RRT[181.014]PS[166.9984]PPPR	-1.19	6.2E-01	-1.15	8.5E-01	-1.37	3.8E-01
SRRM1_HUMAN	Serine/arginine repetitive matrix protein 1 - Homo sapiens (Human)	RRT[181.014]PT[181.014]PPPR	-1.02	9.2E-01	-1.35	8.3E-01	-1.38	6.8E-01
SRRM1_HUMAN	Serine/arginine repetitive matrix protein 1 - Homo sapiens (Human)	RYS[166.9984]PS[166.9984]PPPKR	1.04	9.9E-01	-1.47	6.7E-01	-1.42	5.8E-01
SRRM1_HUMAN	Serine/arginine repetitive matrix protein 1 - Homo sapiens (Human)	S[166.9984]PS[166.9984]PAPPPR	-1.06	9.4E-01	-1.42	5.8E-01	-1.50	3.4E-01
SRRM1_HUMAN	Serine/arginine repetitive matrix protein 1 - Homo sapiens (Human)	SRVS[166.9984]VS[166.9984]PGR	-1.30	5.9E-01	-1.17	8.9E-01	-1.52	2.8E-01
SRRM1_HUMAN	Serine/arginine repetitive matrix protein 1 - Homo sapiens (Human)	TAS[166.9984]PPPPK	1.09	9.8E-01	-1.74	5.2E-01	-1.59	2.1E-01
SRRM1_HUMAN	Serine/arginine repetitive matrix protein 1 - Homo sapiens (Human)	VPKPEPIPEPKEPS[166.9984]PEK	1.19	9.2E-01	-3.03	8.5E-08	-2.54	8.8E-05
SRRM2_HUMAN	Serine/arginine repetitive matrix protein 2 - Homo sapiens (Human)	AQT[181.014]PPGPSLSGSK	1.19	6.4E-01	1.35	8.6E-01	1.60	6.8E-01
SRRM2_HUMAN	Serine/arginine repetitive matrix protein 2 - Homo sapiens (Human)	AQT[181.014]PPGPSLSGSKS[166.9984]PC[160.0307]PQEK	2.53	2.5E-01	-1.79	5.1E-01	1.41	7.3E-01
SRRM2_HUMAN	Serine/arginine repetitive matrix protein 2 - Homo sapiens (Human)	AQTPPGPSLS[166.9984]GSKSPC[160.0307]PQEK	1.43	6.3E-01	-1.12	8.1E-01	1.28	9.5E-01
SRRM2_HUMAN	Serine/arginine repetitive matrix protein 2 - Homo sapiens (Human)	GEFSAS[166.9984]PM[147.0354]LK	-1.55	5.4E-01	1.95	3.8E-01	1.26	8.9E-01
SRRM2_HUMAN	Serine/arginine repetitive matrix protein 2 - Homo sapiens (Human)	GEGDAPFSEPGTTSTQRPS[166.9984]SPETATK	1.04	9.8E-01	1.16	9.0E-01	1.21	8.8E-01
SRRM2_HUMAN	Serine/arginine repetitive matrix protein 2 - Homo sapiens (Human)	HAS[166.9984]S[166.9984]S[166.9984]PES[166.9984]PKPAPAPGSHR	-1.03	9.6E-01	1.14	9.2E-01	1.11	9.5E-01
SRRM2_HUMAN	Serine/arginine repetitive matrix protein 2 - Homo sapiens (Human)	HGGS[166.9984]PQPLATTPLSQEPVNPPSEASPT[181.014]R	2.24	5.2E-01	-2.03	5.8E-01	1.10	9.8E-01
SRRM2_HUMAN	Serine/arginine repetitive matrix protein 2 - Homo sapiens (Human)	NHS[166.9984]GSRT[181.014]PPVALNSSR	1.17	8.1E-01	-1.10	7.5E-01	1.06	8.7E-01
SRRM2_HUMAN	Serine/arginine repetitive matrix protein 2 - Homo sapiens (Human)	NHSGS[166.9984]RT[181.014]PPVALNSSR	1.24	9.0E-01	-1.17	9.2E-01	1.06	9.7E-01
SRRM2_HUMAN	Serine/arginine repetitive matrix protein 2 - Homo sapiens (Human)	RGEGDAPFSEPGTTSTQRPS[166.9984]SPETATK	1.37	4.5E-12	-1.32	1.7E-08	1.03	9.2E-01
SRRM2_HUMAN	Serine/arginine repetitive matrix protein 2 - Homo sapiens (Human)	RGEGDAPFSEPGTTSTQRPSS[166.9984]PETATK	1.25	6.9E-01	-1.22	6.7E-01	1.03	9.0E-01
SRRM2_HUMAN	Serine/arginine repetitive matrix protein 2 - Homo sapiens (Human)	RPS[166.9984]PQPSPR	-1.21	6.0E-01	1.25	7.6E-01	1.03	9.5E-01
SRRM2_HUMAN	Serine/arginine repetitive matrix protein 2 - Homo sapiens (Human)	RRPS[166.9984]PQPS[166.9984]PR	1.97	4.7E-01	-1.95	4.5E-01	1.01	9.1E-01
SRRM2_HUMAN	Serine/arginine repetitive matrix protein 2 - Homo sapiens (Human)	RRPS[166.9984]PQPSPR	1.07	8.9E-01	-1.06	8.0E-01	1.01	9.0E-01
SRRM2_HUMAN	Serine/arginine repetitive matrix protein 2 - Homo sapiens (Human)	RVPS[166.9984]PTPAPK	1.19	8.7E-01	-1.19	7.5E-01	1.00	9.0E-01
SRRM2_HUMAN	Serine/arginine repetitive matrix protein 2 - Homo sapiens (Human)	S[166.9984]RS[166.9984]PS[166.9984]SPELNNK	1.06	9.6E-01	-1.07	8.5E-01	-1.01	8.8E-01
SRRM2_HUMAN	Serine/arginine repetitive matrix protein 2 - Homo sapiens (Human)	SATRPS[166.9984]PS[166.9984]PER	-1.17	5.7E-01	1.13	9.4E-01	-1.04	6.9E-01
SRRM2_HUMAN	Serine/arginine repetitive matrix protein 2 - Homo sapiens (Human)	SC[160.0307]FESS[166.9984]PDPELK	-1.00	9.9E-01	-1.10	7.2E-01	-1.11	7.0E-01
SRRM2_HUMAN	Serine/arginine repetitive matrix protein 2 - Homo sapiens (Human)	SRT[181.014]PPSAPSQSR	1.07	9.4E-01	-1.20	6.3E-01	-1.13	7.0E-01
SRRM2_HUMAN	Serine/arginine repetitive matrix protein 2 - Homo sapiens (Human)	SSS[166.9984]PVTELASR	-1.21	6.9E-01	1.06	9.6E-01	-1.14	6.9E-01
SRRM2_HUMAN	Serine/arginine repetitive matrix protein 2 - Homo sapiens (Human)	SST[181.014]PPGESYFGVSSLQLK	1.33	7.8E-01	-1.60	5.9E-01	-1.20	8.7E-01
SRRM2_HUMAN	Serine/arginine repetitive matrix protein 2 - Homo sapiens (Human)	SSTPPGESY[243.0297]FGVSSLQLK	1.22	8.8E-01	-1.62	6.4E-01	-1.33	7.0E-01
SRRM2_HUMAN	Serine/arginine repetitive matrix protein 2 - Homo sapiens (Human)	THTTALAGRS[166.9984]PS[166.9984]PASGR	1.02	9.5E-01	-1.37	4.9E-01	-1.34	5.0E-01
SRRM2_HUMAN	Serine/arginine repetitive matrix protein 2 - Homo sapiens (Human)	THTTALAGRS[166.9984]PSPAS[166.9984]GR	1.12	9.1E-01	-1.61	4.5E-01	-1.44	6.2E-01
SRRM2_HUMAN	Serine/arginine repetitive matrix protein 2 - Homo sapiens (Human)	THTTALAGRS[166.9984]PSPAS[166.9984]GRR	-1.35	5.9E-01	-1.17	8.6E-01	-1.59	3.7E-01
SRRM2_HUMAN	Serine/arginine repetitive matrix protein 2 - Homo sapiens (Human)	VSGRT[181.014]SPPLLDR	1.11	1.0E+00	-2.07	2.9E-01	-1.88	1.0E-01
STA13_HUMAN	StAR-related lipid transfer protein 13 OS=Homo sapiens GN=STARD13 PE=1 SV=2	HKGSGRT[181.014]GGLVISGPMLQQEPES[166.9984]FK	1.00	9.1E-01	-2.30	7.4E-02	-2.30	3.2E-01
STMN1_HUMAN	Stathmin - Homo sapiens (Human)	RAS[166.9984]GQAFELILSPR	1.80	5.7E-01	-1.14	9.3E-01	1.58	6.0E-01
STUB1_HUMAN	STIP1 homology and U box-containing protein 1 OS=Homo sapiens GN=STUB1 PE=1 SV=2	LGAGGGS[166.9984]PEKSPSAQELK	1.20	9.0E-01	-1.45	6.6E-01	-1.21	7.3E-01
SUV41_HUMAN	Histone-lysine N-methyltransferase SUV420H1 OS=Homo sapiens GN=SUV420H1 PE=1 SV=2	QSM[147.0354]SRIPASS[166.9984]NS[166.9984]T[181.014]S[166.9984]SKLTHINNSR	-1.06	9.0E-01	1.18	8.8E-01	1.11	9.8E-01
SYCP1_HUMAN	Synaptonemal complex protein 1 OS=Homo sapiens GN=SYCP1 PE=1 SV=2	SVST[181.014]QKALEEDLQIAT[181.014]K	1.44	3.9E-01	-1.61	1.8E-01	-1.11	9.0E-01
SYG_HUMAN	Glycyl-tRNA synthetase - Homo sapiens (Human)	T[181.014]FFSFPAVVAPFKC[160.0307]SVLPLSQNQEFM[147.0354]PFVK	-1.23	6.1E-01	-1.44	5.5E-01	-1.77	1.4E-01
SYNP2_HUMAN	Synaptopodin-2 OS=Homo sapiens GN=SYNPO2 PE=1 SV=2	AQS[166.9984]PTPS[166.9984]LPASWK	1.86	3.5E-01	-1.30	7.9E-01	1.43	6.8E-01
SYNP2_HUMAN	Synaptopodin-2 OS=Homo sapiens GN=SYNPO2 PE=1 SV=2	AQS[166.9984]PTPSLPASWK	1.90	3.2E-01	-1.57	5.6E-01	1.21	8.3E-01
SYNPO_HUMAN	Synaptopodin OS=Homo sapiens GN=SYNPO PE=1 SV=2	AAS[166.9984]PAKPSSLDLVPNLPK	2.92	5.2E-02	-1.09	9.2E-01	2.68	2.4E-02
SYT5_HUMAN	Synaptotagmin-5 OS=Homo sapiens GN=SYT5 PE=2 SV=2	VY[243.0297]LLPDKR	3.66	1.2E-01	-1.58	7.6E-01	2.32	3.6E-01
TAU_HUMAN	Microtubule-associated protein tau - Homo sapiens (Human)	SPVVSGDTS[166.9984]PR	-1.00	9.3E-01	-1.01	9.3E-01	-1.01	1.0E+00
TAU_HUMAN	Microtubule-associated protein tau - Homo sapiens (Human)	TDHGAEIVYKS[166.9984]PVVS[166.9984]GDTS[166.9984]PR	-1.05	9.2E-01	-1.30	8.5E-01	-1.37	7.3E-01
TCAL3_HUMAN	Transcription elongation factor A protein-like 3 OS=Homo sapiens GN=TCEAL3 PE=1 SV=1	GTDDS[166.9984]PKDSQEDLQER	1.21	7.8E-01	1.18	9.4E-01	1.44	7.2E-01
TCAL3_HUMAN	Transcription elongation factor A protein-like 3 OS=Homo sapiens GN=TCEAL3 PE=1 SV=1	NEGNLENEGKPEDEVEPDDEGKS[166.9984]DEEEKPDVEGK	1.14	7.3E-01	1.00	8.3E-01	1.14	9.8E-01
TCEA1_HUMAN	Transcription elongation factor A protein 1 OS=Homo sapiens GN=TCEA1 PE=1 SV=2	KKEPAITSQNS[166.9984]PEAR	1.60	4.4E-01	1.35	3.5E-01	2.16	3.2E-04
TCEA3_HUMAN	Transcription elongation factor A protein 3 OS=Homo sapiens GN=TCEA3 PE=2 SV=2	GLEC[160.0307]SDWKPEAGLS[166.9984]PPR	1.28	8.6E-01	-1.42	7.6E-01	-1.11	9.1E-01
TCOF_HUMAN	Treacle protein OS=Homo sapiens GN=TCOF1 PE=1 SV=2	KLS[166.9984]GDQPAAR	1.44	6.5E-01	-1.72	4.7E-01	-1.20	7.3E-01
TCOF_HUMAN	Treacle protein OS=Homo sapiens GN=TCOF1 PE=1 SV=2	SLGNILQAKPT[181.014]SSPAK	1.32	8.1E-01	-1.75	4.9E-01	-1.32	5.4E-01
TCOF_HUMAN	Treacle protein OS=Homo sapiens GN=TCOF1 PE=1 SV=2	TSQVGAASAPAKES[166.9984]PR	-1.44	9.2E-01	-1.61	4.5E-01	-2.33	4.5E-02
TEBP_HUMAN	Prostaglandin E synthase 3 - Homo sapiens (Human)	DWEDDS[166.9984]DEDMSNFDR	1.60	9.2E-01	-2.24	7.5E-01	-1.41	8.1E-01
TEBP_HUMAN	Prostaglandin E synthase 3 - Homo sapiens (Human)	LNWLSVDFNNWKDWEDDS[166.9984]DEDM[147.0354]SNFDR	-2.65	3.6E-01	1.78	8.0E-01	-1.49	7.9E-01
TEBP_HUMAN	Prostaglandin E synthase 3 - Homo sapiens (Human)	LNWLSVDFNNWKDWEDDS[166.9984]DEDMSNFDR	-2.56	1.5E-01	1.60	5.0E-01	-1.60	6.9E-01
TELT_HUMAN	Telethonin OS=Homo sapiens GN=TCAP PE=1 SV=1	EEREDT[181.014]PIQLQELLALETALGGQC[160.0307]VDR	1.26	6.9E-01	1.07	9.3E-01	1.34	5.9E-01
TELT_HUMAN	Telethonin OS=Homo sapiens GN=TCAP PE=1 SV=1	SMS[166.9984]QEAQRG	-1.36	9.2E-01	-1.19	9.6E-01	-1.62	8.9E-01
TENS1_HUMAN	Tensin-1 OS=Homo sapiens GN=TNS1 PE=1 SV=2	AQFSVAGVHTVPGS[166.9984]PQAR	3.06	1.0E-03	-1.29	6.3E-01	2.37	5.5E-02
TENS1_HUMAN	Tensin-1 OS=Homo sapiens GN=TNS1 PE=1 SV=2	VATTPGS[166.9984]PSLGR	3.24	7.0E-03	-1.46	6.7E-01	2.21	2.4E-01
TGON2_HUMAN	Trans-Golgi network integral membrane protein 2 OS=Homo sapiens GN=TGOLN2 PE=1 SV=2	DSPSKS[166.9984]SAEAQTPEDTPNK	1.18	9.1E-01	-1.32	7.6E-01	-1.12	9.1E-01
TGON2_HUMAN	Trans-Golgi network integral membrane protein 2 OS=Homo sapiens GN=TGOLN2 PE=1 SV=2	DSPSKSS[166.9984]AEAQTPEDTPNK	1.14	9.0E-01	-1.40	7.1E-01	-1.23	8.8E-01
THUM1_HUMAN	THUMP domain-containing protein 1 OS=Homo sapiens GN=THUMPD1 PE=1 SV=2	FTDKDQQPS[166.9984]GS[166.9984]EGEDDDAEAALKK	1.28	3.5E-01	-1.34	3.7E-01	-1.05	7.3E-01
TIAM2_HUMAN	T-lymphoma invasion and metastasis-inducing protein 2 OS=Homo sapiens GN=TIAM2 PE=2 SV=3	QDSKS[166.9984]TSPGK	-1.05	7.1E-01	-1.42	5.2E-01	-1.49	3.4E-02
TITIN_HUMAN	Titin - Homo sapiens (Human)	AVS[166.9984]PTETKPTPTEK	3.10	3.0E-02	1.73	5.3E-01	5.36	7.8E-05
TITIN_HUMAN	Titin - Homo sapiens (Human)	AVTS[166.9984]PPR	3.76	4.6E-11	-2.18	7.0E-05	1.72	6.4E-01
TITIN_HUMAN	Titin - Homo sapiens (Human)	IELSPS[166.9984]M[147.0354]EAPK	1.90	6.4E-02	-1.54	3.3E-01	1.24	6.8E-01
TITIN_HUMAN	Titin - Homo sapiens (Human)	RRT[181.014]PS[166.9984]PDYDFYYRPR	3.01	2.2E-01	-2.48	3.8E-01	1.21	9.2E-01
TITIN_HUMAN	Titin - Homo sapiens (Human)	RVKS[166.9984]PEPSHPK	1.88	8.8E-02	-1.62	4.4E-01	1.16	7.7E-01
TITIN_HUMAN	Titin - Homo sapiens (Human)	S[166.9984]LS[166.9984]PTYIELM[147.0354]RPVSELIR	1.96	6.2E-01	-1.76	6.5E-01	1.11	9.4E-01
TITIN_HUMAN	Titin - Homo sapiens (Human)	SRPQPAEEYEDDT[181.014]ERRSPT[181.014]PER	1.60	2.6E-01	-1.48	4.3E-01	1.08	9.2E-01
TITIN_HUMAN	Titin - Homo sapiens (Human)	SRPQPAEEYEDDTERRS[166.9984]PTPER	3.73	3.6E-01	-3.57	4.4E-01	1.05	9.8E-01
TITIN_HUMAN	Titin - Homo sapiens (Human)	SRS[166.9984]PTPPSIAAK	2.03	5.9E-02	-2.05	1.8E-01	-1.01	8.0E-01
TITIN_HUMAN	Titin - Homo sapiens (Human)	VKS[166.9984]PEAVKS[166.9984]PK	2.56	3.7E-02	-2.64	4.5E-02	-1.03	9.6E-01
TM130_HUMAN	Transmembrane protein 130 OS=Homo sapiens GN=TMEM130 PE=2 SV=1	NAT[181.014]QQKDM[147.0354]VEVADFD<br>FSPM[147.0354]S[166.9984]DKNPEPPSGVR	-1.12	9.3E-01	1.59	6.8E-01	1.42	7.5E-01
TNNC1_HUMAN	Troponin C, slow skeletal and cardiac muscles OS=Homo sapiens GN=TNNC1 PE=1 SV=1	GKS[166.9984]EEELSDLFR	1.09	9.6E-01	-1.85	5.4E-01	-1.70	6.1E-01
TNNI3_HUMAN	Troponin I, cardiac muscle OS=Homo sapiens GN=TNNI3 PE=1 SV=3	RRS[166.9984]S[166.9984]NYR	-1.56	4.5E-01	-1.30	5.5E-01	-2.02	1.0E-01
TNNT2_HUMAN	Troponin T, cardiac muscle OS=Homo sapiens GN=TNNT2 PE=1 SV=3	ELWQS[166.9984]IYNLEAEKFDLQEK	-1.41	7.5E-01	-1.09	1.0E+00	-1.54	6.8E-01
TNR16_HUMAN	Tumor necrosis factor receptor superfamily member 16 OS=Homo sapiens GN=NGFR PE=1 SV=1	LHSDSGISVDS[166.9984]QSLHDQQPHTQTASGQALK	1.20	9.0E-01	-1.14	8.0E-01	1.05	9.5E-01
TOIP1_HUMAN	Torsin-1A-interacting protein 1 OS=Homo sapiens GN=TOR1AIP1 PE=1 SV=2	VNFSEEGET[181.014]EEDDQDSSHSSVTTVK	-1.21	7.2E-01	1.28	6.9E-01	1.06	9.1E-01
TOM70_HUMAN	Mitochondrial precursor proteins import receptor - Homo sapiens (Human)	AS[166.9984]PAPGSGHPEGPGAHLDM[147.0354]NSLDR	3.63	2.9E-01	-2.15	6.7E-01	1.69	6.6E-01
TPD53_HUMAN	Tumor protein D53 OS=Homo sapiens GN=TPD52L1 PE=1 SV=1	NSPTFKS[166.9984]FEER	-1.08	9.1E-01	1.12	9.6E-01	1.04	9.5E-01
TPD54_HUMAN	Tumor protein D54 - Homo sapiens (Human)	NSATFKS[166.9984]FEDR	2.65	2.0E-03	-1.24	6.9E-01	2.15	6.4E-02
TPIS_HUMAN	Triosephosphate isomerase - Homo sapiens (Human)	IIYGGS[166.9984]VTGATC[160.0307]K	2.69	4.9E-02	-2.17	2.2E-01	1.24	7.6E-01
TPIS_HUMAN	Triosephosphate isomerase - Homo sapiens (Human)	KQS[166.9984]LGELIGTLNAAK	-1.24	8.2E-01	-1.29	7.4E-01	-1.60	4.0E-01
TPM1_HUMAN	Tropomyosin alpha-1 chain - Homo sapiens (Human)	KLVIIES[166.9984]DLER	1.10	9.2E-01	-1.65	4.4E-01	-1.51	3.2E-01
TPM1_HUMAN	Tropomyosin alpha-1 chain - Homo sapiens (Human)	LVIIES[166.9984]DLERAEER	1.14	9.4E-01	-1.77	3.7E-01	-1.55	2.8E-01
TPM1_HUMAN	Tropomyosin alpha-1 chain - Homo sapiens (Human)	S[166.9984]IDDLEDELYAQK	-1.23	8.5E-01	-1.47	4.8E-01	-1.81	2.7E-01
TPM1_HUMAN	Tropomyosin alpha-1 chain OS=Homo sapiens GN=TPM1 PE=1 SV=2	AISEELDHALNDM[147.0354]TS[166.9984]I	2.39	2.4E-01	-2.79	1.4E-01	-1.16	8.1E-01
TPM1_HUMAN	Tropomyosin alpha-1 chain OS=Homo sapiens GN=TPM1 PE=1 SV=2	AISEELDHALNDMTS[166.9984]I	1.49	8.4E-01	-2.00	5.9E-01	-1.34	7.1E-01
TPM1_HUMAN	Tropomyosin alpha-1 chain OS=Homo sapiens GN=TPM1 PE=1 SV=2	ATDAEADVAS[166.9984]LNRR	1.33	7.8E-01	-1.97	3.1E-01	-1.48	6.6E-01
TPM1_HUMAN	Tropomyosin alpha-1 chain OS=Homo sapiens GN=TPM1 PE=1 SV=2	KATDAEADVAS[166.9984]LNR	1.45	6.8E-01	-2.24	2.7E-01	-1.54	5.6E-01
TPM1_HUMAN	Tropomyosin alpha-1 chain OS=Homo sapiens GN=TPM1 PE=1 SV=2	KATDAEADVAS[166.9984]LNRR	-1.89	5.7E-01	1.05	6.9E-01	-1.80	5.3E-01
TPM1_HUMAN	Tropomyosin alpha-1 chain OS=Homo sapiens GN=TPM1 PE=1 SV=2	LAT[181.014]ALQK	-1.32	8.8E-01	-2.27	3.8E-01	-3.00	1.4E-01
TPM2_HUMAN	Tropomyosin beta chain - Homo sapiens (Human)	AISEELDNALNDITS[166.9984]L	1.12	7.1E-01	-1.35	1.8E-01	-1.20	6.9E-01
TPPP_HUMAN	Tubulin polymerization-promoting protein OS=Homo sapiens GN=TPPP PE=1 SV=1	AANRT[181.014]PPKSPGDPSK	3.26	3.0E-02	-1.71	4.1E-01	1.91	7.1E-01
TPPP_HUMAN	Tubulin polymerization-promoting protein OS=Homo sapiens GN=TPPP PE=1 SV=1	AISS[166.9984]PTVSR	1.05	1.2E-06	-2.47	2.1E-07	-2.36	1.4E-01
TR150_HUMAN	Thyroid hormone receptor-associated protein 3 OS=Homo sapiens GN=THRAP3 PE=1 SV=2	ASAVSELS[166.9984]PR	1.86	1.2E-01	-1.74	1.2E-01	1.07	9.0E-01
TR150_HUMAN	Thyroid hormone receptor-associated protein 3 OS=Homo sapiens GN=THRAP3 PE=1 SV=2	ERS[166.9984]PALKS[166.9984]PLQSVVVR	1.61	5.4E-01	-1.92	3.2E-01	-1.20	8.2E-01
TR150_HUMAN	Thyroid hormone receptor-associated protein 3 OS=Homo sapiens GN=THRAP3 PE=1 SV=2	GSFS[166.9984]DTGLGDGK	1.01	9.8E-01	-1.21	8.5E-01	-1.20	8.0E-01
TR150_HUMAN	Thyroid hormone receptor-associated protein 3 OS=Homo sapiens GN=THRAP3 PE=1 SV=2	IDIS[166.9984]PSTFR	-1.02	1.0E+00	-1.29	5.4E-01	-1.31	5.5E-01
TR150_HUMAN	Thyroid hormone receptor-associated protein 3 OS=Homo sapiens GN=THRAP3 PE=1 SV=2	MDS[166.9984]FDEDLARPSGLLAQER	1.41	4.9E-01	-2.15	5.2E-02	-1.52	4.1E-01
TR150_HUMAN	Thyroid hormone receptor-associated protein 3 OS=Homo sapiens GN=THRAP3 PE=1 SV=2	NKKS[166.9984]PEIHR	1.10	9.2E-01	-1.73	3.8E-01	-1.57	3.6E-01
TR150_HUMAN	Thyroid hormone receptor-associated protein 3 OS=Homo sapiens GN=THRAP3 PE=1 SV=2	RIDIS[166.9984]PSTFR	-3.09	3.9E-02	1.37	8.9E-01	-2.25	3.0E-01
TR150_HUMAN	Thyroid hormone receptor-associated protein 3 OS=Homo sapiens GN=THRAP3 PE=1 SV=2	S[166.9984]PPSTGSTYGSSQK	-1.96	9.3E-02	-1.19	7.2E-01	-2.34	1.9E-02
TR150_HUMAN	Thyroid hormone receptor-associated protein 3 OS=Homo sapiens GN=THRAP3 PE=1 SV=2	SPVGKS[166.9984]PPSTGSTYGSSQK	1.35	9.2E-01	-3.17	3.4E-01	-2.34	4.8E-01
TRA2A_HUMAN	Transformer-2 protein homolog OS=Homo sapiens GN=TRA2A PE=1 SV=1	AHT[181.014]PTPGIYM[147.0354]GRPTHSGGGGGGGGGGGGGGR	1.17	7.1E-01	-1.32	3.6E-01	-1.13	5.1E-01
TRA2A_HUMAN	Transformer-2 protein homolog OS=Homo sapiens GN=TRA2A PE=1 SV=1	RRS[166.9984]PS[166.9984]PYYSR	1.13	9.0E-01	-1.29	5.9E-01	-1.14	6.8E-01
TRA2A_HUMAN	Transformer-2 protein homolog OS=Homo sapiens GN=TRA2A PE=1 SV=1	RRS[166.9984]PSPY[243.0297]YSR	2.49	5.3E-01	-4.52	1.5E-01	-1.81	3.4E-01
TRA2B_HUMAN	Splicing factor, arginine/serine-rich 10 OS=Homo sapiens GN=SFRS10 PE=1 SV=1	RHS[166.9984]HS[166.9984]HS[166.9984]PMSTR	-2.20	9.0E-01	2.74	4.6E-01	1.25	7.3E-01
TRA2B_HUMAN	Splicing factor, arginine/serine-rich 10 OS=Homo sapiens GN=SFRS10 PE=1 SV=1	RPHT[181.014]PTPGIYM[147.0354]GRPTYGSSR	3.27	6.7E-01	-4.13	4.6E-01	-1.27	8.1E-01
TRAK1_HUMAN	Trafficking kinesin-binding protein 1 OS=Homo sapiens GN=TRAK1 PE=1 SV=1	NINQVVKQRS[166.9984]LT[181.014]PS[166.9984]PM[147.0354]N<br>IPGS[166.9984]NQSSAM[147.0354]NSLLSSC[160.0307]VST[181.014]PR	1.12	7.4E-01	1.93	1.1E-01	2.17	6.6E-02
TRIP4_HUMAN	Activating signal cointegrator 1 OS=Homo sapiens GN=TRIP4 PE=1 SV=4	LERET[181.014]LQK	1.45	5.8E-01	-1.14	7.9E-01	1.28	9.3E-01
TRPC1_HUMAN	Short transient receptor potential channel 1 OS=Homo sapiens GN=TRPC1 PE=1 SV=1	M[147.0354]MAALYPSTDLSGASSSSLPSSPSSS[166.9984]SPNEVM[147.0354]ALKDVREVK	1.17	9.4E-01	-1.13	8.2E-01	1.03	8.2E-01
TTC15_HUMAN	Tetratricopeptide repeat protein 15 OS=Homo sapiens GN=TTC15 PE=1 SV=3	LKDS[166.9984]LR	1.84	2.9E-01	-1.46	5.5E-01	1.25	8.8E-01
TTC28_HUMAN	Tetratricopeptide repeat protein 28 OS=Homo sapiens GN=TTC28 PE=2 SV=3	NM[147.0354]S[166.9984]PS[166.9984]SGHQSPA<br>GSAPSPALSYSS[166.9984]AGS[166.9984]AR	1.13	8.8E-01	1.13	7.9E-01	1.27	5.6E-01
TTLL4_HUMAN	Tubulin polyglutamylase TTLL4 OS=Homo sapiens GN=TTLL4 PE=1 SV=2	M[147.0354]ASAGT[181.014]QHY[243.0297]S[166.9984]IGLRQKNSFK	1.75	3.7E-01	-1.26	7.8E-01	1.39	6.6E-01
VASP_HUMAN	Vasodilator-stimulated phosphoprotein OS=Homo sapiens GN=VASP PE=1 SV=3	KVS[166.9984]KQEEASGGPTAPK	1.13	9.8E-01	1.58	6.8E-01	1.79	6.3E-01
VDAC1_HUMAN	Voltage-dependent anion-selective channel protein 1 - Homo sapiens (Human)	VNNS[166.9984]SLIGLGYTQTLKPGIK	-1.30	8.0E-01	-1.89	1.6E-01	-2.44	1.4E-02
VDAC2_HUMAN	Voltage-dependent anion-selective channel protein 2 - Homo sapiens (Human)	LTFDTTFSPNT[181.014]GK	-1.12	9.8E-01	-1.15	8.9E-01	-1.29	8.7E-01
VDAC2_HUMAN	Voltage-dependent anion-selective channel protein 2 - Homo sapiens (Human)	VNNS[166.9984]SLIGVGYTQTLRPGVK	-1.30	6.8E-01	-1.87	1.9E-01	-2.43	1.4E-02
VIME_HUMAN	Vimentin - Homo sapiens (Human)	LRS[166.9984]SVPGVR	1.07	9.8E-01	4.16	2.1E-02	4.45	1.7E-02
VIME_HUMAN	Vimentin - Homo sapiens (Human)	TYS[166.9984]LGSALRPSTSR	-1.31	8.8E-01	2.31	6.3E-01	1.76	6.8E-01
VINEX_HUMAN	Vinexin OS=Homo sapiens GN=SORBS3 PE=1 SV=1	LC[160.0307]DDGPQLPTS[166.9984]PR	1.55	6.1E-01	1.16	8.5E-01	1.79	3.0E-01
WD42A_HUMAN	WD repeat-containing protein 42A OS=Homo sapiens GN=WDR42A PE=1 SV=1	VHDRS[166.9984]EEEEEEEEEEEEQPR	1.28	7.3E-01	-1.15	8.4E-01	1.12	9.1E-01
YETS2_HUMAN	YEATS domain-containing protein 2 OS=Homo sapiens GN=YEATS2 PE=1 SV=2	T[181.014]TLFTQAAHGGQASLM[147.0354]KISDSTLK	-3.54	3.0E-04	4.00	1.0E-03	1.13	9.0E-01
YTDC1_HUMAN	YTH domain-containing protein 1 OS=Homo sapiens GN=YTHDC1 PE=1 SV=3	GIS[166.9984]PIVFDR	-1.04	1.0E+00	-1.18	8.8E-01	-1.23	8.8E-01
YTDC1_HUMAN	YTH domain-containing protein 1 OS=Homo sapiens GN=YTHDC1 PE=1 SV=3	LSSESHHGGS[166.9984]PIHWVLPAGM[147.0354]SAK	2.13	9.4E-01	-3.26	5.0E-01	-1.53	3.3E-01
ZC3HD_HUMAN	Zinc finger CCCH domain-containing protein 13 OS=Homo sapiens GN=ZC3H13 PE=1 SV=1	SLS[166.9984]PSHLTEDR	1.90	2.8E-01	-2.48	1.5E-01	-1.31	7.0E-01
ZCH12_HUMAN	Zinc finger CCHC domain-containing protein 12 OS=Homo sapiens GN=ZCCHC12 PE=2 SV=2	S[166.9984]LGRSLGPIMASMADR	-1.79	9.6E-01	-1.08	9.1E-01	-1.93	9.0E-01
ZCH18_HUMAN	Zinc finger CCCH domain-containing protein 18 OS=Homo sapiens GN=ZC3H18 PE=1 SV=1	LGVSVS[166.9984]PSR	1.50	6.1E-01	-1.80	4.3E-01	-1.20	5.7E-01
ZEB2_HUMAN	Zinc finger E-box-binding homeobox 2 OS=Homo sapiens GN=ZEB2 PE=1 SV=1	TGS[166.9984]S[166.9984]PNS[166.9984]PM[147.0354]VS[166.9984]SSPT<br>NSAITQLRNKLENGKPLSMSEQTGLLK	2.24	4.1E-02	-1.10	8.6E-01	2.05	1.4E-01
ZFP2_HUMAN	Zinc finger protein 2 homolog OS=Homo sapiens GN=ZFP2 PE=2 SV=1	C[160.0307]GKSFSQS[166.9984]T[181.014]YLIEHQR	-1.28	7.1E-01	3.42	3.3E-02	2.67	2.9E-01
ZN281_HUMAN	Zinc finger protein 281 OS=Homo sapiens GN=ZNF281 PE=1 SV=1	IGS[166.9984]GFLS[166.9984]GGGGTGSSGGSGSGGGGSGGGGGGGGSSGRR	-1.23	8.7E-01	-1.74	2.4E-02	-2.14	9.8E-02
ZN410_HUMAN	Zinc finger protein 410 OS=Homo sapiens GN=ZNF410 PE=1 SV=2	QFTT[181.014]AGNLK	-1.01	9.2E-01	-1.12	8.7E-01	-1.13	7.7E-01
ZN433_HUMAN	Zinc finger protein 433 OS=Homo sapiens GN=ZNF433 PE=2 SV=1	QC[160.0307]GKAFRSASLLQT[181.014]HGR	-1.18	1.2E-01	1.81	3.0E-03	1.53	9.3E-02
ZN644_HUMAN	Zinc finger protein 644 OS=Homo sapiens GN=ZNF644 PE=1 SV=2	RS[166.9984]FLQQDVNK	1.19	8.3E-01	-1.15	8.6E-01	1.04	9.5E-01
ZNF22_HUMAN	Zinc finger protein 22 OS=Homo sapiens GN=ZNF22 PE=1 SV=3	S[166.9984]LDDKPYK	-2.19	6.8E-01	1.58	8.3E-01	-1.39	9.0E-01
ZNF34_HUMAN	Zinc finger protein 34 OS=Homo sapiens GN=ZNF34 PE=2 SV=2	DVMLETYGNLVSLGVGPAGPKPGVIS[166.9984]QLER	-1.07	9.2E-01	-1.31	7.4E-01	-1.40	6.6E-01

ZNRF2_HUMAN	E3 ubiquitin-protein ligase ZNRF2 OS=Homo sapiens GN=ZNRF2 PE=1 SV=1	DRPVGGS[166.9984]PGGPR	1.45	1.7E-07	1.07	1.0E-03	1.55	6.8E-01
ZP4_HUMAN	Zona pellucida sperm-binding protein 4 OS=Homo sapiens GN=ZP4 PE=2 SV=1	DKNY[243.0297]GS[166.9984]YYGVGDYPVVKLLR	-1.29	2.1E-01	-2.14	1.2E-02	-2.77	2.5E-07
ZRAB2_HUMAN	Zinc finger Ran-binding domain-containing protein 2 OS=Homo sapiens GN=ZRANB2 PE=1 SV=2	ENVEYIEREES[166.9984]DGEYDEFGR	-1.01	9.9E-01	-1.09	8.5E-01	-1.11	8.9E-01
ZRAB2_HUMAN	Zinc finger Ran-binding domain-containing protein 2 OS=Homo sapiens GN=ZRANB2 PE=1 SV=2	EVEDKES[166.9984]EGEEEDEDEDLSK	-1.05	9.2E-01	-1.08	8.8E-01	-1.13	7.7E-01
ZRAB2_HUMAN	Zinc finger Ran-binding domain-containing protein 2 OS=Homo sapiens GN=ZRANB2 PE=1 SV=2	YNLDAS[166.9984]EEEDSNKK	1.01	9.7E-01	-1.32	3.2E-01	-1.30	2.9E-01
ZSC22_HUMAN	Zinc finger and SCAN domain-containing protein 22 OS=Homo sapiens GN=ZSCAN22 PE=1 SV=2	QSDLGESEPS[166.9984]NVTETLM[147.0354]GGVSLGPAFVK	1.50	6.2E-01	-1.26	8.2E-01	1.18	7.6E-01
