# Supplementary material for: Phosphoproteomic Profiling of Human Myocardial Tissues Distinguishes Ischemic from Non-Ischemic End Stage Heart Failure
Source: PLoS One. 2014 Aug 12;9(8):e104157. doi: 10.1371/journal.pone.0104157 (PMC4130503; doi:10.1371/journal.pone.0104157)
Supplement: Table S3 — Expression profile of 68 unenriched overlapping proteins. (PDF) [file pone.0104157.s005.pdf]

SUPPLEMENTAL TABLE 3. EXPRESSION PROFILE OF 68 UNENRICHED OVERLAPPING PROTEINS

Entry Name	Protein Description	ProteinTeller Probability	Peptide Count	NIF v IF Fold Change	NIF v IF p-value (ANOVA)	IF v NF Fold Change	IF v NF p-value (ANOVA)	NIF v NF Fold Change	NIF v NF p-value (ANOVA)
ALBU_HUMAN	Serum albumin OS=Homo sapiens GN=ALB PE=1 SV=2	1	242	1.07	6.8E-01	1.36	5.0E-07	1.46	1.9E-06
ALDOA_HUMAN	Fructose-bisphosphate aldolase A OS=Homo sapiens GN=ALDOA PE=1 SV=2	1	24	1.10	8.2E-01	-1.25	4.1E-01	-1.13	5.4E-01
ANT3_HUMAN	Antithrombin-III OS=Homo sapiens GN=SERPINC1 PE=1 SV=1	1	16	-1.32	4.3E-01	1.27	4.3E-01	-1.04	7.9E-01
BASI_HUMAN	Basigin OS=Homo sapiens GN=BSG PE=1 SV=2	1	14	-1.13	8.1E-01	-1.19	2.7E-01	-1.35	5.0E-02
CALD1_HUMAN	Caldesmon OS=Homo sapiens GN=CALD1 PE=1 SV=2	1	6	1.17	6.8E-01	1.03	8.9E-01	1.21	1.4E-01
CAPZB_HUMAN	F-actin-capping protein subunit beta OS=Homo sapiens GN=CAPZB PE=1 SV=4	1	2	1.02	9.7E-01	-1.07	8.4E-01	-1.05	8.2E-01
CASQ2_HUMAN	Calsequestrin-2 OS=Homo sapiens GN=CASQ2 PE=1 SV=2	1	33	-1.23	3.2E-01	-1.03	8.4E-01	-1.27	2.9E-02
CRIP2_HUMAN	Cysteine-rich protein 2 OS=Homo sapiens GN=CRIP2 PE=1 SV=1	1	11	-1.00	9.7E-01	1.06	7.3E-01	1.05	5.9E-01
CRYAB_HUMAN	Alpha-crystallin B chain OS=Homo sapiens GN=CRYAB PE=1 SV=2	1	12	-1.10	7.5E-01	-1.02	8.8E-01	-1.12	2.5E-01
CSPG2_HUMAN	Versican core protein OS=Homo sapiens GN=VCAN PE=1 SV=3	1	9	1.50	8.7E-01	1.58	4.9E-01	2.37	1.4E-01
CSRP3_HUMAN	Cysteine and glycine-rich protein 3 OS=Homo sapiens GN=CSRP3 PE=1 SV=1	1	39	-1.11	7.1E-01	-1.00	1.0E+00	-1.12	5.4E-01
CYC_HUMAN	Cytochrome c OS=Homo sapiens GN=CYCS PE=1 SV=2	1	36	-1.03	9.5E-01	-1.17	1.6E-01	-1.20	2.6E-01
DESM_HUMAN	Desmin OS=Homo sapiens GN=DES PE=1 SV=3	1	33	1.15	8.7E-01	1.07	8.3E-01	1.23	4.0E-01
DSG2_HUMAN	Desmoglein-2 OS=Homo sapiens GN=DSG2 PE=1 SV=2	1	4	1.08	9.4E-01	-1.27	4.9E-01	-1.18	5.3E-01
EF1B_HUMAN	Elongation factor 1-beta OS=Homo sapiens GN=EEF1B2 PE=1 SV=3	0.82	1	-1.13	6.8E-01	1.10	4.5E-01	-1.03	8.6E-01
FBLN1_HUMAN	Fibulin-1 OS=Homo sapiens GN=FBLN1 PE=1 SV=4	0.99	2	1.13	8.9E-01	1.90	2.8E-02	2.16	4.7E-02
FETUA_HUMAN	Alpha-2-HS-glycoprotein OS=Homo sapiens GN=AHSG PE=1 SV=1	1	14	1.03	9.4E-01	1.66	1.1E-11	1.71	1.3E-06
FHL2_HUMAN	Four and a half LIM domains protein 2 OS=Homo sapiens GN=FHL2 PE=1 SV=3	1	40	-1.02	9.9E-01	-1.19	4.9E-01	-1.22	4.5E-01
FRIH_HUMAN	Ferritin heavy chain OS=Homo sapiens GN=FTH1 PE=1 SV=2	1	8	1.51	2.0E-01	-1.78	6.7E-03	-1.18	4.6E-01
G3P_HUMAN	Glyceraldehyde-3-phosphate dehydrogenase OS=Homo sapiens GN=GAPDH PE=1 SV=3	1	57	-1.02	9.8E-01	-1.06	7.3E-01	-1.08	6.1E-01
H12_HUMAN	Histone H1.2 OS=Homo sapiens GN=HIST1H1C PE=1 SV=2	1	21	1.03	9.5E-01	-1.10	5.4E-01	-1.07	5.3E-01
H31_HUMAN	Histone H3.1 OS=Homo sapiens GN=HIST1H3A PE=1 SV=2	1	1	1.21	7.9E-01	1.01	9.0E-01	1.22	3.4E-01
HNRPD_HUMAN	Heterogeneous nuclear ribonucleoprotein D0 OS=Homo sapiens GN=HNRNPD PE=1 SV=1	1	5	1.07	6.8E-01	-1.16	4.5E-02	-1.09	4.6E-02
HP1B3_HUMAN	Heterochromatin protein 1-binding protein 3 OS=Homo sapiens GN=HP1BP3 PE=1 SV=1	1	4	-1.03	9.5E-01	-1.18	2.8E-01	-1.21	1.3E-01
HS90A_HUMAN	Heat shock protein HSP 90-alpha OS=Homo sapiens GN=HSP90AA1 PE=1 SV=5	1	16	1.05	9.5E-01	-1.07	8.0E-01	-1.02	8.7E-01
HS90B_HUMAN	Heat shock protein HSP 90-beta OS=Homo sapiens GN=HSP90AB1 PE=1 SV=4	1	3	1.06	9.3E-01	-1.15	6.6E-01	-1.08	7.7E-01
HSPB1_HUMAN	Heat shock protein beta-1 OS=Homo sapiens GN=HSPB1 PE=1 SV=2	1	24	-1.10	6.8E-01	1.06	5.1E-01	-1.04	7.4E-01
HSPB7_HUMAN	Heat shock protein beta-7 OS=Homo sapiens GN=HSPB7 PE=1 SV=1	1	13	-1.15	3.4E-01	-1.15	4.2E-01	-1.33	1.1E-02
ICAL_HUMAN	Calpastatin OS=Homo sapiens GN=CAST PE=1 SV=4	1	20	-1.40	3.4E-01	1.06	8.1E-01	-1.31	7.6E-02
KAD1_HUMAN	Adenylate kinase isoenzyme 1 OS=Homo sapiens GN=AK1 PE=1 SV=3	1	19	1.12	2.1E-01	-1.26	3.6E-02	-1.12	2.4E-01
KCRM_HUMAN	Creatine kinase M-type OS=Homo sapiens GN=CKM PE=1 SV=2	1	41	-1.23	6.8E-01	-1.41	2.8E-01	-1.73	4.5E-03
KCRS_HUMAN	Creatine kinase S-type, mitochondrial OS=Homo sapiens GN=CKMT2 PE=1 SV=2	1	22	1.03	9.7E-01	-1.28	4.8E-01	-1.24	2.6E-01
KNG1_HUMAN	Kininogen-1 OS=Homo sapiens GN=KNG1 PE=1 SV=2	1	10	-1.16	7.3E-01	1.67	4.2E-03	1.44	1.1E-01
LU_HUMAN	Lutheran blood group glycoprotein precursor - Homo sapiens	1	7	-1.07	8.7E-01	-1.17	7.4E-02	-1.26	4.6E-02
MARCS_HUMAN	Myristoylated alanine-rich C-kinase substrate OS=Homo sapiens GN=MARCKS PE=1 SV=4	1	3	-1.16	8.7E-01	1.06	8.3E-01	-1.10	6.9E-01
MLRV_HUMAN	Myosin regulatory light chain 2, ventricular/cardiac muscle isoform OS=Homo sapiens GN=MYL2 PE=1 SV=3	1	43	1.11	7.4E-01	-1.29	2.8E-03	-1.15	2.5E-01
MRLC2_HUMAN	Myosin regulatory light chain MRLC2 OS=Homo sapiens GN=MYLC2B PE=1 SV=2	0.93	2	1.01	9.9E-01	1.24	3.1E-01	1.26	1.1E-01
MYOZ2_HUMAN	Myozenin-2 OS=Homo sapiens GN=MYOZ2 PE=1 SV=1	1	38	1.02	9.6E-01	-1.16	4.9E-01	-1.13	4.4E-01
MYPT1_HUMAN	Protein phosphatase 1 regulatory subunit 12A OS=Homo sapiens GN=PPP1R12A PE=1 SV=1	0	1	-1.28	5.3E-01	1.03	8.3E-01	-1.24	2.2E-01
MYPT2_HUMAN	Protein phosphatase 1 regulatory subunit 12B OS=Homo sapiens GN=PPP1R12B PE=1 SV=2	1	4	-1.13	8.8E-01	-1.23	2.6E-01	-1.39	1.8E-01
NCAM1_HUMAN	Neural cell adhesion molecule 1 OS=Homo sapiens GN=NCAM1 PE=1 SV=3	1	3	-1.22	6.8E-01	-1.20	7.1E-01	-1.46	5.9E-02
NEBL_HUMAN	Nebulette OS=Homo sapiens GN=NEBL PE=1 SV=1	1	7	1.30	7.1E-01	-1.23	5.5E-01	1.06	9.4E-01
NEXN_HUMAN	Nexilin OS=Homo sapiens GN=NEXN PE=1 SV=1	0.98	2	1.19	7.9E-01	-1.24	4.3E-01	-1.04	9.4E-01
NP1L4_HUMAN	Nucleosome assembly protein 1-like 4 OS=Homo sapiens GN=NAP1L4 PE=1 SV=1	0.98	2	-1.06	8.7E-01	-1.02	9.6E-01	-1.08	3.5E-01
OCAD1_HUMAN	OCIA domain-containing protein 1 OS=Homo sapiens GN=OCIAD1 PE=1 SV=1	1	6	-1.02	9.5E-01	-1.04	8.4E-01	-1.05	6.9E-01
ODPA_HUMAN	Pyruvate dehydrogenase E1 component subunit alpha, somatic form, mitochondrial OS=Homo sapiens GN=PDHA1 PE=1 SV=3	1	11	1.05	9.4E-01	-1.23	4.6E-01	-1.16	5.0E-01
PEBP1_HUMAN	Phosphatidylethanolamine-binding protein 1 OS=Homo sapiens GN=PEBP1 PE=1 SV=3	1	16	-1.08	5.5E-01	-1.16	8.4E-03	-1.25	3.6E-04
PGRC2_HUMAN	Membrane-associated progesterone receptor component 2 OS=Homo sapiens GN=PGRMC2 PE=1 SV=1	0	1	-1.07	7.5E-01	-1.15	2.1E-01	-1.23	3.0E-02
POPD1_HUMAN	Blood vessel epicardial substance OS=Homo sapiens GN=BVES PE=2 SV=1	0.94	1	-1.20	8.4E-01	-1.17	5.7E-01	-1.40	1.5E-01
PTRF_HUMAN	Polymerase I and transcript release factor OS=Homo sapiens GN=PTRF PE=1 SV=1	1	19	1.07	7.3E-01	-1.08	4.9E-01	-1.02	9.2E-01
QCR6_HUMAN	Cytochrome b-c1 complex subunit 6, mitochondrial OS=Homo sapiens GN=UQCRH PE=1 SV=2	1	11	-1.17	7.9E-01	-1.36	2.8E-03	-1.59	4.1E-02
RLA2_HUMAN	60S acidic ribosomal protein P2 OS=Homo sapiens GN=RPLP2 PE=1 SV=1	1	7	-1.16	6.8E-01	-1.18	2.7E-01	-1.36	1.0E-03
ROA3_HUMAN	Heterogeneous nuclear ribonucleoprotein A3 OS=Homo sapiens GN=HNRNPA3 PE=1 SV=2	0	1	1.09	7.8E-01	-1.18	3.1E-01	-1.08	5.3E-01
SDPR_HUMAN	Serum deprivation-response protein OS=Homo sapiens GN=SDPR PE=1 SV=3	1	14	-1.08	6.8E-01	-1.03	4.9E-01	-1.12	1.2E-01
SRBS2_HUMAN	Sorbin and SH3 domain-containing protein 2 OS=Homo sapiens GN=SORBS2 PE=1 SV=3	0.96	1	1.44	6.5E-01	1.01	9.4E-01	1.46	2.0E-01
SRCH_HUMAN	Sarcoplasmic reticulum histidine-rich calcium-binding protein OS=Homo sapiens GN=HRC PE=2 SV=1	1	23	-1.36	3.3E-01	1.02	8.9E-01	-1.33	6.5E-02
TEBP_HUMAN	Prostaglandin E synthase 3 OS=Homo sapiens GN=PTGES3 PE=1 SV=1	1	4	-1.16	6.2E-01	-1.10	6.1E-01	-1.28	2.2E-02
TELT_HUMAN	Telethonin OS=Homo sapiens GN=TCAP PE=1 SV=1	1	4	1.11	7.9E-01	-1.51	7.3E-04	-1.36	2.3E-02
TNNC1_HUMAN	Troponin C, slow skeletal and cardiac muscles OS=Homo sapiens GN=TNNC1 PE=1 SV=1	1	42	-1.12	7.1E-01	-1.22	2.3E-03	-1.36	2.4E-03
TNNI3_HUMAN	Troponin I, cardiac muscle OS=Homo sapiens GN=TNNI3 PE=1 SV=3	1	41	-1.05	9.1E-01	-1.13	6.2E-02	-1.19	1.7E-01
TNNT2_HUMAN	Troponin T, cardiac muscle OS=Homo sapiens GN=TNNT2 PE=1 SV=3	1	66	-1.16	5.8E-01	-1.23	2.2E-02	-1.42	1.2E-05
TPIS_HUMAN	Triosephosphate isomerase OS=Homo sapiens GN=TPI1 PE=1 SV=2	1	23	1.11	6.8E-01	-1.22	2.0E-01	-1.10	4.4E-01
TPM1_HUMAN	Tropomyosin alpha-1 chain OS=Homo sapiens GN=TPM1 PE=1 SV=2	1	73	-1.09	5.8E-01	-1.16	1.6E-02	-1.27	2.5E-04
TPM2_HUMAN	Tropomyosin beta chain OS=Homo sapiens GN=TPM2 PE=1 SV=1	1	62	-1.10	3.4E-01	-1.23	2.7E-03	-1.35	7.7E-06
TPPP_HUMAN	Tubulin polymerization-promoting protein OS=Homo sapiens GN=TPPP PE=1 SV=1	0.83	1	-1.04	9.6E-01	-1.41	1.7E-01	-1.47	1.3E-01
VDAC1_HUMAN	Voltage-dependent anion-selective channel protein 1 OS=Homo sapiens GN=VDAC1 PE=1 SV=2	1	10	1.01	9.7E-01	-1.17	4.9E-01	-1.16	2.2E-01
VDAC2_HUMAN	Voltage-dependent anion-selective channel protein 2 OS=Homo sapiens GN=VDAC2 PE=1 SV=2	1	14	-1.03	9.5E-01	-1.17	3.3E-01	-1.21	8.4E-02
VIME_HUMAN	Vimentin OS=Homo sapiens GN=VIM PE=1 SV=4	1	18	1.33	7.3E-01	1.16	6.9E-01	1.54	1.4E-01
