# Supplementary material for: Phosphoproteomic Profiling of Human Myocardial Tissues Distinguishes Ischemic from Non-Ischemic End Stage Heart Failure
Source: PLoS One. 2014 Aug 12;9(8):e104157. doi: 10.1371/journal.pone.0104157 (PMC4130503; doi:10.1371/journal.pone.0104157)
Supplement: Table S4 — Differential phosphorylation of proteins in NIF and IF heart. (PDF) [file pone.0104157.s006.pdf]

SUPPLMENTAL TABLE 4: DIFFERENTIAL PHOSPHORYLATION OF PROTEINS IN NIF AND IF HEART

Entry Name	Protein Description	Modified Peptide Sequence	NIF v IF Fold Change	NIF v IF p-value (ANOVA)	IF v NF Fold Change	IF v NF p-value (ANOVA)	NIF v NF Fold Change	NIF v NF p-value (ANOVA
ALBU_HUMAN	Serum albumin precursor - Homo sapiens (Human)	TC[160.0307]VADES[166.9984]AENC[160.0307]DK	1.27	7.1E-01	2.34	2.6E-05	2.99	1.3E-07
ALDOA_HUMAN	Fructose-bisphosphate aldolase A - Homo sapiens (Human)	GILAADES[166.9984]TGSIAK	-1.86	1.2E-01	1.00	8.2E-01	-1.85	1.7E-01
ALDOA_HUMAN	Fructose-bisphosphate aldolase A - Homo sapiens (Human)	GILAADESTGS[166.9984]IAK	-1.38	5.0E-01	1.02	8.5E-01	-1.35	5.9E-01
ALDOA_HUMAN	Fructose-bisphosphate aldolase A - Homo sapiens (Human)	GILAADESTGS[166.9984]IAKR	1.43	9.0E-01	-1.85	5.9E-01	-1.29	6.0E-01
ANT3_HUMAN	Antithrombin-III OS=Homo sapiens GN=SERPINC1 PE=1 SV=1	ATEDEGS[166.9984]EQKIPEATNR	-1.40	2.9E-01	2.77	1.2E-01	1.98	5.6E-01
ANT3_HUMAN	Antithrombin-III OS=Homo sapiens GN=SERPINC1 PE=1 SV=1	KATEDEGS[166.9984]EQKIPEATNR	-1.50	3.4E-01	2.50	2.1E-01	1.66	6.6E-01
BASI_HUMAN	Basigin precursor - Homo sapiens (Human)	KPEDVLDDDDAGSAPLKSS[166.9984]GQHQNDK	-7.27	1.3E-05	4.62	4.6E-04	-1.57	6.8E-01
BASI_HUMAN	Basigin precursor - Homo sapiens (Human)	RKPEDVLDDDDAGS[166.9984]APLK	1.27	6.1E-01	-1.66	2.2E-01	-1.31	7.3E-01
BASI_HUMAN	Basigin precursor - Homo sapiens (Human)	RKPEDVLDDDDAGSAPLKS[166.9984]SGQHQNDK	-5.36	2.2E-04	2.48	5.9E-02	-2.16	5.7E-01
BASI_HUMAN	Basigin precursor - Homo sapiens (Human)	RKPEDVLDDDDAGSAPLKSS[166.9984]GQHQNDK	-4.36	3.6E-02	2.56	9.4E-02	-1.70	8.6E-01
CALD1_HUMAN	Caldesmon OS=Homo sapiens GN=CALD1 PE=1 SV=2	RGS[166.9984]IGENQVEVM[147.0354]VEEK	1.79	6.4E-01	-5.25	1.6E-02	-2.93	1.9E-02
CALD1_HUMAN	Caldesmon OS=Homo sapiens GN=CALD1 PE=1 SV=2	TPDGNKS[166.9984]PAPKPSDLRPGDVSSK	1.62	4.0E-01	-1.28	6.0E-01	1.26	9.7E-01
CAPZB_HUMAN	F-actin-capping protein subunit beta - Homo sapiens (Human)	ELS[166.9984]QVLTQR	-2.24	6.0E-03	1.27	7.6E-01	-1.76	1.4E-01
CASQ2_HUMAN	Calsequestrin-2 OS=Homo sapiens GN=CASQ2 PE=1 SV=2	KYDLLC[160.0307]LYYHEPVS[166.9984]SDKVTQK	-2.48	2.4E-01	-1.55	4.0E-01	-3.84	2.1E-02
CRIP2_HUMAN	Cysteine-rich protein 2 - Homo sapiens (Human)	ASS[166.9984]VTTFTGEPNTC[160.0307]PR	-1.61	5.2E-04	1.36	6.4E-01	-1.19	6.8E-01
CRYAB_HUMAN	Alpha-crystallin B chain OS=Homo sapiens GN=CRYAB PE=1 SV=2	RPFFPFHSPS[166.9984]R	-1.64	6.3E-01	1.11	9.8E-01	-1.48	7.4E-01
CSPG2_HUMAN	Versican core protein OS=Homo sapiens GN=VCAN PE=1 SV=3	TDGQVS[166.9984]GEAIK	2.31	1.1E-01	-1.05	9.1E-01	2.20	3.6E-01
CSRP3_HUMAN	Cysteine and glycine-rich protein 3 OS=Homo sapiens GN=CSRP3 PE=1 SV=1	FGES[166.9984]EKC[160.0307]PR	-1.65	3.1E-01	1.02	1.0E+00	-1.62	2.9E-01
CSRP3_HUMAN	Cysteine and glycine-rich protein 3 OS=Homo sapiens GN=CSRP3 PE=1 SV=1	GIGYGQGAGC[160.0307]LST[181.014]DTGEHLGLQFQQSPKPAR	-1.25	3.4E-01	-1.04	1.0E+00	-1.29	3.4E-01
CSRP3_HUMAN	Cysteine and glycine-rich protein 3 OS=Homo sapiens GN=CSRP3 PE=1 SV=1	GIGYGQGAGC[160.0307]LSTDTGEHLGLQFQQS[166.9984]PKPAR	-1.13	7.8E-01	-1.05	8.5E-01	-1.18	6.6E-01
CSRP3_HUMAN	Cysteine and glycine-rich protein 3 OS=Homo sapiens GN=CSRP3 PE=1 SV=1	S[166.9984]LESTNVTDKDGELYC[160.0307]K	-1.12	9.9E-01	-1.10	7.0E-01	-1.23	7.7E-01
CYC_HUMAN	Cytochrome c - Homo sapiens (Human)	KTGQAPGYS[166.9984]YTAANK	1.05	9.4E-01	-1.41	6.5E-01	-1.35	8.3E-01
CYC_HUMAN	Cytochrome c - Homo sapiens (Human)	KTGQAPGYSYT[181.014]AANK	1.78	1.7E-01	-1.84	1.3E-01	-1.03	9.8E-01
CYC_HUMAN	Cytochrome c - Homo sapiens (Human)	TGQAPGYS[166.9984]YTAANK	1.33	4.0E-01	-2.60	2.0E-03	-1.95	7.0E-01
DESM_HUMAN	Desmin - Homo sapiens (Human)	TFGGAPGFPLGS[166.9984]PLSS[166.9984]PVFPR	1.50	2.5E-01	-1.18	8.3E-01	1.27	6.2E-01
DESM_HUMAN	Desmin - Homo sapiens (Human)	TFGGAPGFPLGS[166.9984]PLSSPVFPR	2.16	3.5E-01	-1.25	9.7E-01	1.72	1.5E-01
DESM_HUMAN	Desmin - Homo sapiens (Human)	TFGGAPGFPLGSPLSS[166.9984]PVFPR	1.41	7.7E-01	1.82	3.6E-01	2.55	6.2E-02
DSG2_HUMAN	Desmoglein-2 OS=Homo sapiens GN=DSG2 PE=1 SV=2	VVPSFLPVDQGGS[166.9984]LVGR	1.77	5.6E-01	-2.81	1.8E-01	-1.58	6.4E-01
DSG2_HUMAN	Desmoglein-2 OS=Homo sapiens GN=DSG2 PE=1 SV=2	WEEHRS[166.9984]LLSGR	2.33	1.7E-01	-2.29	2.1E-01	1.02	8.7E-01
EF1B_HUMAN	Elongation factor 1-beta - Homo sapiens (Human)	YGPADVEDTTGSGATDSKDDDDIDLFGS[166.9984]DDEEESEEAKR	1.08	9.1E-01	-1.68	3.3E-01	-1.56	4.0E-01
FBN1_HUMAN	Fibrillin-1 OS=Homo sapiens GN=FBN1 PE=1 SV=1	GNPEPPVS[166.9984]GEM[147.0354]DDNSLSPEAC[160.0307]YEC[160.0307]K	3.80	7.4E-04	-2.39	7.3E-02	1.59	6.4E-01
FETUA_HUMAN	Alpha-2-HS-glycoprotein precursor - Homo sapiens (Human)	C[160.0307]DSSPDS[166.9984]AEDVR	-2.17	3.7E-01	3.48	1.3E-01	1.60	7.4E-01
FETUA_HUMAN	Alpha-2-HS-glycoprotein precursor - Homo sapiens (Human)	C[160.0307]DSSPDS[166.9984]AEDVRK	-2.09	3.9E-01	4.22	3.1E-02	2.02	5.4E-01
FETUA_HUMAN	Alpha-2-HS-glycoprotein precursor - Homo sapiens (Human)	HTFM[147.0354]GVVSLGSPS[166.9984]GEVSHPR	2.41	9.2E-01	2.14	3.5E-01	5.15	5.9E-02
FETUA_HUMAN	Alpha-2-HS-glycoprotein precursor - Homo sapiens (Human)	HTFMGVVSLGSPS[166.9984]GEVSHPR	-2.32	7.8E-01	20.09	2.3E-11	8.67	2.4E-06
FETUA_HUMAN	Alpha-2-HS-glycoprotein precursor - Homo sapiens (Human)	HTFMGVVSLGSPSGEVS[166.9984]HPR	-1.88	7.6E-01	7.95	7.2E-04	4.24	1.1E-01
FHL2_HUMAN	Four and a half LIM domains protein 2 OS=Homo sapiens GN=FHL2 PE=1 SV=3	YIS[166.9984]FEER	-3.34	1.3E-02	-1.04	9.2E-01	-3.49	3.5E-02
FRIH_HUMAN	Ferritin heavy chain OS=Homo sapiens GN=FTH1 PE=1 SV=2	KMGAPESGLAEYLFDKHTLGDS[166.9984]DNES	-1.39	7.1E-01	-1.17	9.6E-01	-1.63	6.7E-01
G3P_HUMAN	Glyceraldehyde-3-phosphate dehydrogenase - Homo sapiens (Human)	GALQNIIPAS[166.9984]TGAAK	-1.32	6.3E-01	-1.35	7.7E-01	-1.79	1.4E-01
G3P_HUMAN	Glyceraldehyde-3-phosphate dehydrogenase - Homo sapiens (Human)	IISNASC[160.0307]T[181.014]TNC[160.0307]LAPLAK	-1.27	8.0E-01	1.17	8.1E-01	-1.09	9.5E-01
G3P_HUMAN	Glyceraldehyde-3-phosphate dehydrogenase - Homo sapiens (Human)	VIHDNFGIVEGLM[147.0354]TTVHAITAT[181.014]QK	-1.16	5.3E-01	-1.61	9.2E-01	-1.86	3.2E-01
G3P_HUMAN	Glyceraldehyde-3-phosphate dehydrogenase - Homo sapiens (Human)	VIHDNFGIVEGLMTTVHAITAT[181.014]QK	-1.56	6.7E-01	1.78	4.9E-01	1.14	9.0E-01
H12_HUMAN	Histone H1.2 - Homo sapiens (Human)	S[166.9984]ETAPAAPAAAPPAEK	1.31	8.6E-01	-1.56	6.0E-01	-1.20	7.7E-01
H31_HUMAN	Histone H3.1 OS=Homo sapiens GN=HIST1H3A PE=1 SV=2	ST[181.014]ELLIR	1.39	8.0E-01	-2.13	2.9E-01	-1.53	6.5E-01
HNRPD_HUMAN	Heterogeneous nuclear ribonucleoprotein D0 - Homo sapiens (Human)	IDASKNEEDEGHSNS[166.9984]SPR	1.86	1.5E-01	-1.43	4.3E-01	1.30	6.7E-01
HNRPD_HUMAN	Heterogeneous nuclear ribonucleoprotein D0 - Homo sapiens (Human)	IDASKNEEDEGHSNSS[166.9984]PR	1.60	5.2E-01	-1.28	9.4E-01	1.25	9.8E-02
HP1B3_HUMAN	Heterochromatin protein 1-binding protein 3 - Homo sapiens (Human)	TVNSTRET[181.014]PPK	1.45	5.8E-01	-1.52	4.3E-01	-1.05	9.0E-01
HS90A_HUMAN	Heat shock protein HSP 90-alpha - Homo sapiens (Human)	ES[166.9984]EDKPEIEDVGSDEEEEKK	-3.42	1.7E-04	3.74	6.5E-06	1.09	9.0E-01
HS90A_HUMAN	Heat shock protein HSP 90-alpha - Homo sapiens (Human)	ES[166.9984]EDKPEIEDVGSDEEEEKKDGDK	-1.09	7.1E-01	1.80	8.6E-02	1.65	3.2E-01
HS90A_HUMAN	Heat shock protein HSP 90-alpha - Homo sapiens (Human)	ESEDKPEIEDVGS[166.9984]DEEEEKKDGDK	1.07	9.8E-01	1.24	6.8E-01	1.33	5.3E-01
HS90B_HUMAN	Heat shock protein HSP 90-beta - Homo sapiens (Human)	IEDVGS[166.9984]DEEDDSGK	2.71	8.5E-02	2.76	4.3E-01	7.48	6.0E-03
HS90B_HUMAN	Heat shock protein HSP 90-beta - Homo sapiens (Human)	IEDVGS[166.9984]DEEDDSGKDK	1.29	9.1E-01	-1.03	9.9E-01	1.25	8.9E-01
HS90B_HUMAN	Heat shock protein HSP 90-beta - Homo sapiens (Human)	IEDVGS[166.9984]DEEDDSGKDKK	1.55	4.6E-01	-1.27	8.1E-01	1.22	7.2E-01
HSPB1_HUMAN	Heat shock protein beta-1 - Homo sapiens (Human)	GPS[166.9984]WDPFR	-4.01	1.5E-04	1.76	4.8E-01	-2.28	7.2E-02
HSPB1_HUMAN	Heat shock protein beta-1 - Homo sapiens (Human)	GPS[166.9984]WDPFRDWYPHSR	-3.32	5.1E-06	-1.07	8.0E-01	-3.56	1.4E-04
HSPB1_HUMAN	Heat shock protein beta-1 - Homo sapiens (Human)	QLS[166.9984]SGVSEIR	-3.99	3.6E-01	4.53	7.7E-04	1.14	6.1E-01
HSPB7_HUMAN	Heat shock protein beta-7 OS=Homo sapiens GN=HSPB7 PE=1 SV=1	AERS[166.9984]FHSSSSSSSSSTSSSASR	-2.80	8.8E-02	3.83	5.2E-02	1.36	8.4E-01
HSPB7_HUMAN	Heat shock protein beta-7 OS=Homo sapiens GN=HSPB7 PE=1 SV=1	AERSFHSSSSSSSSS[166.9984]TSSSASR	-3.08	1.4E-01	5.03	1.0E-01	1.63	8.3E-01
HSPB7_HUMAN	Heat shock protein beta-7 OS=Homo sapiens GN=HSPB7 PE=1 SV=1	S[166.9984]FHSSSSSSSSSTSSSASR	-3.46	6.0E-03	2.83	5.1E-02	-1.22	8.2E-01
HSPB7_HUMAN	Heat shock protein beta-7 OS=Homo sapiens GN=HSPB7 PE=1 SV=1	SFHS[166.9984]S[166.9984]SSSSSSSTSSSASR	-3.20	1.5E-01	2.51	3.6E-01	-1.27	8.8E-01
HSPB7_HUMAN	Heat shock protein beta-7 OS=Homo sapiens GN=HSPB7 PE=1 SV=1	SFHS[166.9984]SSSSSSSSTSSSASR	-2.56	2.0E-03	2.27	1.7E-01	-1.13	6.9E-01
ICAL_HUMAN	Calpastatin OS=Homo sapiens GN=CAST PE=1 SV=4	EGITGPPADSSKPIGPDDAIDALSSDFTC[160.0307]GS[166.9984]PTAAGK	-1.07	9.2E-01	-1.86	2.9E-01	-2.00	6.6E-01
ICAL_HUMAN	Calpastatin OS=Homo sapiens GN=CAST PE=1 SV=4	EGITGPPADSSKPIGPDDAIDALSSDFTC[160.0307]GSPT[181.014]AAGK	-1.16	9.7E-01	-1.63	3.6E-01	-1.89	6.7E-01
ICAL_HUMAN	Calpastatin OS=Homo sapiens GN=CAST PE=1 SV=4	KEGITGPPADSSKPIGPDDAIDALSSDFTC[160.0307]GS[166.9984]PTAAGK	1.20	6.7E-01	-1.54	4.4E-01	-1.28	9.8E-01
KAD1_HUMAN	Adenylate kinase isoenzyme 1 OS=Homo sapiens GN=AK1 PE=1 SV=3	KVNAEGS[166.9984]VDSVFSQVC[160.0307]THLDALK	1.46	6.7E-01	-1.61	6.0E-01	-1.10	9.0E-01
KAD1_HUMAN	Adenylate kinase isoenzyme 1 OS=Homo sapiens GN=AK1 PE=1 SV=3	VNAEGS[166.9984]VDSVFSQVC[160.0307]THLDALK	-1.06	9.4E-01	-1.01	9.6E-01	-1.08	9.6E-01
KAD1_HUMAN	Adenylate kinase isoenzyme 1 OS=Homo sapiens GN=AK1 PE=1 SV=3	YGYTHLS[166.9984]TGDLLR	1.58	5.7E-01	-2.00	3.1E-01	-1.27	7.4E-01
KCRM_HUMAN	Creatine kinase M-type OS=Homo sapiens GN=CKM PE=1 SV=2	GQS[166.9984]IDDMIPAQK	-1.19	9.4E-01	-2.11	3.9E-01	-2.52	3.8E-01
KCRM_HUMAN	Creatine kinase M-type OS=Homo sapiens GN=CKM PE=1 SV=2	GTGGVDTAAVGS[166.9984]VFDVSNADR	-2.11	3.7E-02	-1.79	5.8E-01	-3.77	8.4E-05
KCRM_HUMAN	Creatine kinase M-type OS=Homo sapiens GN=CKM PE=1 SV=2	RGT[181.014]GGVDTAAVGSVFDVSNADR	-1.24	8.5E-01	-2.04	2.7E-01	-2.52	5.9E-02
KCRM_HUMAN	Creatine kinase M-type OS=Homo sapiens GN=CKM PE=1 SV=2	RGTGGVDTAAVGS[166.9984]VFDVSNADR	-1.42	4.6E-01	-3.67	5.0E-03	-5.20	1.5E-08
KCRS_HUMAN	Creatine kinase, sarcomeric mitochondrial OS=Homo sapiens GN=CKMT2 PE=1 SV=2	LGYILTC[160.0307]PS[166.9984]NLGTGLR	1.30	9.4E-01	-2.28	6.8E-01	-1.76	4.6E-01
KNG1_HUMAN	Kininogen-1 OS=Homo sapiens GN=KNG1 PE=1 SV=2	ETTC[160.0307]S[166.9984]KESNEELTESC[160.0307]ETK	-1.30	1.4E-01	2.52	1.0E-03	1.93	6.2E-02
KNG1_HUMAN	Kininogen-1 OS=Homo sapiens GN=KNG1 PE=1 SV=2	ETTC[160.0307]S[166.9984]KESNEELTESC[160.0307]ETKK	-1.05	9.6E-01	2.47	5.3E-02	2.36	1.4E-01
KNG1_HUMAN	Kininogen-1 OS=Homo sapiens GN=KNG1 PE=1 SV=2	ETTC[160.0307]SKES[166.9984]NEELTESC[160.0307]ETK	-1.39	6.3E-01	4.07	3.3E-05	2.93	2.4E-02
KNG1_HUMAN	Kininogen-1 OS=Homo sapiens GN=KNG1 PE=1 SV=2	ETTC[160.0307]SKES[166.9984]NEELTESC[160.0307]ETKK	-1.10	7.0E-01	2.16	8.0E-03	1.96	5.4E-02
LU_HUMAN	Lutheran blood group glycoprotein precursor - Homo sapiens (Human)	GAPPPGEPGLS[166.9984]HSGSEQPEQTGLLM[147.0354]GGASGGAR	1.18	9.6E-01	-1.00	8.4E-01	1.18	8.8E-01
LU_HUMAN	Lutheran blood group glycoprotein precursor - Homo sapiens (Human)	GAPPPGEPGLSHS[166.9984]GSEQPEQTGLLMGGASGGAR	-2.76	1.7E-01	1.23	9.6E-01	-2.24	4.3E-01
MARCS_HUMAN	Myristoylated alanine-rich C-kinase substrate - Homo sapiens (Human)	AEDGATPSPSNET[181.014]PK	1.84	4.5E-01	1.75	2.4E-01	3.23	2.0E-03
MARCS_HUMAN	Myristoylated alanine-rich C-kinase substrate - Homo sapiens (Human)	AEDGATPSPSNET[181.014]PKK	1.61	2.0E-07	1.62	2.9E-02	2.61	1.8E-01
MARCS_HUMAN	Myristoylated alanine-rich C-kinase substrate - Homo sapiens (Human)	EAPAEGEAAEPGS[166.9984]PTAAEGEAASAASSTSSPK	-1.99	3.1E-01	1.39	6.3E-01	-1.43	7.5E-01
MARCS_HUMAN	Myristoylated alanine-rich C-kinase substrate - Homo sapiens (Human)	GEPAAAAAPEAGAS[166.9984]PVEK	-2.16	4.5E-01	2.66	6.1E-02	1.23	7.7E-01
MARCS_HUMAN	Myristoylated alanine-rich C-kinase substrate - Homo sapiens (Human)	LSGFS[166.9984]FK	2.15	3.2E-01	-1.00	8.6E-01	2.15	4.4E-02
MARCS_HUMAN	Myristoylated alanine-rich C-kinase substrate - Homo sapiens (Human)	LSGFS[166.9984]FKK	1.89	2.4E-01	-1.27	7.4E-01	1.50	2.9E-01
MLRV_HUMAN	Myosin regulatory light chain 2, ventricular/cardiac muscle isoform OS=Homo sapiens GN=MYL2 PE=1 SV=3	AGGANS[166.9984]NVFSM[147.0354]FEQTQIQEFK	2.86	3.4E-01	-1.86	7.5E-01	1.53	4.1E-01
MLRV_HUMAN	Myosin regulatory light chain 2, ventricular/cardiac muscle isoform OS=Homo sapiens GN=MYL2 PE=1 SV=3	AGGANS[166.9984]NVFSMFEQTQIQEFK	-3.53	6.0E-03	1.70	7.8E-01	-2.08	8.8E-02
MLRV_HUMAN	Myosin regulatory light chain 2, ventricular/cardiac muscle isoform OS=Homo sapiens GN=MYL2 PE=1 SV=3	RAGGANS[166.9984]NVFSM[147.0354]FEQTQIQEFK	3.01	2.0E-01	-1.98	5.5E-01	1.52	7.0E-01
MLRV_HUMAN	Myosin regulatory light chain 2, ventricular/cardiac muscle isoform OS=Homo sapiens GN=MYL2 PE=1 SV=3	RAGGANS[166.9984]NVFSMFEQTQIQEFK	-2.29	3.5E-01	1.88	5.6E-01	-1.22	8.8E-01
MYOZ2_HUMAN	Myozenin-2 OS=Homo sapiens GN=MYOZ2 PE=1 SV=1	DIMLEELSHLS[166.9984]NR	-1.26	9.5E-01	1.55	1.8E-01	1.23	4.3E-01
MYOZ2_HUMAN	Myozenin-2 OS=Homo sapiens GN=MYOZ2 PE=1 SV=1	S[166.9984]PPNPDNIAPGYSGPLK	1.20	5.4E-01	-1.48	2.4E-01	-1.23	4.5E-01
MYOZ2_HUMAN	Myozenin-2 OS=Homo sapiens GN=MYOZ2 PE=1 SV=1	VDGSNLEGGS[166.9984]QQAPLTPPNTPDPR	1.60	8.5E-01	-1.22	1.0E+00	1.31	8.2E-01
MYOZ2_HUMAN	Myozenin-2 OS=Homo sapiens GN=MYOZ2 PE=1 SV=1	VDGSNLEGGSQQAPLT[181.014]PPNT[181.014]PDPR	-1.53	1.8E-01	3.02	2.5E-04	1.97	3.0E-03
MYOZ2_HUMAN	Myozenin-2 OS=Homo sapiens GN=MYOZ2 PE=1 SV=1	VDGSNLEGGSQQAPLT[181.014]PPNTPDPR	-1.03	9.8E-01	-1.24	4.5E-01	-1.28	2.2E-01
MYPT1_HUMAN	Protein phosphatase 1 regulatory subunit 12A OS=Homo sapiens GN=PPP1R12A PE=1 SV=1	KTGS[166.9984]YGALAEITASK	1.21	7.8E-01	1.21	9.1E-01	1.46	6.7E-01
MYPT1_HUMAN	Protein phosphatase 1 regulatory subunit 12A OS=Homo sapiens GN=PPP1R12A PE=1 SV=1	KTGSY[243.0297]GALAEITASK	1.41	7.0E-01	1.31	6.5E-01	1.84	1.9E-01
MYPT1_HUMAN	Protein phosphatase 1 regulatory subunit 12A OS=Homo sapiens GN=PPP1R12A PE=1 SV=1	RS[166.9984]TQGVTLTDLQEAEK	-2.68	2.6E-02	1.25	6.5E-01	-2.15	1.1E-01
MYPT1_HUMAN	Protein phosphatase 1 regulatory subunit 12A OS=Homo sapiens GN=PPP1R12A PE=1 SV=1	RST[181.014]QGVTLTDLQEAEK	-1.92	3.2E-01	1.20	8.2E-01	-1.60	4.3E-01
MYPT1_HUMAN	Protein phosphatase 1 regulatory subunit 12A OS=Homo sapiens GN=PPP1R12A PE=1 SV=1	S[166.9984]YLTPVRDEESESQR	-1.02	8.9E-01	1.28	7.5E-01	1.25	8.1E-01
MYPT2_HUMAN	Protein phosphatase 1 regulatory subunit 12B OS=Homo sapiens GN=PPP1R12B PE=1 SV=2	DEDET[181.014]DGSEEVKETWHER	2.24	5.9E-01	-3.41	1.7E-01	-1.52	3.4E-01
MYPT2_HUMAN	Protein phosphatase 1 regulatory subunit 12B OS=Homo sapiens GN=PPP1R12B PE=1 SV=2	S[166.9984]LDEEPIC[160.0307]HR	-2.20	1.1E-01	-1.37	6.5E-01	-3.03	4.0E-03
NCAM1_HUMAN	Neural cell adhesion molecule 1 OS=Homo sapiens GN=NCAM1 PE=1 SV=3	AAFSKDES[166.9984]KEPIVEVR	-1.09	9.0E-01	-1.66	5.8E-01	-1.81	3.9E-01
NEBL_HUMAN	Nebulette OS=Homo sapiens GN=NEBL PE=1 SV=1	TDPGS[166.9984]IFDLDPLEDNIQSR	1.58	7.1E-01	1.75	6.5E-01	2.78	1.8E-01
NEXN_HUMAN	Nexilin OS=Homo sapiens GN=NEXN PE=1 SV=1	EM[147.0354]LAS[166.9984]DDEEDVSSK	5.55	1.5E-01	-5.85	1.8E-01	-1.05	7.2E-01
NEXN_HUMAN	Nexilin OS=Homo sapiens GN=NEXN PE=1 SV=1	EM[147.0354]LAS[166.9984]DDEEDVSSKVEK	13.46	5.3E-04	-16.05	3.1E-04	-1.19	6.9E-01
NEXN_HUMAN	Nexilin OS=Homo sapiens GN=NEXN PE=1 SV=1	TIS[166.9984]QEFLTPGK	2.79	1.4E-01	-1.15	9.2E-01	2.42	2.0E-03
NP1L4_HUMAN	Nucleosome assembly protein 1-like 4 OS=Homo sapiens GN=NAP1L4 PE=1 SV=1	EFITGDVEPTDAESEWHS[166.9984]ENEEEEK	-1.12	5.7E-01	1.44	3.9E-01	1.28	5.9E-01
NP1L4_HUMAN	Nucleosome assembly protein 1-like 4 OS=Homo sapiens GN=NAP1L4 PE=1 SV=1	EFITGDVEPTDAESEWHS[166.9984]ENEEEEKLAGDMK	-1.46	8.8E-01	1.36	7.7E-01	-1.07	9.6E-01
OCAD1_HUMAN	OCIA domain-containing protein 1 OS=Homo sapiens GN=OCIAD1 PE=1 SV=1	RSS[166.9984]PPGHYYQK	3.33	1.4E-02	-3.33	5.1E-02	-1.00	9.1E-01
ODPA_HUMAN	ODPA_HUMAN	YGM[147.0354]GT[181.014]SVER	123.00	6.0E-14	-61.57	8.9E-14	2.00	7.0E-01
ODPA_HUMAN	Pyruvate dehydrogenase E1 component subunit alpha, somatic form, mitochondrial OS=Homo sapiens GN=PDHA1 PE=1 SV=3	YHGHS[166.9984]M[147.0354]SDPGVS[166.9984]YR	32.46	1.8E-10	-32.49	2.3E-11	-1.00	9.4E-01
ODPA_HUMAN	Pyruvate dehydrogenase E1 component subunit alpha, somatic form, mitochondrial OS=Homo sapiens GN=PDHA1 PE=1 SV=3	YHGHS[166.9984]M[147.0354]SDPGVSY[243.0297]R	25.87	1.1E-13	-27.30	6.0E-14	-1.06	9.1E-01
ODPA_HUMAN	Pyruvate dehydrogenase E1 component subunit alpha, somatic form, mitochondrial OS=Homo sapiens GN=PDHA1 PE=1 SV=3	YHGHS[166.9984]M[147.0354]SDPGVSYR	1.16	5.7E-01	-1.15	6.5E-01	1.01	9.4E-01
ODPA_HUMAN	Pyruvate dehydrogenase E1 component subunit alpha, somatic form, mitochondrial OS=Homo sapiens GN=PDHA1 PE=1 SV=3	YHGHS[166.9984]MSDPGVSYR	-2.20	5.9E-01	1.89	6.4E-01	-1.17	9.5E-01
ODPA_HUMAN	Pyruvate dehydrogenase E1 component subunit alpha, somatic form, mitochondrial OS=Homo sapiens GN=PDHA1 PE=1 SV=3	YHGHSMS[166.9984]DPGVSYR	-1.64	9.0E-01	1.56	8.4E-01	-1.05	9.7E-01
PEBP1_HUMAN	Phosphatidylethanolamine-binding protein 1 - Homo sapiens (Human)	NRPTS[166.9984]ISWDGLDSGK	-1.00	1.0E+00	-1.14	7.4E-01	-1.14	7.3E-01
PEBP1_HUMAN	Phosphatidylethanolamine-binding protein 1 - Homo sapiens (Human)	NRPTSIS[166.9984]WDGLDSGK	-1.01	9.8E-01	-1.96	3.2E-01	-1.99	1.5E-01
PGRC2_HUMAN	Membrane-associated progesterone receptor component 2 - Homo sapiens (Human)	LLKPGEEPS[166.9984]EYTDEEDTKDHNKQD	1.32	1.6E-01	-1.22	4.0E-01	1.08	9.3E-01
PGRC2_HUMAN	Membrane-associated progesterone receptor component 2 - Homo sapiens (Human)	LLKPGEEPSEYT[181.014]DEEDTK	-1.17	8.7E-01	1.25	8.0E-01	1.07	9.5E-01
PGRC2_HUMAN	Membrane-associated progesterone receptor component 2 - Homo sapiens (Human)	LLKPGEEPSEYT[181.014]DEEDTKDHNK	-1.53	4.5E-01	1.46	6.3E-01	-1.05	9.0E-01
PGRC2_HUMAN	Membrane-associated progesterone receptor component 2 - Homo sapiens (Human)	LLKPGEEPSEYT[181.014]DEEDTKDHNKQD	1.15	4.1E-01	-1.13	5.9E-01	1.02	9.8E-01
POPD1_HUMAN	Blood vessel epicardial substance OS=Homo sapiens GN=BVES PE=2 SV=1	GTSS[166.9984]MS[166.9984]SLHVSSPHQR	-8.02	1.2E-02	2.03	9.4E-01	-3.96	1.7E-01
POPD1_HUMAN	Blood vessel epicardial substance OS=Homo sapiens GN=BVES PE=2 SV=1	GTSSM[147.0354]S[166.9984]SLHVSSPHQR	1.68	9.4E-01	-2.65	7.5E-01	-1.58	7.5E-01
POPD1_HUMAN	Blood vessel epicardial substance OS=Homo sapiens GN=BVES PE=2 SV=1	GTSSMSS[166.9984]LHVSSPHQR	-5.94	8.4E-02	1.35	7.0E-01	-4.40	1.3E-01
POPD1_HUMAN	Blood vessel epicardial substance OS=Homo sapiens GN=BVES PE=2 SV=1	M[147.0354]KPIEEGAEDDDDVFEPAS[166.9984]PNTLK	2.59	5.4E-01	-2.02	7.0E-01	1.28	8.9E-01
POPD1_HUMAN	Blood vessel epicardial substance OS=Homo sapiens GN=BVES PE=2 SV=1	MKPIEEGAEDDDDVFEPAS[166.9984]PNTLK	-1.69	7.8E-01	1.37	9.2E-01	-1.24	9.1E-01
POPD1_HUMAN	Blood vessel epicardial substance OS=Homo sapiens GN=BVES PE=2 SV=1	NS[166.9984]IASSSDSDDGLHQFLR	-10.06	6.0E-14	1.30	1.0E+00	-7.72	7.9E-11
POPD1_HUMAN	Blood vessel epicardial substance OS=Homo sapiens GN=BVES PE=2 SV=1	NSIASSS[166.9984]DSDDGLHQFLR	1.59	5.8E-01	-2.23	8.0E-02	-1.40	1.6E-01
POPD1_HUMAN	Blood vessel epicardial substance OS=Homo sapiens GN=BVES PE=2 SV=1	NSIASSSDS[166.9984]DDGLHQFLR	1.41	5.6E-01	-1.71	3.9E-01	-1.21	7.2E-01
PTRF_HUMAN	Polymerase I and transcript release factor OS=Homo sapiens GN=PTRF PE=1 SV=1	ES[166.9984]EALPEKEGEELGEGERPEEDAAALELS[166.9984]SDEAVEVEEVIEESR	-1.12	1.0E+00	1.12	8.6E-01	-1.00	9.0E-01
PTRF_HUMAN	Polymerase I and transcript release factor OS=Homo sapiens GN=PTRF PE=1 SV=1	ES[166.9984]EALPEKEGEELGEGERPEEDAAALELSS[166.9984]DEAVEVEEVIEESR	-1.04	9.8E-01	-1.03	9.7E-01	-1.06	1.0E+00
PTRF_HUMAN	Polymerase I and transcript release factor OS=Homo sapiens GN=PTRF PE=1 SV=1	KVS[166.9984]VNVK	2.15	2.2E-01	-2.38	2.3E-01	-1.11	9.4E-01
PTRF_HUMAN	Polymerase I and transcript release factor OS=Homo sapiens GN=PTRF PE=1 SV=1	LPAKLS[166.9984]ISK	1.27	1.3E-01	-1.18	6.5E-01	1.08	8.6E-01
PTRF_HUMAN	Polymerase I and transcript release factor OS=Homo sapiens GN=PTRF PE=1 SV=1	RGS[166.9984]S[166.9984]PDVHALLEITEESDAVLVDK	1.53	5.2E-01	-1.10	9.2E-01	1.38	5.8E-01

PTRF_HUMAN	Polymerase I and transcript release factor OS=Homo sapiens GN=PTRF PE=1 SV=1	S[166.9984]FTPDHVVYAR	1.08	8.9E-01	1.54	2.7E-01	1.67	1.1E-01
PTRF_HUMAN	Polymerase I and transcript release factor OS=Homo sapiens GN=PTRF PE=1 SV=1	S[166.9984]LKESEALPEK	-1.25	8.2E-01	1.39	7.0E-01	1.11	9.1E-01
PTRF_HUMAN	Polymerase I and transcript release factor OS=Homo sapiens GN=PTRF PE=1 SV=1	VM[147.0354]IYQDEVKLPAKLSIS[166.9984]K	2.28	6.3E-01	-2.22	6.5E-01	1.03	9.2E-01
QCR6_HUMAN	Cytochrome b-c1 complex subunit 6, mitochondrial precursor - Homo sapiens (Human)	S[166.9984]HTEEDC[160.0307]TEELFDFLHAR	-1.38	8.8E-01	-1.30	8.2E-01	-1.80	6.9E-01
QCR6_HUMAN	Cytochrome b-c1 complex subunit 6, mitochondrial precursor - Homo sapiens (Human)	SHT[181.014]EEDC[160.0307]TEELFDFLHAR	-1.08	9.8E-01	-1.81	4.6E-01	-1.95	7.0E-01
RLA2_HUMAN	60S acidic ribosomal protein P2 OS=Homo sapiens GN=RPLP2 PE=1 SV=1	KEES[166.9984]EES[166.9984]DDDM[147.0354]GFGLFD	3.57	5.3E-01	-5.06	3.0E-01	-1.42	8.5E-01
RLA2_HUMAN	60S acidic ribosomal protein P2 OS=Homo sapiens GN=RPLP2 PE=1 SV=1	KEES[166.9984]EES[166.9984]DDDMGFGLFD	-3.84	1.1E-01	1.35	9.4E-01	-2.84	3.8E-01
ROA3_HUMAN	Heterogeneous nuclear ribonucleoprotein A3 - Homo sapiens (Human)	SSGS[166.9984]PYGGGYGSGGGSGGYGSR	1.53	4.9E-01	-1.19	8.0E-01	1.29	7.4E-01
SDPR_HUMAN	Serum deprivation-response protein OS=Homo sapiens GN=SDPR PE=1 SV=3	EELPDENKSLEETLHT[181.014]VDLS[166.9984]SDDDLPHDEEALEDSAEEKVEESR	1.39	9.8E-01	1.21	2.7E-01	1.68	1.9E-01
SDPR_HUMAN	Serum deprivation-response protein OS=Homo sapiens GN=SDPR PE=1 SV=3	IS[166.9984]S[166.9984]GKS[166.9984]SPFKVSPLTFGR	1.42	8.3E-01	-1.22	9.2E-01	1.16	9.0E-01
SDPR_HUMAN	Serum deprivation-response protein OS=Homo sapiens GN=SDPR PE=1 SV=3	ISSGKS[166.9984]S[166.9984]PFKVS[166.9984]PLTFGR	1.26	1.0E+00	1.01	8.9E-01	1.27	8.7E-01
SDPR_HUMAN	Serum deprivation-response protein OS=Homo sapiens GN=SDPR PE=1 SV=3	S[166.9984]SPFKVS[166.9984]PLTFGR	1.04	9.2E-01	1.10	8.2E-01	1.14	7.7E-01
SDPR_HUMAN	Serum deprivation-response protein OS=Homo sapiens GN=SDPR PE=1 SV=3	SLEETLHTVDLS[166.9984]S[166.9984]DDDLPHDEEALEDS[166.9984]AEEKVEESR	-1.12	9.1E-01	1.21	6.3E-01	1.08	8.3E-01
SDPR_HUMAN	Serum deprivation-response protein OS=Homo sapiens GN=SDPR PE=1 SV=3	SLEETLHTVDLS[166.9984]S[166.9984]DDDLPHDEEALEDSAEEK	-1.65	6.3E-01	1.40	3.6E-01	-1.17	9.9E-01
SDPR_HUMAN	Serum deprivation-response protein OS=Homo sapiens GN=SDPR PE=1 SV=3	SLEETLHTVDLS[166.9984]S[166.9984]DDDLPHDEEALEDSAEEKVEESR	1.06	9.3E-01	1.31	3.2E-01	1.39	2.9E-01
SDPR_HUMAN	Serum deprivation-response protein OS=Homo sapiens GN=SDPR PE=1 SV=3	SLEETLHTVDLSS[166.9984]DDDLPHDEEALEDS[166.9984]AEEKVEESR	-1.02	9.5E-01	-1.12	1.0E+00	-1.13	9.4E-01
SDPR_HUMAN	Serum deprivation-response protein OS=Homo sapiens GN=SDPR PE=1 SV=3	SSPFKVS[166.9984]PLTFGR	1.46	5.8E-01	-2.42	8.2E-02	-1.66	5.7E-01
SDPR_HUMAN	Serum deprivation-response protein OS=Homo sapiens GN=SDPR PE=1 SV=3	VS[166.9984]PLTFGR	1.45	6.5E-01	-1.21	8.6E-01	1.20	7.3E-01
SRBS2_HUMAN	Sorbin and SH3 domain-containing protein 2 OS=Homo sapiens GN=SORBS2 PE=1 SV=3	DAS[166.9984]SPVPPPHVPPPVPPLRPR	2.26	4.0E-03	1.52	3.4E-01	3.44	2.6E-05
SRBS2_HUMAN	Sorbin and SH3 domain-containing protein 2 OS=Homo sapiens GN=SORBS2 PE=1 SV=3	DASS[166.9984]PVPPPHVPPPVPPLRPR	2.64	1.4E-02	2.64	2.4E-01	6.98	6.8E-04
SRBS2_HUMAN	Sorbin and SH3 domain-containing protein 2 OS=Homo sapiens GN=SORBS2 PE=1 SV=3	GAEDYPDPPIPHS[166.9984]YSSDR	-2.16	1.9E-01	3.77	2.9E-04	1.75	3.7E-01
SRBS2_HUMAN	Sorbin and SH3 domain-containing protein 2 OS=Homo sapiens GN=SORBS2 PE=1 SV=3	RKS[166.9984]EPAVGPPR	1.77	2.6E-02	1.25	8.0E-01	2.21	3.6E-02
SRBS2_HUMAN	Sorbin and SH3 domain-containing protein 2 OS=Homo sapiens GN=SORBS2 PE=1 SV=3	S[166.9984]EPAVGPPR	2.67	3.3E-01	2.68	4.4E-01	7.16	1.8E-02
SRBS2_HUMAN	Sorbin and SH3 domain-containing protein 2 OS=Homo sapiens GN=SORBS2 PE=1 SV=3	SFTSSS[166.9984]PS[166.9984]SPSR	5.55	3.7E-08	-1.69	2.9E-01	3.28	3.5E-02
SRBS2_HUMAN	Sorbin and SH3 domain-containing protein 2 OS=Homo sapiens GN=SORBS2 PE=1 SV=3	SFTSSSPS[166.9984]SPSR	1.86	8.1E-02	1.39	3.8E-01	2.60	2.0E-03
SRBS2_HUMAN	Sorbin and SH3 domain-containing protein 2 OS=Homo sapiens GN=SORBS2 PE=1 SV=3	SHS[166.9984]DNSPNAFK	-1.20	9.2E-01	1.25	8.9E-01	1.04	9.8E-01
SRBS2_HUMAN	Sorbin and SH3 domain-containing protein 2 OS=Homo sapiens GN=SORBS2 PE=1 SV=3	T[181.014]SPGRVDLPGSSTTLTK	3.15	1.0E-03	-1.07	8.4E-01	2.95	6.0E-03
SRBS2_HUMAN	Sorbin and SH3 domain-containing protein 2 OS=Homo sapiens GN=SORBS2 PE=1 SV=3	TSPGRVDLPGS[166.9984]STTLTK	3.70	4.8E-05	1.04	1.0E+00	3.85	4.2E-04
SRCH_HUMAN	Sarcoplasmic reticulum histidine-rich calcium-binding protein OS=Homo sapiens GN=HRC PE=2 SV=1	AEVGAPLS[166.9984]PDHS[166.9984]EEEEEEEEGLEEDEPR	1.81	2.2E-01	-1.65	3.7E-01	1.10	7.7E-01
SRCH_HUMAN	Sarcoplasmic reticulum histidine-rich calcium-binding protein OS=Homo sapiens GN=HRC PE=2 SV=1	AEVGAPLS[166.9984]PDHSEEEEEEEEGLEEDEPR	-1.16	5.7E-01	-1.00	8.5E-01	-1.16	3.9E-01
SRCH_HUMAN	Sarcoplasmic reticulum histidine-rich calcium-binding protein OS=Homo sapiens GN=HRC PE=2 SV=1	AEVGAPLSPDHS[166.9984]EEEEEEEEGLEEDEPR	-1.26	4.4E-01	1.09	9.8E-01	-1.16	4.5E-01
SRCH_HUMAN	Sarcoplasmic reticulum histidine-rich calcium-binding protein OS=Homo sapiens GN=HRC PE=2 SV=1	DDSEEEKEKEEDPGS[166.9984]HEEDDESSEQGEK	-1.32	6.7E-01	-1.02	9.6E-01	-1.34	7.0E-01
SRCH_HUMAN	Sarcoplasmic reticulum histidine-rich calcium-binding protein OS=Homo sapiens GN=HRC PE=2 SV=1	DEEEDEDVS[166.9984]TER	-1.30	8.0E-01	-1.11	9.2E-01	-1.44	6.6E-01
SRCH_HUMAN	Sarcoplasmic reticulum histidine-rich calcium-binding protein OS=Homo sapiens GN=HRC PE=2 SV=1	EEAGGASS[166.9984]EEESGEDTGPQDAQEYGNYQPGSLC[160.0307]GYC[160.0307]SFC[160.0307]NR	-1.37	4.4E-01	-1.20	8.1E-01	-1.64	2.2E-01
SRCH_HUMAN	Sarcoplasmic reticulum histidine-rich calcium-binding protein OS=Homo sapiens GN=HRC PE=2 SV=1	EEDEEVS[166.9984]AELGHQAPSHR	-2.20	7.0E-02	1.20	1.0E+00	-1.84	1.1E-01
SRCH_HUMAN	Sarcoplasmic reticulum histidine-rich calcium-binding protein OS=Homo sapiens GN=HRC PE=2 SV=1	EKEEDPGS[166.9984]HEEDDESSEQGEK	-3.18	4.0E-03	1.65	4.9E-01	-1.92	3.1E-01
SRCH_HUMAN	Sarcoplasmic reticulum histidine-rich calcium-binding protein OS=Homo sapiens GN=HRC PE=2 SV=1	GHDGEDDEGEEEEEEEEEEEEAS[166.9984]TEYGHQAHR	-2.57	2.8E-02	-1.36	6.7E-01	-3.50	4.0E-03
SRCH_HUMAN	Sarcoplasmic reticulum histidine-rich calcium-binding protein OS=Homo sapiens GN=HRC PE=2 SV=1	GHGS[166.9984]EEDEDVSDGHHHHGPSHR	-1.73	5.8E-01	1.09	6.3E-01	-1.58	3.7E-01
SRCH_HUMAN	Sarcoplasmic reticulum histidine-rich calcium-binding protein OS=Homo sapiens GN=HRC PE=2 SV=1	GHGSEDT[181.014]EDSAEHR	-1.66	1.0E+00	-2.62	9.2E-07	-4.36	1.1E-05
SRCH_HUMAN	Sarcoplasmic reticulum histidine-rich calcium-binding protein OS=Homo sapiens GN=HRC PE=2 SV=1	GHGSEDTEDS[166.9984]AEHR	-1.28	7.5E-01	-2.61	6.5E-01	-3.34	1.9E-01
SRCH_HUMAN	Sarcoplasmic reticulum histidine-rich calcium-binding protein OS=Homo sapiens GN=HRC PE=2 SV=1	GHKS[166.9984]DEEDFQDEYK	1.23	8.0E-01	-1.04	8.4E-01	1.18	9.5E-01
SRCH_HUMAN	Sarcoplasmic reticulum histidine-rich calcium-binding protein OS=Homo sapiens GN=HRC PE=2 SV=1	HQGHEEDDDDDDDDDDDDDDDDVS[166.9984]IEYR	-1.88	1.7E-01	1.17	8.5E-01	-1.61	3.3E-01
SRCH_HUMAN	Sarcoplasmic reticulum histidine-rich calcium-binding protein OS=Homo sapiens GN=HRC PE=2 SV=1	HQGHRDEEEDEDVS[166.9984]TER	-1.07	1.0E+00	-1.16	4.7E-01	-1.25	6.9E-01
SRCH_HUMAN	Sarcoplasmic reticulum histidine-rich calcium-binding protein OS=Homo sapiens GN=HRC PE=2 SV=1	HRS[166.9984]HEEDDNDDDDVS[166.9984]TEYGHQAHR	-1.04	9.4E-01	1.68	7.6E-01	1.63	7.2E-01
SRCH_HUMAN	Sarcoplasmic reticulum histidine-rich calcium-binding protein OS=Homo sapiens GN=HRC PE=2 SV=1	HRS[166.9984]HEEDDNDDDDVSTEYGHQAHR	-1.92	4.5E-01	1.17	9.7E-01	-1.64	4.3E-01
SRCH_HUMAN	Sarcoplasmic reticulum histidine-rich calcium-binding protein OS=Homo sapiens GN=HRC PE=2 SV=1	SHEEDDNDDDDVS[166.9984]TEYGHQAHR	-1.11	8.1E-01	-1.11	6.4E-01	-1.24	5.8E-01
SRCH_HUMAN	Sarcoplasmic reticulum histidine-rich calcium-binding protein OS=Homo sapiens GN=HRC PE=2 SV=1	VGDEGVS[166.9984]GEEVFAEHGGQAR	-1.31	6.3E-01	-2.26	7.0E-03	-2.97	3.0E-03
SRCH_HUMAN	Sarcoplasmic reticulum histidine-rich calcium-binding protein OS=Homo sapiens GN=HRC PE=2 SV=1	VPREEDEEVS[166.9984]AELGHQAPSHR	1.18	1.7E-01	-1.34	2.2E-01	-1.13	4.9E-01
TEBP_HUMAN	Prostaglandin E synthase 3 - Homo sapiens (Human)	DWEDDS[166.9984]DEDMSNFDR	-2.65	3.6E-01	1.78	8.0E-01	-1.49	7.9E-01
TEBP_HUMAN	Prostaglandin E synthase 3 - Homo sapiens (Human)	LNWLSVDFNNWKDWEDDS[166.9984]DEDM[147.0354]SNFDR	1.60	9.2E-01	-2.24	7.5E-01	-1.41	8.1E-01
TEBP_HUMAN	Prostaglandin E synthase 3 - Homo sapiens (Human)	LNWLSVDFNNWKDWEDDS[166.9984]DEDMSNFDR	-2.56	1.5E-01	1.60	5.0E-01	-1.60	6.9E-01
TELT_HUMAN	Telethonin OS=Homo sapiens GN=TCAP PE=1 SV=1	EEREDT[181.014]PIQLQELLALETALGGQC[160.0307]VDR	1.26	6.9E-01	1.07	9.3E-01	1.34	5.9E-01
TELT_HUMAN	Telethonin OS=Homo sapiens GN=TCAP PE=1 SV=1	SMS[166.9984]QEAQRG	-1.36	9.2E-01	-1.19	9.6E-01	-1.62	8.9E-01
TNNC1_HUMAN	Troponin C, slow skeletal and cardiac muscles OS=Homo sapiens GN=TNNC1 PE=1 SV=1	GKS[166.9984]EEELSDLFR	1.09	9.6E-01	-1.85	5.4E-01	-1.70	6.1E-01
TNNI3_HUMAN	Troponin I, cardiac muscle OS=Homo sapiens GN=TNNI3 PE=1 SV=3	RRS[166.9984]S[166.9984]NYR	-1.56	4.5E-01	-1.30	5.5E-01	-2.02	1.0E-01
TNNT2_HUMAN	Troponin T, cardiac muscle OS=Homo sapiens GN=TNNT2 PE=1 SV=3	ELWQS[166.9984]IYNLEAEKFDLQEK	-1.41	7.5E-01	-1.09	1.0E+00	-1.54	6.8E-01
TPIS_HUMAN	Triosephosphate isomerase - Homo sapiens (Human)	IIYGGS[166.9984]VTGATC[160.0307]K	-1.24	8.2E-01	-1.29	7.4E-01	-1.60	4.0E-01
TPIS_HUMAN	Triosephosphate isomerase - Homo sapiens (Human)	KQS[166.9984]LGELIGTLNAAK	2.69	4.9E-02	-2.17	2.2E-01	1.24	7.6E-01
TPM1_HUMAN	Tropomyosin alpha-1 chain - Homo sapiens (Human)	KLVIIES[166.9984]DLER	1.14	9.4E-01	-1.77	3.7E-01	-1.55	2.8E-01
TPM1_HUMAN	Tropomyosin alpha-1 chain - Homo sapiens (Human)	LVIIES[166.9984]DLERAEER	1.10	9.2E-01	-1.65	4.4E-01	-1.51	3.2E-01
TPM1_HUMAN	Tropomyosin alpha-1 chain - Homo sapiens (Human)	S[166.9984]IDDLEDELYAQK	-1.23	8.5E-01	-1.47	4.8E-01	-1.81	2.7E-01
TPM1_HUMAN	Tropomyosin alpha-1 chain OS=Homo sapiens GN=TPM1 PE=1 SV=2	AISEELDHALNDM[147.0354]TS[166.9984]I	2.39	2.4E-01	-2.79	1.4E-01	-1.16	8.1E-01
TPM1_HUMAN	Tropomyosin alpha-1 chain OS=Homo sapiens GN=TPM1 PE=1 SV=2	AISEELDHALNDMTS[166.9984]I	-1.89	5.7E-01	1.05	6.9E-01	-1.80	5.3E-01
TPM1_HUMAN	Tropomyosin alpha-1 chain OS=Homo sapiens GN=TPM1 PE=1 SV=2	ATDAEADVAS[166.9984]LNRR	1.45	6.8E-01	-2.24	2.7E-01	-1.54	5.6E-01
TPM1_HUMAN	Tropomyosin alpha-1 chain OS=Homo sapiens GN=TPM1 PE=1 SV=2	KATDAEADVAS[166.9984]LNR	1.33	7.8E-01	-1.97	3.1E-01	-1.48	6.6E-01
TPM1_HUMAN	Tropomyosin alpha-1 chain OS=Homo sapiens GN=TPM1 PE=1 SV=2	KATDAEADVAS[166.9984]LNRR	1.49	8.4E-01	-2.00	5.9E-01	-1.34	7.1E-01
TPM1_HUMAN	Tropomyosin alpha-1 chain OS=Homo sapiens GN=TPM1 PE=1 SV=2	LAT[181.014]ALQK	-1.32	8.8E-01	-2.27	3.8E-01	-3.00	1.4E-01
TPM2_HUMAN	Tropomyosin beta chain - Homo sapiens (Human)	AISEELDNALNDITS[166.9984]L	1.12	7.1E-01	-1.35	1.8E-01	-1.20	6.9E-01
TPPP_HUMAN	Tubulin polymerization-promoting protein OS=Homo sapiens GN=TPPP PE=1 SV=1	AANRT[181.014]PPKSPGDPSK	1.05	1.2E-06	-2.47	2.1E-07	-2.36	1.4E-01
TPPP_HUMAN	Tubulin polymerization-promoting protein OS=Homo sapiens GN=TPPP PE=1 SV=1	AISS[166.9984]PTVSR	3.26	3.0E-02	-1.71	4.1E-01	1.91	7.1E-01
VDAC1_HUMAN	Voltage-dependent anion-selective channel protein 1 - Homo sapiens (Human)	VNNS[166.9984]SLIGLGYTQTLKPGIK	-1.30	8.0E-01	-1.89	1.6E-01	-2.44	1.4E-02
VDAC2_HUMAN	Voltage-dependent anion-selective channel protein 2 - Homo sapiens (Human)	LTFDTTFSPNT[181.014]GK	-1.12	9.8E-01	-1.15	8.9E-01	-1.29	8.7E-01
VDAC2_HUMAN	Voltage-dependent anion-selective channel protein 2 - Homo sapiens (Human)	VNNS[166.9984]SLIGVGYTQTLRPGVK	-1.30	6.8E-01	-1.87	1.9E-01	-2.43	1.4E-02
VIME_HUMAN	Vimentin - Homo sapiens (Human)	LRS[166.9984]SVPGVR	-1.31	8.8E-01	2.31	6.3E-01	1.76	6.8E-01
VIME_HUMAN	Vimentin - Homo sapiens (Human)	TYS[166.9984]LGSALRPSTSR	1.07	9.8E-01	4.16	2.1E-02	4.45	1.7E-02

This table illustrates the state dependent phosphorylation of statistically significant proteins common to the unenriched proteins identified by LC MS/MS. P-values were generated by ANOVA analysis of the peptide intensities quantified by LC MS/MS
